# Rare, protein-truncating variants in *ATM*, *CHEK2* and *PALB2*, but not *XRCC2*, are associated with increased breast cancer risks

**DOI:** 10.1136/jmedgenet-2017-104588

**Published:** 2017-08-04

**Authors:** Brennan Decker, Jamie Allen, Craig Luccarini, Karen A Pooley, Mitul Shah, Manjeet K Bolla, Qin Wang, Shahana Ahmed, Caroline Baynes, Don M Conroy, Judith Brown, Robert Luben, Elaine A Ostrander, Paul DP Pharoah, Alison M Dunning, Douglas F Easton

**Affiliations:** 1 Department of Public Health and Primary Care, Centre for Cancer Genetic Epidemiology, University of Cambridge, Cambridge, UK; 2 Cancer Genetics and Comparative Genomics Branch, National Human Genome Research Institute, National Institutes of Health, Bethesda, Maryland, USA; 3 Department of Oncology, Centre for Cancer Genetic Epidemiology, University of Cambridge, Cambridge, UK

**Keywords:** Cancer: breast, Genetic Epidemiology, Evidence Based Practice, Geneticscreening/counselling

## Abstract

**Background:**

Breast cancer (BC) is the most common malignancy in women and has a major heritable component. The risks associated with most rare susceptibility variants are not well estimated. To better characterise the contribution of variants in *ATM*, *CHEK2*, *PALB2* and *XRCC2*, we sequenced their coding regions in 13 087 BC cases and 5488 controls from East Anglia, UK.

**Methods:**

Gene coding regions were enriched via PCR, sequenced, variant called and filtered for quality. ORs for BC risk were estimated separately for carriers of truncating variants and of rare missense variants, which were further subdivided by functional domain and pathogenicity as predicted by four *in silico* algorithms.

**Results:**

Truncating variants in *PALB2* (OR=4.69, 95% CI 2.27 to 9.68), *ATM* (OR=3.26; 95% CI 1.82 to 6.46) and *CHEK2* (OR=3.11; 95% CI 2.15 to 4.69), but not *XRCC2* (OR=0.94; 95% CI 0.26 to 4.19) were associated with increased BC risk. Truncating variants in *ATM* and *CHEK2* were more strongly associated with risk of oestrogen receptor (ER)-positive than ER-negative disease, while those in *PALB2* were associated with similar risks for both subtypes. There was also some evidence that missense variants in *ATM*, *CHEK2* and *PALB2* may contribute to BC risk, but larger studies are necessary to quantify the magnitude of this effect.

**Conclusions:**

Truncating variants in *PALB2* are associated with a higher risk of BC than those in *ATM* or *CHEK2*. A substantial risk of BC due to truncating *XRCC2* variants can be excluded.

## Introduction

Breast cancer (BC) is the most common malignancy and the second leading cause of cancer deaths in women worldwide.^1^ Both twin and population-based family studies indicate a substantial heritable component to BC risk.^2^ To date, approximately half of familial risk of BC has been explained by a combination of common variants with small effect sizes,^3^ together with rarer, protein coding variants that confer higher risks.^4^ Truncating variants in some genes, including *BRCA1* and *BRCA2*, are associated with very high absolute risks, while deleterious variants in other genes, including *ATM*, *CHEK2* and *PALB2,* have been reported to confer more moderate risks.^4^ However, since susceptibility alleles in these genes are rare, the risks associated with these have not yet been well estimated. Variants in several other genes, including *XRCC2*, have been suggested to contribute to risk, but evidence is more equivocal.^4^

*ATM*, *CHEK2*, *PALB2* and *XRCC2* play important roles in DNA repair. The *ATM* gene encodes a protein kinase that recognises double stranded DNA breaks and initiates multiple aspects of the damage response cascade. A recent meta-analysis estimated that truncating *ATM* variants were associated with a relative risk of 2.8,^4^ and several studies have reported that certain subgroups of missense variants also contribute to BC risk.^5–9^ *CHEK2* encodes a checkpoint kinase that interacts with cell cycle regulators and DNA repair proteins. The most common protein-truncating genetic variant in Western European populations is the frame shift c.1100delC (p.Thr367MetfsTer15, rs555607708); a recent analysis by the Breast Cancer Association Consortium (BCAC) estimated a relative risk of 2.26 for this variant.^10^ Truncations of *PALB2,* the partner and localiser of *BRCA2*, have been associated with a fivefold relative risk,^4^ though studies in different populations have produced divergent estimates.^11–14^ *XRCC2* encodes a protein in the Rad51 family that participates in homologous recombination repair of double-stranded DNA breaks. Protein-truncating mutations in this gene are uncommon, but an association with BC risk has been suggested for rare missense variants.^15^

These four genes are now included in most multigene BC risk sequencing panels,^4^ and it is therefore critical for genetic counselling to have accurate estimates of the BC risk associated with variants in these genes. To provide such estimates, we undertook a large, population-based study in which we sequenced the protein coding exons and intron-exon boundaries of *ATM*, *CHEK2*, *PALB2* and *XRCC2* in 13 087 BC cases and 5488 controls. We evaluated sets of rare variants, defined by predicted functional effect, and more common individual variants (population frequency >0.1%), for association with BC risk.

## Materials and methods

### Study population

Cases were drawn from SEARCH, a population-based study of BC in the region of East Anglia (UK) covered by the Eastern Cancer Registration and Information Centre (ECRIC).^16^ The study enrolled subjects diagnosed before age 55 years with invasive BC from 1991 onwards and who were still alive at the start of the study in 1996 (prevalent cases, n=1087; median age=48 years), together with all patients diagnosed before age 70 years between 1996 and the present (n=12 000). Data on oestrogen-receptor and progesterone-receptor status and bilaterality were obtained through ECRIC and abstraction of medical records. Controls were drawn from three sources: (1) general practices participating in SEARCH who were frequency matched by age to the cases; (2) the European Prospective Investigation of Cancer (EPIC)-Norfolk study, a population-based cohort study of diet and health in Norfolk, East Anglia;^17^ and (3) women undergoing breast screening as part of the National Health Service Breast Screening Programme in screening centres in Cambridgeshire, who participated in the Sisters in Breast Screening study.^18^ Sequence analysis was conducted on samples from 13 824 BC cases and 5952 controls (pooled from the three studies above) of which 13 087 cases and 5488 controls passed all QC filters (see online supplementary figure s1 and supplementary methods) and were used in the analysis. Ethics approval was provided by the Cambridgeshire Research Ethics Committee, and written consent was obtained at the time of sample collection.

10.1136/jmedgenet-2017-104588.supp9 Supplementary data

### Amplicon design, enrichment, sequencing and variant calling

The Fluidigm Access Array 48.48 system was used for library preparation (see online supplementary methods). We designed 211 amplicons (see online supplementary table S1) to cover 98.1% of the bases within Consensus Coding Sequence (CCDS) exons of the four genes (see online supplementary table S2). Each library of 211 amplicons for 1536 samples was sequenced in 100-base paired-end mode on a single lane of an Illumina Hi-Seq2000. Raw sequence data were demultiplexed using the Illumina CASAVA 1.8 pipeline and aligned to the hg19 human reference sequence with BWA-MEM V.0.7.^19^ GATK UnifiedGenotyper was used to perform SNP and indel discovery and variant calling across all samples simultaneously (see online supplementary methods).^20–22^ After filtering samples and variants with >5% missing calls, 5488/5952 controls (92%) and 13 087/13 824 cases (95%) were retained for further analysis. Final variant calls were reproducible within this study and concordant with orthogonal methods (see online  supplementary table s3 and supplementary methods).

10.1136/jmedgenet-2017-104588.supp1 Supplementary data

10.1136/jmedgenet-2017-104588.supp2 Supplementary data

10.1136/jmedgenet-2017-104588.supp3 Supplementary data

### Functional prediction and variant frequency classification

The Ensembl Variant Effect Predictor^23^ was used to assign the canonical transcript-level and protein-level consequence for each genetic variant. Frameshift, stop/gain and canonical splice variants were grouped as protein truncating. Missense variants were further annotated with effect predictions from CADD,^24^ PolyPhen2,^25^ SIFT,^26^ and AlignGVGD.^27^ The consequences of the putative splice site variant *CHEK2* c.320–5T>A were evaluated using the *in silico* prediction tools SpliceSiteFinder-like,^28^ MaxEntScan,^29^ NNSPLICE,^30^ GeneSplicer^31^ and Human Splicing Finder.^32^

Variants detected in these four genes were annotated with allele frequencies observed in populations catalogued in the Exome Aggregation Consortium (ExAC) variation database (http://www.exac.broadinstitute.org).^33^ All coding variants with a carrier frequency >0.1% in the 33 370 non-Finnish, European ExAC subjects were classified as common, while all others (including those not reported by ExAC) were classified as rare. The combined East Anglian case and control frequency of <0.1% was used to define rare non-coding variants.

### Statistical analysis

For each variant with a carrier frequency >0.1% in ExAC European subjects (coding variants) or study cases and controls combined (non-coding variants), per-allele ORs and 95% CIs were computed, and Cochran-Armitage trend tests were carried out using the ‘prop.trend.test’ module of R.^34^

Since most variants were too rare to derive individual risk estimates, variants were grouped into classes (truncating, missense, synonymous and non-canonical splice) based on their predicted effect, and estimates were derived for carriers of any variant in each class. Rare missense variants were further subdivided based on domain, functional prediction scores and protein position. ORs were estimated by unconditional logistic regression, using the glm package in R. Profile likelihood-based confidence limits were derived using the confint routine. ORs were estimated using both the study controls (n=5488) and non-Finnish European ExAC subjects (n=33 370).

For truncating variants, separate analyses were conducted for oestrogen receptor (ER)-negative and ER-positive BC. Differences in ORs by subtype were assessed using case-only analyses.

## Results

### Spectrum of variation

**Figure 1:**
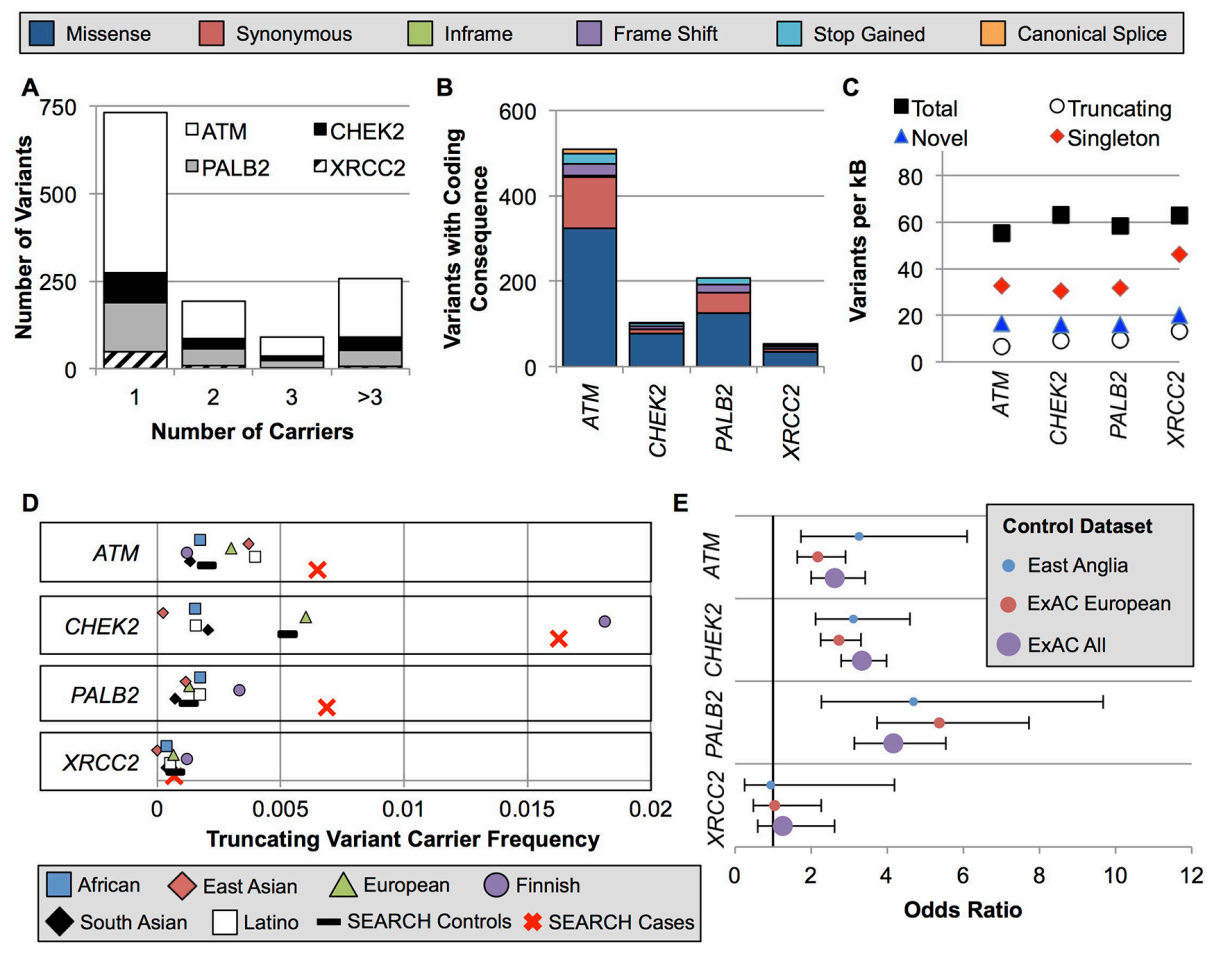
Spectrum of *ATM*, *CHEK2*, *PALB2* and *XRCC2* variation in ExAC populations and East Anglian cases and controls. (A) Nearly all variants were very rare or private. (B) The absolute number of variants per gene was highly divergent due to the difference in gene size. (C) However, when corrected for gene size, the genes had similar per-kB rates of total, truncating, singleton and novel variants not previously reported in the dbSNP, ExAC or COSMIC. (D) The carrier frequency for truncating variants in the study controls was near the mean of the ExAC populations. Notably, the ExAC Finnish population was an outlier for *CHEK2* and *PALB2* due to well-studied founder effects. (E) OR point estimates were similar using the study controls versus ExAC populations as controls. ExAC, Exome Aggregation Consortium.

We identified 1273 variants in the four genes: 785 in *ATM*, 165 in *CHEK2*, 255 in *PALB2* and 68 in *XRCC2* (online supplementary tables S4 and S5). Among these, 72 were common variants (for coding variants: carrier frequency >0.1% in ExAC non-Finnish, European ancestry subjects; for non-coding variants: carrier frequency >0.1% in cases and controls combined) (see online supplementary table S4). However, the majority of variants (731/1273, 57.4%) were identified in a single subject (figure 1A). Most variants encoded a missense substitution (figure 1B), and 29.1% of the coding variants identified in this study had not been previously reported in the dbSNP, ExAC or COSMIC databases (figure 1C). All four genes had similar variant rates after adjusting for gene length, with approximately 60 variants per kilobase of coding sequence (figure 1C).

### BC risks associated with *ATM*, *CHEK2* and *PALB2* truncating variants

**Figure 2:**
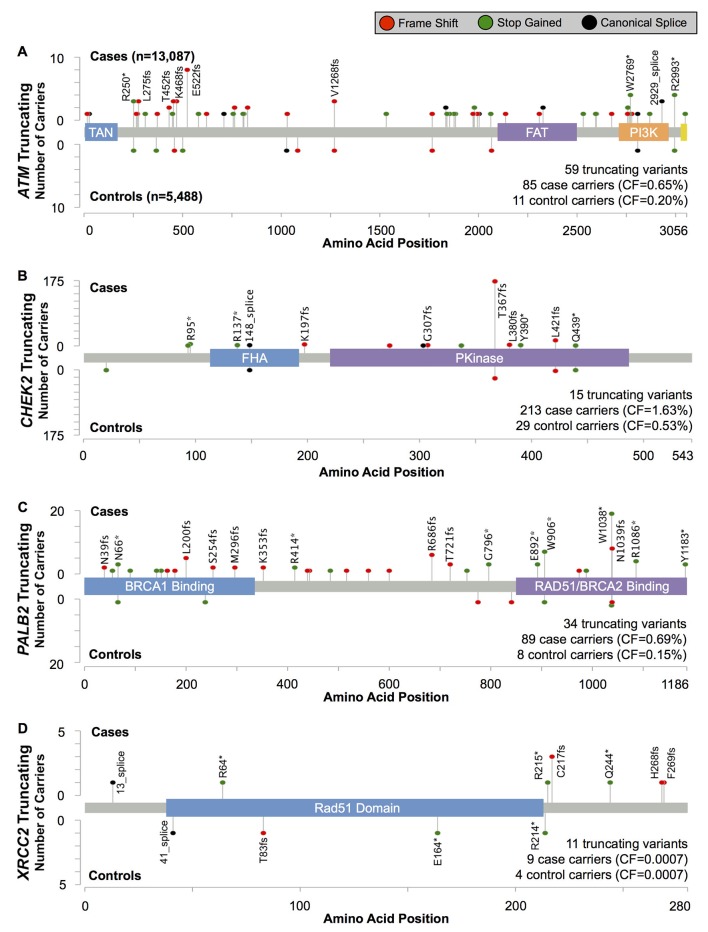
Position and frequency of protein-truncating variants in (A) *ATM*, (B) *CHEK2*, (C) *PALB2* and (D) *XRCC2*.

Of the four genes, truncating variants in *PALB2* were associated with the highest BC risk, with an estimated OR=4.69 (95% CI 2.27 to 9.68, p=6.9×10^−6^)(table 1 and figure 2). Most truncating variants were rare, with 19/35 (54%) observed as singletons. Three variants, all in the final 280 codons of the gene, were more common: *PALB2* c.2718G>A (p.Trp906Ter, rs180177122; seven cases, one control), c.3113G>A (p.Trp1038Ter, rs180177132; 20 cases, one control) and c.3116delA (p.Asn1039IlefsTer2, rs180177133; eight cases, one control) (see online supplementary table 5). The combined OR for these three more common, C-terminal truncations was 3.57 (95% CI 1.27 to 10.1, p=0.016), somewhat, but not significantly, lower than the point estimate for all other truncating variants in the gene (OR=5.79; 95% CI 2.10 to 16.0, p=2.2×10^−4^; P_diff_=0.78).

10.1136/jmedgenet-2017-104588.supp5 Supplementary data

**Table 1:** Overall BC risk estimates for truncating mutations in each gene. Variant category tallies (online supplementary table S4) and carrier counts (online supplementary table S5) are available in the supplementary materials

	**Cases**			**Controls**			**OR (95% CI)**	**P-Value**
	**Carriers**	**Non-Carriers**	**Carriers Frequency**	**Carriers**	**Non-Carriers**	**Carrier Frequency**		
***ATM***	85	13,002	0.00649	11	5,477	0.00200	**3.26 (1.82–6.46)**	**2.1×10^-5^**
***CHEK2***	213	12,874	0.01628	29	5,459	0.00528	**3.11 (2.15**–**4.69)**	**5.6**×**10^-11^**
***PALB2***	89	12,998	0.00685	8	5,480	0.00146	**4.69 (2.27**–**9.68)**	**6.9**×**10^-6^**
***XRCC2***	9	13,078	0.00069	4	5,484	0.00073	0.94 (0.26–4.19)	0.92

10.1136/jmedgenet-2017-104588.supp4 Supplementary data

The risk estimate for *CHEK2*-truncating variants was OR=3.11 (95% CI 2.15 to 4.69, p=5.6×10^−11^; table 1 and figure 2). The most common truncating variant, *CHEK2* c.1100delC (p.Thr367MetfsTer15, rs555607708) accounted for 81% (196/242) of truncating variant carriers (online supplementary table S5) and was associated with an OR=3.18 (95% CI 2.01 to 4.92, p=6.1×10^−8^). The risk estimate for the aggregate of the remaining 14 rare truncating variants was OR=2.83 (95% CI 1.20 to 6.69, p=0.020), consistent with the estimate for *CHEK2* c.1100delC.

Protein-truncating variants in *ATM* were associated with an elevated risk of BC similar to *CHEK2* (OR=3.26; 95% CI 1.82 to 6.46, p=2.1×10^−5^; table 1 and figure 2).

Only 13 carriers of truncating variants in *XRCC2* were found in our study (table 1 and figure 2). There was no evidence for association with BC risk (OR=0.94; 95% CI 0.26 to 4.19, p=0.92).

For each of the four genes, the truncating variant carrier frequencies in the ExAC non-Finnish European population were similar to those in the study control group. Consequently, similar OR estimates were obtained when this dataset was substituted for the study controls (figure 1D–E and see online supplementary table S6).

10.1136/jmedgenet-2017-104588.supp6 Supplementary data

### BC associations for protein truncating variants disease subtype, age and family history

Truncating variants in *CHEK2* were associated with a higher relative risk for ER-positive (OR=3.42; 95% CI 2.33 to 5.21; table 2), and lower, non-significant risk for ER-negative BC (OR=1.59; 95% CI 0.80 to 3.00; P_diff_=0.0032). A similar pattern was observed for progesterone receptor status, though the difference was not significant (P_diff_=0.18). Truncating variants in *ATM* were also associated with a higher risk for ER-positive disease (OR=3.42; 95% CI 2.33 to 5.21) than ER-negative disease (OR=1.59; 95% CI 0.80 to 3.00), though not significantly so (P_diff_=0.11). There was no evidence of a difference in the estimated OR by ER-status for *PALB2* variants (ER-positive OR=4.32; 95% CI 2.07 to 10.5 vs ER-negative OR=5.58; 95% CI 2.19 to 15.2; P_diff_=0.55).

**Table 2:** Associations by family history, synchronous or metachronous bilateral disease, age at diagnosis and hormone receptor subtype.

		*ATM*			*CHEK2*			*PALB2*		
	All Cases	Carriers	OR (95%CI)	P-Value	Carriers	OR (95%CI)	P-Value	Carriers	OR (95%CI)	P-Value
Family History*										
**Positive**	1301 (17.0%)	**16 (29.6%)**			28 (23.5%)			7 (13.2%)		
**Negative**	6330 (83.0%)	**38 (70.4%)**	**2.06 (1.12–3.65)**	**0.022**	91 (76.5%)	1.51 (0.97–2.28)	0.070	46 (86.8%)	0.74 (0.60–1.54)	0.44
Bilateral Disease*										
**Positive**	220 (1.7%)	0 (0.0%)			11 (5.3%)			4 (4.7%)		
**Negative**	12 526 (98.3%)	83 (100.0%)	NA	0.089	198 (94.7%)	**3.27 (1.66–5.83)**	**0.0014**	81 (95.3%)	2.85 (0.86–6.91)	0.080
Age at Diagnosis*										
**Before 50**	3674 (28.4%)	25 (29.8%)	3.41 (1.72–7.23)		**76 (36.0%)**	**3.98 (2.62–6.21)**		27 (31.4%)	5.80 (2.67–14.5)	
**50s**	4952 (38.3%)	34 (40.5%)	3.44 (1.80–7.13)		**87 (41.2%)**	**3.37 (2.24–5.22)**		41 (47.7%)	6.54 (3.12–16.0)	
**After 60**	4305 (33.3%)	25 (29.8%)	2.91 (1.47–6.17)	0.66	**48 (22.7%)**	**2.12 (1.35–3.41)**	**1.2**×**10**^−^**^5^**	18 (20.9%)	3.29 (1.43–8.47)	0.22
**ER Status†**										
**Positive**	7845 (83.6%)	**50 (92.6%)**	**3.19 (1.73–6.47)**	**1.0×10^−4^**	**140 (91.5%)**	**3.42 (2.33–5.21)**	**1.5**×**10**^−^**^11^**	**43 (79.6%)**	**4.32 (2.07–10.5)**	**2.7**×**10**^−^**^5^**
**Negative**	1544 (16.4%)	4 (7.4%)	1.28 (0.36–3.76)	0.67	13 (8.5%)	1.59 (0.80–3.00)	0.18	**11 (20.4%)**	**5.58 (2.19–15.2)**	**3.6**×**10**^−^**^4^**
**PR Status†**										
**Positive**	3026 (66.5%)	**16 (64%)**	**2.65 (1.24–5.87)**	**0.012**	**61 (81.3%)**	**3.87 (2.51–6.12)**	**4.3E-10**	**16 (57.1%)**	**4.16 (1.77–10.8)**	**9.0**×**10**^−^**^4^**
**Negative**	1524 (33.5%)	**9 (36%)**	**2.96 (1.19–7.16)**	**0.021**	14 (18.7%)	1.75 (0.89–3.25)	0.100	**12 (42.9%)**	**6.21 (2.5–16.7)**	**1.0**×**10**^−^**^4^**

*Case only analysis.

†Case-control analysis.

Truncating *ATM* variants were more common in BC cases with a family history of BC among first-degree relatives (OR=2.06; 95% CI 1.12 to 3.64, p=0.022; table 2). There was some evidence of an association with a positive family history for carriers of truncating *CHEK2* variants (OR=1.51; 95% CI 0.97 to 2.28, p=0.070), but not for *PALB2* truncation carriers (OR=0.74; 95% CI 0.60 to 1.54, p=0.44). Bilateral BC was more common than unilateral disease in women with *CHEK2-*truncating variants (OR=3.27; 95% CI 1.66 to 5.83, p=0.0014; table 2). There was some evidence of an association with bilaterality for *PALB2* variant carriers (OR=2.85, 95% CI 0.86 to 6.91, p=0.080). No *ATM* truncations were found among bilateral BC cases.

The relative risk associated with *CHEK2-*truncating variants declined with increasing age, with estimated ORs of 3.98 for diagnosis before age 50, 3.37 between ages 50 and 59 and 2.12 after age 60 (P_trend_=1.2×10^−5^; table 2). For *ATM* and *PALB2* variant carriers, there was no clear evidence for an OR trend by age (P_trend_=0.66 and 0.22, respectively).

### Risk associations for subsets of missense variants

Thirty-eight missense variants had an ExAC carrier frequency >0.1% (25/299 in *ATM*, 4/77 in *CHEK2*, 7/125 in *PALB2* and 2/34 in *XRCC2*; see online supplementary table S4), and two were significantly associated with BC risk: *ATM* c.7390T>C (p.Cys2464Arg, rs55801750, OR(Arg/Cys)=0.37; 95% CI 0.19 to 0.73, P_trend_=0.0028) and *XRCC2* c.563G>A (p.Arg188His, rs3218536, OR(His/Arg)=0.90; 95% CI 0.83 to 0.97, P_trend_=0.0080) (see online supplementary table S7). However, neither of these associations was significant after adjusting for multiple testing. Of note, *CHEK2* c.470T>C (p.Ile157Thr, rs17879961) was found in 0.13% of subjects (20 cases, four controls; see online supplementary table S5) in this study. The relative risk estimate (OR(Thr/Ile)=2.10; 95% CI 0.72 to 6.14, p=0.17), although non-significant, is compatible with the 1.4-fold increased risk previously reported for the same variant in Finnish and Eastern European populations.^3 35^

10.1136/jmedgenet-2017-104588.supp7 Supplementary data

**Figure 3:**
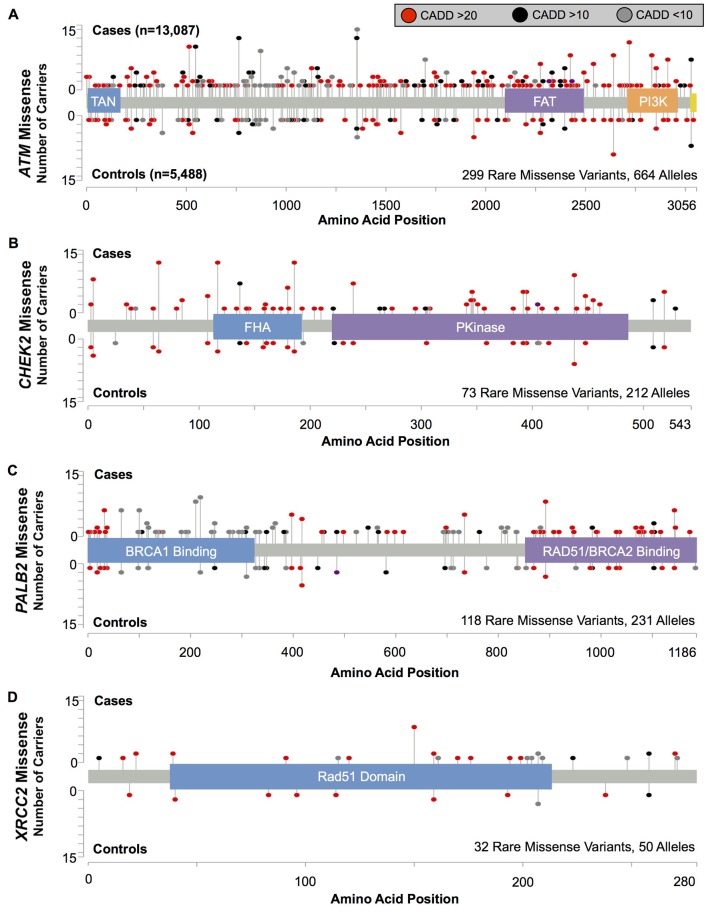
Position and frequency for rare missense variants in (A) *ATM* (B) *CHEK2* and (C) *PALB2*. ORs were calculated for all Pfam (ATM and CHEK2) and UniProt (PALB2) domains (table 2).

We tested for risks associated with the aggregate of all rare missense variants in each gene, irrespective of position or predicted deleteriousness (figure 3 and table 3). We found some evidence of increased BC risk associated with the combined rare missense substitutions in *ATM* (OR=1.18; 95% CI 0.99 to 1.40, p=0.073), *CHEK2* (OR=1.36; 95% CI 0.99 to 1.87, p=0.066) and *PALB2* (OR=1.28; 95% CI 0.95 to 1.73, p=0.12), but not in *XRCC2*. Considered together, rare missense variants in *ATM*, *CHEK2* and *PALB2* were associated with an estimated OR=1.24; 95% CI 1.08 to 1.43, p=0.0025 (table 3). Variants localised within protein domains (as defined by UniProt or Pfam) of these three genes had a somewhat higher risk estimate (OR=1.45; 95% CI 1.17 to 1.80; figure 3 and table 3) than those outside of the annotated domains (OR=1.09; 95% CI 0.92 to 1.31; P_diff_=0.060).

**Table 3:** Rare missense variant associations with BC by domain

	**Domain (AA Start-AA End)**	**Case carriers**	**Control carriers**	**OR (95% CI)**	**p Value**	**P_diff_**
***ATM***	All rare missense	489	175	1.18 (0.99 to 1.40)	0.073	
	In any domain	127	37	1.44 (1.00 to 2.08)	0.059	0.24
	Not in any domain	362	138	1.10 (0.90 to 1.35)	0.36	
	TAN (7-165)	17	10	0.71 (0.33 to 1.56)	0.52	
	FAT (2097–2488)	67	18	1.56 (0.93 to 2.63)	0.12	
	PI3K (2714–2961)	43	9	2.01 (0.98 to 4.12)	0.074	
	**FAT + PI3K domains**	**110**	**27**	**1.71 (1.12 to 2.61)**	**0.015**	
***CHEK2***	All rare missense	162	50	1.36 (0.99 to 1.87)	0.066	
	**In any domain**	**115**	**32**	**1.51 (1.02 to 2.24)**	**0.047**	0.45
	Not in any domain	47	18	1.10 (0.64 to 1.89)	0.85	
	FHA (113-191)	47	14	1.41 (0.78 to 2.56)	0.32	
	Pkinase (220-486)	68	18	1.59 (0.94 to 2.67)	0.10	
***PALB2***	All rare missense	174	57	1.28 (0.95 to 1.73)	0.12	
	In any domain	132	40	1.39 (0.97 to 1.98)	0.083	0.50
	Not in any domain	42	17	1.04 (0.59 to 1.82)	1.00	
	**BRCA1 binding (1-319)**	**71**	**17**	**1.76 (1.03 to 2.98)**	**0.047**	
	ChAM (395-466)	11	8	0.58 (0.23 to 1.43)	0.34	
	RAD51/BRCA2 binding (853–1186)	50	15	1.40 (0.79 to 2.49)	0.31	
	**Both binding domains**	**121**	**32**	**1.59 (1.08 to 2.35)**	**0.024**	
**Combined**	**All rare missense**	**825**	**282**	**1.24 (1.08 to 1.43)**	**0.0025**	
	**In any domain**	**374**	**109**	**1.45 (1.17 to 1.80)**	**7.3x10^-4^**	0.060
	Not in any domain	451	173	1.09 (0.92 to 1.31)	0.33	

Domain boundaries were determined from Pfam (*ATM* and *CHEK2*) or UniProt (*PALB2*).

BC, breast cancer.

There was no evidence that risk was higher among variants predicted to be deleterious by CADD, PolyPhen2, SIFT or AlignGVGD, for any of the four genes, and no subset of variants stratified by these annotations was significantly associated with risk (see online supplementary table S8). Similarly, the risk estimate for the aggregate of rare variants in *ATM*, *CHEK2* and *PALB2* with deleterious functional predictions was not significantly higher than for predicted benign variants (three genes combined P_diff_=0.91, 0.74, 0.71 and 0.76 for CADD, PolyPhen, SIFT and AlignGVGD, respectively; see online supplementary table S8).

10.1136/jmedgenet-2017-104588.supp8 Supplementary data

Previous analyses have indicated that *ATM* missense variants within the FRAP-ATM-TRRAP (FAT) and phosphatidylinositol 3-kinase (PI3K) domains were specifically associated with increased BC risk.^5 6^ We found evidence for increased risk for variants in both these domains (combined OR=1.71; 95% CI 1.12 to 2.61, p=0.015), but estimates did not differ significantly from those for the aggregate of all rare missense variants (P_diff_=0.31). Of note, c.7271T>G (p.Val2424Gly, rs28904921), which has been implicated in a milder Ataxia-Telangiectasia disease phenotype and has previously been associated with a substantial BC risk,^5 7 8^ occurred in eight cases and no controls in our study (figure 3 and see online supplementary table S5). After excluding this variant, the remaining rare missense substitutions in the FAT and PI3K domains in aggregate were still associated with BC risk (OR=1.59; 95% CI 1.04 to 2.43, p=0.040).

In *PALB2*, missense variants within the N-terminal BRCA1 binding domain were most strongly associated with risk (OR=1.76; 95% CI 1.03 to 2.98, p=0.047). This signal was driven by rare missense variants (n=29) between amino acids 70 and 300, and few of these were predicted by CADD, PolyPhen2, SIFT or AlignGVGD to have a deleterious effect on the protein (see online supplementary table S5).

### Non-canonical splice variants and BC risk

We also examined associations for common variants in non-coding regions (see online supplementary table S7). Among these, only *CHEK2* c.320–5T>A (rs121908700; OR=13.9; 95% CI 1.89 to 101, P_trend_=6.7×10^−4^) was significantly associated with risk after correction for multiple testing. This variant, in a non-canonical splice site, was predicted to reduce recognition of the normal splice acceptor site of exon 3 and introduce a new acceptor site three nucleotides upstream. At the protein level, this change would preserve the reading frame and cause the insertion of a valine residue. There was some suggestion of an association for the aggregate of other non-canonical splice variants in *CHEK2*, which were found in 12 cases and two controls (OR=2.52, 95% CI 0.56 to 11.3, p=0.26; online supplementary table S5).

## Discussion

This study, the largest experiment to date to systematically sequence the coding and exon-flanking regions of these genes in a population-based series of BC cases and controls, provides additional confirmation that protein-truncating mutations in *ATM*, *CHEK2* and *PALB2* are associated with increased BC risks. For *ATM* and *CHEK2*, the relative risks were higher for ER-positive than ER-negative disease, but we observed no differential effect by ER-status for *PALB2*. In contrast, *XRCC2*-truncating variants were not significantly associated with risk, but a twofold increased risk could not be excluded because these variants were very rare (13/18 575 samples; upper 95% confidence limit 4.19). These findings underscore the fact that, despite the large size of the study, the data are too sparse to accurately estimate risks for very rare variant classes and less common BC subtypes (eg, triple negative disease).

The BC risk estimates for all three of the associated genes were similar to estimates from smaller case–control studies and studies based on family-based designs (in the combined analysis of previous studies reported by Easton *et al* 4: *PALB2*: meta analysis OR=5.3; 95% CI 3.0 to 9.4 vs table 1 OR=4.69; 95% CI 2.27 to 9.68; *ATM*: meta analysis OR=2.8; 95% CI 2.2 to 3.7 vs table 1 OR=3.26; 95% CI 1.82 to 6.46; and *CHEK2:* meta analysis OR=3.0; 95% CI 2.6 to 3.5 vs table 1 OR=3.11; 95% CI 2.15 to 4.69). Based on the estimated population frequencies and relative risks from this study, truncating variants in *ATM*, *CHEK2* and *PALB2* would explain approximately 4% of the twofold familial relative risk of BC and approximately 2% of all BC cases. While these estimates were derived from a study in the UK, the comparability of the combined frequency of truncating variants in our study with those from ExAC suggests that these estimates are likely to be broadly applicable to other European populations. Somewhat surprisingly, we observed no association between carrying a truncating *PALB2* variant and a BC family history, but this may reflect lack of power: there were only 53 carriers for whom family history data were available.

The vast majority of the truncating variants in this study were very rare: 117/119 were found in <0.1% of samples. The most notable exception was *CHEK2* c.1100delC, which was identified in approximately 1.1% of subjects and accounted for 81% of truncation carriers in this gene. Our risk estimate for this variant (OR=3.18; 95% CI to 2.01 to 4.92) was somewhat higher than two recent analyses (BCAC: OR=2.26; 95% CI 1.90 to 2.69^10^; Danish cohort: OR=2.08; 95% CI 1.51 to 2.85).^36^ These differences might be explained by, for example, differences in the age distribution of the study subjects. The risk estimate for aggregated non-c.1100delC truncating variants in *CHEK2* was similar to that for c.1100delC, suggesting that results for this founder variant can reasonably be extrapolated to other truncating variants.

No individual missense variants showed evidence of association with BC risk at p<0.001, nor did we find strong evidence for the aggregate of rare missense variants in a single gene. There was, however, an association with BC risk for all rare, non-synonymous substitutions combined across *ATM*, *CHEK2* and *PALB2.* This risk could be mediated by a small subset of variants conferring a high risk, or a larger subset of variants associated with a lower risk. We observed little evidence of association by predicted effect severity, but there was, however, some suggestion that rare missense variants within functional domains may contribute to BC risk.

## Conclusions

This report, based on a large population-based study, provides relative risk estimates associated with truncating variants in *ATM*, *CHEK2* and *PALB2*. Our results confirm that risk estimates for *ATM* and *CHEK2* gene variants are similar and firmly within the twofold to fourfold range. *PALB2* protein-truncating variants conferred a somewhat higher risk, supporting previous suggestions that specific management may be justified in *PALB2* carriers.^11^ The absolute risks and age-specific penetrance in carriers will depend on additional influences, including common susceptibility variants, lifestyle risk factors and family history—considerations that can be built into more comprehensive risk prediction models.^37^ Clinically useful risk estimates for rarer disease subtypes and for missense variants will require studies that are substantially larger than the current experiment; these are becoming possible through large consortia and technological advances.

10.1136/jmedgenet-2017-104588.supp10 Supplementary data
