# Supplementary figures and images for: Rare, protein-truncating variants in ATM, CHEK2 and PALB2, but not XRCC2, are associated with increased breast cancer risks
Source: J Med Genet. 2017 Aug 4;54(11):732–41. doi: 10.1136/jmedgenet-2017-104588 (PMC5740532; doi:10.1136/jmedgenet-2017-104588)

Figure S1

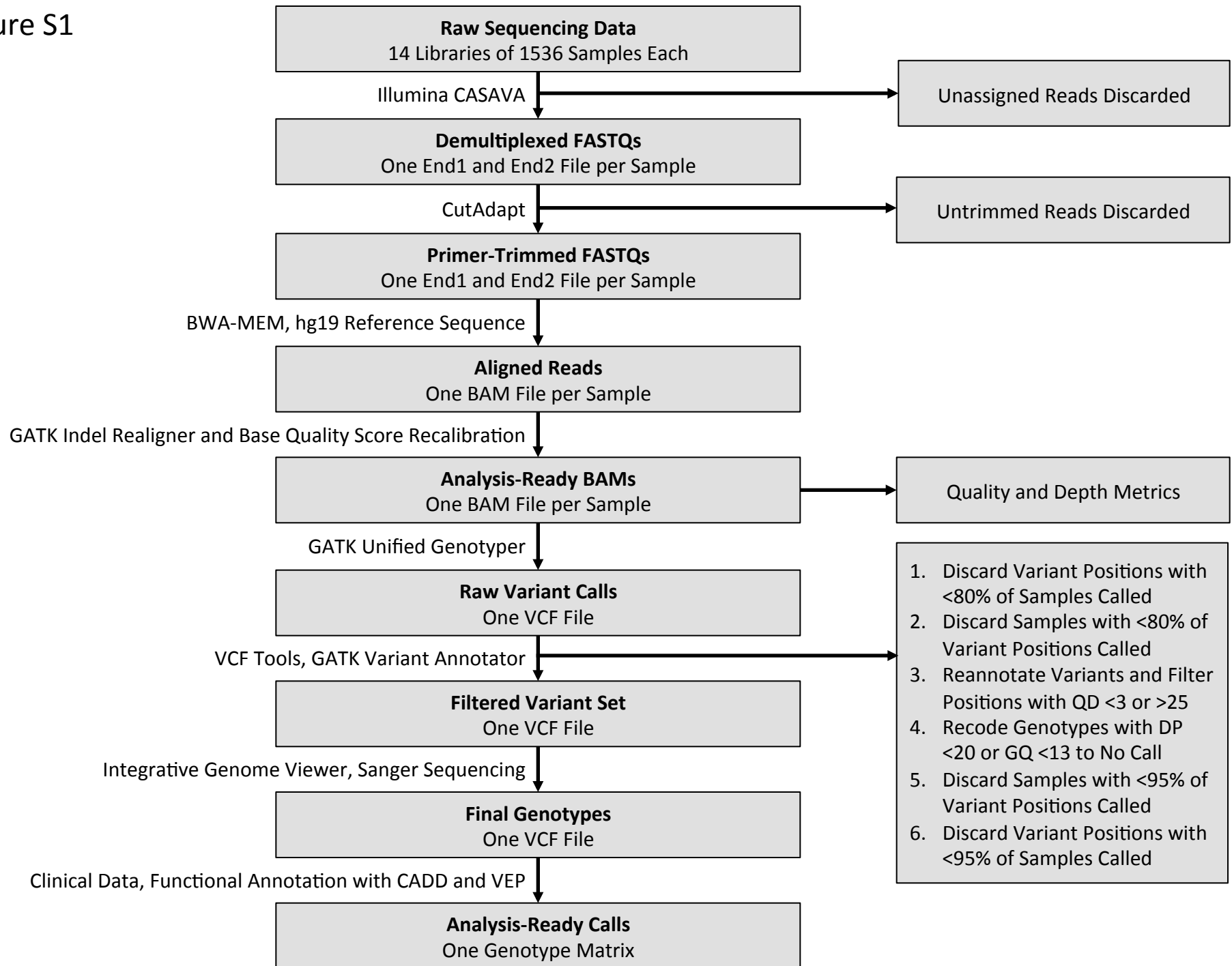

Supplement: Supplementary data [file jmedgenet-2017-104588supp009.pdf]
