# Supplementary material for: Rare, protein-truncating variants in ATM, CHEK2 and PALB2, but not XRCC2, are associated with increased breast cancer risks
Source: J Med Genet. 2017 Aug 4;54(11):732–41. doi: 10.1136/jmedgenet-2017-104588 (PMC5740532; doi:10.1136/jmedgenet-2017-104588)
Supplement: Supplementary data [file jmedgenet-2017-104588supp001.pdf]

Table S1. Primer sequnces and amplicon details. Partially overlapping amplicons were tiled across the target regions with a maximum amplicon length (forward and reverse primer length plus intervening unique sequence) of 200-bp. Primers were designed with melting temperature range 59.0°-61.0° C. Each primer included an orientation-specific tail sequence for subsequent ligation of adapter and barcode sequences.

Gene	Chr	Start	End	F-Primer	R-Primer	F-Primer Length	R-Primer Length	Unique Sequence Length	Total Amplicon Length	GC %
ATM	chr11	108098316	108098513	TTACAGACAGTGATGTGTGTTCT	TCAACTTCTTTCTGAAAATAAAAAGGAAATAA	23	32	143	198	32
ATM	chr11	108098462	108098632	GTGTGATTAGTAACCCATTATTATTTCCTT	AAATTTGAATAGAATACCTAAAAACAGCATCC	30	32	109	171	30
ATM	chr11	108098508	108098694	AGTTGAGAAATTTAAGCGCCTGAT	ACACACAAAAGTAATATCACAACAGAA	24	27	136	187	29
ATM	chr11	108099820	108100000	CTTTGTGATGGCATGAACAGCTT	ATCTTTTTCTGCCTGGAGGCTTG	23	23	135	181	31
ATM	chr11	108099927	108100103	AAAGAAACAGAATGTCTGAGAATAGC	CACGCGACAGTAATCTGTTAAGC	26	23	128	177	36
ATM	chr11	108106328	108106504	AGTTGCCATTCCAAGTGTCTTAT	TGAGTAGTATGTTGCTACAATCAGC	23	25	129	177	31
ATM	chr11	108106395	108106590	AGGAGCACCTAGGCTAAAATGTC	ATTCACAAACAACAACCTTCAAAAC	23	25	148	196	35
ATM	chr11	108114576	108114764	TCCTTTTTCTGTATGGGATTATGGA	ACAGCATGAATTATTCTAGCCACT	25	24	140	189	26
ATM	chr11	108114683	108114870	TGTTCTCTGTGTACTTCAGGCTC	ACAAAAGAAAAAGAGATTAGATTACCTCG	23	29	136	188	37
ATM	chr11	108114719	108114918	CACAAGATGTTCATAGAGTTTTAGTGG	CTGAGTCTAAAACATGGTCTTGC	27	23	150	200	35
ATM	chr11	108114741	108114919	AGTGGCTAGAATAATTCATGCTGT	ACTGAGTCTAAAACATGGTCTTGC	24	24	131	179	35
ATM	chr11	108115460	108115633	TTGTACAGTTTGTTCCCCCTGTT	TGGGAAGAATTTCATCTCCTAATTCACA	23	28	123	174	40
ATM	chr11	108115551	108115750	AGCAGCTCTTACTATCTTCCTCA	TTTCTTGGGTTTTGGCTCCTTTC	23	23	154	200	35
ATM	chr11	108115614	108115808	AGGAGATGAAATTCTTCCCACTTT	ACTTCTATGTTTGAATGAAGAAGCAA	24	26	145	195	31
ATM	chr11	108117623	108117815	TTTGTGGGAGCTAGCAGTGTAAA	TTTGACGGCAATATTACGAAATCC	23	24	146	193	31
ATM	chr11	108117736	108117932	ATGATCTGCTAGTGAATGAGATAAGT	TTTAAAAGCCCAAAATGCCCAGT	26	23	148	197	33
ATM	chr11	108117791	108117986	AGGATTTCGTAATATTGCCGTCAAAG	AGGAATAGTTGCATGTACAGAGTCA	26	25	145	196	34
ATM	chr11	108119559	108119753	GATACGAGATCGTGCTGTTCC	TCCTTTTGCAAGGGACACTGTAAT	21	24	150	195	36
ATM	chr11	108119688	108119880	AGATTTCTCAATCTTACACTACTACACA	AACATACAAGAGATTAAAATGACACTGAA	28	29	136	193	35
ATM	chr11	108121365	108121538	TTTCCTTTTAGTTTGTTAATGTGATGGA	CCATGTCGCTGTTGGGGTAG	28	20	126	174	34
ATM	chr11	108121468	108121666	AGTTTACCTAACTGTGAGCTGTCT	AGGTAATACACCAAATTTTATTCCAGAGT	24	29	146	199	40
ATM	chr11	108121552	108121722	TATGTGTTACGATGCCTTACGGA	GGCTCCAAGTAAGCCAAAGTTTTC	23	24	124	171	37
ATM	chr11	108121652	108121851	TTTGGTGTATTACCTTTCGTGGT	TGTGTCTGTGTGTGTTTATCTGTAAGT	23	27	150	200	40
ATM	chr11	108122503	108122698	AGATAAAGTCTTTGCCCCTCCAA	AAGAGCCATTTCATTATTGATTCCTTT	23	27	146	196	37
ATM	chr11	108122626	108122820	TGGGAATAGAGCAAAATATGTGTGAA	AGTGGAGAGAGCCTGATAAAACA	26	23	146	195	34
ATM	chr11	108123492	108123662	TCTTTACATGGCTTTTGGTCTTCT	AAGAGCATTATTAGATAACATACCATTCTG	24	30	117	171	33
ATM	chr11	108123547	108123725	TTTTCCTCATCTTGTACTGGAGAAA	GATGCAGCTACTACCCAGCTAAA	25	23	131	179	33
ATM	chr11	108124488	108124684	AGGCAAAGCATTAGGTACTTGGT	GAGAAGCCAATACTGGACTGGTG	23	23	151	197	35
ATM	chr11	108124529	108124727	ACTTTCTTGAAGTGAACACCACCA	CAGAAGACAGCGATCCAGTGATT	24	23	152	199	39
ATM	chr11	108124643	108124842	AGAATGTGGTATAGAAAAGCACCA	CCCCTATTTCTCCTTCCTAACAG	24	23	153	200	38
ATM	chr11	108126837	108127036	GAACTTTTGTTTTTTAATATGTATGTAGAATTTGTT	GCTTCCTCTTCAGCTATTACACCC	36	24	140	200	31
ATM	chr11	108126940	108127114	AGATTACAAATTCAGAAACTCTTGTCC	ATTTCATTCAAATTTATCCGAAACTTTATTAGT	27	33	115	175	37
ATM	chr11	108128126	108128315	CCAAGATCAAAGTACACTGTAAAAAGCA	GCAACGTGTACATAGCTGCATCA	28	23	139	190	33
ATM	chr11	108128198	108128373	TTCACAATAGTCTCTAATGCAATGTG	TCCTATCTCAAAAAACAAAACAAGAAGA	26	28	122	176	35
ATM	chr11	108129666	108129837	TGTTTTTATTTCTTTGTTGCTTGGTTCT	tcaaaatatgATAGCAAAACAGGAAGC	28	27	117	172	31
ATM	chr11	108137849	108138043	TGCTCCTGCAAGAAGCCAT	TCGTTTGCATCACTAACACTACTATCA	19	27	149	195	40
ATM	chr11	108137915	108138110	GCCATTTGACCGTGGAGAAGTA	AAAGAAGTAAAAGAAATCCCAAGTAGTAAA	22	30	144	196	37
ATM	chr11	108139084	108139260	TATATGGCTGTTGTGCCCTTCTC	GCTGCCCTAAAGGACACAGTATT	23	23	131	177	37
ATM	chr11	108139135	108139327	AGGTGCCATTAATCCTTTAGCTG	GTGGAGGGATTTGGTAGGTTCT	23	22	148	193	40
ATM	chr11	108139255	108139439	GGCAGCTGATATTCGGAGGAAAT	TCAATTCTTCAAAGACACCATGTGA	23	25	137	185	38
ATM	chr11	108141739	108141891	AAAGTAAATGATTTGTGGATAAACCTGA	AAGGTTTTAATTTCTTACGATAGTGGTT	28	28	97	153	36
ATM	chr11	108141813	108142007	TTCCTGGAGAAGAGTACCCCTTG	TGGTCACGACGATACAAAGAACA	23	23	149	195	33
ATM	chr11	108141856	108142052	ACTTCTGAAACCACTATCGTAAGAA	TTCACTACATGAAGGACATGGTT	25	23	149	197	29
ATM	chr11	108141993	108142192	GTATCGTCGTGACCAAGATGTTT	TGCATTCGTATCCACAGATAGCA	23	23	154	200	38
ATM	chr11	108143152	108143347	AGCACAGAAAGACATATTGGAAGT	TGCAAAAACTCACCTCAAGCAAA	24	23	149	196	33
ATM	chr11	108143256	108143454	CAGGCATCTAACAAAGGAGAGGA	ATCAGCCTACGGGAAAAGAACTG	23	23	153	199	32
ATM	chr11	108143380	108143553	AAGCAGTCTTTGTTTGTTAATGAGT	GCGAACTTGGTGATGATTGTCAG	25	23	126	174	34
ATM	chr11	108143427	108143626	AACCACAGTTCTTTTCCCGTAGG	TGCCATCTGCAGCATTCCAAATA	23	23	154	200	39
ATM	chr11	108150143	108150331	ATGCTTTGGAAAGTAGGGTTTGA	TCTCTCATTCCTTCCTGAGCTTT	23	23	143	189	37
ATM	chr11	108150215	108150412	AAGATTGTTCCAGGACACGAAGG	CCAAACTTGGTGAAGTAATTTATGGGA	23	27	148	198	37
ATM	chr11	108151619	108151817	TGTGTATAGCTTGTCAAAAAATCTGG	AGGGCTACAGGATAAAACCACAG	26	23	150	199	29
ATM	chr11	108151640	108151837	TCTGGAGTTCAGTTGGGATTTTAT	AAAGCCTGTTTTTCGCAGATAGG	24	23	151	198	30
ATM	chr11	108151724	108151896	CCATAGTGCTGAGAACCCTGAAA	CCTTTTTCACAAGGTGAGGTTCTA	23	24	126	173	39
ATM	chr11	108151805	108151985	ATCCTGTAGCCCTATCTGCGAAA	AAGTGCCACTCAGAAAATCTAGC	23	23	135	181	36
ATM	chr11	108153336	108153531	tgaATGTTGTTTCTAGGTCCTACTC	AGATTTAGCCATTCCAAAACCAGA	25	24	147	196	29
ATM	chr11	108153425	108153622	TGGTTCGTGCAGGTTTTAGAGAA	TCATGTATAAACTTACCTATAGAAATCCTCAAT	23	33	142	198	30
ATM	chr11	108153489	108153681	TGGCATCTCATTTAGATTATCTGGT	GGTGAGGGGACTTGCTAAGTATT	25	23	145	193	28
ATM	chr11	108154946	108155121	ATTTTTAGATCTTGTTATAAGGTTTTGATTCC	TCTCTGGTACCCTCATAGGCAAA	32	23	121	176	35
ATM	chr11	108154966	108155165	GGTTTTGATTCCACATCTGGTGA	CATAGACCTTGGTAGCAGTCTCTC	23	24	153	200	41
ATM	chr11	108155026	108155211	TCAGATTCAAGAGGACTGGAAAAGT	TGAAGCCATACCTGTTTTCCCAA	25	23	138	186	40
ATM	chr11	108155137	108155333	GCAAAGAGAGACTGCTACCAAGG	TGCTAGATAATGATTACCACAAGCTAAGT	23	29	145	197	30
ATM	chr11	108158251	108158445	AATATATGCCTTTTGAGCTGTCTTG	TACCCTGAAAAGTCACAGAGGTC	25	23	147	195	35
ATM	chr11	108158316	108158499	TGCCCTTGCAGATTGATCACTTA	ACTTCTTTAAAAACTTGTGAAGTTAGAGC	23	29	132	184	38
ATM	chr11	108159628	108159799	TCATTTTGGAAGTTCACTGGTCTA	TTTAACTTGGTTTTATGACAATTGCTG	24	27	121	172	34
ATM	chr11	108159697	108159875	CTGTTAGGGATTTGGATCCTGCT	AAAAAGAAGGAATGTTCTATTATTAAACTCATCA	23	34	122	179	33
ATM	chr11	108160276	108160447	TGTAGCCGAGTATCTAATTAAACAAGT	TCTTTCAGTAATAAACTAACAAACAGGTG	27	29	116	172	28
ATM	chr11	108160360	108160539	GTGAGCAAGCAGCTGAAACAAAT	ATGTTATTTACCTTTGGTTGATATAGTGAAT	23	31	126	180	32
ATM	chr11	108160418	108160588	TCACCTGTTTGTTAGTTTATTACTGAAAG	aaAAACAGGAAGAACAGGATAGAAAGA	29	27	115	171	33
ATM	chr11	108163250	108163437	AGAGATGCTGAACAAAAGGACTTC	TACAGTAAGTCACGGCTGTCTGG	24	23	141	188	37
ATM	chr11	108163372	108163553	TACGTAGCTTCTCCCTTTGTTGT	AAATACCATTTTGAAGATGAGTCAGAAA	23	28	131	182	40
ATM	chr11	108163979	108164165	TGTTGGCTTACTTTAAAATTATTTCTCTCC	TTGCTGAGTAATACGCAAATCCT	30	23	134	187	28
ATM	chr11	108164114	108164284	AGATCCTTTTCCTGACCATGTTGTT	ACTGCTAGAGCATTACAGATTTTTGA	25	26	120	171	32
ATM	chr11	108165598	108165782	TTATTTCACAGGCTTAACCAATACG	AGAAGCTCTCATAATGTCCACCA	25	23	137	185	35
ATM	chr11	108165662	108165833	CCATTTTCTCTCAGTAAGTGTTTATGATGC	TCAAAAAGAAACAGGTAGAAATAGCC	30	26	116	172	38
ATM	chr11	108167958	108168157	AACAAAAGTGTTGTCTTCATGCT	TGATCCGCAGTTGACTGAAA	23	20	157	200	36
ATM	chr11	108170338	108170537	TGAAGTACAGAAAAACAGCATTATAGTT	TTAAGGGCCTTGGTATAAGATGC	28	23	149	200	34
ATM	chr11	108170456	108170645	GCTTGGGAGAAGTGGGTCCTAT	GAGATTACATATAAGGTATCAATAAATACTCACCA	22	35	133	190	37
ATM	chr11	108170556	108170731	ACTTCAGTGGACCTTCATAATGCT	AGACACTGAGATTTAAAAAAGGACAAA	24	27	125	176	34
ATM	chr11	108172289	108172487	AGTTTTGAAATTTTTTCAGTGGAGGT	GATAGGCCAGCATTGGATCTGTT	26	23	150	199	35
ATM	chr11	108172386	108172571	GATCAGCAGCTGTTACCTGTTTG	ACAGAACTGTTTTAGATATGCTGGGT	23	26	137	186	35
ATM	chr11	108173515	108173710	TGTGTAGGAAAGGTACAATGATTTCCA	CCACTGTCCAAAAAAGCACAAGT	27	23	146	196	35
ATM	chr11	108173609	108173802	AAACCCTTTTGAAGGCCTGGATG	ACAGGTCATAAACAAGGAATTATATCAGAC	23	30	141	194	34
ATM	chr11	108175309	108175508	ATATGTCAACGGGGCATGAAAAT	CCCTGAACATGTGTAGAAAGCAG	23	23	154	200	31
ATM	chr11	108175408	108175579	ACTGACTTTTGTCAGACTGTACTTC	CTGAATCCAAGTTTGCAGGGGTT	25	23	124	172	41
ATM	chr11	108175504	108175674	CAGGGATTTTTCACCAGCTGTCT	AATCCTGTAAAGTGCTTTTAGTGGGA	23	26	122	171	39
ATM	chr11	108178569	108178745	GAAGGTGTGTAAGCAAGAATGCC	TCCTAAACGTAAGAAGCAACACTCA	23	25	129	177	40
ATM	chr11	108180792	108180991	AAGCAGTCACTACCATTGTATTCTA	AGTAAAGCTGTAAAGTGAGCAGCA	25	24	151	200	32
ATM	chr11	108180910	108181102	ATGATGCTTTCTGGCTGGATTTA	ACCCTTATTGAGACAATGCCAAC	23	23	147	193	34
ATM	chr11	108183082	108183269	TCAGGAGCTTCCAAATAGTATGTTCT	ATGAGATAAATACTGTCATAAATAATAGAGCC	26	32	130	188	32
ATM	chr11	108186449	108186630	AGTTGGGAGTTACATATTGGTAATGA	GGGTTGTAACATCTTCCCTCCAC	26	23	133	182	35
ATM	chr11	108186584	108186775	AGCCAGATAGTTTGTATGGCTGT	CTTTGCCCCACATTGCTTCG	23	20	149	192	37
ATM	chr11	108186693	108186863	TGTGTGTGTAAAACCCAAAGCTA	ACCAAATCTGGGAAAAAATGTACCTG	23	26	122	171	42
ATM	chr11	108186747	108186927	ATATGAACACGAAGCAATGTGGG	TCTGGCTGTGTAAATATCCACCAA	23	24	134	181	44
ATM	chr11	108188040	108188235	TCTGGTTTTCTGTTGATATCTTTGATTACT	AATGGTCCCACTGCATATTCCTC	30	23	143	196	36
ATM	chr11	108188115	108188299	GGACTCTGCCATATTCTTTCCGT	TTTAGAATGAGGAGAGAGGCAAAA	23	24	138	185	38
ATM	chr11	108190595	108190794	ATTTTGTCCTTTGGTGAAGCTATTT	TCATAATACCTGGCATATTTGAGACTT	25	27	148	200	32
ATM	chr11	108190680	108190879	GCAAAGAAGTAGAAGGAACCAGT	GCAAATCCTTACATTAAAATTGTTTCTCA	23	29	148	200	32
ATM	chr11	108191966	108192144	ACATTTATTTCCCTGAAAACCTCTTCT	GAGAAAAGCTCCCCAATGCTTTC	27	23	129	179	42
ATM	chr11	108192034	108192204	AGAAGTGGAAGAGATGTGTAAGCG	ACTATTGGTAACAGAAAAGCTGCAC	24	25	122	171	44
ATM	chr11	108195995	108196166	TCATTTCTCTTGCTTACATGAACTCT	CAGGATCTCCAAAATGACTGTGC	26	23	123	172	42
ATM	chr11	108196070	108196241	TATTAAGTGGCAGAAACACTCCC	AGAGAGTTCTACAAGGTGTTTGGT	23	24	125	172	41

ATM	chr11	108196144	108196316	GCACAGTCATTTTGGAGATCCTG	AGGAAAGTCAAGAGGTAAGATGAC	23	24	126	173	38
ATM	chr11	108196740	108196939	ATCAGTAGTAAAAGTATTTATTCCCATATGTC	GCATCCAACTTCTTGATCATTTGCT	32	25	143	200	40
ATM	chr11	108196815	108196990	CAGTACAATTCAGTTAGCTGTGGAG	ACAGTAAAACACTAATCCAGCCAA	25	24	127	176	44
ATM	chr11	108198316	108198507	GTTGGGTACAGTCATGGTAATGC	AGGGTTGCTCCAAAAATCTTACCT	23	24	145	192	40
ATM	chr11	108198368	108198557	ACAGAACAATCCCAGCCTAAAACT	ATTGATCTTGATGAAAAGATGAAGCA	24	26	140	190	39
ATM	chr11	108199673	108199848	ACCTTAATTTGAGTGATTCTTTAGATGTATT	TGAGTATCTGAAAACCGGGCTAA	31	23	122	176	32
ATM	chr11	108199730	108199910	GGTTGTGTTTTCTTGAAGGCAGTAG	TCAGGAGAGCTTGCTTGTTTTCA	25	23	133	181	37
ATM	chr11	108199828	108200004	AGCCCGGTTTTCAGATACTCAAT	TGGTGAACATAAAATTGTCACTTGTAGAAA	23	30	124	177	37
ATM	chr11	108200875	108201072	TGTGTGATTTTGTAGTTCTGTTAAAGTTC	TGTTCTTCTCCACTTAATAAGCAGTT	29	26	143	198	36
ATM	chr11	108200990	108201182	CGTGCACTGAAAGAGGATCGTAA	CACAGGGTAGAATATTGGGCTGA	23	23	147	193	40
ATM	chr11	108202126	108202312	TGAAATACCTTGTTTCTTAATTTTGTGTC	ttGTGGTTTGATTTTCAGGTTTACT	29	25	133	187	35
ATM	chr11	108202479	108202677	CAGGCATACACGCTCTACCC	GTTTGCATTTGCTAAGGCCAGT	20	22	157	199	32
ATM	chr11	108202628	108202810	ATCACCCCCATCACACTTTGTTT	ACTGAATATCACACTTCTAAAAGGTACG	23	28	132	183	37
ATM	chr11	108203392	108203580	TGTGCATAAATTCTGTTTTTCTCTTTGTTT	TAAGCATCACAAAGTGCCTCAAC	30	23	136	189	33
ATM	chr11	108203491	108203679	TCGAACAGAGGCTGCAAATAGAA	ACAGAGAGTAACACAGCAAGAAAGT	23	25	141	189	37
ATM	chr11	108204495	108204691	GAGCCTGAACCACAGATTAGCAA	ATTTCCATAGTAGGGACAACAACA	23	24	150	197	39
ATM	chr11	108204623	108204802	TATTCCAGCAGACCAGCCAATTA	TGCCAATATTTAACCAATTTTGACCT	23	26	131	180	26
ATM	chr11	108205618	108205812	TGCCATTTATAATGTATTTTTCTTTAAGTGC	GCCATCGGAACCTACACAATCTA	31	23	141	195	33
ATM	chr11	108205689	108205874	TTATTAGGTGGACCACACAGGAG	AAAACTCTAAGGGCTAAGCCAGA	23	23	140	186	41
ATM	chr11	108206475	108206666	CAGATTGGTTTGAGTGCCCTTTG	TTCCTCTTCCTAGTTTCCGTGTT	23	23	146	192	39
ATM	chr11	108206559	108206750	AATTATTCTGAAGGGCCGTGATG	cacccaaccAAATGGCATCTTTTA	23	24	145	192	38
ATM	chr11	108213864	108214058	TTGCACTGACTCTGATAGCTGAA	GGCCTGTATCTTTTATGAGCACCA	23	24	148	195	41
ATM	chr11	108213975	108214162	TGTTCTTGAATGGTGCACAGGA	GCTGTCAGCTTTAATAAGCCATT	22	23	143	188	37
ATM	chr11	108216364	108216557	TCATCTTTATTGCCCCTATATCTGTC	TGCAGAAGTAACGGAAAACTGGT	26	23	145	194	26
ATM	chr11	108216503	108216680	GAAGTCTTCATGGATGTTTGCC	CCAAACAACAAAGTGCTCAATCT	22	23	133	178	38
ATM	chr11	108217970	108218167	CAGTGATTTCAGATTGTTTGTTTCTTTTT	CTTGGTAGGCAAACAACATTCCA	29	23	146	198	32
ATM	chr11	108224395	108224580	AACATGTGGTTTCTTGCCTTTGT	ATGCCCATGCCATCCACAATATC	23	23	140	186	39
ATM	chr11	108224489	108224668	TTAGGTGTTGCTTTTGAACAGGG	gcccagcCCATGTAATTTTGAC	23	22	135	180	42
ATM	chr11	108225438	108225608	GCATAGGCTCAGCATACTACACA	ACTTTACCTCTACAATGGTTAACAGA	23	26	122	171	36
ATM	chr11	108225539	108225720	TGCTGTGAGAAAACCATGGAAGT	CCAGCCACATCCCCCTATGTTA	23	22	137	182	39
ATM	chr11	108235716	108235915	ACTGGCTTATTTGTATGATACTGGTTC	GCATTCAGAGTAGGGTGAAGCTC	27	23	150	200	37
ATM	chr11	108235828	108236007	TCTTTGACTGGACCATGAATCCTT	AGGCCTTGGGAATAAGAAAATCTG	24	24	132	180	42
ATM	chr11	108235968	108236167	CCTGTTGTCAGTTTTTCAGATTTTCTT	GCAAATTCACTTGTCCACCAACA	27	23	150	200	41
ATM	chr11	108236070	108236269	CAACAAAGTAGCTGAACGTGTCTT	AGGCTGAATGAAAGGGTAATTCATA	24	25	151	200	44
CHEK2	chr22	29083764	29083961	TGACTGTGAAAAAGCAATTATTCCCATA	GAAAGCGGCCCCGTGAAG	28	18	152	198	43
CHEK2	chr22	29083884	29084080	TTCACAACACAGCAGCACACAC	ACATCAAATGCCCCCACTTTACT	22	23	152	197	46
CHEK2	chr22	29085056	29085244	TCATCATCTTTGCTTATCAGCTCCT	TCCTTCAGACACAGCTACTTATG	25	23	141	189	41
CHEK2	chr22	29089944	29090135	TGTCCTCTGTCTCATGTCTCTCA	TCACTGTGATTTGCCCAATTGTT	23	23	146	192	49
CHEK2	chr22	29091010	29091198	ACAGAATTGACAGGAGAAAACACCA	GGACTCAAGTGTCACTGAAGGAT	25	23	141	189	41
CHEK2	chr22	29091109	29091283	TCATACCTTTCTCTGAGACTTCTGC	CCACTGAGAATGCCACTTGATTT	25	23	127	175	42
CHEK2	chr22	29091641	29091818	TGTGACTTCATCTAATCACCTCCT	TCATGAGAACCTTATGTGGAACC	24	23	131	178	44
CHEK2	chr22	29091714	29091901	CTCCTAAACTCCAGCAGTCCAC	TGGCAAGTTCAACATTATTCCCT	22	23	143	188	46
CHEK2	chr22	29092834	29093019	AGTTTCTGAACAAGAATCTACAGGA	ATCTACTGCATGAATCTGAGGGT	25	23	138	186	39
CHEK2	chr22	29095758	29095954	TGCCTAATTCAGGGAGTAATTCAAC	GAACCCCTTGCCTTGCCTTT	25	20	152	197	46
CHEK2	chr22	29099400	29099584	CAGGATGAGAAAGGCAAGCCTAC	ACTTTCCCTTTTTTCTCCCCCTC	23	23	139	185	32
CHEK2	chr22	29105921	29106070	ACTGAAAGGCTTTATACTCTTCTCATATT	CTCTGTTATTCTGTTTATCAAAGGACCC	29	28	93	150	29
CHEK2	chr22	29105986	29106135	ATACTTACATGATTTAGCTTTTTCAAAATTTCTATT	CTTGAGTCAACTGAGTTTAACTGTAAATGTTTTT	36	34	80	150	27
CHEK2	chr22	29107772	29107951	ACTCACAAATTCATCCATCTAAGCA	AGCCATAAAGATCATCAGCAAAAGG	25	25	130	180	36
CHEK2	chr22	29107872	29108071	TGATCAGCCTTTTATTGGTACTTACTG	CCCAGGAGTGGTAGGTCTCATA	27	22	151	200	41
CHEK2	chr22	29115310	29115504	CAGAAATGAGAAACCACCAATCACA	TGAAACCCATTTCTACTCTTTTCTTC	25	26	144	195	30
CHEK2	chr22	29120878	29121073	ACCATATTCTGTAAGGACAGGACAAA	GATCACAGTGGCAATGGAACCTT	26	23	147	196	34
CHEK2	chr22	29120969	29121145	ATTTCTGCTTAGTGACAGTGCAA	TCAAAGTCTGAAACAAAATGTTCTCTAT	23	28	126	177	35
CHEK2	chr22	29121130	29121325	TTGTTTCAGACTTTGAATAGCAGAG	GGGACAAAAGCTGTGAATATTGCT	25	24	147	196	35
CHEK2	chr22	29121185	29121384	GACCAAATTACCAGCTCTCCT	GCCCTCTGATGCATGCTTTTAT	21	22	157	200	39
CHEK2	chr22	29130301	29130491	AACTCCAATCAGAACCTTCCACC	TATTCCTGAGGACCAAGAACCTG	23	23	145	191	46
CHEK2	chr22	29130405	29130587	CCATCCTGAAGGGCCCATAATC	CTCTACCAGCACGATGCCAAA	22	21	140	183	56
CHEK2	chr22	29130478	29130650	ggtcctcaggAATAGAATAGAGTTC	GCCCCATGGCAGCGTTA	25	17	131	173	57
CHEK2	chr22	29130506	29130690	AGTGGACACTGTCTCTAAGGAGC	TTGAGGCTCAGCAGTCTCATGG	23	22	140	185	57
CHEK2	chr22	29130550	29130749	GAGAGGACTGGCTGGAGTTTG	ACTCACCTTTGTTGTTGGACACT	21	23	156	200	55
PALB2	chr16	23614718	23614903	ACTCCAAAAAAATACTAAGAGGCCCAA	GTGTACTGCCCTCCTCCCA	26	19	141	186	39
PALB2	chr16	23614813	23614984	TTTTTGTCCAGCCAGCAAATGAG	GGAAGGTGACGTGAAAGATCACT	23	23	126	172	47
PALB2	chr16	23614918	23615095	GTCCCAAATGGCAATTGTTCCAG	TTTGTTCCTGTTGCTGGTTTTGG	23	23	132	178	37
PALB2	chr16	23619121	23619297	TTTTGTGTTTGCACAGTGCCTTT	AAAGAGAGTGAGTCGTTGCGAAG	23	23	131	177	49
PALB2	chr16	23619192	23619382	CCTGCCCTGGAGGAAGACA	ACATACTCTTGACAGTCTATTTGGGA	19	26	146	191	45
PALB2	chr16	23625270	23625462	CACTTAATGAGACCAACAGTAACAC	ACTGGTTTGTTGGAAGAATGTGA	25	23	145	193	37
PALB2	chr16	23632598	23632797	TTAATCTTCACAACAACCCTGTAAAAT	GCAAAGAAAACCAATTTTTGATGCC	27	25	148	200	39
PALB2	chr16	23632656	23632854	AACTGAGGACCTAGAGGGAAAGC	ACCTAGAGACTGCTTTAGTGCAA	23	23	153	199	38
PALB2	chr16	23634201	23634397	ACGAGATCCTAGTTACCCAACTTT	TGGAAATATAAAAGCTGTGCTTGGC	24	25	148	197	38
PALB2	chr16	23634333	23634503	AAAGGGTCCCACTGCTACTAACT	TATTAAAAGGTTACTCCTCACATCACCC	23	28	120	171	40
PALB2	chr16	23635275	23635447	AGCTGACAGAGACAAAGATGAAGG	AGCATAATTTTTGGCTGCTTTGTTT	24	25	124	173	36
PALB2	chr16	23637478	23637667	CCAATTTTGGTAAGCTGCCCATC	GCCGGTTGTAAAGAGCCATGTAT	23	23	144	190	44
PALB2	chr16	23637543	23637741	AGATTCCCACTTACCTCTGCGAA	CAGAACTGTTGCCATTGTGTCAG	23	23	153	199	44
PALB2	chr16	23637669	23637839	CTTTCCCAAAACATGGCACTCAC	AAGACTTAAATTTTACATACCTACTGTTTCA	23	31	117	171	37
PALB2	chr16	23640434	23640632	AGAACAAGAAGCTATATGACTGAATTCTT	AAGTGTAGACTAATGATGTGACTTTTG	29	27	143	199	36
PALB2	chr16	23640891	23641085	TCATGCTGTTTACATTCACTAAGGC	AACCTACCTGTGACTGTGACTCT	25	23	147	195	43
PALB2	chr16	23640986	23641180	TCCTGGCATGTGTTTCTACAGAG	CTCAGTCTGTCTTGCCAGTGATA	23	23	149	195	48
PALB2	chr16	23641064	23641260	GAGTCACAGTCACAGGTAGGTTG	CCTCAAGGCTCCTATGAAAAAGC	23	23	151	197	48
PALB2	chr16	23641189	23641388	ATGAGCAAGTTGGGGTGTGC	CGGGCCTTTCTTCATCCATATTACT	20	25	155	200	46
PALB2	chr16	23641246	23641444	ATAGGAGCCTTGAGGGCCAAA	TCTACCAGGAAAATCACATCCCA	21	23	155	199	46
PALB2	chr16	23641297	23641488	AGGTGAACACATGTCTGTGGTAG	CGCATGGATACAGAAATGGAGGA	23	23	146	192	43
PALB2	chr16	23641355	23641541	GGAGTATAAAGTAATATGGATGAAGAAAGGC	AGAGACATCTTAAAGAGGGAAGCTG	31	25	131	187	42
PALB2	chr16	23641463	23641633	AAGTCCTCCATTTCTGTATCCATGC	ACCTGATGAAGACTTTGGACCTC	25	23	123	171	42
PALB2	chr16	23641559	23641731	TCAAAGGGCTCCACTGGTTTTTC	TCCTTGGATGATGATGCTTTCAC	23	23	127	173	42
PALB2	chr16	23641644	23641843	CTGTGATACTGAGAAAAGACAGTAGT	TGTCTGTTTTGTTGGGTTTTGTT	26	23	151	200	37
PALB2	chr16	23646073	23646272	acacttggccCTGTCACTTTTT	CAACTTCTAGCCTGTCGATTGTT	22	23	155	200	35
PALB2	chr16	23646250	23646438	AACAATCGACAGGCTAGAAGTTG	ACCTCTCAGAAACTTCTCTCATTAAC	23	26	140	189	46
PALB2	chr16	23646305	23646480	GTGCAGGCTGATTTTCTTTTTCC	ATTAGGATGTCTGGCACATGCAC	23	23	130	176	46
PALB2	chr16	23646420	23646592	AGAGAAGTTTCTGAGAGGTTCTTGA	GGAGGCTGTCATTCAGAGTCATT	25	23	125	173	37
PALB2	chr16	23646508	23646698	AAGGTTTAAATTTTTACTTGCATCCTTATTT	CTGCAGAAAAACATTCTTGCACA	31	23	137	191	37
PALB2	chr16	23646599	23646791	ACTTTCCTCTGGCAATTGGACAT	GTGACACTCTTGATGGCAGGAAT	23	23	147	193	42
PALB2	chr16	23646733	23646929	AGGTTGACTTAGAATCTCACTTTCCTG	GCCAACTGCCCACAAGTTCTAAT	27	23	147	197	34
PALB2	chr16	23646919	23647101	GGGCAGTTGGCCACTTTTACTTA	AGCGGTAGTAGTCAGCACCTT	23	21	139	183	40
PALB2	chr16	23646992	23647191	TGTGCCTCCAAACTTACAGGT	ACTGCCCAACCAGAAAAAGGT	21	21	158	200	40
PALB2	chr16	23647117	23647307	TAAAGGAACTGTAGTCGCCCTG	CTGCTAGATCACCAGTAACTGAAA	22	24	145	191	37
PALB2	chr16	23647173	23647351	CTTTTTCTGGTTGGGCAGTTGG	TGGGAAAAGACTAAAGGAACAGGA	22	24	133	179	36
PALB2	chr16	23647303	23647497	AGCAGGATTTTTGCTACTGATTTCT	ACCCAAGAACATTTTCCCCACAG	25	23	147	195	44
PALB2	chr16	23647438	23647630	CAGCTTCTGCTTTTGCTCACC	TGACAAGTTACACATCAAAACCCA	21	24	148	193	46
PALB2	chr16	23647607	23647786	TGGGTTTTGATGTGTAACTTGTCAT	TGAATGAAATGTCACTGATTCTTTCTTAAAT	25	31	124	180	21
PALB2	chr16	23649110	23649300	AGCCAAAATATACCTGGGAAATGA	TTCTGGGGCTGTTTTTGTCTCC	24	22	145	191	39
PALB2	chr16	23649324	23649504	AGAGTCAAGAACTGTTTTTAAATTGTTTG	CTCTGACTCCACCTTTCCACTTG	29	23	129	181	38
PALB2	chr16	23652326	23652511	AGATGATACTGCTGCCCTCG	GACGGCTGCTCTTTTCGTTCTG	20	22	144	186	63
XRCC2	chr7	152345680	152345871	TTTAAGGcTTGCGTAGTACCCTG	aCTGGTGAAGCACAGGATGTTTT	23	23	146	192	32
XRCC2	chr7	152345730	152345916	ACAAAATTCAACCCCACTTTCTCC	TGTGGACAtAGACTACAGACCTT	24	23	140	187	35
XRCC2	chr7	152345847	152346021	GAAAAACATCCTGTGCTTCACCA	TGTAAATGACTATCgCCTGGTTCT	23	24	128	175	45
XRCC2	chr7	152345919	152346111	ACACAGTCGTCGAGAGGCAT	TTTTTACTGGATAGACCGCGTCA	20	23	150	193	44
XRCC2	chr7	152346023	152346203	GCTTCTCTAAGCACTGAGAACAT	AGCACCCACTTACTTCTTACACT	23	23	135	181	43
XRCC2	chr7	152346086	152346283	CATTGACGCGGTCTATCCAGTAA	TTGAGCACAGACTATCCCAAAGC	23	23	152	198	40
XRCC2	chr7	152346180	152346364	AAGTGTAAGAAGTAAGTGGGTGCT	AATCAGAAGGTGGCCTGGAAGTA	24	23	138	185	39
XRCC2	chr7	152346259	152346438	GAGCTTTGGGATAGTCTGTGCTC	TGAATTTCATGGCCCAGAAGGAA	23	23	134	180	41

XRCC2	chr7	152346296	152346494	GCCGGAGCATATCAAAGTGGTAA	CCCTTGAGCTACTGCATTTTGAC	23	23	153	199	39
XRCC2	chr7	152357715	152357901	ACATGTGAGCTTATGTGaAAAATCCT	AATAATGAGTTTTCCTTCtCTCTTcTTTT	26	29	132	187	32
XRCC2	chr7	152373031	152373227	CCCCAAGCCTCCCAATCC	CAGTTGGTGAATGGCGTTGGT	18	21	158	197	66
								138.5258216		
