# Supplementary material for: Rare, protein-truncating variants in ATM, CHEK2 and PALB2, but not XRCC2, are associated with increased breast cancer risks
Source: J Med Genet. 2017 Aug 4;54(11):732–41. doi: 10.1136/jmedgenet-2017-104588 (PMC5740532; doi:10.1136/jmedgenet-2017-104588)
Supplement: Supplementary data [file jmedgenet-2017-104588supp002.pdf]

Table S2. Design, callable bases, and depth summary.

	Ensembl Transcript ID	Ensembl Protein ID	Exon Length	Amplicons	Percent Target Sequence Covered	Percent Callable Bases within Target			Sequence Depth Coverage per Gene		
						Median	Mean	SD	Median	Mean	SD
ATM	ENST00000278616.4	ENSP00000278616.4	9171	135	99.7%	99.2%	98.8%	0.6%	425.2	428.6	99.2
CHEK2	ENST00000328354.6	ENSP00000329178.6	1632	25	100.0%	97.1%	97.0%	0.7%	399.4	404.9	88.7
PALB2	ENST00000261584.4	ENSP00000261584.4	3561	50	97.7%	96.9%	96.9%	0.3%	454.8	460.2	101.9
XRCC2	ENST00000359321.1	ENSP00000352271.1	843	11	100.0%	99.9%	99.6%	1.5%	472.2	481.8	118.6
