# Supplementary material for: Rare, protein-truncating variants in ATM, CHEK2 and PALB2, but not XRCC2, are associated with increased breast cancer risks
Source: J Med Genet. 2017 Aug 4;54(11):732–41. doi: 10.1136/jmedgenet-2017-104588 (PMC5740532; doi:10.1136/jmedgenet-2017-104588)
Supplement: Supplementary data [file jmedgenet-2017-104588supp003.pdf]

Table S3. Concordance between duplicates and agreement between orthogonal genotyping methods.

	iCOGS SNPs	OncoArray Indels	TOTAL
**Samples**	13779	4320	
**Polymorphic variants**	37	80	
**No found on sequencing**	4	7	
**Rare variants (MAF<0.01)**			
Variants	26	70	
Correctly called by sequencing	716	543	1259
Incorrectly called by sequencing	5	11	16
Not called by sequencing	12	43	55
Variant not found by sequencing	13	15	28
Sensitivity (called genotypes only)	99.30%	98.00%	98.70%
Sensitivity (all known heterozygotes)	95.98%	88.70%	92.70%
**Common variants (MAF>=0.01)**			
Variants	11	10	21
Concordant	145744	42987	188731
Not concordant	71	2	73
Concordance	99.95%	99.995%	99.96%
