# Supplementary material for: Rare, protein-truncating variants in ATM, CHEK2 and PALB2, but not XRCC2, are associated with increased breast cancer risks
Source: J Med Genet. 2017 Aug 4;54(11):732–41. doi: 10.1136/jmedgenet-2017-104588 (PMC5740532; doi:10.1136/jmedgenet-2017-104588)
Supplement: Supplementary data [file jmedgenet-2017-104588supp005.pdf]

Table S5. All variants with annotations and frequencies.

Gene	Chromosome	Position	Reference Allele	Alternate Allele	Known Variation	Truncating	CADD Consequence Detail	HGVS CDS Notation	HGVS Protein Notation	Amino Acid Substitution	Protein Domain	CADD Scaled Score	PolyPhen2 Category	PolyPhen2 Score	SIFT Category	SIFT Score	AlignGVGD Score	ExAC Non-Finnish European Carrier Frequency
ATM	11	108098353	T	C	CM960095,COSM1561141,COSM1561140	NO	initiator_codon	ENST00000278616.4:c.2T>C	ENSP00000278616.4:p.Met1?	M1T	PTHR11139,PTHR11139:SF66	25	probably_damaging	0.997	deleterious	0	NA	0.00000
ATM	11	108098354	G	A	CM030187,rs781404312	NO	initiator_codon	ENST00000278616.4:c.3G>A	ENSP00000278616.4:p.Met1?	M1I	PTHR11139,PTHR11139:SF66	27.3	probably_damaging	0.995	deleterious	0	NA	0.00000
ATM	11	108098386	G	A	NA	NO	missense	ENST00000278616.4:c.35G>A	ENSP00000278616.4:p.Cys12Tyr	C12Y	Pfam_domain:PF11640,PTHR11139,PTHR11139:SF66	26.6	probably_damaging	1	deleterious	0	65	0.00000
ATM	11	108098389	G	A	rs778201041,COSM1183947,COSM1183946	NO	missense	ENST00000278616.4:c.38G>A	ENSP00000278616.4:p.Arg13His	R13H	Pfam_domain:PF11640,PTHR11139,PTHR11139:SF66	22.8	benign	0.176	tolerated	0.21	0	0.00000
ATM	11	108098390	T	C	NA	NO	synonymous	ENST00000278616.4:c.39T>C	NA	R13R	Pfam_domain:PF11640,PTHR11139,PTHR11139:SF66	4.066	NA	NA	NA	NA	NA	0.00000
ATM	11	108098393	AC	A	rs771887195	YES	frameshift,feature_truncation	ENST00000278616.4:c.43delC	ENSP00000278616.4:p.Leu15Ter	L15X	Pfam_domain:PF11640,PTHR11139,PTHR11139:SF66	25.6	NA	NA	NA	NA	NA	0.00000
ATM	11	108098407	G	A	NA	NO	missense	ENST00000278616.4:c.56G>A	ENSP00000278616.4:p.Arg19Lys	R19K	Pfam_domain:PF11640,PTHR11139,PTHR11139:SF66	9.874	benign	0.012	tolerated	1	0	0.00000
ATM	11	108098417	A	G	NA	NO	synonymous	ENST00000278616.4:c.66A>G	NA	E22E	Pfam_domain:PF11640,PTHR11139,PTHR11139:SF66	8.13	NA	NA	NA	NA	NA	0.00000
ATM	11	108098445	A	T	rs761358619	NO	intron	ENST00000278616.4:c.72+22A>T	NA	NA	NA	3.755	NA	NA	NA	NA	NA	0.00000
ATM	11	108098501	A	G	CS053444	YES	splice_acceptor	ENST00000278616.4:c.73-2A>G	NA	NA	NA	24.9	NA	NA	NA	NA	NA	0.00000
ATM	11	108098508	A	T	NA	NO	missense	ENST00000278616.4:c.78A>T	ENSP00000278616.4:p.Glu26Asp	E26D	Pfam_domain:PF11640,PTHR11139,PTHR11139:SF66	23.5	possibly_damaging	0.528	tolerated	0.12	0	0.00000
ATM	11	108098514	G	A	NA	NO	synonymous	ENST00000278616.4:c.84G>A	NA	E28E	Pfam_domain:PF11640,PTHR11139,PTHR11139:SF66	11.65	NA	NA	NA	NA	NA	0.00000
ATM	11	108098563	C	T	rs3218684	NO	missense	ENST00000278616.4:c.133C>T	ENSP00000278616.4:p.Arg45Trp	R45W	Pfam_domain:PF11640,PTHR11139,PTHR11139:SF66	28.9	possibly_damaging	0.893	deleterious	0	25	0.00006
ATM	11	108098568	T	C	CD982445,rs770834907	NO	synonymous	ENST00000278616.4:c.138T>C	NA	H46H	Pfam_domain:PF11640,PTHR11139,PTHR11139:SF66	0.142	NA	NA	NA	NA	NA	0.00003
ATM	11	108098576	C	G	CM990201,rs1800054	NO	missense	ENST00000278616.4:c.146C>G	ENSP00000278616.4:p.Ser49Cys	S49C	Pfam_domain:PF11640,PTHR11139,PTHR11139:SF66	25.1	possibly_damaging	0.841	deleterious	0	15	0.02247
ATM	11	108098592	T	C	rs3218690	NO	synonymous	ENST00000278616.4:c.162T>C	NA	Y54Y	Pfam_domain:PF11640,PTHR11139,PTHR11139:SF66	0.051	NA	NA	NA	NA	NA	0.00498
ATM	11	108098598	T	G	NA	NO	missense	ENST00000278616.4:c.168T>G	ENSP00000278616.4:p.Asn56Lys	N56K	Pfam_domain:PF11640,PTHR11139,PTHR11139:SF66	26.3	probably_damaging	0.95	deleterious	0.04	15	0.00000
ATM	11	108098606	C	T	rs760880388	NO	missense	ENST00000278616.4:c.176C>T	ENSP00000278616.4:p.Ala59Val	A59V	Pfam_domain:PF11640,PTHR11139,PTHR11139:SF66	26.3	possibly_damaging	0.826	tolerated	0.05	0	0.00000
ATM	11	108098614	A	G	NA	NO	missense,splice	ENST00000278616.4:c.184A>G	ENSP00000278616.4:p.Arg62Gly	R62G	Pfam_domain:PF11640,PTHR11139,PTHR11139:SF66	26.9	probably_damaging	0.938	deleterious	0.01	0	0.00000
ATM	11	108098623	A	G	rs753891198,COSM3979080,COSM3979079	NO	splice,intron	ENST00000278616.4:c.185+8A>G	NA	NA	NA	9.836	NA	NA	NA	NA	NA	0.00000
ATM	11	108098640	T	A	NA	NO	intron	ENST00000278616.4:c.185+25T>A	NA	NA	NA	13.54	NA	NA	NA	NA	NA	0.00000
ATM	11	108098655	C	CTA	NA	NO	intron	ENST00000278616.4:c.185+41_185+42insAT	NA	NA	NA	8.097	NA	NA	NA	NA	NA	0.00000
ATM	11	108098660	A	G	rs759014058	NO	intron	ENST00000278616.4:c.185+45A>G	NA	NA	NA	2.911	NA	NA	NA	NA	NA	0.00000
ATM	11	108099844	T	G	NA	NO	intron	ENST00000278616.4:c.186-61T>G	NA	NA	NA	1.023	NA	NA	NA	NA	NA	0.00000
ATM	11	108099859	TTAAG	T	rs776876770	NO	intron	ENST00000278616.4:c.186-43_186-40delAGTA	NA	NA	NA	8.784	NA	NA	NA	NA	NA	0.00000
ATM	11	108099870	A	G	NA	NO	intron	ENST00000278616.4:c.186-35A>G	NA	NA	NA	11.34	NA	NA	NA	NA	NA	0.00000
ATM	11	108099871	C	T	rs371745892	NO	intron	ENST00000278616.4:c.186-34C>T	NA	NA	NA	10.83	NA	NA	NA	NA	NA	0.00000
ATM	11	108099872	G	A	rs777293439	NO	intron	ENST00000278616.4:c.186-33G>A	NA	NA	NA	11.91	NA	NA	NA	NA	NA	0.00000
ATM	11	108099886	G	A	rs748791429	NO	intron	ENST00000278616.4:c.186-19G>A	NA	NA	NA	9.11	NA	NA	NA	NA	NA	0.00000
ATM	11	108099944	G	A	NA	NO	synonymous	ENST00000278616.4:c.225G>A	NA	L75L	Pfam_domain:PF11640,PTHR11139,PTHR11139:SF66	8.402	NA	NA	NA	NA	NA	0.00000
ATM	11	108099970	C	T	rs587781937	NO	missense	ENST00000278616.4:c.251C>T	ENSP00000278616.4:p.Ala84Val	A84V	Pfam_domain:PF11640,PTHR11139,PTHR11139:SF66	23.2	benign	0.119	deleterious	0.05	0	0.00000
ATM	11	108099991	A	C	NA	NO	missense	ENST00000278616.4:c.272A>C	ENSP00000278616.4:p.Gln91Pro	Q91P	Pfam_domain:PF11640,PTHR11139,PTHR11139:SF66	25.5	probably_damaging	0.953	deleterious	0.01	0	0.00000
ATM	11	108099993	A	C	rs756969590	NO	missense	ENST00000278616.4:c.274A>C	ENSP00000278616.4:p.Lys92Gln	K92Q	Pfam_domain:PF11640,PTHR11139,PTHR11139:SF66	25.6	probably_damaging	0.998	deleterious	0.02	0	0.00000
ATM	11	108100010	C	T	rs746762110	NO	synonymous	ENST00000278616.4:c.291C>T	NA	I97I	Pfam_domain:PF11640,PTHR11139,PTHR11139:SF66	13.08	NA	NA	NA	NA	NA	0.00006
ATM	11	108100014	A	G	rs137882485,CM0910478	NO	missense	ENST00000278616.4:c.295A>G	ENSP00000278616.4:p.Ser99Gly	S99G	Pfam_domain:PF11640,PTHR11139,PTHR11139:SF66	23.3	benign	0.168	deleterious	0.03	0	0.00041
ATM	11	108100039	G	A	CM030778,rs142358238	NO	missense	ENST00000278616.4:c.320G>A	ENSP00000278616.4:p.Cys107Tyr	C107Y	Pfam_domain:PF11640,PTHR11139,PTHR11139:SF66	16.18	benign	0.158	tolerated	0.46	0	0.00000
ATM	11	108106371	T	C	NA	NO	intron	ENST00000278616.4:c.332-26T>C	NA	NA	NA	1.662	NA	NA	NA	NA	NA	0.00000
ATM	11	108106379	T	A	NA	NO	intron	ENST00000278616.4:c.332-18T>A	NA	NA	NA	10.7	NA	NA	NA	NA	NA	0.00000
ATM	11	108106399	G	A	rs146382972	NO	missense,splice	ENST00000278616.4:c.334G>A	ENSP00000278616.4:p.Ala112Thr	A112T	Pfam_domain:PF11640,PTHR11139,PTHR11139:SF66	23	benign	0.41	tolerated	0.06	0	0.00000
ATM	11	108106403	C	T	NA	NO	missense	ENST00000278616.4:c.338C>T	ENSP00000278616.4:p.Pro113Leu	P113L	Pfam_domain:PF11640,PTHR11139,PTHR11139:SF66	33	probably_damaging	1	deleterious	0	65	0.00000
ATM	11	108106435	A	G	CD000908,rs148590073	NO	missense	ENST00000278616.4:c.370A>G	ENSP00000278616.4:p.Ile124Val	I124V	Pfam_domain:PF11640,PTHR11139,PTHR11139:SF66	0.491	benign	0.006	tolerated	1	0	0.00024
ATM	11	108106437	C	A	rs773495195	NO	synonymous	ENST00000278616.4:c.372C>A	NA	I124I	Pfam_domain:PF11640,PTHR11139,PTHR11139:SF66	17.97	NA	NA	NA	NA	NA	0.00006
ATM	11	108106443	T	A	rs587781449,rs2234997,CM077897,COSM224	NO	missense	ENST00000278616.4:c.378T>A	ENSP00000278616.4:p.Asp126Glu	D126E	Pfam_domain:PF11640,PTHR11139,PTHR11139:SF66	4.635	benign	0.006	tolerated	1	0	0.00324
ATM	11	108106463	A	G	rs730881330	NO	missense	ENST00000278616.4:c.398A>G	ENSP00000278616.4:p.Asn133Ser	N133S	Pfam_domain:PF11640,PTHR11139,PTHR11139:SF66	0.001	benign	0.006	tolerated	1	0	0.00000
ATM	11	108106466	G	A	rs771166271	NO	missense	ENST00000278616.4:c.401G>A	ENSP00000278616.4:p.Gly134Asp	G134D	Pfam_domain:PF11640,PTHR11139,PTHR11139:SF66	14.5	possibly_damaging	0.493	tolerated	0.42	0	0.00000
ATM	11	108106565	T	C	rs587781375	NO	splice,intron	ENST00000278616.4:c.496+4T>C	NA	NA	NA	4.514	NA	NA	NA	NA	NA	0.00000
ATM	11	108114609	G	A	NA	NO	intron	ENST00000278616.4:c.497-71G>A	NA	NA	NA	2.263	NA	NA	NA	NA	NA	0.00000
ATM	11	108114632	T	G	rs2235001	NO	intron	ENST00000278616.4:c.497-48T>G	NA	NA	NA	0.205	NA	NA	NA	NA	NA	0.00000
ATM	11	108114637	A	G	NA	NO	intron	ENST00000278616.4:c.497-43A>G	NA	NA	NA	1.119	NA	NA	NA	NA	NA	0.00000
ATM	11	108114655	C	G	NA	NO	intron	ENST00000278616.4:c.497-25C>G	NA	NA	NA	1.705	NA	NA	NA	NA	NA	0.00000
ATM	11	108114666	T	A	rs757147178	NO	intron	ENST00000278616.4:c.497-14T>A	NA	NA	NA	2.356	NA	NA	NA	NA	NA	0.00000
ATM	11	108114680	A	T	NA	NO	missense,splice	ENST00000278616.4:c.497A>T	ENSP00000278616.4:p.Glu166Val	E166V	PTHR11139,PTHR11139:SF66	23.2	benign	0.219	deleterious	0.01	15	0.00000
ATM	11	108114727	G	C	CM035638,rs3218707	NO	missense	ENST00000278616.4:c.544G>C	ENSP00000278616.4:p.Val182Leu	V182L	PTHR11139,PTHR11139:SF66	12.39	benign	0.009	tolerated	0.2	0	0.00223
ATM	11	108114773	G	A	CM093706,rs753806542	NO	missense	ENST00000278616.4:c.590G>A	ENSP00000278616.4:p.Gly197Glu	G197E	PTHR11139,PTHR11139:SF66	27.9	probably_damaging	0.999	deleterious	0.01	65	0.00000
ATM	11	108114792	C	T	rs144709948,CM0910480	NO	synonymous	ENST00000278616.4:c.609C>T	NA	D203D	PTHR11139,PTHR11139:SF66	14.94	NA	NA	NA	NA	NA	0.00438
ATM	11	108114793	G	A	rs147915571,CM056524,COSM4017609,COSN	NO	missense	ENST00000278616.4:c.610G>A	ENSP00000278616.4:p.Gly204Arg	G204R	PTHR11139,PTHR11139:SF66	23.7	possibly_damaging	0.609	tolerated	0.44	0	0.00027
ATM	11	108114829	G	T	rs2235002	NO	missense	ENST00000278616.4:c.646G>T	ENSP00000278616.4:p.Ala216Ser	A216S	PTHR11139,PTHR11139:SF66	20.8	benign	0.42	tolerated	0.23	0	0.00003
ATM	11	108114840	T	C	rs2235003	NO	synonymous	ENST00000278616.4:c.657T>C	NA	C219C	PTHR11139,PTHR11139:SF66	0.007	NA	NA	NA	NA	NA	0.00027
ATM	11	108114859	T	A	NA	NO	intron	ENST00000278616.4:c.662+14T>A	NA	NA	NA	4.518	NA	NA	NA	NA	NA	0.00000
ATM	11	108114883	T	C	rs2235004	NO	intron	ENST00000278616.4:c.662+38T>C	NA	NA	NA	5.368	NA	NA	NA	NA	NA	0.00000
ATM	11	108114887	T	A	rs762688194	NO	intron	ENST00000278616.4:c.662+42T>A	NA	NA	NA	7.911	NA	NA	NA	NA	NA	0.00000
ATM	11	108115483	A	G	NA	NO	intron	ENST00000278616.4:c.663-32A>G	NA	NA	NA	1.573	NA	NA	NA	NA	NA	0.00000
ATM	11	108115522	A	G	rs145053092,CM000650	NO	missense	ENST00000278616.4:c.670A>G	ENSP00000278616.4:p.Lys224Glu	K224E	PTHR11139,PTHR11139:SF66	23.5	benign	0.157	deleterious	0.02	15	0.00021
ATM	11	108115580	T	C	COSM117860	NO	missense	ENST00000278616.4:c.728T>C	ENSP00000278616.4:p.Leu243Ser	L243S	PTHR11139,PTHR11139:SF66	15.13	benign	0.006	tolerated	0.21	0	0.00000
ATM	11	108115587	C	T	rs3218674	NO	synonymous	ENST00000278616.4:c.735C>T	NA	V245V	PTHR11139,PTHR11139:SF66	11.67	NA	NA	NA	NA	NA	0.03115
ATM	11	108115600	C	T	rs772821016,CM030188,CS991299,COSM173	YES	stop_gained	ENST00000278616.4:c.748C>T	ENSP00000278616.4:p.Arg250Ter	R250*	PTHR11139,PTHR11139:SF66	36	NA	NA	NA	NA	NA	0.00000
ATM	11	108115601	G	A	CM092584,rs56123940	NO	missense	ENST00000278616.4:c.749G>A	ENSP00000278616.4:p.Arg250Gln	R250Q	PTHR11139,PTHR11139:SF66	13.4	benign	0.02	tolerated	0.36	0	0.00015
ATM	11	108115639	CT	C	rs774609577	YES	frameshift,feature_truncation	ENST00000278616.4:c.790delT	ENSP00000278616.4:p.Tyr264IlefsTer12	L263X	Superfamily_domains:SSF48371,PTHR11139,PTHR11139:SF66	31	NA	NA	NA	NA	NA	0.00003
ATM	11	108115657	CAT	C	NA	YES	frameshift,feature_truncation	ENST00000278616.4:c.807_808delTA	ENSP00000278616.4:p.His269GlnfsTer3	H269X	Superfamily_domains:SSF48371,PTHR11139,PTHR11139:SF66	34	NA	NA	NA	NA	NA	0.00000
ATM	11	108115663	C	A	rs730881339	NO	missense	ENST00000278616.4:c.811C>A	ENSP00000278616.4:p.Leu271Ile	L271I	Superfamily_domains:SSF48371,PTHR11139,PTHR11139:SF66	18.06	benign	0.32	tolerated	0.24	0	0.00003
ATM	11	108115673	CT	C	NA	YES	frameshift,feature_truncation	ENST00000278616.4:c.824delT	ENSP00000278616.4:p.Leu275Ter	S274X	Superfamily_domains:SSF48371,PTHR11139,PTHR11139:SF66	23.9	NA	NA	NA	NA	NA	0.00000
ATM	11	108115698	A	T	rs754008850	NO	missense	ENST00000278616.4:c.846A>T	ENSP00000278616.4:p.Leu282Phe	L282F	Superfamily_domains:SSF48371,PTHR11139,PTHR11139:SF66	6.125	benign	0.009	tolerated	1	0	0.00000
ATM	11	108115703	A	T	NA	NO	missense	ENST00000278616.4:c.851A>T	ENSP00000278616.4:p.Gln284Leu	Q284L	Superfamily_domains:SSF48371,PTHR11139,PTHR11139:SF66	16.06	benign	0.003	tolerated	0.29	0	0.00000
ATM	11	108115706	T	G	NA	NO	missense	ENST00000278616.4:c.854T>G	ENSP00000278616.4:p.Leu285Arg	L285R	Superfamily_domains:SSF48371,PTHR11139,PTHR11139:SF66	28.5	probably_damaging	0.977	deleterious	0	65	0.00000
ATM	11	108115718	T	C	rs35261362	NO	missense	ENST00000278616.4:c.866T>C	ENSP00000278616.4:p.Ile289Thr	I289T	Superfamily_domains:SSF48371,PTHR11139,PTHR11139:SF66	22.9	benign	0.097	deleterious	0.03	0	0.00000
ATM	11	108115728	G	A	rs755860432	NO	synonymous	ENST00000278616.4:c.876G>A	NA	P292P	Superfamily_domains:SSF48371,PTHR11139,PTHR11139:SF66	12.23	NA	NA	NA	NA	NA	0.00003
ATM	11	108115778	T	G	rs1800735	NO	intron	ENST00000278616.4:c.901+25T>G	NA	NA	NA	5.574	NA	NA	NA	NA	NA	0.00000
ATM	11	108117681	T	C	NA	NO	intron	ENST00000278616.4:c.902-10T>C	NA	NA	NA	3.225	NA	NA	NA	NA	NA	0.00000
ATM	11	108117691	G	A	rs202208861,CM0910482	NO	missense,splice	ENST00000278616.4:c.902G>A	ENSP00000278616.4:p.Gly301Asp	G301D	Superfamily_domains:SSF48371,PTHR11139,PTHR11139:SF66	23.8	benign	0.374	deleterious	0.01	65	0.00015
ATM	11	108117714	A	T	NA	YES	stop_gained	ENST00000278616.4:c.925A>T	ENSP00000278616.4:p.Arg309Ter	R309*	Superfamily_domains:SSF48371,PTHR11139,PTHR11139:SF66	36	NA	NA	NA	NA	NA	0.00000
ATM	11	108117735	T	C	rs142317485	NO	missense	ENST00000278616.4:c.946T>C	ENSP00000278616.4:p.Tyr316His	Y316H	Superfamily_domains:SSF48371,PTHR11139,PTHR11139:SF66	27.3	probably_damaging	0.986	deleterious	0	35	0.00009
ATM	11	108117747	G	A	NA	NO	missense	ENST00000278616.4:c.958G>A	ENSP00000278616.4:p.Val320Met	V320M	Superfamily_domains:SSF48371,PTHR11139,PTHR11139:SF66	24.9	possibly_damaging	0.513	deleterious	0.01	0	0.00000
ATM	11	108117767	A	G	NA	NO	missense	ENST00000278616.4:c.978A>G	ENSP00000278616.4:p.Ile326Met	I326M	Superfamily_domains:SSF48371,PTHR11139,PTHR11139:SF66	23.2	probably_damaging	0.954	tolerated	0.05	0	0.00000
ATM	11	108117787	C	T	rs28904919	NO	missense	ENST00000278616.4:c.998C>T	ENSP00000278616.4:p.Ser333Phe	S333F	Superfamily_domains:SSF48371,PTHR11139,PTHR11139:SF66	25.9	possibly_damaging	0.602	deleterious	0.01	15	0.00280
ATM	11	108117798	C	T	CM0910483,rs138398778,COSM21323,COSM	NO	missense	ENST00000278616.4:c.1009C>T	ENSP00000278616.4:p.Arg337Cys	R337C	Superfamily_domains:SSF48371,PTHR11139,PTHR11139:SF66	35	probably_damaging	0.999	deleterious	0	55	0.00027
ATM	11	108117799	G	A	rs202160435,COSM1235401,COSM21301,COS	NO	missense	ENST00000278616.4:c.1010G>A	ENSP00000278616.4:p.Arg337His	R337H	Superfamily_domains:SSF48371,PTHR11139,PTHR11139:SF66	34	probably_damaging	0.995	deleterious	0.02	0	0.00027
ATM	11	108117840	G	A	COSM922703,COSM1585367	NO	missense	ENST00000278616.4:c.1051G>A	ENSP00000278616.4:p.Asp351Asn	D351N	Superfamily_domains:SSF48371,PTHR11139,PTHR11139:SF66	27	probably_damaging	0.983	tolerated	0.05	15	0.00000
ATM	11	108117952	G	A	NA	NO	intron	ENST00000278616.4:c.1065+98G>A	NA	NA	NA	4.007	NA	NA	NA	NA	NA	0.00000
ATM	11	108119581	C	T	NA	NO	intron	ENST00000278616.4:c.1066-79C>T	NA	NA	NA	2.072	NA	NA	NA	NA	NA	0.00000
ATM	11	108119586	A	G	NA	NO	intron	ENST00000278616.4:c.1066-74A>G	NA	NA	NA	1.944	NA	NA	NA	NA	NA	0.00000
ATM	11	108119595	A	G	NA	NO	intron	ENST00000278616.4:c.1066-65A>G	NA	NA	NA	1.083	NA	NA	NA	NA	NA	0.00000
ATM	11	108119599	C	T	NA	NO	intron	ENST00000278616.4:c.1066-61C>T	NA	NA	NA	0.777	NA	NA	NA	NA	NA	0.00000
ATM	11	108119602	C	T	NA	NO	intron	ENST00000278616.4:c.1066-58C>T	NA	NA	NA	0.695	NA	NA	NA	NA	NA	0.00000
ATM	11	108119603	G	A	rs4987935	NO	intron	ENST00000278616.4:c.1066-57G>A	NA	NA	NA	0.647	NA	NA	NA	NA	NA	0.00000
ATM	11	108119654	T	G	rs201686625,CS008846,COSM758349	NO	splice,intron	ENST00000278616.4:c.1066-6T>G	NA	NA	NA	11.93	NA	NA	NA	NA	NA	0.00000
ATM	11	108119667	A	G	rs149636614	NO	missense	ENST00000278616.4:c.1073A>G	ENSP00000278616.4:p.Asn358Ser	N358S	Superfamily_domains:SSF48371,PTHR11139,PTHR11139:SF66	0.284	benign	0.001	tolerated	0.95	0	0.00006
ATM	11	108119687	G	T	NA	YES	stop_gained	ENST00000278616.4:c.1093G>T	ENSP00000278616.4:p.Glu365Ter	E365*	PTHR11139,PTHR11139:SF66	38	NA	NA	NA	NA	NA	0.00000
ATM	11	108119702	T	TA	NA	YES	frameshift,feature_elongation	ENST00000278616.4:c.1109dupA	ENSP00000278616.4:p.Tyr370Ter	Y370*	PTHR11139,PTHR11139:SF66	25.7	NA	NA	NA	NA	NA	0.00000
ATM	11	108119704	C	T	rs376170600,CM031634,CD991597	NO	synonymous	ENST00000278616.4:c.1110C>T	NA	Y370Y	PTHR11139,PTHR11139:SF66	0.158	NA	NA	NA	NA	NA	0.00000
ATM	11	108119732	T	A	rs34083085	NO	missense	ENST00000278616.4:c.1138T>A	ENSP00000278616.4:p.Tyr380Asn	Y380N	PTHR11139,PTHR11139:SF66	0.213	benign	0.021	tolerated	0.38	0	0.00003
ATM	11	108119740	C	T	NA	NO	synonymous	ENST00000278616.4:c.1146C>T	NA	V382V	PTHR11139,PTHR11139:SF66	8.232	NA	NA	NA	NA	NA	0.00000
ATM	11	108119770	C	G	rs1800727	NO	synonymous	ENST00000278616.4:c.1176C>G	NA	G392G	PTHR11139,PTHR11139:SF66	12.47	NA	NA	NA	NA	NA	0.00033
ATM	11	108119791	C	T	NA	NO	synonymous	ENST00000278616.4:c.1197C>T	NA	H399H	PTHR11139,PTHR11139:SF66	13	NA	NA	NA	NA	NA	0.00000
ATM	11	108119823	T	C	CM035782,rs56128736,COSM1350756,COSM	NO	missense	ENST00000278616.4:c.1229T>C	ENSP00000278616.4:p.Val410Ala	V410A	PTHR11139,PTHR11139:SF66	23.2	benign	0.22	deleterious	0.01	25	0.00649
ATM	11	108119839	T	TA	rs773187419	NO	intron,feature_elongation	ENST00000278616.4:c.1235+11dupA	NA	NA	NA	10.84	NA	NA	NA	NA	NA	0.00000
ATM	11	108119849	T	G	rs762656752	NO	intron	ENST00000278616.4:c.1235+20T>G	NA	NA	NA	7.632	NA	NA	NA	NA	NA	0.00000
ATM	11	108121446	A	G	rs4987943	NO	synonymous	ENST00000278616.4:c.1254A>G	NA	Q418Q	PTHR11139,PTHR11139:SF66	0.816	NA	NA	NA	NA	NA	0.00153
ATM	11	108121464	T	C	rs35578748	NO	synonymous	ENST00000278616.4:c.1272T>C	NA	P424P	PTHR11139,PTHR11139:SF66	3.835	NA	NA	NA	NA	NA	0.00091
ATM	11	108121479	CTG	C	NA	YES	frameshift,feature_truncation	ENST00000278616.4:c.1290_1291delTG	ENSP00000278616.4:p.Cys430Ter	C430X	PTHR11139,PTHR11139:SF66	24.8	NA	NA	NA	NA	NA	0.00000
ATM	11	108121524	C	A	rs763361384	NO	synonymous	ENST00000278616.4:c.1332C>A	NA	P444P	Low_complexity_(Seg):seg,PTHR11139,PTHR11139:SF66	13.76	NA	NA	NA	NA	NA	0.00000
ATM	11	108121527	A	G	NA	NO	synonymous	ENST00000278616.4:c.1335A>G	NA	Q445Q	Low_complexity_(Seg):seg,PTHR11139,PTHR11139:SF66	4.486	NA	NA	NA	NA	NA	0.00000
ATM	11	108121531	C	T	rs587779815,CM960098,COSM3733240,COSN	YES	stop_gained	ENST00000278616.4:c.1339C>T	ENSP00000278616.4:p.Arg447Ter	R447*	PTHR11139,PTHR11139:SF66	39	NA	NA	NA	NA	NA	0.00000
ATM	11	108121543	C	T	rs201719927,COSM1321925,COSM1321924	NO	missense	ENST00000278616.4:c.1351C>T	ENSP00000278616.4:p.Arg451Cys	R451C	PTHR11139,PTHR11139:SF66	33	probably_damaging	0.994	deleterious	0.03	25	0.00009
ATM	11	108121546	AC	A	rs201333862,rs587781776,CD982449	YES	frameshift,feature_truncation	ENST00000278616.4:c.1355delC	ENSP00000278616.4:p.Thr452AsnfsTer21	T452X	PTHR11139,PTHR11139:SF66	24.8	NA	NA	NA	NA	NA	0.00000
ATM	11	108121546	A	G	NA	NO	missense	ENST00000278616.4:c.1354A>G	ENSP00000278616.4:p.Thr452Ala	T452A	PTHR11139,PTHR11139:SF66	4.082	benign	0.005	tolerated	0.41	0	0.00000
ATM	11	108121551	A	T	NA	NO	synonymous	ENST00000278616.4:c.1359A>T	NA	P453P	PTHR11139,PTHR11139:SF66	0.007	NA	NA	NA	NA	NA	0.00000
ATM	11	108121559	T	TA	NA	YES	frameshift,feature_elongation	ENST00000278616.4:c.1368dupA	ENSP00000278616.4:p.Arg457ThrfsTer30	L456LX	PTHR11139,PTHR11139:SF66	29.2	NA	NA	NA	NA	NA	0.00000
ATM	11	108121570	A	C	rs587782729	NO	missense	ENST00000278616.4:c.1378A>C	ENSP00000278616.4:p.Thr460Pro	T460P	PTHR11139,PTHR11139:SF66	8.091	benign	0.078	tolerated	0.22	0	0.00000
ATM	11	108121593	CAA	C	rs587781347,COSM758338,COSM1289451	YES	frameshift,feature_truncation	ENST00000278616.4:c.1402_1403delAA	ENSP00000278616.4:p.Lys468GlufsTer18	K468X	PTHR11139,PTHR11139:SF66	28.3	NA	NA	NA	NA	NA	0.00006
ATM	11	108121608	A	G	COSM127379	NO	synonymous	ENST00000278616.4:c.1416A>G	NA	L472L	PTHR11139,PTHR11139:SF66	2.68	NA	NA	NA	NA	NA	0.00000
ATM	11	108121632	A	C	rs370240037	NO	missense	ENST00000278616.4:c.1440A>C	ENSP00000278616.4:p.Leu480Phe	L480F	PTHR11139,PTHR11139:SF66	23.8	probably_damaging	0.996	deleterious	0.02	0	0.00003
ATM	11	108121635	A	C	rs753808755	NO	missense	ENST00000278616.4:c.1443A>C	ENSP00000278616.4:p.Leu481Phe	L481F	PTHR11139,PTHR11139:SF66	8.643	benign	0.058	tolerated	0.71	0	0.00000
ATM	11	108121636	A	C	rs202173660,COSM922707,COSM1585363	NO	missense	ENST00000278616.4:c.1444A>C	ENSP00000278616.4:p.Lys482Gln	K482Q	PTHR11139,PTHR11139:SF66	17.59	benign	0.342	tolerated	0.08	0	0.00024
ATM	11	108121656	G	T	rs377597949	NO	missense	ENST00000278616.4:c.1464G>T	ENSP00000278616.4:p.Trp488Cys	W488C	PTHR11139,PTHR11139:SF66	23.7	benign	0.242	deleterious	0.05	65	0.00009
ATM	11	108121687	C	T	COSM267932	YES	stop_gained	ENST00000278616.4:c.1495C>T	ENSP00000278616.4:p.Gln499Ter	Q499*	PTHR11139,PTHR11139:SF66	38	NA	NA	NA	NA	NA	0.00000
ATM	11	108121708	G	T	rs587779816	NO	missense	ENST00000278616.4:c.1516G>T	ENSP00000278616.4:p.Gly506Cys	G506C	PTHR11139,PTHR11139:SF66	29	possibly_damaging	0.67	deleterious	0.01	15	0.00021
ATM	11	108121718	G	A	NA	NO	missense	ENST00000278616.4:c.1526G>A	ENSP00000278616.4:p.Gly509Glu	G509E	PTHR11139,PTHR11139:SF66	16.19	benign	0.027	tolerated	0.15	0	0.00000
ATM	11	108121733	G	A	CM024583,rs2235000	NO	missense	ENST00000278616.4:c.1541G>A	ENSP00000278616.4:p.Gly514Asp	G514D	PTHR11139,PTHR11139:SF66	23.6	possibly_damaging	0.493	tolerated	0.3	0	0.00024
ATM	11	108121754	CAG	C	NA	YES	frameshift,feature_truncation	ENST00000278616.4:c.1564_1565delGA	ENSP00000278616.4:p.Glu522IlefsTer43	E522fs	PTHR11139,PTHR11139:SF66	NA	NA	NA	NA	NA	NA	0.00000
ATM	11	108121787	G	A	rs35963548	NO	missense	ENST00000278616.4:c.1595G>A	ENSP00000278616.4:p.Cys532Tyr	C532Y	PTHR11139,PTHR11139:SF66	23.7	benign	0.062	deleterious	0	0	0.00027
ATM	11	108122529	C	T	NA	NO	intron	ENST00000278616.4:c.1608-35C>T	NA	NA	NA	4.379	NA	NA	NA	NA	NA	0.00000
ATM	11	108122538	C	T	NA	NO	intron	ENST00000278616.4:c.1608-26C>T	NA	NA	NA	4.226	NA	NA	NA	NA	NA	0.00000
ATM	11	108122542	A	G	rs747703201	NO	intron	ENST00000278616.4:c.1608-22A>G	NA	NA	NA	4.746	NA	NA	NA	NA	NA	0.00000
ATM	11	108122583	A	G	NA	NO	missense	ENST00000278616.4:c.1627A>G	ENSP00000278616.4:p.Thr543Ala	T543A	PTHR11139,PTHR11139:SF66	6.153	benign	0.001	tolerated	0.78	0	0.00000
ATM	11	108122592	C	G	CM0910485,rs2227924	NO	missense	ENST00000278616.4:c.1636C>G	ENSP00000278616.4:p.Leu546Val	L546V	PTHR11139,PTHR11139:SF66	13.24	benign	0.287	deleterious	0.01	0	0.00039
ATM	11	108122617	C	T	NA	NO	missense	ENST00000278616.4:c.1661C>T	ENSP00000278616.4:p.Thr554Met	T554M	PTHR11139,PTHR11139:SF66	12.45	benign	0.018	tolerated	0.26	0	0.00000
ATM	11	108122622	A	G	NA	NO	missense	ENST00000278616.4:c.1666A>G	ENSP00000278616.4:p.Lys556Glu	K556E	PTHR11139,PTHR11139:SF66	0.021	benign	0.001	tolerated	0.93	0	0.00000
ATM	11	108122644	T	G	rs750815208,COSM1506638,COSM1506637	NO	missense	ENST00000278616.4:c.1688T>G	ENSP00000278616.4:p.Met563Arg	M563R	PTHR11139,PTHR11139:SF66	2.309	benign	0.039	tolerated	0.23	0	0.00003
ATM	11	108122659	G	T	rs200381392	NO	missense	ENST00000278616.4:c.1703G>T	ENSP00000278616.4:p.Arg568Ile	R568I	PTHR11139,PTHR11139:SF66	15.26	benign	0.042	tolerated	0.2	0	0.00042
ATM	11	108122683	T	C	rs730881342	NO	missense	ENST00000278616.4:c.1727T>C	ENSP00000278616.4:p.Ile576Thr	I576T	PTHR11139,PTHR11139:SF66	23.5	benign	0.424	deleterious	0.01	65	0.00042
ATM	11	108122693	G	A	NA	YES	stop_gained	ENST00000278616.4:c.1737G>A	ENSP00000278616.4:p.Trp579Ter	W579*	PTHR11139,PTHR11139:SF66	37	NA	NA	NA	NA	NA	0.00000
ATM	11	108122694	C	T	NA	NO	missense	ENST00000278616.4:c.1738C>T	ENSP00000278616.4:p.Leu580Phe	L580F	PTHR11139,PTHR11139:SF66	26.3	possibly_damaging	0.786	deleterious	0.01	0	0.00000
ATM	11	108122700	T	C	CM023329,rs2235006	NO	missense	ENST00000278616.4:c.1744T>C	ENSP00000278616.4:p.Phe582Leu	F582L	PTHR11139,PTHR11139:SF66	3.12	benign	0.001	tolerated	1	0	0.00201
ATM	11	108122704	A	G	rs587780614	NO	missense	ENST00000278616.4:c.1748A>G	ENSP00000278616.4:p.Tyr583Cys	Y583C	PTHR11139,PTHR11139:SF66	2.663	benign	0.001	tolerated	0.19	0	0.00000
ATM	11	108122729	T	C	rs61734356	NO	synonymous	ENST00000278616.4:c.1773T>C	NA	N591N	PTHR11139,PTHR11139:SF66	0.12	NA	NA	NA	NA	NA	0.00045
ATM	11	108122797	T	C	rs752359325	NO	intron	ENST00000278616.4:c.1802+39T>C	NA	NA	NA	7.764	NA	NA	NA	NA	NA	0.00000
ATM	11	108123551	C	T	CM045888,rs2227922,COSM1350778,COSM2	NO	missense	ENST00000278616.4:c.1810C>T	ENSP00000278616.4:p.Pro604Ser	P604S	PTHR11139,PTHR11139:SF66	24.3	possibly_damaging	0.53	tolerated	0.1	0	0.00742
ATM	11	108123582	G	C	rs770377706,COSM200656	NO	missense	ENST00000278616.4:c.1841G>C	ENSP00000278616.4:p.Ser614Thr	S614T	PTHR11139,PTHR11139:SF66	23.6	possibly_damaging	0.764	tolerated	0.21	0	0.00003
ATM	11	108123596	A	C	rs140882609	NO	missense	ENST00000278616.4:c.1855A>C	ENSP00000278616.4:p.Asn619His	N619H	PTHR11139,PTHR11139:SF66	17.52	benign	0.031	deleterious	0.01	0	0.00003
ATM	11	108123598	CTG	C	NA	YES	frameshift,feature_truncation	ENST00000278616.4:c.1859_1860delGT	ENSP00000278616.4:p.Cys620Ter	C620X	PTHR11139,PTHR11139:SF66	32	NA	NA	NA	NA	NA	0.00000
ATM	11	108123621	T	G	rs546087885,CM023330	NO	missense	ENST00000278616.4:c.1880T>G	ENSP00000278616.4:p.Phe627Cys	F627C	PTHR11139,PTHR11139:SF66	23.4	benign	0.08	deleterious	0.05	15	0.00006
ATM	11	108123633	C	T	rs756782634	NO	missense	ENST00000278616.4:c.1892C>T	ENSP00000278616.4:p.Pro631Leu	P631L	PTHR11139,PTHR11139:SF66	22.1	benign	0.001	tolerated	0.12	0	0.00003
ATM	11	108123636	A	G	NA	NO	missense	ENST00000278616.4:c.1895A>G	ENSP00000278616.4:p.Glu632Gly	E632G	PTHR11139,PTHR11139:SF66	18.19	benign	0	tolerated	0.22	0	0.00000
ATM	11	108123680	G	A	NA	NO	intron	ENST00000278616.4:c.1898+41G>A	NA	NA	NA	8.548	NA	NA	NA	NA	NA	0.00000
ATM	11	108123695	A	G	NA	NO	intron	ENST00000278616.4:c.1898+56A>G	NA	NA	NA	1.714	NA	NA	NA	NA	NA	0.00000
ATM	11	108123699	T	C	NA	NO	intron	ENST00000278616.4:c.1898+60T>C	NA	NA	NA	3.969	NA	NA	NA	NA	NA	0.00000
ATM	11	108124517	A	G	rs771353416	NO	intron	ENST00000278616.4:c.1899-24A>G	NA	NA	NA	5.1	NA	NA	NA	NA	NA	0.00000
ATM	11	108124525	T	A	NA	NO	intron	ENST00000278616.4:c.1899-16T>A	NA	NA	NA	6.024	NA	NA	NA	NA	NA	0.00000
ATM	11	108124527	T	C	NA	NO	intron	ENST00000278616.4:c.1899-14T>C	NA	NA	NA	0.482	NA	NA	NA	NA	NA	0.00000
ATM	11	108124571	T	A	NA	NO	synonymous	ENST00000278616.4:c.1929T>A	NA	L643L	Low_complexity_(Seg):seg,PTHR11139,PTHR11139:SF66	9.095	NA	NA	NA	NA	NA	0.00000
ATM	11	108124582	A	G	NA	NO	missense	ENST00000278616.4:c.1940A>G	ENSP00000278616.4:p.Glu647Gly	E647G	Low_complexity_(Seg):seg,PTHR11139,PTHR11139:SF66	23.9	benign	0.341	deleterious	0.01	0	0.00000
ATM	11	108124595	A	G	rs730881283	NO	synonymous	ENST00000278616.4:c.1953A>G	NA	L651L	Low_complexity_(Seg):seg,PTHR11139,PTHR11139:SF66	8.608	NA	NA	NA	NA	NA	0.00003
ATM	11	108124602	C	A	CM0910487,rs528165789	NO	missense	ENST00000278616.4:c.1960C>A	ENSP00000278616.4:p.Gln654Lys	Q654K	PTHR11139,PTHR11139:SF66	14.54	benign	0.005	tolerated	0.32	0	0.00009
ATM	11	108124628	T	C	rs1800055	NO	synonymous	ENST00000278616.4:c.1986T>C	NA	F662F	PTHR11139,PTHR11139:SF66	0.337	NA	NA	NA	NA	NA	0.00144
ATM	11	108124635	A	C	NA	NO	missense	ENST00000278616.4:c.1993A>C	ENSP00000278616.4:p.Ile665Leu	I665L	PTHR11139,PTHR11139:SF66	0.001	benign	0.004	tolerated	0.75	0	0.00000
ATM	11	108124640	G	A	rs748380150	NO	synonymous	ENST00000278616.4:c.1998G>A	NA	V666V	PTHR11139,PTHR11139:SF66	0.863	NA	NA	NA	NA	NA	0.00003
ATM	11	108124651	G	A	NA	NO	missense	ENST00000278616.4:c.2009G>A	ENSP00000278616.4:p.Gly670Asp	G670D	PTHR11139,PTHR11139:SF66	0.002	benign	0.001	tolerated	0.62	0	0.00000
ATM	11	108124661	G	A	NA	NO	synonymous	ENST00000278616.4:c.2019G>A	NA	K673K	PTHR11139,PTHR11139:SF66	3.944	NA	NA	NA	NA	NA	0.00000
ATM	11	108124663	A	G	rs201762714	NO	missense	ENST00000278616.4:c.2021A>G	ENSP00000278616.4:p.His674Arg	H674R	PTHR11139,PTHR11139:SF66	0.001	benign	0	tolerated	0.35	0	0.00015
ATM	11	108124669	CCAGTATTGG	CACTGGA	NA	NO	inframe_deletion	ENST00000278616.4:c.2028_2036delCAGTATTGGinsACTG	ENSP00000278616.4:p.Ser677_Gly679delinsLeuAsp	SSIG676-679SLD	PTHR11139,PTHR11139:SF66	18.37	NA	NA	NA	NA	NA	0.00000
ATM	11	108124671	A	G	rs568026188	NO	missense	ENST00000278616.4:c.2029A>G	ENSP00000278616.4:p.Ser677Gly	S677G	PTHR11139,PTHR11139:SF66	0.181	benign	0.002	tolerated	0.13	0	0.00000
ATM	11	108124704	G	A	CD000910,rs769338089	NO	missense	ENST00000278616.4:c.2062G>A	ENSP00000278616.4:p.Glu688Lys	E688K	PTHR11139,PTHR11139:SF66	23.3	benign	0.097	tolerated	0.05	0	0.00006
ATM	11	108124716	C	T	rs765965513	NO	missense	ENST00000278616.4:c.2074C>T	ENSP00000278616.4:p.Arg692Cys	R692C	PTHR11139,PTHR11139:SF66	12.88	benign	0.006	tolerated	0.15	0	0.00003
ATM	11	108124746	C	G	CM0910488	NO	missense	ENST00000278616.4:c.2104C>G	ENSP00000278616.4:p.Leu702Val	L702V	PTHR11139,PTHR11139:SF66	22.6	probably_damaging	0.936	tolerated	0.14	0	0.00000
ATM	11	108124755	T	C	rs757260641,CD961795	NO	missense	ENST00000278616.4:c.2113T>C	ENSP00000278616.4:p.Tyr705His	Y705H	PTHR11139,PTHR11139:SF66	20.7	benign	0.062	tolerated	0.11	0	0.00000
ATM	11	108124757	C	T	NA	NO	synonymous	ENST00000278616.4:c.2115C>T	NA	Y705Y	PTHR11139,PTHR11139:SF66	9.487	NA	NA	NA	NA	NA	0.00000
ATM	11	108124761	T	C	rs4986761,CM013692,COSM41595	NO	missense	ENST00000278616.4:c.2119T>C	ENSP00000278616.4:p.Ser707Pro	S707P	PTHR11139,PTHR11139:SF66	15.81	benign	0.003	tolerated	0.38	0	0.02192
ATM	11	108124767	G	A	NA	YES	splice_donor	ENST00000278616.4:c.2124+1G>A	NA	NA	NA	26.4	NA	NA	NA	NA	NA	0.00000
ATM	11	108124779	A	T	rs199648955,rs35211268	NO	intron	ENST00000278616.4:c.2124+13A>T	NA	NA	NA	4.283	NA	NA	NA	NA	NA	0.00000
ATM	11	108124780	A	G	rs772719886	NO	intron	ENST00000278616.4:c.2124+14A>G	NA	NA	NA	6.863	NA	NA	NA	NA	NA	0.00000
ATM	11	108124801	A	G	NA	NO	intron	ENST00000278616.4:c.2124+35A>G	NA	NA	NA	1.426	NA	NA	NA	NA	NA	0.00000
ATM	11	108124815	G	A	rs376376863	NO	intron	ENST00000278616.4:c.2124+49G>A	NA	NA	NA	2.307	NA	NA	NA	NA	NA	0.00000
ATM	11	108126976	G	A	rs55830714	NO	missense	ENST00000278616.4:c.2159G>A	ENSP00000278616.4:p.Arg720His	R720H	PTHR11139,PTHR11139:SF66	22.8	benign	0.223	tolerated	0.08	0	0.00006
ATM	11	108126992	C	T	rs767380441,rs140110298	NO	synonymous	ENST00000278616.4:c.2175C>T	NA	V725V	PTHR11139,PTHR11139:SF66	15.08	NA	NA	NA	NA	NA	0.00006
ATM	11	108127004	C	T	rs373430058	NO	synonymous	ENST00000278616.4:c.2187C>T	NA	Y729Y	PTHR11139,PTHR11139:SF66	10.98	NA	NA	NA	NA	NA	0.00012
ATM	11	108127010	C	T	rs2229019,COSM758327	NO	synonymous	ENST00000278616.4:c.2193C>T	NA	Y731Y	PTHR11139,PTHR11139:SF66	7.924	NA	NA	NA	NA	NA	0.00039
ATM	11	108127037	A	G	rs56353517	NO	synonymous	ENST00000278616.4:c.2220A>G	NA	A740A	PTHR11139,PTHR11139:SF66	9.523	NA	NA	NA	NA	NA	0.00000
ATM	11	108127039	A	G	NA	NO	missense	ENST00000278616.4:c.2222A>G	ENSP00000278616.4:p.Tyr741Cys	Y741C	PTHR11139,PTHR11139:SF66	0.69	benign	0.001	tolerated	0.34	0	0.00000
ATM	11	108127053	T	C	NA	NO	missense	ENST00000278616.4:c.2236T>C	ENSP00000278616.4:p.Phe746Leu	F746L	PTHR11139,PTHR11139:SF66	25.5	probably_damaging	0.915	tolerated	0.07	0	0.00000
ATM	11	108127057	A	G	COSM1350799,COSM1350798	NO	missense	ENST00000278616.4:c.2240A>G	ENSP00000278616.4:p.Gln747Arg	Q747R	PTHR11139,PTHR11139:SF66	15.74	benign	0.13	tolerated	0.16	0	0.00000
ATM	11	108127067	G	A	CS982070,rs1137887,COSM21643	NO	splice,synonymous	ENST00000278616.4:c.2250G>A	NA	K750	PTHR11139,PTHR11139:SF66	18.21	NA	NA	NA	NA	NA	0.00000
ATM	11	108127077	T	A	NA	NO	intron	ENST00000278616.4:c.2250+10T>A	NA	NA	NA	15.75	NA	NA	NA	NA	NA	0.00000
ATM	11	108128165	T	G	rs765419792	NO	intron	ENST00000278616.4:c.2251-43T>G	NA	NA	NA	18.36	NA	NA	NA	NA	NA	0.00000
ATM	11	108128189	TTC	T	NA	NO	intron	ENST00000278616.4:c.2251-18_2251-17delTC	NA	NA	NA	6.513	NA	NA	NA	NA	NA	0.00000
ATM	11	108128191	C	T	NA	NO	intron	ENST00000278616.4:c.2251-17C>T	NA	NA	NA	4.562	NA	NA	NA	NA	NA	0.00000
ATM	11	108128198	T	G	rs730881346,CS982069	NO	intron	ENST00000278616.4:c.2251-10T>G	NA	NA	NA	4.234	NA	NA	NA	NA	NA	0.00000
ATM	11	108128217	C	A	rs3205809	NO	missense	ENST00000278616.4:c.2260C>A	ENSP00000278616.4:p.Gln754Lys	Q754K	PTHR11139,PTHR11139:SF66	21.4	benign	0.22	tolerated	0.48	0	0.00000
ATM	11	108128222	T	A	NA	YES	stop_gained	ENST00000278616.4:c.2265T>A	ENSP00000278616.4:p.Cys755Ter	C755*	PTHR11139,PTHR11139:SF66	37	NA	NA	NA	NA	NA	0.00000
ATM	11	108128226	G	A	rs587779819	NO	missense	ENST00000278616.4:c.2269G>A	ENSP00000278616.4:p.Gly757Arg	G757R	PTHR11139,PTHR11139:SF66	32	probably_damaging	0.994	deleterious	0.01	15	0.00003
ATM	11	108128238	ACT	A	NA	YES	frameshift,feature_truncation	ENST00000278616.4:c.2284_2285delCT	ENSP00000278616.4:p.Leu762ValfsTer2	T761X	PTHR11139,PTHR11139:SF66	34	NA	NA	NA	NA	NA	0.00000
ATM	11	108128243	G	C	NA	NO	synonymous	ENST00000278616.4:c.2286G>C	NA	L762L	PTHR11139,PTHR11139:SF66	9.774	NA	NA	NA	NA	NA	0.00000
ATM	11	108128246	T	A	rs34231402	NO	missense	ENST00000278616.4:c.2289T>A	ENSP00000278616.4:p.Phe763Leu	F763L	PTHR11139,PTHR11139:SF66	16.81	benign	0.001	tolerated	0.56	0	0.00081
ATM	11	108128290	A	C	rs587779820	NO	missense	ENST00000278616.4:c.2333A>C	ENSP00000278616.4:p.Asn778Thr	N778T	PTHR11139,PTHR11139:SF66	0.008	benign	0.001	tolerated	0.65	0	0.00000
ATM	11	108128319	A	C	CM021943,rs641252	NO	missense	ENST00000278616.4:c.2362A>C	ENSP00000278616.4:p.Ser788Arg	S788R	PTHR11139,PTHR11139:SF66	7.245	benign	0.007	tolerated	0.34	0	0.00006
ATM	11	108128324	C	T	NA	NO	synonymous	ENST00000278616.4:c.2367C>T	NA	N789N	PTHR11139,PTHR11139:SF66	5.835	NA	NA	NA	NA	NA	0.00000
ATM	11	108129706	A	G	NA	NO	splice,intron	ENST00000278616.4:c.2377-7A>G	NA	NA	NA	6.512	NA	NA	NA	NA	NA	0.00000
ATM	11	108129722	A	T	rs201793499	NO	missense	ENST00000278616.4:c.2386A>T	ENSP00000278616.4:p.Asn796Tyr	N796Y	PTHR11139,PTHR11139:SF66	7.75	benign	0.092	tolerated	0.23	0	0.00003
ATM	11	108129732	C	T	rs199954262	NO	missense	ENST00000278616.4:c.2396C>T	ENSP00000278616.4:p.Ala799Val	A799V	PTHR11139,PTHR11139:SF66	1.751	benign	0.002	tolerated	1	0	0.00018
ATM	11	108129735	C	A	NA	NO	missense	ENST00000278616.4:c.2399C>A	ENSP00000278616.4:p.Ser800Tyr	S800Y	PTHR11139,PTHR11139:SF66	26.4	probably_damaging	0.942	deleterious	0	0	0.00000
ATM	11	108129749	C	T	rs780619951,CM960099,COSM1183959,COSN	YES	stop_gained	ENST00000278616.4:c.2413C>T	ENSP00000278616.4:p.Arg805Ter	R805*	PTHR11139,PTHR11139:SF66	35	NA	NA	NA	NA	NA	0.00006
ATM	11	108129754	G	T	rs587781296	NO	missense	ENST00000278616.4:c.2418G>T	ENSP00000278616.4:p.Leu806Phe	L806F	PTHR11139,PTHR11139:SF66	23.2	probably_damaging	0.957	deleterious	0.04	0	0.00003
ATM	11	108129761	T	G	NA	NO	missense	ENST00000278616.4:c.2425T>G	ENSP00000278616.4:p.Ser809Ala	S809A	PTHR11139,PTHR11139:SF66	13.37	benign	0.085	tolerated	0.69	0	0.00000
ATM	11	108129762	C	A	rs730881348	YES	stop_gained	ENST00000278616.4:c.2426C>A	ENSP00000278616.4:p.Ser809Ter	S809*	PTHR11139,PTHR11139:SF66	37	NA	NA	NA	NA	NA	0.00000
ATM	11	108129764	A	G	rs201909756	NO	missense	ENST00000278616.4:c.2428A>G	ENSP00000278616.4:p.Lys810Glu	K810E	PTHR11139,PTHR11139:SF66	23.7	possibly_damaging	0.576	tolerated	0.29	0	0.00000
ATM	11	108129778	C	A	rs3218695	NO	missense	ENST00000278616.4:c.2442C>A	ENSP00000278616.4:p.Asp814Glu	D814E	PTHR11139,PTHR11139:SF66	12.89	benign	0.318	tolerated	0.77	0	0.00021
ATM	11	108129802	A	G	rs747108452	NO	splice,synonymous	ENST00000278616.4:c.2466A>G	NA	L822	PTHR11139,PTHR11139:SF66	5.643	NA	NA	NA	NA	NA	0.00000
ATM	11	108137891	C	T	rs768850329	NO	splice,intron	ENST00000278616.4:c.2467-7C>T	NA	NA	NA	3.994	NA	NA	NA	NA	NA	0.00000
ATM	11	108137907	A	C	rs587782397,COSM132864	NO	missense	ENST00000278616.4:c.2476A>C	ENSP00000278616.4:p.Ile826Leu	I826L	PTHR11139,PTHR11139:SF66	8.262	benign	0.004	tolerated	0.47	0	0.00018
ATM	11	108137909	CA	C	NA	YES	frameshift,feature_truncation	ENST00000278616.4:c.2483delA	ENSP00000278616.4:p.Lys828SerfsTer8	K827X	PTHR11139,PTHR11139:SF66	31	NA	NA	NA	NA	NA	0.00000
ATM	11	108137925	C	T	rs2229022,COSM143902	NO	missense	ENST00000278616.4:c.2494C>T	ENSP00000278616.4:p.Arg832Cys	R832C	PTHR11139,PTHR11139:SF66	17.83	benign	0	tolerated	0.18	0	0.00048
ATM	11	108137956	C	A	NA	NO	missense	ENST00000278616.4:c.2525C>A	ENSP00000278616.4:p.Thr842Asn	T842N	PTHR11139,PTHR11139:SF66	0.023	benign	0.042	tolerated	0.52	0	0.00000
ATM	11	108137962	G	A	rs587781808	NO	missense	ENST00000278616.4:c.2531G>A	ENSP00000278616.4:p.Gly844Glu	G844E	PTHR11139,PTHR11139:SF66	1.412	benign	0	tolerated	1	0	0.00006
ATM	11	108137962	G	C	rs587781808	NO	missense	ENST00000278616.4:c.2531G>C	ENSP00000278616.4:p.Gly844Ala	G844A	PTHR11139,PTHR11139:SF66	0.14	benign	0	tolerated	0.77	0	0.00003
ATM	11	108137963	A	G	rs755261743	NO	synonymous	ENST00000278616.4:c.2532A>G	NA	G844G	PTHR11139,PTHR11139:SF66	6.121	NA	NA	NA	NA	NA	0.00006
ATM	11	108137976	G	T	NA	NO	missense	ENST00000278616.4:c.2545G>T	ENSP00000278616.4:p.Val849Leu	V849L	PTHR11139,PTHR11139:SF66	4.976	benign	0.015	tolerated	0.86	0	0.00000
ATM	11	108137981	G	A	NA	NO	synonymous	ENST00000278616.4:c.2550G>A	NA	E850E	PTHR11139,PTHR11139:SF66	9.826	NA	NA	NA	NA	NA	0.00000
ATM	11	108138003	T	C	CM061641,rs1800056,COSM21826	NO	missense	ENST00000278616.4:c.2572T>C	ENSP00000278616.4:p.Phe858Leu	F858L	PTHR11139,PTHR11139:SF66	8.823	benign	0.011	tolerated	0.1	0	0.02903
ATM	11	108138015	C	T	NA	NO	missense	ENST00000278616.4:c.2584C>T	ENSP00000278616.4:p.Pro862Ser	P862S	PTHR11139,PTHR11139:SF66	0.007	benign	0.001	tolerated	0.75	0	0.00000
ATM	11	108138037	C	T	NA	NO	missense	ENST00000278616.4:c.2606C>T	ENSP00000278616.4:p.Ala869Val	A869V	PTHR11139,PTHR11139:SF66	8.497	benign	0.003	tolerated	0.29	0	0.00003
ATM	11	108138041	C	T	rs587780618,COSM1350811,COSM1350810	NO	synonymous	ENST00000278616.4:c.2610C>T	NA	N870N	PTHR11139,PTHR11139:SF66	11.52	NA	NA	NA	NA	NA	0.00006
ATM	11	108138045	C	T	CM023331,rs3218673	NO	missense	ENST00000278616.4:c.2614C>T	ENSP00000278616.4:p.Pro872Ser	P872S	PTHR11139,PTHR11139:SF66	7.493	benign	0	tolerated	0.81	0	0.00030
ATM	11	108138077	C	T	rs776116433	NO	splice,intron	ENST00000278616.4:c.2638+8C>T	NA	NA	NA	2.036	NA	NA	NA	NA	NA	0.00000

ATM	11	108139107	T	C	rs756418769	NO	intron	ENST00000278616.4:c.2639-30T>C	NA	NA	NA	6.648	NA	NA	NA	NA	NA	0.00000
ATM	11	108139120	G	T	rs2234994	NO	intron	ENST00000278616.4:c.2639-17G>T	NA	NA	NA	6.735	NA	NA	NA	NA	NA	0.00000
ATM	11	108139177	A	G	rs139316519	NO	synonymous	ENST00000278616.4:c.2679A>G	NA	Q893Q	PTHR11139,PTHR11139:SF66	2.565	NA	NA	NA	NA	NA	0.00003
ATM	11	108139183	A	G	rs3218687	NO	synonymous	ENST00000278616.4:c.2685A>G	NA	L895L	Low_complexity_(Seg):seg,PTHR11139,PTHR11139:SF66	1.417	NA	NA	NA	NA	NA	0.00024
ATM	11	108139187	T	A	rs147122522,CD000911	NO	missense	ENST00000278616.4:c.2689T>A	ENSP00000278616.4:p.Phe897Ile	F897I	Low_complexity_(Seg):seg,PTHR11139,PTHR11139:SF66	9.477	benign	0.001	tolerated	0.25	0	0.00009
ATM	11	108139221	T	C	rs754738085	NO	missense	ENST00000278616.4:c.2723T>C	ENSP00000278616.4:p.Val908Ala	V908A	PTHR11139,PTHR11139:SF66	8.54	benign	0.003	tolerated	0.85	0	0.00000
ATM	11	108139236	C	A	NA	NO	missense	ENST00000278616.4:c.2738C>A	ENSP00000278616.4:p.Thr913Asn	T913N	PTHR11139,PTHR11139:SF66	0.086	benign	0.001	tolerated	0.5	0	0.00000
ATM	11	108139302	C	T	rs3218708	NO	missense	ENST00000278616.4:c.2804C>T	ENSP00000278616.4:p.Thr935Met	T935M	PTHR11139,PTHR11139:SF66	8.915	benign	0.003	tolerated	0.12	0	0.00027
ATM	11	108139303	G	C	rs55934812,COSM592	NO	synonymous	ENST00000278616.4:c.2805G>C	NA	T935T	PTHR11139,PTHR11139:SF66	0.701	NA	NA	NA	NA	NA	0.00093
ATM	11	108139306	A	G	rs547791214	NO	synonymous	ENST00000278616.4:c.2808A>G	NA	L936L	PTHR11139,PTHR11139:SF66	0.06	NA	NA	NA	NA	NA	0.00000
ATM	11	108139322	C	T	rs3218688,COSM168656	NO	missense	ENST00000278616.4:c.2824C>T	ENSP00000278616.4:p.Leu942Phe	L942F	PTHR11139,PTHR11139:SF66	23.2	possibly_damaging	0.847	deleterious	0.02	0	0.00000
ATM	11	108139345	C	G	rs370160823,COSM237019	NO	intron	ENST00000278616.4:c.2838+9C>G	NA	NA	NA	3.099	NA	NA	NA	NA	NA	0.00000
ATM	11	108139346	G	A	NA	NO	intron	ENST00000278616.4:c.2838+10G>A	NA	NA	NA	0.566	NA	NA	NA	NA	NA	0.00000
ATM	11	108139364	T	TA	rs2234995	NO	intron	ENST00000278616.4:c.2838+28_2838+29insA	NA	NA	NA	3.857	NA	NA	NA	NA	NA	0.00000
ATM	11	108139376	G	C	rs745417415	NO	intron	ENST00000278616.4:c.2838+40G>C	NA	NA	NA	3.873	NA	NA	NA	NA	NA	0.00000
ATM	11	108139382	G	T	rs374343554	NO	intron	ENST00000278616.4:c.2838+46G>T	NA	NA	NA	6.19	NA	NA	NA	NA	NA	0.00000
ATM	11	108139396	A	G	NA	NO	intron	ENST00000278616.4:c.2838+60A>G	NA	NA	NA	9.165	NA	NA	NA	NA	NA	0.00000
ATM	11	108139397	T	A	rs45454103	NO	intron	ENST00000278616.4:c.2838+61T>A	NA	NA	NA	10.64	NA	NA	NA	NA	NA	0.00000
ATM	11	108139405	C	CA	NA	NO	intron	ENST00000278616.4:c.2838+70dupA	NA	NA	NA	5.538	NA	NA	NA	NA	NA	0.00000
ATM	11	108141775	C	T	rs761953913	NO	intron	ENST00000278616.4:c.2839-16C>T	NA	NA	NA	3.354	NA	NA	NA	NA	NA	0.00000
ATM	11	108141816	C	A	COSM1492405,COSM1492404	NO	missense	ENST00000278616.4:c.2864C>A	ENSP00000278616.4:p.Pro955His	P955H	PTHR11139,PTHR11139:SF66	29.4	probably_damaging	0.986	deleterious	0.01	0	0.00000
ATM	11	108141841	G	A	rs35098825	NO	missense	ENST00000278616.4:c.2889G>A	ENSP00000278616.4:p.Met963Ile	M963I	PTHR11139,PTHR11139:SF66	16.68	benign	0.001	tolerated	0.18	0	0.00000
ATM	11	108141871	A	G	rs587779829	NO	splice,synonymous	ENST00000278616.4:c.2919A>G	NA	L973	Superfamily_domains:SSF48371,PTHR11139,PTHR11139:SF66	12.03	NA	NA	NA	NA	NA	0.00000
ATM	11	108141889	C	A	rs558376224	NO	intron	ENST00000278616.4:c.2921+16C>A	NA	NA	NA	12.84	NA	NA	NA	NA	NA	0.00000
ATM	11	108141891	T	TA	rs575967175	NO	intron,feature_elongation	ENST00000278616.4:c.2921+19dupA	NA	NA	NA	10.58	NA	NA	NA	NA	NA	0.00000
ATM	11	108141894	G	GT	rs753113491	NO	intron,feature_elongation	ENST00000278616.4:c.2921+23dupT	NA	NA	NA	7.419	NA	NA	NA	NA	NA	0.00000
ATM	11	108141897	A	G	rs767051555	NO	intron	ENST00000278616.4:c.2921+24A>G	NA	NA	NA	4.58	NA	NA	NA	NA	NA	0.00000
ATM	11	108141899	G	A	NA	NO	intron	ENST00000278616.4:c.2921+26G>A	NA	NA	NA	0.262	NA	NA	NA	NA	NA	0.00000
ATM	11	108141913	T	A	NA	NO	intron	ENST00000278616.4:c.2921+40T>A	NA	NA	NA	4.231	NA	NA	NA	NA	NA	0.00000
ATM	11	108141924	TG	T	NA	NO	intron,feature_truncation	ENST00000278616.4:c.2921+52delG	NA	NA	NA	9.711	NA	NA	NA	NA	NA	0.00000
ATM	11	108141929	T	G	NA	NO	intron	ENST00000278616.4:c.2922-49T>G	NA	NA	NA	1.953	NA	NA	NA	NA	NA	0.00000
ATM	11	108141937	T	A	rs749535458	NO	intron	ENST00000278616.4:c.2922-41T>A	NA	NA	NA	3.092	NA	NA	NA	NA	NA	0.00000
ATM	11	108141941	C	A	NA	NO	intron	ENST00000278616.4:c.2922-37C>A	NA	NA	NA	4.429	NA	NA	NA	NA	NA	0.00000
ATM	11	108141943	T	G	rs771026892	NO	intron	ENST00000278616.4:c.2922-35T>G	NA	NA	NA	1.669	NA	NA	NA	NA	NA	0.00000
ATM	11	108141980	A	G	rs730881354,CM0910495	NO	missense,splice	ENST00000278616.4:c.2924A>G	ENSP00000278616.4:p.Asn975Ser	N975S	Superfamily_domains:SSF48371,PTHR11139,PTHR11139:SF66	8.852	benign	0.001	tolerated	0.42	0	0.00000
ATM	11	108141988	T	C	rs139552233,COSM922715,COSM21828,COS	NO	missense	ENST00000278616.4:c.2932T>C	ENSP00000278616.4:p.Ser978Pro	S978P	Superfamily_domains:SSF48371,PTHR11139,PTHR11139:SF66	26.8	possibly_damaging	0.797	deleterious	0.01	65	0.00147
ATM	11	108141990	T	G	rs368678134,COSM1350813,COSM1350812	NO	synonymous	ENST00000278616.4:c.2934T>G	NA	S978S	Superfamily_domains:SSF48371,PTHR11139,PTHR11139:SF66	1.101	NA	NA	NA	NA	NA	0.00015
ATM	11	108141995	A	T	NA	NO	missense	ENST00000278616.4:c.2939A>T	ENSP00000278616.4:p.Tyr980Phe	Y980F	Superfamily_domains:SSF48371,PTHR11139,PTHR11139:SF66	24	possibly_damaging	0.501	tolerated	0.08	0	0.00000
ATM	11	108141998	G	A	rs755896387	NO	missense	ENST00000278616.4:c.2942G>A	ENSP00000278616.4:p.Arg981His	R981H	Superfamily_domains:SSF48371,PTHR11139,PTHR11139:SF66	23.2	benign	0.017	tolerated	0.07	25	0.00000
ATM	11	108142000	C	T	rs587779830,COSM1297603,COSM1297602	NO	missense	ENST00000278616.4:c.2944C>T	ENSP00000278616.4:p.Arg982Cys	R982C	Superfamily_domains:SSF48371,PTHR11139,PTHR11139:SF66	34	possibly_damaging	0.893	deleterious	0	35	0.00006
ATM	11	108142010	A	T	NA	NO	missense	ENST00000278616.4:c.2954A>T	ENSP00000278616.4:p.Asp985Val	D985V	Superfamily_domains:SSF48371,PTHR11139,PTHR11139:SF66	23.4	benign	0.273	deleterious	0.01	0	0.00000
ATM	11	108142070	A	G	rs146531614	NO	missense	ENST00000278616.4:c.3014A>G	ENSP00000278616.4:p.Asn1005Ser	N1005S	Superfamily_domains:SSF48371,PTHR11139,PTHR11139:SF66	0.001	benign	0.001	tolerated	0.97	0	0.00078
ATM	11	108142072	A	G	rs139893395	NO	missense	ENST00000278616.4:c.3016A>G	ENSP00000278616.4:p.Met1006Val	M1006V	Superfamily_domains:SSF48371,PTHR11139,PTHR11139:SF66	0.001	benign	0.001	tolerated	0.62	0	0.00018
ATM	11	108142074	G	T	NA	NO	missense	ENST00000278616.4:c.3018G>T	ENSP00000278616.4:p.Met1006Ile	M1006I	Superfamily_domains:SSF48371,PTHR11139,PTHR11139:SF66	0.105	benign	0.004	tolerated	0.46	0	0.00000
ATM	11	108142107	G	T	NA	NO	missense	ENST00000278616.4:c.3051G>T	ENSP00000278616.4:p.Gln1017His	Q1017H	Superfamily_domains:SSF48371,PTHR11139,PTHR11139:SF66	11.5	benign	0.008	tolerated	0.33	0	0.00000
ATM	11	108142141	C	G	NA	NO	splice,intron	ENST00000278616.4:c.3077+8C>G	NA	NA	NA	4.917	NA	NA	NA	NA	NA	0.00000
ATM	11	108142158	C	T	rs773984258	NO	intron	ENST00000278616.4:c.3077+25C>T	NA	NA	NA	3.553	NA	NA	NA	NA	NA	0.00000
ATM	11	108142160	A	G	rs200922643	NO	intron	ENST00000278616.4:c.3077+27A>G	NA	NA	NA	3.704	NA	NA	NA	NA	NA	0.00000
ATM	11	108143182	C	T	rs664677	NO	intron	ENST00000278616.4:c.3078-77C>T	NA	NA	NA	4.058	NA	NA	NA	NA	NA	0.00000
ATM	11	108143184	A	G	NA	NO	intron	ENST00000278616.4:c.3078-75A>G	NA	NA	NA	4.808	NA	NA	NA	NA	NA	0.00000
ATM	11	108143237	T	C	NA	NO	intron	ENST00000278616.4:c.3078-22T>C	NA	NA	NA	1.06	NA	NA	NA	NA	NA	0.00000
ATM	11	108143258	G	A	rs750663117,CS992442	YES	splice_acceptor	ENST00000278616.4:c.3078-1G>A	NA	NA	NA	26.4	NA	NA	NA	NA	NA	0.00003
ATM	11	108143261	A	G	NA	NO	missense,splice	ENST00000278616.4:c.3080A>G	ENSP00000278616.4:p.His1027Arg	H1027R	Superfamily_domains:SSF48371,PTHR11139,PTHR11139:SF66	12.5	benign	0.003	tolerated	0.35	0	0.00000
ATM	11	108143263	C	A	NA	NO	missense	ENST00000278616.4:c.3082C>A	ENSP00000278616.4:p.Leu1028Ile	L1028I	Superfamily_domains:SSF48371,PTHR11139,PTHR11139:SF66	23.7	possibly_damaging	0.566	tolerated	0.05	0	0.00000
ATM	11	108143266	AC	A	NA	YES	frameshift,feature_truncation	ENST00000278616.4:c.3086delC	ENSP00000278616.4:p.Thr1029LysfsTer10	T1029X	Superfamily_domains:SSF48371,PTHR11139,PTHR11139:SF66	35	NA	NA	NA	NA	NA	0.00000
ATM	11	108143293	G	A	NA	NO	missense	ENST00000278616.4:c.3112G>A	ENSP00000278616.4:p.Val1038Ile	V1038I	Superfamily_domains:SSF48371,PTHR11139,PTHR11139:SF66	26.9	probably_damaging	0.94	deleterious	0	25	0.00000
ATM	11	108143295	A	T	rs374451781	NO	synonymous	ENST00000278616.4:c.3114A>T	NA	V1038V	Superfamily_domains:SSF48371,PTHR11139,PTHR11139:SF66	1.385	NA	NA	NA	NA	NA	0.00000
ATM	11	108143299	A	G	rs3092857,COSM22486	NO	missense	ENST00000278616.4:c.3118A>G	ENSP00000278616.4:p.Met1040Val	M1040V	Superfamily_domains:SSF48371,PTHR11139,PTHR11139:SF66	0.019	benign	0.001	tolerated	1	0	0.00003
ATM	11	108143313	T	C	NA	NO	synonymous	ENST00000278616.4:c.3132T>C	NA	N1044N	Superfamily_domains:SSF48371,PTHR11139,PTHR11139:SF66	0.158	NA	NA	NA	NA	NA	0.00000
ATM	11	108143331	T	C	rs3092859	NO	synonymous	ENST00000278616.4:c.3150T>C	NA	L1050L	Superfamily_domains:SSF48371,PTHR11139,PTHR11139:SF66	4.799	NA	NA	NA	NA	NA	0.00105
ATM	11	108143367	A	G	NA	NO	intron	ENST00000278616.4:c.3153+33A>G	NA	NA	NA	4.394	NA	NA	NA	NA	NA	0.00000
ATM	11	108143392	T	A	NA	NO	intron	ENST00000278616.4:c.3154-57T>A	NA	NA	NA	11.69	NA	NA	NA	NA	NA	0.00000
ATM	11	108143422	T	C	rs758643104	NO	intron	ENST00000278616.4:c.3154-27T>C	NA	NA	NA	5.864	NA	NA	NA	NA	NA	0.00000
ATM	11	108143437	C	A	rs781387283	NO	intron	ENST00000278616.4:c.3154-12C>A	NA	NA	NA	8.895	NA	NA	NA	NA	NA	0.00000
ATM	11	108143444	C	T	rs55719759	NO	splice,intron	ENST00000278616.4:c.3154-5C>T	NA	NA	NA	8.171	NA	NA	NA	NA	NA	0.00000
ATM	11	108143445	G	A	rs199543313	NO	splice,intron	ENST00000278616.4:c.3154-4G>A	NA	NA	NA	5.041	NA	NA	NA	NA	NA	0.00000
ATM	11	108143450	C	T	NA	NO	missense,splice	ENST00000278616.4:c.3155C>T	ENSP00000278616.4:p.Ala1052Val	A1052V	Superfamily_domains:SSF48371,PTHR11139,PTHR11139:SF66	4.126	benign	0.007	tolerated	1	0	0.00000
ATM	11	108143456	C	G	CM973365,rs1800057,COSM21827	NO	missense	ENST00000278616.4:c.3161C>G	ENSP00000278616.4:p.Pro1054Arg	P1054R	Superfamily_domains:SSF48371,PTHR11139,PTHR11139:SF66	26.5	probably_damaging	0.982	deleterious	0	65	0.05150
ATM	11	108143470	G	A	rs370282831	NO	missense	ENST00000278616.4:c.3175G>A	ENSP00000278616.4:p.Ala1059Thr	A1059T	Superfamily_domains:SSF48371,PTHR11139,PTHR11139:SF66	16.44	benign	0.082	tolerated	0.28	0	0.00000
ATM	11	108143486	T	C	NA	NO	missense	ENST00000278616.4:c.3191T>C	ENSP00000278616.4:p.Met1064Thr	M1064T	Superfamily_domains:SSF48371,PTHR11139,PTHR11139:SF66	0.044	benign	0.028	tolerated	0.16	0	0.00000
ATM	11	108143514	A	G	CD052443,rs752849892	NO	synonymous	ENST00000278616.4:c.3219A>G	NA	V1073V	Superfamily_domains:SSF48371,PTHR11139,PTHR11139:SF66	10.47	NA	NA	NA	NA	NA	0.00003
ATM	11	108143537	A	G	rs368111672,COSM3720571,COSM3720570	NO	missense	ENST00000278616.4:c.3242A>G	ENSP00000278616.4:p.Asn1081Ser	N1081S	Superfamily_domains:SSF48371,PTHR11139,PTHR11139:SF66	0.037	benign	0.001	tolerated	1	0	0.00006
ATM	11	108143540	CATC	CTGAT	NA	YES	frameshift,feature_elongation	ENST00000278616.4:c.3246_3248delTCAinsTGAT	ENSP00000278616.4:p.His1083AspfsTer13	QS1082-1083HDX	Superfamily_domains:SSF48371,PTHR11139,PTHR11139:SF66	35	NA	NA	NA	NA	NA	0.00000
ATM	11	108143560	G	T	rs730881358	NO	missense	ENST00000278616.4:c.3265G>T	ENSP00000278616.4:p.Ala1089Ser	A1089S	Superfamily_domains:SSF48371,PTHR11139,PTHR11139:SF66	18	benign	0.035	tolerated	0.21	0	0.00000
ATM	11	108143601	A	C	rs763939403	NO	intron	ENST00000278616.4:c.3284+22A>C	NA	NA	NA	4.479	NA	NA	NA	NA	NA	0.00000
ATM	11	108150188	T	A	rs370294782	NO	intron	ENST00000278616.4:c.3285-30T>A	NA	NA	NA	6.498	NA	NA	NA	NA	NA	0.00000
ATM	11	108150198	G	A	NA	NO	intron	ENST00000278616.4:c.3285-20G>A	NA	NA	NA	0.153	NA	NA	NA	NA	NA	0.00000
ATM	11	108150207	CT	C	rs1799757	NO	intron,feature_truncation	ENST00000278616.4:c.3285-9delT	NA	NA	NA	7.431	NA	NA	NA	NA	NA	0.00000
ATM	11	108150225	C	A	rs773964990	NO	missense	ENST00000278616.4:c.3292C>A	ENSP00000278616.4:p.Gln1098Lys	Q1098K	Superfamily_domains:SSF48371,PTHR11139,PTHR11139:SF66	10.53	benign	0.028	tolerated	1	0	0.00000
ATM	11	108150228	G	T	rs372966951,CM0910497	NO	missense	ENST00000278616.4:c.3295G>T	ENSP00000278616.4:p.Asp1099Tyr	D1099Y	Superfamily_domains:SSF48371,PTHR11139,PTHR11139:SF66	21	benign	0.011	deleterious	0.01	0	0.00000
ATM	11	108150233	G	A	rs587780621	NO	synonymous	ENST00000278616.4:c.3300G>A	NA	T1100T	Superfamily_domains:SSF48371,PTHR11139,PTHR11139:SF66	6.657	NA	NA	NA	NA	NA	0.00008
ATM	11	108150237	G	A	rs147557621	NO	missense	ENST00000278616.4:c.3304G>A	ENSP00000278616.4:p.Gly1102Arg	G1102R	Superfamily_domains:SSF48371,PTHR11139,PTHR11139:SF66	0.227	benign	0.001	tolerated	0.55	0	0.00004
ATM	11	108150261	G	A	rs147112946	NO	missense	ENST00000278616.4:c.3328G>A	ENSP00000278616.4:p.Ala1110Thr	A1110T	PTHR11139,PTHR11139:SF66	21.5	benign	0.097	tolerated	0.21	0	0.00007
ATM	11	108150275	G	A	rs138393322	NO	synonymous	ENST00000278616.4:c.3342G>A	NA	K1114K	PTHR11139,PTHR11139:SF66	9.436	NA	NA	NA	NA	NA	0.00167
ATM	11	108150285	A	G	rs572564322	NO	missense	ENST00000278616.4:c.3352A>G	ENSP00000278616.4:p.Thr1118Ala	T1118A	PTHR11139,PTHR11139:SF66	11.56	benign	0.001	tolerated	0.36	0	0.00000
ATM	11	108150287	A	G	rs377316982	NO	synonymous	ENST00000278616.4:c.3354A>G	NA	T1118T	PTHR11139,PTHR11139:SF66	2.459	NA	NA	NA	NA	NA	0.00006
ATM	11	108150302	A	G	rs587780543,rs587781752	NO	synonymous	ENST00000278616.4:c.3369A>G	NA	A1123A	PTHR11139,PTHR11139:SF66	10.21	NA	NA	NA	NA	NA	0.00003
ATM	11	108150304	A	T	NA	NO	missense	ENST00000278616.4:c.3371A>T	ENSP00000278616.4:p.Tyr1124Phe	Y1124F	PTHR11139,PTHR11139:SF66	20.5	benign	0.097	deleterious	0.03	15	0.00000
ATM	11	108150311	A	G	rs149182949	NO	synonymous	ENST00000278616.4:c.3378A>G	NA	K1126K	PTHR11139,PTHR11139:SF66	3.043	NA	NA	NA	NA	NA	0.00018
ATM	11	108150316	A	G	CM023332,rs2229020,COSM21936,COSM135	NO	missense	ENST00000278616.4:c.3383A>G	ENSP00000278616.4:p.Gln1128Arg	Q1128R	PTHR11139,PTHR11139:SF66	21.9	benign	0.005	deleterious	0.02	0	0.00009
ATM	11	108150364	TATC	T	rs545376366	NO	intron,feature_truncation	ENST00000278616.4:c.3402+32_3402+34delCAT	NA	NA	NA	7.454	NA	NA	NA	NA	NA	0.00000
ATM	11	108150372	G	A	rs200168792	NO	intron	ENST00000278616.4:c.3402+37G>A	NA	NA	NA	4.275	NA	NA	NA	NA	NA	0.00000
ATM	11	108150374	T	C	rs373402943	NO	intron	ENST00000278616.4:c.3402+39T>C	NA	NA	NA	5.233	NA	NA	NA	NA	NA	0.00000
ATM	11	108151674	T	TA	NA	NO	intron,feature_elongation	ENST00000278616.4:c.3403-45dupA	NA	NA	NA	1.829	NA	NA	NA	NA	NA	0.00000
ATM	11	108151688	T	A	rs148368017	NO	intron	ENST00000278616.4:c.3403-34T>A	NA	NA	NA	7.549	NA	NA	NA	NA	NA	0.00000
ATM	11	108151706	T	TA	rs569564716	NO	intron,feature_elongation	ENST00000278616.4:c.3403-16_3403-15insA	NA	NA	NA	0.011	NA	NA	NA	NA	NA	0.00000
ATM	11	108151726	A	G	rs768490475	NO	missense	ENST00000278616.4:c.3407A>G	ENSP00000278616.4:p.His1136Arg	H1136R	PTHR11139,PTHR11139:SF66	0.003	benign	0.001	tolerated	0.59	0	0.00000
ATM	11	108151740	C	A	NA	NO	missense	ENST00000278616.4:c.3421C>A	ENSP00000278616.4:p.Pro1141Thr	P1141T	PTHR11139,PTHR11139:SF66	0.548	benign	0.003	tolerated	0.17	0	0.00000
ATM	11	108151786	C	T	rs759951393,CM0910499	NO	missense	ENST00000278616.4:c.3467C>T	ENSP00000278616.4:p.Thr1156Met	T1156M	PTHR11139,PTHR11139:SF66	15.37	benign	0.002	tolerated	0.18	0	0.00006
ATM	11	108151787	G	A	rs148358896	NO	synonymous	ENST00000278616.4:c.3468G>A	NA	T1156T	PTHR11139,PTHR11139:SF66	14.59	NA	NA	NA	NA	NA	0.00012
ATM	11	108151797	G	C	rs567344545	NO	missense	ENST00000278616.4:c.3478G>C	ENSP00000278616.4:p.Val1160Leu	V1160L	PTHR11139,PTHR11139:SF66	17.79	benign	0.022	tolerated	0.13	0	0.00003
ATM	11	108151836	T	C	rs141460670	NO	synonymous	ENST00000278616.4:c.3517T>C	NA	L1173L	PTHR11139,PTHR11139:SF66	2.645	NA	NA	NA	NA	NA	0.00006
ATM	11	108151840	T	C	NA	NO	missense	ENST00000278616.4:c.3521T>C	ENSP00000278616.4:p.Phe1174Ser	F1174S	PTHR11139,PTHR11139:SF66	29.4	probably_damaging	0.991	deleterious	0	65	0.00000
ATM	11	108153392	A	T	NA	NO	intron	ENST00000278616.4:c.3577-45A>T	NA	NA	NA	6.103	NA	NA	NA	NA	NA	0.00000
ATM	11	108153409	T	G	rs373116929	NO	intron	ENST00000278616.4:c.3577-28T>G	NA	NA	NA	4.884	NA	NA	NA	NA	NA	0.00000
ATM	11	108153420	C	T	NA	NO	intron	ENST00000278616.4:c.3577-17C>T	NA	NA	NA	1.359	NA	NA	NA	NA	NA	0.00000
ATM	11	108153431	G	A	rs56006345	NO	splice,intron	ENST00000278616.4:c.3577-6G>A	NA	NA	NA	0.101	NA	NA	NA	NA	NA	0.00000
ATM	11	108153448	A	G	rs376524625	NO	synonymous	ENST00000278616.4:c.3588A>G	NA	K1196K	PTHR11139,PTHR11139:SF66	0.009	NA	NA	NA	NA	NA	0.00009
ATM	11	108153466	A	T	NA	NO	synonymous	ENST00000278616.4:c.3606A>T	NA	G1202G	PTHR11139,PTHR11139:SF66	0.102	NA	NA	NA	NA	NA	0.00000
ATM	11	108153474	G	A	rs769106895	NO	missense	ENST00000278616.4:c.3614G>A	ENSP00000278616.4:p.Arg1205His	R1205H	PTHR11139,PTHR11139:SF66	7.767	benign	0.001	tolerated	0.26	0	0.00000
ATM	11	108153489	T	C	rs192572042	NO	missense	ENST00000278616.4:c.3629T>C	ENSP00000278616.4:p.Met1210Thr	M1210T	PTHR11139,PTHR11139:SF66	23.1	benign	0.131	deleterious	0.02	0	0.00000
ATM	11	108153490	G	A	rs587778073,CM023333	NO	missense	ENST00000278616.4:c.3630G>A	ENSP00000278616.4:p.Met1210Ile	M1210I	PTHR11139,PTHR11139:SF66	21.7	benign	0.05	tolerated	0.16	0	0.00006
ATM	11	108153503	G	C	NA	NO	missense	ENST00000278616.4:c.3643G>C	ENSP00000278616.4:p.Asp1215His	D1215H	PTHR11139,PTHR11139:SF66	24.3	probably_damaging	0.921	tolerated	0.19	0	0.00000
ATM	11	108153549	A	G	rs587782195	NO	missense	ENST00000278616.4:c.3689A>G	ENSP00000278616.4:p.Asn1230Ser	N1230S	PTHR11139,PTHR11139:SF66	0.528	benign	0	tolerated	1	0	0.00000
ATM	11	108153623	C	T	NA	NO	intron	ENST00000278616.4:c.3746+17C>T	NA	NA	NA	2.428	NA	NA	NA	NA	NA	0.00000
ATM	11	108153654	T	C	NA	NO	intron	ENST00000278616.4:c.3746+48T>C	NA	NA	NA	4.34	NA	NA	NA	NA	NA	0.00000
ATM	11	108155000	T	G	rs371526361	NO	missense	ENST00000278616.4:c.3793T>G	ENSP00000278616.4:p.Phe1265Val	F1265V	Superfamily_domains:SSF48371,PTHR11139,PTHR11139:SF66	24.1	possibly_damaging	0.49	tolerated	0.07	0	0.00000
ATM	11	108155007	AG	A	rs765158119	YES	frameshift,feature_truncation	ENST00000278616.4:c.3802delG	ENSP00000278616.4:p.Val1268Ter	E1267X	Superfamily_domains:SSF48371,PTHR11139,PTHR11139:SF66	28.3	NA	NA	NA	NA	NA	0.00009
ATM	11	108155013	A	G	rs146017595	NO	missense	ENST00000278616.4:c.3806A>G	ENSP00000278616.4:p.Lys1269Arg	K1269R	Superfamily_domains:SSF48371,PTHR11139,PTHR11139:SF66	22	benign	0.033	tolerated	0.06	25	0.00003
ATM	11	108155051	C	A	NA	NO	missense	ENST00000278616.4:c.3844C>A	ENSP00000278616.4:p.Leu1282Ile	L1282I	Superfamily_domains:SSF48371,PTHR11139,PTHR11139:SF66	24.3	benign	0.391	deleterious	0.02	0	0.00000
ATM	11	108155090	C	T	NA	NO	missense	ENST00000278616.4:c.3883C>T	ENSP00000278616.4:p.Leu1295Phe	L1295F	Superfamily_domains:SSF48371,PTHR11139,PTHR11139:SF66	30	probably_damaging	0.997	deleterious	0	15	0.00000
ATM	11	108155132	G	A	rs149711770,COSM22507	NO	missense	ENST00000278616.4:c.3925G>A	ENSP00000278616.4:p.Ala1309Thr	A1309T	Superfamily_domains:SSF48371,PTHR11139,PTHR11139:SF66	20.6	benign	0.027	tolerated	0.08	0	0.00183
ATM	11	108155168	A	G	rs730881366	NO	missense	ENST00000278616.4:c.3961A>G	ENSP00000278616.4:p.Met1321Val	M1321V	Superfamily_domains:SSF48371,PTHR11139,PTHR11139:SF66	7.022	benign	0.001	tolerated	0.6	0	0.00000
ATM	11	108155200	G	C	COSM309331,COSM1350849,COSM1350848	NO	missense,splice	ENST00000278616.4:c.3993G>C	ENSP00000278616.4:p.Gln1331His	Q1331H	Superfamily_domains:SSF48371,PTHR11139,PTHR11139:SF66	25.8	possibly_damaging	0.806	deleterious	0.01	0	0.00000
ATM	11	108155205	G	T	CS023467,rs3092842	NO	splice,intron	ENST00000278616.4:c.3993+5G>T	NA	NA	NA	0.154	NA	NA	NA	NA	NA	0.00000
ATM	11	108155238	A	G	NA	NO	intron	ENST00000278616.4:c.3993+38A>G	NA	NA	NA	2.868	NA	NA	NA	NA	NA	0.00000
ATM	11	108155240	G	A	rs3218709	NO	intron	ENST00000278616.4:c.3993+40G>A	NA	NA	NA	5.965	NA	NA	NA	NA	NA	0.00000
ATM	11	108155248	A	G	rs3218712	NO	intron	ENST00000278616.4:c.3993+48A>G	NA	NA	NA	1.71	NA	NA	NA	NA	NA	0.00000
ATM	11	108155251	T	C	rs767641380	NO	intron	ENST00000278616.4:c.3993+51T>C	NA	NA	NA	3.254	NA	NA	NA	NA	NA	0.00000
ATM	11	108155275	T	C	NA	NO	intron	ENST00000278616.4:c.3993+75T>C	NA	NA	NA	1.067	NA	NA	NA	NA	NA	0.00000
ATM	11	108155279	C	T	rs541218835	NO	intron	ENST00000278616.4:c.3993+79C>T	NA	NA	NA	0.859	NA	NA	NA	NA	NA	0.00000
ATM	11	108155280	G	A	rs557974976	NO	intron	ENST00000278616.4:c.3993+80G>A	NA	NA	NA	0.797	NA	NA	NA	NA	NA	0.00000
ATM	11	108155289	G	A	NA	NO	intron	ENST00000278616.4:c.3993+89G>A	NA	NA	NA	0.568	NA	NA	NA	NA	NA	0.00000
ATM	11	108155297	A	G	NA	NO	intron	ENST00000278616.4:c.3993+97A>G	NA	NA	NA	3.063	NA	NA	NA	NA	NA	0.00000
ATM	11	108158277	C	T	rs753142634	NO	intron	ENST00000278616.4:c.3994-50C>T	NA	NA	NA	0.601	NA	NA	NA	NA	NA	0.00000
ATM	11	108158288	A	G	rs778072373	NO	intron	ENST00000278616.4:c.3994-39A>G	NA	NA	NA	4.549	NA	NA	NA	NA	NA	0.00000
ATM	11	108158289	T	C	NA	NO	intron	ENST00000278616.4:c.3994-38T>C	NA	NA	NA	3.229	NA	NA	NA	NA	NA	0.00000
ATM	11	108158320	C	T	rs769346400	NO	splice,intron	ENST00000278616.4:c.3994-7C>T	NA	NA	NA	12.49	NA	NA	NA	NA	NA	0.00000
ATM	11	108158375	T	C	rs56355831	NO	synonymous	ENST00000278616.4:c.4042T>C	NA	L1348L	Superfamily_domains:SSF48371,PTHR11139,PTHR11139:SF66	2.599	NA	NA	NA	NA	NA	0.00006
ATM	11	108158393	C	A	rs145119475	NO	missense	ENST00000278616.4:c.4060C>A	ENSP00000278616.4:p.Pro1354Thr	P1354T	Superfamily_domains:SSF48371,PTHR11139,PTHR11139:SF66	14.83	benign	0.003	tolerated	0.48	0	0.00075
ATM	11	108158399	A	G	CM035634,rs147600485	NO	missense	ENST00000278616.4:c.4066A>G	ENSP00000278616.4:p.Asn1356Asp	N1356D	Superfamily_domains:SSF48371,PTHR11139,PTHR11139:SF66	0.024	benign	0.001	tolerated	0.93	0	0.00048
ATM	11	108158400	A	T	NA	NO	missense	ENST00000278616.4:c.4067A>T	ENSP00000278616.4:p.Asn1356Ile	N1356I	Superfamily_domains:SSF48371,PTHR11139,PTHR11139:SF66	17.7	benign	0.012	deleterious	0.04	0	0.00000
ATM	11	108158404	T	C	rs767516955	NO	synonymous	ENST00000278616.4:c.4071T>C	NA	S1357S	Superfamily_domains:SSF48371,PTHR11139,PTHR11139:SF66	4.227	NA	NA	NA	NA	NA	0.00006
ATM	11	108158448	T	C	rs368606937	NO	splice,intron	ENST00000278616.4:c.4109+6T>C	NA	NA	NA	9.079	NA	NA	NA	NA	NA	0.00000
ATM	11	108159667	A	T	rs778523394	NO	intron	ENST00000278616.4:c.4110-37A>T	NA	NA	NA	10.16	NA	NA	NA	NA	NA	0.00000
ATM	11	108159706	A	G	NA	NO	missense,splice	ENST00000278616.4:c.4112A>G	ENSP00000278616.4:p.Asp1371Gly	D1371G	Superfamily_domains:SSF48371,PTHR11139,PTHR11139:SF66	22.2	benign	0.101	tolerated	0.07	0	0.00000
ATM	11	108159732	C	T	CM021944,rs3092856,COSM24627	NO	missense	ENST00000278616.4:c.4138C>T	ENSP00000278616.4:p.His1380Tyr	H1380Y	Superfamily_domains:SSF48371,PTHR11139,PTHR11139:SF66	6.964	benign	0.001	tolerated	1	0	0.00123
ATM	11	108159742	C	T	rs141087784,CM014033	NO	missense	ENST00000278616.4:c.4148C>T	ENSP00000278616.4:p.Ser1383Leu	S1383L	Superfamily_domains:SSF48371,PTHR11139,PTHR11139:SF66	26.5	benign	0.11	deleterious	0	65	0.00012
ATM	11	108159743	G	A	rs749180334	NO	synonymous	ENST00000278616.4:c.4149G>A	NA	S1383S	Superfamily_domains:SSF48371,PTHR11139,PTHR11139:SF66	11.25	NA	NA	NA	NA	NA	0.00003
ATM	11	108159761	A	G	rs183214437	NO	synonymous	ENST00000278616.4:c.4167A>G	NA	T1389T	Superfamily_domains:SSF48371,PTHR11139,PTHR11139:SF66	5.974	NA	NA	NA	NA	NA	0.00018
ATM	11	108159790	C	A	NA	NO	missense	ENST00000278616.4:c.4196C>A	ENSP00000278616.4:p.Thr1399Asn	T1399N	Superfamily_domains:SSF48371,PTHR11139,PTHR11139:SF66	23.8	possibly_damaging	0.558	tolerated	0.11	0	0.00000
ATM	11	108160310	A	C	rs753311330	NO	intron	ENST00000278616.4:c.4237-19A>C	NA	NA	NA	2.029	NA	NA	NA	NA	NA	0.00000
ATM	11	108160339	A	G	rs758180727	NO	missense	ENST00000278616.4:c.4247A>G	ENSP00000278616.4:p.Gln1416Arg	Q1416R	Superfamily_domains:SSF48371,PTHR11139,PTHR11139:SF66	24.7	possibly_damaging	0.864	deleterious	0	35	0.00003
ATM	11	108160350	C	T	CM000653,rs1800058	NO	missense	ENST00000278616.4:c.4258C>T	ENSP00000278616.4:p.Leu1420Phe	L1420F	Superfamily_domains:SSF48371,PTHR11139,PTHR11139:SF66	15.68	benign	0.102	tolerated	0.07	0	0.03759
ATM	11	108160396	A	C	rs769980220	NO	missense	ENST00000278616.4:c.4304A>C	ENSP00000278616.4:p.Lys1435Thr	K1435T	Superfamily_domains:SSF48371,PTHR11139,PTHR11139:SF66	24.9	possibly_damaging	0.611	deleterious	0.01	25	0.00000
ATM	11	108160398	C	T	rs544891616	NO	missense	ENST00000278616.4:c.4306C>T	ENSP00000278616.4:p.His1436Tyr	H1436Y	Superfamily_domains:SSF48371,PTHR11139,PTHR11139:SF66	25.4	possibly_damaging	0.493	deleterious	0.04	15	0.00015
ATM	11	108160416	T	C	rs201666889,CM0910502	NO	missense	ENST00000278616.4:c.4324T>C	ENSP00000278616.4:p.Tyr1442His	Y1442H	Superfamily_domains:SSF48371,PTHR11139,PTHR11139:SF66	27.3	probably_damaging	1	deleterious	0	65	0.00150
ATM	11	108160424	G	A	rs753570046	NO	synonymous	ENST00000278616.4:c.4332G>A	NA	L1444L	Superfamily_domains:SSF48371,PTHR11139,PTHR11139:SF66	8.868	NA	NA	NA	NA	NA	0.00000
ATM	11	108160424	G	C	rs753570046	NO	synonymous	ENST00000278616.4:c.4332G>C	NA	L1444L	Superfamily_domains:SSF48371,PTHR11139,PTHR11139:SF66	8.869	NA	NA	NA	NA	NA	0.00003
ATM	11	108160450	T	A	CD052086,rs587782126	NO	missense	ENST00000278616.4:c.4358T>A	ENSP00000278616.4:p.Ile1453Lys	I1453K	Superfamily_domains:SSF48371,PTHR11139,PTHR11139:SF66	24.7	benign	0.258	deleterious	0	35	0.00000
ATM	11	108160454	A	C	CM0910503,rs148993589,COSM22501	NO	missense	ENST00000278616.4:c.4362A>C	ENSP00000278616.4:p.Lys1454Asn	K1454N	Superfamily_domains:SSF48371,PTHR11139,PTHR11139:SF66	15.75	benign	0.041	tolerated	0.15	0	0.00134
ATM	11	108160457	T	A	rs527471560	NO	missense	ENST00000278616.4:c.4365T>A	ENSP00000278616.4:p.Ser1455Arg	S1455R	Superfamily_domains:SSF48371,PTHR11139,PTHR11139:SF66	22.6	benign	0.058	tolerated	0.06	0	0.00000
ATM	11	108160463	A	G	rs765713557	NO	synonymous	ENST00000278616.4:c.4371A>G	NA	L1457L	Superfamily_domains:SSF48371,PTHR11139,PTHR11139:SF66	6.787	NA	NA	NA	NA	NA	0.00000
ATM	11	108160467	G	A	rs145667735	NO	missense	ENST00000278616.4:c.4375G>A	ENSP00000278616.4:p.Gly1459Arg	G1459R	Superfamily_domains:SSF48371,PTHR11139,PTHR11139:SF66	24.4	benign	0.072	deleterious	0	15	0.00009
ATM	11	108160480	T	G	CM022199,rs138327406,COSM22502,COSM1	NO	missense	ENST00000278616.4:c.4388T>G	ENSP00000278616.4:p.Phe1463Cys	F1463C	Superfamily_domains:SSF48371,PTHR11139,PTHR11139:SF66	28.7	probably_damaging	0.984	deleterious	0	55	0.00446
ATM	11	108160488	C	G	CM990210,rs730881369,COSM171676,COSM	NO	missense	ENST00000278616.4:c.4396C>G	ENSP00000278616.4:p.Arg1466Gly	R1466G	Superfamily_domains:SSF48371,PTHR11139,PTHR11139:SF66	29.5	possibly_damaging	0.677	deleterious	0	25	0.00003
ATM	11	108160493	C	T	NA	NO	synonymous	ENST00000278616.4:c.4401C>T	NA	D1467D	Superfamily_domains:SSF48371,PTHR11139,PTHR11139:SF66	15.06	NA	NA	NA	NA	NA	0.00000
ATM	11	108160506	T	G	rs539676759	NO	missense	ENST00000278616.4:c.4414T>G	ENSP00000278616.4:p.Leu1472Val	L1472V	Superfamily_domains:SSF48371,PTHR11139,PTHR11139:SF66	23.3	possibly_damaging	0.903	deleterious	0.01	15	0.00013
ATM	11	108160508	G	A	rs201526888,COSM77308	NO	synonymous	ENST00000278616.4:c.4416G>A	NA	L1472L	Superfamily_domains:SSF48371,PTHR11139,PTHR11139:SF66	9.586	NA	NA	NA	NA	NA	0.00000
ATM	11	108160516	A	G	rs34640941,COSM22503	NO	missense	ENST00000278616.4:c.4424A>G	ENSP00000278616.4:p.Tyr1475Cys	Y1475C	Superfamily_domains:SSF48371,PTHR11139,PTHR11139:SF66	18.12	benign	0.003	tolerated	0.1	15	0.00153
ATM	11	108160518	A	G	NA	NO	missense	ENST00000278616.4:c.4426A>G	ENSP00000278616.4:p.Ile1476Val	I1476V	Superfamily_domains:SSF48371,PTHR11139,PTHR11139:SF66	18.66	benign	0.347	tolerated	0.09	15	0.00000
ATM	11	108160538	A	G	NA	NO	regulatory	ENST00000278616.4:c.4436+10A>G	NA	NA	NA	4.83	NA	NA	NA	NA	NA	0.00000
ATM	11	108163316	T	G	NA	NO	intron	ENST00000278616.4:c.4437-30T>G	NA	NA	NA	7.596	NA	NA	NA	NA	NA	0.00000
ATM	11	108163323	G	T	rs569919428	NO	intron	ENST00000278616.4:c.4437-23G>T	NA	NA	NA	0.683	NA	NA	NA	NA	NA	0.00000
ATM	11	108163339	A	G	rs370354306	NO	splice,intron	ENST00000278616.4:c.4437-7A>G	NA	NA	NA	0.414	NA	NA	NA	NA	NA	0.00000
ATM	11	108163375	G	A	rs201594549,COSM200666	NO	missense	ENST00000278616.4:c.4466G>A	ENSP00000278616.4:p.Arg1489His	R1489H	Superfamily_domains:SSF48371,PTHR11139,PTHR11139:SF66	24.9	benign	0.033	deleterious	0.03	0	0.00003
ATM	11	108163382	C	T	rs4988008	NO	synonymous	ENST00000278616.4:c.4473C>T	NA	F1491F	Superfamily_domains:SSF48371,PTHR11139,PTHR11139:SF66	16.65	NA	NA	NA	NA	NA	0.00441
ATM	11	108163397	C	T	rs772833802	NO	synonymous	ENST00000278616.4:c.4488C>T	NA	D1496D	Superfamily_domains:SSF48371,PTHR11139,PTHR11139:SF66	12.58	NA	NA	NA	NA	NA	0.00000
ATM	11	108163401	T	C	rs748949478	NO	synonymous	ENST00000278616.4:c.4492T>C	NA	L1498L	Superfamily_domains:SSF48371,PTHR11139,PTHR11139:SF66	1.01	NA	NA	NA	NA	NA	0.00006
ATM	11	108163414	G	T	rs759340881	NO	missense	ENST00000278616.4:c.4505G>T	ENSP00000278616.4:p.Cys1502Phe	C1502F	Superfamily_domains:SSF48371,PTHR11139,PTHR11139:SF66	24.7	benign	0.441	deleterious	0.03	15	0.00003
ATM	11	108163453	A	C	NA	NO	missense	ENST00000278616.4:c.4544A>C	ENSP00000278616.4:p.Asn1515Thr	N1515T	Superfamily_domains:SSF48371,PTHR11139,PTHR11139:SF66	8.767	benign	0.002	tolerated	0.59	0	0.00000
ATM	11	108163473	G	A	NA	NO	missense	ENST00000278616.4:c.4564G>A	ENSP00000278616.4:p.Gly1522Ser	G1522S	Superfamily_domains:SSF48371,PTHR11139,PTHR11139:SF66	23.4	benign	0.42	tolerated	0.18	55	0.00000
ATM	11	108163476	A	G	rs113854991	NO	missense	ENST00000278616.4:c.4567A>G	ENSP00000278616.4:p.Thr1523Ala	T1523A	Superfamily_domains:SSF48371,PTHR11139,PTHR11139:SF66	19.99	benign	0.292	tolerated	0.3	0	0.00000
ATM	11	108163487	C	T	rs1800889	NO	synonymous	ENST00000278616.4:c.4578C>T	NA	P1526P	Superfamily_domains:SSF48371,PTHR11139,PTHR11139:SF66	12.84	NA	NA	NA	NA	NA	0.09200
ATM	11	108163500	C	T	NA	YES	stop_gained	ENST00000278616.4:c.4591C>T	ENSP00000278616.4:p.Gln1531Ter	Q1531*	Superfamily_domains:SSF48371,PTHR11139,PTHR11139:SF66	35	NA	NA	NA	NA	NA	0.00000
ATM	11	108164036	T	G	rs569983068	NO	splice,intron	ENST00000278616.4:c.4612-4T>G	NA	NA	NA	14	NA	NA	NA	NA	NA	0.00000
ATM	11	108164059	A	G	rs779718362	NO	missense	ENST00000278616.4:c.4631A>G	ENSP00000278616.4:p.Tyr1544Cys	Y1544C	PTHR11139,PTHR11139:SF66	25.1	possibly_damaging	0.806	deleterious	0	55	0.00012
ATM	11	108164068	T	C	NA	NO	missense	ENST00000278616.4:c.4640T>C	ENSP00000278616.4:p.Ile1547Thr	I1547T	PTHR11139,PTHR11139:SF66	26.9	possibly_damaging	0.843	deleterious	0.01	25	0.00000
ATM	11	108164152	G	A	rs550552791,CM0910508,COSM428360,COSM	NO	missense	ENST00000278616.4:c.4724G>A	ENSP00000278616.4:p.Arg1575His	R1575H	PTHR11139,PTHR11139:SF66	33	probably_damaging	0.953	deleterious	0.01	25	0.00033
ATM	11	108164159	T	C	rs145236132	NO	synonymous	ENST00000278616.4:c.4731T>C	NA	T1577T	PTHR11139,PTHR11139:SF66	5.971	NA	NA	NA	NA	NA	0.00003
ATM	11	108164187	C	G	NA	NO	missense	ENST00000278616.4:c.4759C>G	ENSP00000278616.4:p.Pro1587Ala	P1587A	PTHR11139,PTHR11139:SF66	9.859	benign	0.001	tolerated	0.66	0	0.00000
ATM	11	108164194	C	T	NA	NO	missense	ENST00000278616.4:c.4766C>T	ENSP00000278616.4:p.Ser1589Leu	S1589L	PTHR11139,PTHR11139:SF66	24.5	benign	0.011	deleterious	0.01	15	0.00000
ATM	11	108164229	A	G	rs775919064	NO	intron	ENST00000278616.4:c.4776+25A>G	NA	NA	NA	7.322	NA	NA	NA	NA	NA	0.00000
ATM	11	108164245	C	T	rs114726455	NO	intron	ENST00000278616.4:c.4776+41C>T	NA	NA	NA	3.545	NA	NA	NA	NA	NA	0.00000
ATM	11	108164245	CAT	C	rs759726222	NO	intron,feature_truncation	ENST00000278616.4:c.4776+43_4776+44delTA	NA	NA	NA	7.465	NA	NA	NA	NA	NA	0.00000
ATM	11	108165628	A	G	NA	NO	intron	ENST00000278616.4:c.4777-26A>G	NA	NA	NA	6.604	NA	NA	NA	NA	NA	0.00000
ATM	11	108165634	A	G	rs3218678	NO	intron	ENST00000278616.4:c.4777-20A>G	NA	NA	NA	2.868	NA	NA	NA	NA	NA	0.00000
ATM	11	108165656	A	G	rs587778076	NO	splice,synonymous	ENST00000278616.4:c.4779A>G	NA	E1593	PTHR11139,PTHR11139:SF66	13.04	NA	NA	NA	NA	NA	0.00000
ATM	11	108165678	A	G	NA	NO	missense	ENST00000278616.4:c.4801A>G	ENSP00000278616.4:p.Ser1601Gly	S1601G	PTHR11139,PTHR11139:SF66	17.8	benign	0.002	tolerated	0.28	0	0.00000
ATM	11	108165679	G	A	rs587782506	NO	missense	ENST00000278616.4:c.4802G>A	ENSP00000278616.4:p.Ser1601Asn	S1601N	PTHR11139,PTHR11139:SF66	18.24	benign	0.002	tolerated	0.18	0	0.00003
ATM	11	108165693	C	T	rs746103632	NO	missense	ENST00000278616.4:c.4816C>T	ENSP00000278616.4:p.Leu1606Phe	L1606F	PTHR11139,PTHR11139:SF66	25.3	possibly_damaging	0.862	tolerated	0.11	0	0.00000
ATM	11	108165698	A	G	NA	NO	synonymous	ENST00000278616.4:c.4821A>G	NA	P1607P	PTHR11139,PTHR11139:SF66	10.39	NA	NA	NA	NA	NA	0.00000
ATM	11	108165789	G	A	rs778685122	NO	splice,intron	ENST00000278616.4:c.4909+3G>A	NA	NA	NA	13.64	NA	NA	NA	NA	NA	0.00000
ATM	11	108168027	T	C	NA	NO	synonymous	ENST00000278616.4:c.4923T>C	NA	D1641D	Superfamily_domains:SSF48371,PTHR11139,PTHR11139:SF66	6.902	NA	NA	NA	NA	NA	0.00000
ATM	11	108168036	G	A	rs730881371,COSM922723,COSM1585350	NO	missense	ENST00000278616.4:c.4932G>A	ENSP00000278616.4:p.Met1644Ile	M1644I	Superfamily_domains:SSF48371,PTHR11139,PTHR11139:SF66	18.39	benign	0.001	tolerated	0.37	0	0.00000
ATM	11	108168053	A	G	rs55870064	NO	missense	ENST00000278616.4:c.4949A>G	ENSP00000278616.4:p.Asn1650Ser	N1650S	Superfamily_domains:SSF48371,PTHR11139,PTHR11139:SF66	4.686	benign	0.001	tolerated	1	0	0.00000
ATM	11	108168084	C	T	rs144338238	NO	synonymous	ENST00000278616.4:c.4980C>T	NA	N1660N	Superfamily_domains:SSF48371,PTHR11139,PTHR11139:SF66	14.12	NA	NA	NA	NA	NA	0.00060
ATM	11	108168092	G	A	COSM346969	NO	missense	ENST00000278616.4:c.4988G>A	ENSP00000278616.4:p.Gly1663Asp	G1663D	Superfamily_domains:SSF48371,PTHR11139,PTHR11139:SF66	23.1	benign	0.134	tolerated	0.14	0	0.00000
ATM	11	108168106	C	T	rs747317946	NO	synonymous	ENST00000278616.4:c.5002C>T	NA	L1668L	Superfamily_domains:SSF48371,PTHR11139,PTHR11139:SF66	14.15	NA	NA	NA	NA	NA	0.00006
ATM	11	108168115	CTA	C	rs587780626	NO	splice,intron,feature_truncation	ENST00000278616.4:c.5005+7_5005+8delTA	NA	NA	NA	18.17	NA	NA	NA	NA	NA	0.00000
ATM	11	108168123	A	T	rs374337879	NO	intron	ENST00000278616.4:c.5005+14A>T	NA	NA	NA	2.507	NA	NA	NA	NA	NA	0.00000
ATM	11	108168124	T	A	NA	NO	intron	ENST00000278616.4:c.5005+15T>A	NA	NA	NA	7.042	NA	NA	NA	NA	NA	0.00000
ATM	11	108168130	G	GGC	rs547799081	NO	intron,feature_elongation	ENST00000278616.4:c.5005+21_5005+22insGC	NA	NA	NA	0.696	NA	NA	NA	NA	NA	0.00000
ATM	11	108170366	T	C	NA	NO	intron	ENST00000278616.4:c.5006-75T>C	NA	NA	NA	7.468	NA	NA	NA	NA	NA	0.00000
ATM	11	108170373	T	G	rs3092906	NO	intron	ENST00000278616.4:c.5006-68T>G	NA	NA	NA	8.257	NA	NA	NA	NA	NA	0.00000
ATM	11	108170378	A	T	NA	NO	intron	ENST00000278616.4:c.5006-63A>T	NA	NA	NA	7.156	NA	NA	NA	NA	NA	0.00000
ATM	11	108170385	G	A	NA	NO	intron	ENST00000278616.4:c.5006-56G>A	NA	NA	NA	7.351	NA	NA	NA	NA	NA	0.00000
ATM	11	108170392	G	GT	rs780690344	NO	intron,feature_elongation	ENST00000278616.4:c.5006-48dupT	NA	NA	NA	4.499	NA	NA	NA	NA	NA	0.00000
ATM	11	108170423	T	A	rs768321973	NO	intron	ENST00000278616.4:c.5006-18T>A	NA	NA	NA	6.655	NA	NA	NA	NA	NA	0.00000
ATM	11	108170477	T	C	COSM1350879,COSM1350878	NO	missense	ENST00000278616.4:c.5042T>C	ENSP00000278616.4:p.Ile1681Thr	I1681T	Superfamily_domains:SSF48371,PTHR11139,PTHR11139:SF66	16.4	benign	0.019	tolerated	0.09	0	0.00000
ATM	11	108170491	A	G	rs145453814	NO	missense	ENST00000278616.4:c.5056A>G	ENSP00000278616.4:p.Ile1686Val	I1686V	Superfamily_domains:SSF48371,PTHR11139,PTHR11139:SF66	19.23	benign	0.099	tolerated	0.14	25	0.00009
ATM	11	108170504	A	G	NA	NO	missense	ENST00000278616.4:c.5069A>G	ENSP00000278616.4:p.His1690Arg	H1690R	Superfamily_domains:SSF48371,PTHR11139,PTHR11139:SF66	13.58	benign	0.003	tolerated	0.22	0	0.00000
ATM	11	108170506	A	C	CM980141,rs1800059	NO	missense	ENST00000278616.4:c.5071A>C	ENSP00000278616.4:p.Ser1691Arg	S1691R	Superfamily_domains:SSF48371,PTHR11139,PTHR11139:SF66	11.03	benign	0.003	tolerated	0.42	0	0.00623
ATM	11	108170524	A	G	rs142455912,CM024584	NO	missense	ENST00000278616.4:c.5089A>G	ENSP00000278616.4:p.Thr1697Ala	T1697A	Superfamily_domains:SSF48371,PTHR11139,PTHR11139:SF66	0.255	benign	0.002	tolerated	0.81	0	0.00012
ATM	11	108170674	C	A	NA	NO	intron	ENST00000278616.4:c.5177+62C>A	NA	NA	NA	4.892	NA	NA	NA	NA	NA	0.00000
ATM	11	108170680	A	G	rs558610972	NO	intron	ENST00000278616.4:c.5177+68A>G	NA	NA	NA	6.493	NA	NA	NA	NA	NA	0.00000
ATM	11	108170684	G	T	NA	NO	intron	ENST00000278616.4:c.5177+72G>T	NA	NA	NA	0.68	NA	NA	NA	NA	NA	0.00000
ATM	11	108170698	C	T	rs531375734	NO	intron	ENST00000278616.4:c.5177+86C>T	NA	NA	NA	3.042	NA	NA	NA	NA	NA	0.00000
ATM	11	108170703	G	A	NA	NO	intron	ENST00000278616.4:c.5177+91G>A	NA	NA	NA	1.222	NA	NA	NA	NA	NA	0.00000
ATM	11	108172364	G	A	rs200876654	NO	intron	ENST00000278616.4:c.5178-11G>A	NA	NA	NA	3.821	NA	NA	NA	NA	NA	0.00000
ATM	11	108172370	T	A	NA	NO	splice,intron	ENST00000278616.4:c.5178-5T>A	NA	NA	NA	13.55	NA	NA	NA	NA	NA	0.00000
ATM	11	108172383	T	C	CM0910510	NO	missense	ENST00000278616.4:c.5186T>C	ENSP00000278616.4:p.Val1729Ala	V1729A	Superfamily_domains:SSF48371,PTHR11139,PTHR11139:SF66	26	possibly_damaging	0.611	deleterious	0	25	0.00000
ATM	11	108172392	C	G	NA	NO	missense	ENST00000278616.4:c.5195C>G	ENSP00000278616.4:p.Ala1732Gly	A1732G	Superfamily_domains:SSF48371,PTHR11139,PTHR11139:SF66	31	probably_damaging	0.971	deleterious	0.01	0	0.00000
ATM	11	108172425	C	T	rs587779844,CM980142,COSM21935	NO	missense	ENST00000278616.4:c.5228C>T	ENSP00000278616.4:p.Thr1743Ile	T1743I	Superfamily_domains:SSF48371,PTHR11139,PTHR11139:SF66	28.1	possibly_damaging	0.459	deleterious	0.02	65	0.00006
ATM	11	108172427	A	G	NA	NO	missense	ENST00000278616.4:c.5230A>G	ENSP00000278616.4:p.Lys1744Glu	K1744E	Superfamily_domains:SSF48371,PTHR11139,PTHR11139:SF66	20.2	benign	0.012	tolerated	0.21	0	0.00000
ATM	11	108172430	A	C	rs587782532	NO	missense	ENST00000278616.4:c.5233A>C	ENSP00000278616.4:p.Thr1745Pro	T1745P	Superfamily_domains:SSF48371,PTHR11139,PTHR11139:SF66	15.24	benign	0.002	tolerated	0.15	0	0.00000
ATM	11	108172439	A	G	NA	NO	missense	ENST00000278616.4:c.5242A>G	ENSP00000278616.4:p.Ser1748Gly	S1748G	Superfamily_domains:SSF48371,PTHR11139,PTHR11139:SF66	10.17	benign	0	tolerated	0.34	0	0.00000
ATM	11	108172459	G	T	CM035779,rs748900588	NO	missense	ENST00000278616.4:c.5262G>T	ENSP00000278616.4:p.Lys1754Asn	K1754N	Superfamily_domains:SSF48371,PTHR11139,PTHR11139:SF66	22.4	benign	0.013	tolerated	0.06	0	0.00009

ATM	11	108172464	C	G	NA	NO	missense	ENST00000278616.4:c.5267C>G	ENSP00000278616.4:p.Thr1756Arg	T1756R	Superfamily_domains:SSF48371,PTHR11139,PTHR11139:SF66	13.1	benign	0.001	tolerated	0.43	0	0.00000
ATM	11	108172486	TC	T	rs587779846,CD991607,COSM21933	YES	frameshift,feature_truncation	ENST00000278616.4:c.5290delC	ENSP00000278616.4:p.Leu1764TyrfsTer12	L1764X	Superfamily_domains:SSF48371,PTHR11139,PTHR11139:SF66	34	NA	NA	NA	NA	NA	0.00003
ATM	11	108172491	A	G	rs35556390	NO	missense	ENST00000278616.4:c.5294A>G	ENSP00000278616.4:p.Gln1765Arg	Q1765R	Superfamily_domains:SSF48371,PTHR11139,PTHR11139:SF66	14.37	benign	0.002	tolerated	0.44	0	0.00000
ATM	11	108172533	A	G	NA	NO	intron	ENST00000278616.4:c.5319+17A>G	NA	NA	NA	9.284	NA	NA	NA	NA	NA	0.00000
ATM	11	108172545	T	C	rs746785712	NO	intron	ENST00000278616.4:c.5319+29T>C	NA	NA	NA	5.968	NA	NA	NA	NA	NA	0.00000
ATM	11	108173547	C	T	rs749105235	NO	intron	ENST00000278616.4:c.5320-33C>T	NA	NA	NA	10.32	NA	NA	NA	NA	NA	0.00000
ATM	11	108173577	T	C	NA	NO	splice,intron	ENST00000278616.4:c.5320-3T>C	NA	NA	NA	12.79	NA	NA	NA	NA	NA	0.00000
ATM	11	108173583	T	G	rs768820804	NO	missense	ENST00000278616.4:c.5323T>G	ENSP00000278616.4:p.Leu1775Val	L1775V	PTHR11139,PTHR11139:SF66	10.59	benign	0.437	tolerated	0.85	0	0.00000
ATM	11	108173612	C	T	rs140641762	NO	synonymous	ENST00000278616.4:c.5352C>T	NA	N1784N	PTHR11139,PTHR11139:SF66	4.862	NA	NA	NA	NA	NA	0.00006
ATM	11	108173655	A	G	NA	NO	missense	ENST00000278616.4:c.5395A>G	ENSP00000278616.4:p.Ser1799Gly	S1799G	PTHR11139,PTHR11139:SF66	1.395	benign	0.001	tolerated	0.43	0	0.00000
ATM	11	108173665	A	G	NA	NO	missense	ENST00000278616.4:c.5405A>G	ENSP00000278616.4:p.His1802Arg	H1802R	PTHR11139,PTHR11139:SF66	24.2	probably_damaging	0.987	deleterious	0	25	0.00000
ATM	11	108173669	C	T	NA	NO	synonymous	ENST00000278616.4:c.5409C>T	NA	D1803D	PTHR11139,PTHR11139:SF66	7.89	NA	NA	NA	NA	NA	0.00000
ATM	11	108173670	A	T	rs769872474,CM0910512	NO	missense	ENST00000278616.4:c.5410A>T	ENSP00000278616.4:p.Ile1804Phe	I1804F	PTHR11139,PTHR11139:SF66	0.06	benign	0.025	tolerated	0.29	0	0.00003
ATM	11	108173730	C	T	rs764784077	NO	missense	ENST00000278616.4:c.5470C>T	ENSP00000278616.4:p.Leu1824Phe	L1824F	PTHR11139,PTHR11139:SF66	24.3	benign	0.112	deleterious	0.01	15	0.00000
ATM	11	108175356	C	T	NA	NO	intron	ENST00000278616.4:c.5497-46C>T	NA	NA	NA	2.423	NA	NA	NA	NA	NA	0.00000
ATM	11	108175387	G	C	rs3092828	NO	intron	ENST00000278616.4:c.5497-15G>C	NA	NA	NA	0.772	NA	NA	NA	NA	NA	0.00000
ATM	11	108175394	T	C	rs3092829	NO	splice,intron	ENST00000278616.4:c.5497-8T>C	NA	NA	NA	3.28	NA	NA	NA	NA	NA	0.00000
ATM	11	108175400	A	C	CS991306	YES	splice_acceptor	ENST00000278616.4:c.5497-2A>C	NA	NA	NA	24	NA	NA	NA	NA	NA	0.00000
ATM	11	108175420	C	T	CM980143	YES	stop_gained	ENST00000278616.4:c.5515C>T	ENSP00000278616.4:p.Gln1839Ter	Q1839*	PTHR11139,PTHR11139:SF66	46	NA	NA	NA	NA	NA	0.00000
ATM	11	108175427	T	C	NA	NO	missense	ENST00000278616.4:c.5522T>C	ENSP00000278616.4:p.Val1841Ala	V1841A	PTHR11139,PTHR11139:SF66	19.68	benign	0.045	tolerated	0.14	25	0.00000
ATM	11	108175429	C	T	NA	NO	missense	ENST00000278616.4:c.5524C>T	ENSP00000278616.4:p.Leu1842Phe	L1842F	PTHR11139,PTHR11139:SF66	24.4	possibly_damaging	0.607	tolerated	0.2	0	0.00000
ATM	11	108175455	A	G	rs35850088	NO	synonymous	ENST00000278616.4:c.5550A>G	NA	L1850L	PTHR11139,PTHR11139:SF66	4.996	NA	NA	NA	NA	NA	0.00000
ATM	11	108175456	C	T	rs200842502	NO	missense	ENST00000278616.4:c.5551C>T	ENSP00000278616.4:p.Leu1851Phe	L1851F	PTHR11139,PTHR11139:SF66	22.3	benign	0.045	tolerated	0.12	0	0.00000
ATM	11	108175459	C	T	rs754562056	YES	stop_gained	ENST00000278616.4:c.5554C>T	ENSP00000278616.4:p.Gln1852Ter	Q1852*	PTHR11139,PTHR11139:SF66	42	NA	NA	NA	NA	NA	0.00003
ATM	11	108175462	G	A	CM077896,rs1801516,COSM41596,COSM373	NO	missense	ENST00000278616.4:c.5557G>A	ENSP00000278616.4:p.Asp1853Asn	D1853N	PTHR11139,PTHR11139:SF66	23.2	benign	0.029	tolerated	0.23	0	0.26659
ATM	11	108175463	A	T	rs1801673,CM083593,COSM1350895,COSM2	NO	missense	ENST00000278616.4:c.5558A>T	ENSP00000278616.4:p.Asp1853Val	D1853V	PTHR11139,PTHR11139:SF66	23.6	benign	0.137	deleterious	0	15	0.01384
ATM	11	108175485	T	C	NA	NO	synonymous	ENST00000278616.4:c.5580T>C	NA	N1860N	PTHR11139,PTHR11139:SF66	2.99	NA	NA	NA	NA	NA	0.00000
ATM	11	108175488	G	A	rs781383087	NO	synonymous	ENST00000278616.4:c.5583G>A	NA	L1861L	PTHR11139,PTHR11139:SF66	7.463	NA	NA	NA	NA	NA	0.00012
ATM	11	108175491	T	C	NA	NO	synonymous	ENST00000278616.4:c.5586T>C	NA	L1862L	PTHR11139,PTHR11139:SF66	0.279	NA	NA	NA	NA	NA	0.00000
ATM	11	108175500	T	C	rs772261410	NO	synonymous	ENST00000278616.4:c.5595T>C	NA	H1865H	PTHR11139,PTHR11139:SF66	0.035	NA	NA	NA	NA	NA	0.00000
ATM	11	108175528	C	T	rs376603775,CM980144,COSM1350897,COSM	YES	stop_gained	ENST00000278616.4:c.5623C>T	ENSP00000278616.4:p.Arg1875Ter	R1875*	PTHR11139,PTHR11139:SF66	36	NA	NA	NA	NA	NA	0.00006
ATM	11	108175544	C	T	rs587780628,COSM4145212,COSM4145211	NO	missense	ENST00000278616.4:c.5639C>T	ENSP00000278616.4:p.Thr1880Met	T1880M	PTHR11139,PTHR11139:SF66	12.07	benign	0.011	tolerated	0.06	0	0.00000
ATM	11	108175549	C	T	CM030193	YES	stop_gained	ENST00000278616.4:c.5644C>T	ENSP00000278616.4:p.Arg1882Ter	R1882*	PTHR11139,PTHR11139:SF66	38	NA	NA	NA	NA	NA	0.00000
ATM	11	108175574	A	G	NA	NO	missense	ENST00000278616.4:c.5669A>G	ENSP00000278616.4:p.Asp1890Gly	D1890G	PTHR11139,PTHR11139:SF66	23.7	benign	0.009	deleterious	0.01	0	0.00000
ATM	11	108175610	G	A	rs550070837	NO	intron	ENST00000278616.4:c.5674+31G>A	NA	NA	NA	6.376	NA	NA	NA	NA	NA	0.00000
ATM	11	108175640	A	G	NA	NO	intron	ENST00000278616.4:c.5674+61A>G	NA	NA	NA	3.508	NA	NA	NA	NA	NA	0.00000
ATM	11	108178661	A	C	NA	NO	missense	ENST00000278616.4:c.5712A>C	ENSP00000278616.4:p.Lys1904Asn	K1904N	PTHR11139,PTHR11139:SF66	12.25	benign	0.001	tolerated	0.52	0	0.00000
ATM	11	108178668	A	C	NA	NO	synonymous	ENST00000278616.4:c.5719A>C	NA	R1907R	PTHR11139,PTHR11139:SF66	0.708	NA	NA	NA	NA	NA	0.00000
ATM	11	108178702	G	C	rs148064985	NO	missense	ENST00000278616.4:c.5753G>C	ENSP00000278616.4:p.Arg1918Thr	R1918T	PTHR11139,PTHR11139:SF66	22.1	benign	0.206	tolerated	0.32	0	0.00025
ATM	11	108180832	A	G	NA	NO	intron	ENST00000278616.4:c.5763-55A>G	NA	NA	NA	0.297	NA	NA	NA	NA	NA	0.00000
ATM	11	108180841	T	C	rs776492831	NO	intron	ENST00000278616.4:c.5763-46T>C	NA	NA	NA	5.95	NA	NA	NA	NA	NA	0.00000
ATM	11	108180917	T	C	rs3092910	NO	synonymous	ENST00000278616.4:c.5793T>C	NA	A1931A	PTHR11139,PTHR11139:SF66	7.568	NA	NA	NA	NA	NA	0.01263
ATM	11	108180945	G	C	rs147187700,CM094667,COSM21922	NO	missense	ENST00000278616.4:c.5821G>C	ENSP00000278616.4:p.Val1941Leu	V1941L	PTHR11139,PTHR11139:SF66	24.5	possibly_damaging	0.573	tolerated	0.08	0	0.00030
ATM	11	108181006	A	G	CM094670,rs56399311,COSM21938	NO	missense	ENST00000278616.4:c.5882A>G	ENSP00000278616.4:p.Tyr1961Cys	Y1961C	PTHR11139,PTHR11139:SF66,PROSITE_profiles:PS51189	28.3	probably_damaging	0.993	deleterious	0	45	0.00012
ATM	11	108181011	G	A	NA	NO	missense	ENST00000278616.4:c.5887G>A	ENSP00000278616.4:p.Asp1963Asn	D1963N	PTHR11139,PTHR11139:SF66,PROSITE_profiles:PS51189	25.5	possibly_damaging	0.473	tolerated	0.22	0	0.00000
ATM	11	108181014	A	G	rs201963507,CM085264,CM0910514	NO	missense	ENST00000278616.4:c.5890A>G	ENSP00000278616.4:p.Lys1964Glu	K1964E	PTHR11139,PTHR11139:SF66,PROSITE_profiles:PS51189	17.81	benign	0.025	tolerated	0.45	0	0.00024
ATM	11	108181032	CA	C	NA	YES	frameshift,feature_truncation	ENST00000278616.4:c.5910delA	ENSP00000278616.4:p.Glu1971ArgfsTer19	Q1970X	PTHR11139,PTHR11139:SF66,PROSITE_profiles:PS51189	35	NA	NA	NA	NA	NA	0.00000
ATM	11	108181048	G	A	NA	NO	splice,intron	ENST00000278616.4:c.5918+6G>A	NA	NA	NA	11.04	NA	NA	NA	NA	NA	0.00000
ATM	11	108181059	T	G	NA	NO	intron	ENST00000278616.4:c.5918+17T>G	NA	NA	NA	15.27	NA	NA	NA	NA	NA	0.00000
ATM	11	108181065	G	T	NA	NO	intron	ENST00000278616.4:c.5918+23G>T	NA	NA	NA	7.388	NA	NA	NA	NA	NA	0.00000
ATM	11	108183137	G	C	NA	YES	splice_acceptor	ENST00000278616.4:c.5919-1G>C	NA	NA	NA	22.4	NA	NA	NA	NA	NA	0.00000
ATM	11	108183140	G	GAACTTATTCA	NA	NO	inframe_insertion	ENST00000278616.4:c.5921_5922insAACTTATTCAAA	ENSP00000278616.4:p.Ser1974delinsArgThrTyrSerAs	S1974RTYSN	PTHR11139,PTHR11139:SF66,PROSITE_profiles:PS51189	11.89	NA	NA	NA	NA	NA	0.00000
ATM	11	108183151	G	T	rs587779852,CS991307,CM980147,COSM123	YES	stop_gained	ENST00000278616.4:c.5932G>T	ENSP00000278616.4:p.Glu1978Ter	E1978*	PTHR11139,PTHR11139:SF66,PROSITE_profiles:PS51189	53	NA	NA	NA	NA	NA	0.00021
ATM	11	108183172	A	G	NA	NO	missense	ENST00000278616.4:c.5953A>G	ENSP00000278616.4:p.Thr1985Ala	T1985A	PTHR11139,PTHR11139:SF66,PROSITE_profiles:PS51189	12.62	benign	0.002	tolerated	0.45	0	0.00000
ATM	11	108183175	A	G	NA	NO	missense	ENST00000278616.4:c.5956A>G	ENSP00000278616.4:p.Ile1986Val	I1986V	PTHR11139,PTHR11139:SF66,PROSITE_profiles:PS51189	16.94	benign	0.263	tolerated	0.32	0	0.00000
ATM	11	108183193	AAAAGT	A	rs749049519	YES	frameshift,feature_truncation	ENST00000278616.4:c.5979_5983delTAAAG	ENSP00000278616.4:p.Ser1993ArgfsTer23	KS1992-1993X	PTHR11139,PTHR11139:SF66,PROSITE_profiles:PS51189	35	NA	NA	NA	NA	NA	0.00000
ATM	11	108183194	A	C	rs150757822	NO	missense	ENST00000278616.4:c.5975A>C	ENSP00000278616.4:p.Lys1992Thr	K1992T	PTHR11139,PTHR11139:SF66,PROSITE_profiles:PS51189	23.6	possibly_damaging	0.459	tolerated	0.08	0	0.00093
ATM	11	108183226	G	C	NA	YES	splice_donor	ENST00000278616.4:c.6006+1G>C	NA	NA	NA	25.6	NA	NA	NA	NA	NA	0.00000
ATM	11	108183233	T	C	rs56019194	NO	splice,intron	ENST00000278616.4:c.6006+8T>C	NA	NA	NA	9.444	NA	NA	NA	NA	NA	0.00000
ATM	11	108186499	A	G	rs372978004	NO	intron	ENST00000278616.4:c.6007-51A>G	NA	NA	NA	6.483	NA	NA	NA	NA	NA	0.00000
ATM	11	108186503	A	T	rs755604182	NO	intron	ENST00000278616.4:c.6007-47A>T	NA	NA	NA	5.922	NA	NA	NA	NA	NA	0.00000
ATM	11	108186563	A	G	rs762001297	NO	missense	ENST00000278616.4:c.6020A>G	ENSP00000278616.4:p.Glu2007Gly	E2007G	PTHR11139,PTHR11139:SF66,PROSITE_profiles:PS51189	28.4	possibly_damaging	0.786	deleterious	0	15	0.00003
ATM	11	108186568	T	C	rs199586999	NO	missense	ENST00000278616.4:c.6025T>C	ENSP00000278616.4:p.Tyr2009His	Y2009H	PTHR11139,PTHR11139:SF66,PROSITE_profiles:PS51189	27.7	probably_damaging	0.963	deleterious	0	35	0.00006
ATM	11	108186599	A	G	CM094662,CD982462,COSM1235436,COSM2	NO	missense	ENST00000278616.4:c.6056A>G	ENSP00000278616.4:p.Tyr2019Cys	Y2019C	PTHR11139,PTHR11139:SF66,PROSITE_profiles:PS51189	27.2	possibly_damaging	0.823	deleterious	0	65	0.00000
ATM	11	108186610	G	A	rs11212587,CM092585,COSM24635	NO	missense	ENST00000278616.4:c.6067G>A	ENSP00000278616.4:p.Gly2023Arg	G2023R	PTHR11139,PTHR11139:SF66,PROSITE_profiles:PS51189	31	probably_damaging	0.973	deleterious	0	25	0.00465
ATM	11	108186631	A	G	CM014793,rs145847315	NO	missense	ENST00000278616.4:c.6088A>G	ENSP00000278616.4:p.Ile2030Val	I2030V	PTHR11139,PTHR11139:SF66,PROSITE_profiles:PS51189	11.21	benign	0.001	tolerated	0.46	0	0.00012
ATM	11	108186644	T	C	NA	NO	splice,intron	ENST00000278616.4:c.6095+6T>C	NA	NA	NA	4.128	NA	NA	NA	NA	NA	0.00000
ATM	11	108186653	T	C	rs3212321	NO	intron	ENST00000278616.4:c.6095+15T>C	NA	NA	NA	3.457	NA	NA	NA	NA	NA	0.00000
ATM	11	108186663	G	A	rs769865854	NO	intron	ENST00000278616.4:c.6095+25G>A	NA	NA	NA	2.658	NA	NA	NA	NA	NA	0.00000
ATM	11	108186664	G	A	rs773648739	NO	intron	ENST00000278616.4:c.6095+26G>A	NA	NA	NA	1.713	NA	NA	NA	NA	NA	0.00000
ATM	11	108186683	G	A	NA	NO	intron	ENST00000278616.4:c.6095+45G>A	NA	NA	NA	6.66	NA	NA	NA	NA	NA	0.00000
ATM	11	108186685	A	G	NA	NO	intron	ENST00000278616.4:c.6095+47A>G	NA	NA	NA	7.36	NA	NA	NA	NA	NA	0.00000
ATM	11	108186698	G	A	rs778590586	NO	intron	ENST00000278616.4:c.6096-40G>A	NA	NA	NA	2.998	NA	NA	NA	NA	NA	0.00000
ATM	11	108186708	C	G	NA	NO	intron	ENST00000278616.4:c.6096-30C>G	NA	NA	NA	1.197	NA	NA	NA	NA	NA	0.00000
ATM	11	108186742	C	A	rs532480170,CM960102,COSM922732,COSM	NO	synonymous	ENST00000278616.4:c.6100C>A	NA	R2034R	PTHR11139,PTHR11139:SF66,PROSITE_profiles:PS51189	15.53	NA	NA	NA	NA	NA	0.00003
ATM	11	108186743	G	A	rs3218670,COSM1315815	NO	missense	ENST00000278616.4:c.6101G>A	ENSP00000278616.4:p.Arg2034Gln	R2034Q	PTHR11139,PTHR11139:SF66,PROSITE_profiles:PS51189	23.1	benign	0.018	tolerated	0.08	0	0.00009
ATM	11	108186754	C	T	NA	NO	missense	ENST00000278616.4:c.6112C>T	ENSP00000278616.4:p.His2038Tyr	H2038Y	PTHR11139,PTHR11139:SF66,PROSITE_profiles:PS51189	23.9	benign	0.26	deleterious	0	65	0.00000
ATM	11	108186756	C	T	rs774993357,COSM200670	NO	synonymous	ENST00000278616.4:c.6114C>T	NA	H2038H	PTHR11139,PTHR11139:SF66,PROSITE_profiles:PS51189	13.13	NA	NA	NA	NA	NA	0.00003
ATM	11	108186795	C	T	NA	NO	synonymous	ENST00000278616.4:c.6153C>T	NA	L2051L	PTHR11139,PTHR11139:SF66,PROSITE_profiles:PS51189	11.84	NA	NA	NA	NA	NA	0.00000
ATM	11	108186807	C	A	rs779848229	NO	synonymous	ENST00000278616.4:c.6165C>A	NA	I2055I	PTHR11139,PTHR11139:SF66,PROSITE_profiles:PS51189	19.15	NA	NA	NA	NA	NA	0.00003
ATM	11	108186817	A	G	NA	NO	missense	ENST00000278616.4:c.6175A>G	ENSP00000278616.4:p.Thr2059Ala	T2059A	PTHR11139,PTHR11139:SF66,PROSITE_profiles:PS51189	13.99	benign	0.007	tolerated	0.3	0	0.00000
ATM	11	108186820	C	T	rs587778078	NO	missense	ENST00000278616.4:c.6178C>T	ENSP00000278616.4:p.Arg2060Cys	R2060C	PTHR11139,PTHR11139:SF66,PROSITE_profiles:PS51189	27	benign	0.022	deleterious	0.05	15	0.00006
ATM	11	108186823	C	T	NA	YES	stop_gained	ENST00000278616.4:c.6181C>T	ENSP00000278616.4:p.Gln2061Ter	Q2061*	PTHR11139,PTHR11139:SF66,PROSITE_profiles:PS51189	45	NA	NA	NA	NA	NA	0.00000
ATM	11	108186833	T	TC	NA	YES	frameshift,feature_elongation	ENST00000278616.4:c.6192dupC	ENSP00000278616.4:p.Ile2065HisfsTer23	I2064IX	PTHR11139,PTHR11139:SF66,PROSITE_profiles:PS51189	35	NA	NA	NA	NA	NA	0.00000
ATM	11	108186884	G	A	NA	NO	intron	ENST00000278616.4:c.6198+44G>A	NA	NA	NA	5.162	NA	NA	NA	NA	NA	0.00000
ATM	11	108186885	C	T	NA	NO	intron	ENST00000278616.4:c.6198+45C>T	NA	NA	NA	7.489	NA	NA	NA	NA	NA	0.00000
ATM	11	108188120	C	T	rs752478345	NO	synonymous	ENST00000278616.4:c.6219C>T	NA	L2073L	PTHR11139,PTHR11139:SF66,PROSITE_profiles:PS51189	14.86	NA	NA	NA	NA	NA	0.00000
ATM	11	108188135	C	T	rs569483748	NO	synonymous	ENST00000278616.4:c.6234C>T	NA	S2078S	PTHR11139,PTHR11139:SF66,PROSITE_profiles:PS51189	12.14	NA	NA	NA	NA	NA	0.00018
ATM	11	108188136	G	A	rs1800060	NO	missense	ENST00000278616.4:c.6235G>A	ENSP00000278616.4:p.Val2079Ile	V2079I	PTHR11139,PTHR11139:SF66,PROSITE_profiles:PS51189	0.005	benign	0.001	tolerated	0.47	0	0.00243
ATM	11	108188149	G	C	NA	NO	missense	ENST00000278616.4:c.6248G>C	ENSP00000278616.4:p.Gly2083Ala	G2083A	PTHR11139,PTHR11139:SF66,PROSITE_profiles:PS51189	22.5	benign	0.405	tolerated	0.07	55	0.00000
ATM	11	108188194	T	C	rs587780631	NO	missense	ENST00000278616.4:c.6293T>C	ENSP00000278616.4:p.Leu2098Pro	L2098P	Pfam_domain:PF02259,PTHR11139,PTHR11139:SF66,PROSITE_profiles:PS51189	23.4	benign	0.217	deleterious	0.02	0	0.00003
ATM	11	108188233	A	G	CM035635	NO	missense	ENST00000278616.4:c.6332A>G	ENSP00000278616.4:p.His2111Arg	H2111R	Pfam_domain:PF02259,PTHR11139,PTHR11139:SF66,PROSITE_profiles:PS51189	4.286	benign	0.027	tolerated	0.35	0	0.00000
ATM	11	108188234	T	C	rs55756349	NO	synonymous	ENST00000278616.4:c.6333T>C	NA	H2111H	Pfam_domain:PF02259,PTHR11139,PTHR11139:SF66,PROSITE_profiles:PS51189	0.011	NA	NA	NA	NA	NA	0.00009
ATM	11	108188267	T	C	rs766810400	NO	intron	ENST00000278616.4:c.6347+19T>C	NA	NA	NA	11.65	NA	NA	NA	NA	NA	0.00000
ATM	11	108190625	T	C	NA	NO	regulatory	ENST00000278616.4:c.6348-56T>C	NA	NA	NA	1.898	NA	NA	NA	NA	NA	0.00000
ATM	11	108190627	T	C	rs116924981	NO	regulatory	ENST00000278616.4:c.6348-54T>C	NA	NA	NA	2.495	NA	NA	NA	NA	NA	0.00000
ATM	11	108190633	T	G	NA	NO	regulatory	ENST00000278616.4:c.6348-48T>G	NA	NA	NA	4.404	NA	NA	NA	NA	NA	0.00000
ATM	11	108190638	G	A	NA	NO	regulatory	ENST00000278616.4:c.6348-43G>A	NA	NA	NA	5.288	NA	NA	NA	NA	NA	0.00000
ATM	11	108190652	T	G	rs771588132	NO	regulatory	ENST00000278616.4:c.6348-29T>G	NA	NA	NA	7.248	NA	NA	NA	NA	NA	0.00000
ATM	11	108190673	T	C	rs730881292	NO	splice,intron	ENST00000278616.4:c.6348-8T>C	NA	NA	NA	0.679	NA	NA	NA	NA	NA	0.00000
ATM	11	108190703	T	C	NA	NO	missense	ENST00000278616.4:c.6370T>C	ENSP00000278616.4:p.Tyr2124His	Y2124H	Pfam_domain:PF02259,PTHR11139,PTHR11139:SF66,PROSITE_profiles:PS51189	27.3	probably_damaging	0.996	deleterious	0	25	0.00000
ATM	11	108190727	C	T	rs551408889,COSM3368196,COSM3368195	NO	synonymous	ENST00000278616.4:c.6394C>T	NA	L2132L	Pfam_domain:PF02259,PTHR11139,PTHR11139:SF66,PROSITE_profiles:PS51189	13.42	NA	NA	NA	NA	NA	0.00003
ATM	11	108190736	C	CTT	rs587782554,CI962208	YES	frameshift,feature_elongation	ENST00000278616.4:c.6404_6405insTT	ENSP00000278616.4:p.Arg2136Ter	L2135LX	Pfam_domain:PF02259,PTHR11139,PTHR11139:SF66,PROSITE_profiles:PS51189	35	NA	NA	NA	NA	NA	0.00000
ATM	11	108190764	A	G	NA	NO	missense	ENST00000278616.4:c.6431A>G	ENSP00000278616.4:p.Tyr2144Cys	Y2144C	Pfam_domain:PF02259,PTHR11139,PTHR11139:SF66,PROSITE_profiles:PS51189	23.7	possibly_damaging	0.762	tolerated	0.17	15	0.00000
ATM	11	108190770	G	C	rs56815840	NO	missense	ENST00000278616.4:c.6437G>C	ENSP00000278616.4:p.Ser2146Thr	S2146T	Pfam_domain:PF02259,PTHR11139,PTHR11139:SF66,PROSITE_profiles:PS51189	7.386	benign	0.002	tolerated	1	0	0.00006
ATM	11	108190810	G	A	rs761600797	NO	intron	ENST00000278616.4:c.6452+25G>A	NA	NA	NA	4.403	NA	NA	NA	NA	NA	0.00000
ATM	11	108190810	G	C	rs761600797	NO	intron	ENST00000278616.4:c.6452+25G>C	NA	NA	NA	1.62	NA	NA	NA	NA	NA	0.00000
ATM	11	108190847	A	G	NA	NO	intron	ENST00000278616.4:c.6452+62A>G	NA	NA	NA	5.511	NA	NA	NA	NA	NA	0.00000
ATM	11	108191994	T	C	NA	NO	intron	ENST00000278616.4:c.6453-34T>C	NA	NA	NA	1.696	NA	NA	NA	NA	NA	0.00000
ATM	11	108192000	CAG	C	rs745661253	NO	intron,feature_truncation	ENST00000278616.4:c.6453-25_6453-24delAG	NA	NA	NA	7.915	NA	NA	NA	NA	NA	0.00000
ATM	11	108192040	G	A	rs140423883	NO	synonymous	ENST00000278616.4:c.6465G>A	NA	V2155V	Pfam_domain:PF02259,PTHR11139,PTHR11139:SF66,PROSITE_profiles:PS51189	11.8	NA	NA	NA	NA	NA	0.00009
ATM	11	108192057	G	A	rs756626462	NO	missense	ENST00000278616.4:c.6482G>A	ENSP00000278616.4:p.Arg2161His	R2161H	Pfam_domain:PF02259,PTHR11139,PTHR11139:SF66,PROSITE_profiles:PS51189	24.2	benign	0.031	tolerated	0.08	0	0.00000
ATM	11	108192078	C	T	rs200431631,COSM3443164,COSM3443163	NO	missense	ENST00000278616.4:c.6503C>T	ENSP00000278616.4:p.Ser2168Leu	S2168L	Pfam_domain:PF02259,PTHR11139,PTHR11139:SF66,PROSITE_profiles:PS51189	29.5	possibly_damaging	0.763	tolerated	0.06	0	0.00000
ATM	11	108192082	C	G	NA	NO	synonymous	ENST00000278616.4:c.6507C>G	NA	L2169L	Pfam_domain:PF02259,PTHR11139,PTHR11139:SF66,PROSITE_profiles:PS51189	9.912	NA	NA	NA	NA	NA	0.00000
ATM	11	108192101	T	C	rs143715818	NO	synonymous	ENST00000278616.4:c.6526T>C	NA	L2176L	Pfam_domain:PF02259,PTHR11139,PTHR11139:SF66,PROSITE_profiles:PS51189	1.683	NA	NA	NA	NA	NA	0.00000
ATM	11	108192106	G	A	NA	NO	synonymous	ENST00000278616.4:c.6531G>A	NA	Q2177Q	Pfam_domain:PF02259,PTHR11139,PTHR11139:SF66,PROSITE_profiles:PS51189	8.668	NA	NA	NA	NA	NA	0.00000
ATM	11	108192126	G	C	rs374551964	NO	missense	ENST00000278616.4:c.6551G>C	ENSP00000278616.4:p.Ser2184Thr	S2184T	Pfam_domain:PF02259,PTHR11139,PTHR11139:SF66,PROSITE_profiles:PS51189	14.7	benign	0.102	tolerated	0.09	0	0.00003
ATM	11	108192129	T	C	rs779611511	NO	missense	ENST00000278616.4:c.6554T>C	ENSP00000278616.4:p.Ile2185Thr	I2185T	Pfam_domain:PF02259,PTHR11139,PTHR11139:SF66,PROSITE_profiles:PS51189	22.8	benign	0.015	deleterious	0.05	0	0.00006
ATM	11	108192158	C	T	rs368049107	NO	intron	ENST00000278616.4:c.6572+11C>T	NA	NA	NA	4.731	NA	NA	NA	NA	NA	0.00000
ATM	11	108192159	G	T	rs3218677	NO	intron	ENST00000278616.4:c.6572+12G>T	NA	NA	NA	0.792	NA	NA	NA	NA	NA	0.00000
ATM	11	108196068	T	G	rs730881311	NO	missense	ENST00000278616.4:c.6604T>G	ENSP00000278616.4:p.Tyr2202Asp	Y2202D	Pfam_domain:PF02259,PTHR11139,PTHR11139:SF66,PROSITE_profiles:PS51189	25.3	possibly_damaging	0.88	tolerated	0.08	35	0.00003
ATM	11	108196074	A	G	NA	NO	missense	ENST00000278616.4:c.6610A>G	ENSP00000278616.4:p.Lys2204Glu	K2204E	Pfam_domain:PF02259,PTHR11139,PTHR11139:SF66,PROSITE_profiles:PS51189	23.1	benign	0.38	tolerated	0.13	0	0.00000
ATM	11	108196134	A	G	rs545873723	NO	missense	ENST00000278616.4:c.6670A>G	ENSP00000278616.4:p.Met2224Val	M2224V	Pfam_domain:PF02259,PTHR11139,PTHR11139:SF66,PROSITE_profiles:PS51189	23	benign	0.439	deleterious	0.04	0	0.00000
ATM	11	108196143	C	T	rs564652222,CM990217,COSM1644415,COSM	NO	missense	ENST00000278616.4:c.6679C>T	ENSP00000278616.4:p.Arg2227Cys	R2227C	Pfam_domain:PF02259,PTHR11139,PTHR11139:SF66,PROSITE_profiles:PS51189	35	probably_damaging	1	deleterious	0	65	0.00000
ATM	11	108196164	C	T	rs760602228	NO	synonymous	ENST00000278616.4:c.6700C>T	NA	L2234L	Pfam_domain:PF02259,PTHR11139,PTHR11139:SF66,PROSITE_profiles:PS51189	12.59	NA	NA	NA	NA	NA	0.00006
ATM	11	108196174	A	C	CD002481,rs35118109	NO	missense	ENST00000278616.4:c.6710A>C	ENSP00000278616.4:p.Lys2237Thr	K2237T	Pfam_domain:PF02259,PTHR11139,PTHR11139:SF66,PROSITE_profiles:PS51189	23.4	possibly_damaging	0.716	tolerated	0.11	0	0.00000
ATM	11	108196203	A	G	rs587781521	NO	missense	ENST00000278616.4:c.6739A>G	ENSP00000278616.4:p.Ile2247Val	I2247V	Pfam_domain:PF02259,PTHR11139,PTHR11139:SF66,PROSITE_profiles:PS51189	6.863	benign	0.038	tolerated	0.39	0	0.00000
ATM	11	108196259	C	T	rs3218699	NO	synonymous	ENST00000278616.4:c.6795C>T	NA	F2265F	Pfam_domain:PF02259,PTHR11139,PTHR11139:SF66,PROSITE_profiles:PS51189	13.33	NA	NA	NA	NA	NA	0.00070
ATM	11	108196264	A	T	NA	NO	missense	ENST00000278616.4:c.6800A>T	ENSP00000278616.4:p.Asn2267Ile	N2267I	Pfam_domain:PF02259,PTHR11139,PTHR11139:SF66,PROSITE_profiles:PS51189	31	probably_damaging	0.997	deleterious	0.01	15	0.00000
ATM	11	108196271	G	A	rs587780638	NO	splice,synonymous	ENST00000278616.4:c.6807G>A	NA	Q2269	Pfam_domain:PF02259,PTHR11139,PTHR11139:SF66,PROSITE_profiles:PS51189	11.11	NA	NA	NA	NA	NA	0.00000
ATM	11	108196797	G	A	CM016181,rs567060474,COSM21637	NO	missense	ENST00000278616.4:c.6820G>A	ENSP00000278616.4:p.Ala2274Thr	A2274T	Pfam_domain:PF02259,PTHR11139,PTHR11139:SF66,PROSITE_profiles:PS51189	34	probably_damaging	0.998	deleterious	0.02	55	0.00039
ATM	11	108196809	A	G	NA	NO	missense	ENST00000278616.4:c.6832A>G	ENSP00000278616.4:p.Ile2278Val	I2278V	Pfam_domain:PF02259,PTHR11139,PTHR11139:SF66,PROSITE_profiles:PS51189	23.5	possibly_damaging	0.45	tolerated	0.06	0	0.00000
ATM	11	108196837	G	C	CM016182,rs1800061	NO	missense	ENST00000278616.4:c.6860G>C	ENSP00000278616.4:p.Gly2287Ala	G2287A	Pfam_domain:PF02259,PTHR11139,PTHR11139:SF66,PROSITE_profiles:PS51189	10.23	benign	0.217	tolerated	0.75	0	0.00066
ATM	11	108196865	A	T	rs200735689	NO	synonymous	ENST00000278616.4:c.6888A>T	NA	A2296A	Pfam_domain:PF02259,PTHR11139,PTHR11139:SF66,PROSITE_profiles:PS51189	0.982	NA	NA	NA	NA	NA	0.00006
ATM	11	108196890	CAG	C	NA	YES	frameshift,feature_truncation	ENST00000278616.4:c.6916_6917delAG	ENSP00000278616.4:p.Leu2307CysfsTer65	Q2305X	Pfam_domain:PF02259,PTHR11139,PTHR11139:SF66,PROSITE_profiles:PS51189	36	NA	NA	NA	NA	NA	0.00000
ATM	11	108196896	C	T	rs56009889	NO	missense	ENST00000278616.4:c.6919C>T	ENSP00000278616.4:p.Leu2307Phe	L2307F	Pfam_domain:PF02259,PTHR11139,PTHR11139:SF66,PROSITE_profiles:PS51189	31	probably_damaging	0.979	deleterious	0.02	0	0.00384
ATM	11	108196929	A	G	NA	NO	missense	ENST00000278616.4:c.6952A>G	ENSP00000278616.4:p.Lys2318Glu	K2318E	Pfam_domain:PF02259,PTHR11139,PTHR11139:SF66,PROSITE_profiles:PS51189	24.1	benign	0.37	deleterious	0.03	55	0.00000
ATM	11	108196931	G	C	NA	NO	missense	ENST00000278616.4:c.6954G>C	ENSP00000278616.4:p.Lys2318Asn	K2318N	Pfam_domain:PF02259,PTHR11139,PTHR11139:SF66,PROSITE_profiles:PS51189	17.8	benign	0.103	tolerated	0.12	65	0.00000
ATM	11	108196952	G	A	rs556778314	NO	splice,synonymous	ENST00000278616.4:c.6975G>A	NA	A2325	Pfam_domain:PF02259,PTHR11139,PTHR11139:SF66,PROSITE_profiles:PS51189	11.24	NA	NA	NA	NA	NA	0.00000
ATM	11	108196952	G	C	rs556778314	NO	splice,synonymous	ENST00000278616.4:c.6975G>C	NA	A2325	Pfam_domain:PF02259,PTHR11139,PTHR11139:SF66,PROSITE_profiles:PS51189	9.95	NA	NA	NA	NA	NA	0.00000
ATM	11	108196965	T	A	NA	NO	intron	ENST00000278616.4:c.6975+13T>A	NA	NA	NA	7.472	NA	NA	NA	NA	NA	0.00000
ATM	11	108198341	T	A	rs774791583	NO	intron	ENST00000278616.4:c.6976-31T>A	NA	NA	NA	8.133	NA	NA	NA	NA	NA	0.00000
ATM	11	108198370	A	C	rs587782403	YES	splice_acceptor	ENST00000278616.4:c.6976-2A>C	NA	NA	NA	22.9	NA	NA	NA	NA	NA	0.00000
ATM	11	108198391	T	C	rs4988111	NO	missense	ENST00000278616.4:c.6995T>C	ENSP00000278616.4:p.Leu2332Pro	L2332P	Superfamily_domains:SSF48371,Pfam_domain:PF02259,PTHR11139,PTHR11139:SF66,PROSITE	12.88	benign	0.005	tolerated	0.2	0	0.00012
ATM	11	108198396	T	A	NA	NO	missense	ENST00000278616.4:c.7000T>A	ENSP00000278616.4:p.Tyr2334Asn	Y2334N	Superfamily_domains:SSF48371,Pfam_domain:PF02259,PTHR11139,PTHR11139:SF66,PROSITE	24.7	possibly_damaging	0.691	deleterious	0.01	0	0.00000
ATM	11	108198409	T	C	CM094664	NO	missense	ENST00000278616.4:c.7013T>C	ENSP00000278616.4:p.Leu2338Pro	L2338P	Superfamily_domains:SSF48371,Pfam_domain:PF02259,PTHR11139,PTHR11139:SF66,PROSITE	28.1	probably_damaging	0.992	deleterious	0	65	0.00000
ATM	11	108198458	G	A	rs143489373	NO	synonymous	ENST00000278616.4:c.7062G>A	NA	A2354A	Superfamily_domains:SSF48371,Pfam_domain:PF02259,PTHR11139,PTHR11139:SF66,PROSITE	15.93	NA	NA	NA	NA	NA	0.00040
ATM	11	108198505	C	T	rs730881275	NO	intron	ENST00000278616.4:c.7089+20C>T	NA	NA	NA	6.059	NA	NA	NA	NA	NA	0.00000
ATM	11	108198512	A	G	rs772522124	NO	intron	ENST00000278616.4:c.7089+27A>G	NA	NA	NA	6.932	NA	NA	NA	NA	NA	0.00000
ATM	11	108198519	C	G	rs187869262	NO	intron	ENST00000278616.4:c.7089+34C>G	NA	NA	NA	3.566	NA	NA	NA	NA	NA	0.00000
ATM	11	108199780	A	C	rs376159946	NO	missense	ENST00000278616.4:c.7122A>C	ENSP00000278616.4:p.Glu2374Asp	E2374D	Superfamily_domains:SSF48371,Pfam_domain:PF02259,PTHR11139,PTHR11139:SF66,PROSITE	12.33	benign	0.005	tolerated	0.5	0	0.00000
ATM	11	108199782	G	A	COSM48243	NO	missense	ENST00000278616.4:c.7124G>A	ENSP00000278616.4:p.Ser2375Asn	S2375N	Superfamily_domains:SSF48371,Pfam_domain:PF02259,PTHR11139,PTHR11139:SF66,PROSITE	14.71	benign	0.008	tolerated	0.29	0	0.00000
ATM	11	108199789	T	C	rs373309822	NO	synonymous	ENST00000278616.4:c.7131T>C	NA	D2377D	Superfamily_domains:SSF48371,Pfam_domain:PF02259,PTHR11139,PTHR11139:SF66,PROSITE	4.058	NA	NA	NA	NA	NA	0.00003
ATM	11	108199842	A	T	COSM330624	NO	missense	ENST00000278616.4:c.7184A>T	ENSP00000278616.4:p.Asp2395Val	D2395V	Superfamily_domains:SSF48371,Pfam_domain:PF02259,PTHR11139,PTHR11139:SF66,PROSITE	28.2	probably_damaging	0.999	deleterious	0	65	0.00000
ATM	11	108199845	C	G	rs370559102,CM092586,COSM22476	NO	missense	ENST00000278616.4:c.7187C>G	ENSP00000278616.4:p.Thr2396Ser	T2396S	Superfamily_domains:SSF48371,Pfam_domain:PF02259,PTHR11139,PTHR11139:SF66,PROSITE	14.35	benign	0.044	tolerated	0.61	0	0.00051
ATM	11	108199875	A	G	rs771994581	NO	missense	ENST00000278616.4:c.7217A>G	ENSP00000278616.4:p.Lys2406Arg	K2406R	Superfamily_domains:SSF48371,Pfam_domain:PF02259,PTHR11139,PTHR11139:SF66,PROSITE	27.2	possibly_damaging	0.897	deleterious	0.01	25	0.00003
ATM	11	108199927	A	T	COSM1350979,COSM1350978	NO	missense	ENST00000278616.4:c.7269A>T	ENSP00000278616.4:p.Glu2423Asp	E2423D	Superfamily_domains:SSF48371,Pfam_domain:PF02259,PTHR11139,PTHR11139:SF66,PROSITE	24	possibly_damaging	0.672	tolerated	0.12	0	0.00000
ATM	11	108199929	T	G	CM960103,rs28904921,COSM22477	NO	missense	ENST00000278616.4:c.7271T>G	ENSP00000278616.4:p.Val2424Gly	V2424G	Superfamily_domains:SSF48371,Pfam_domain:PF02259,PTHR11139,PTHR11139:SF66,PROSITE	28.6	probably_damaging	0.974	deleterious	0	65	0.00009
ATM	11	108199936	C	T	CD961810	NO	synonymous	ENST00000278616.4:c.7278C>T	NA	L2426L	Superfamily_domains:SSF48371,Pfam_domain:PF02259,PTHR11139,PTHR11139:SF66,PROSITE	14.25	NA	NA	NA	NA	NA	0.00000
ATM	11	108199949	A	G	CM035636	NO	missense	ENST00000278616.4:c.7291A>G	ENSP00000278616.4:p.Lys2431Glu	K2431E	Superfamily_domains:SSF48371,Pfam_domain:PF02259,PTHR11139,PTHR11139:SF66,PROSITE	22.7	benign	0.077	tolerated	0.11	0	0.00000
ATM	11	108199952	A	G	rs587781838	NO	missense	ENST00000278616.4:c.7294A>G	ENSP00000278616.4:p.Ile2432Val	I2432V	Superfamily_domains:SSF48371,Pfam_domain:PF02259,PTHR11139,PTHR11139:SF66,PROSITE	7.41	benign	0.004	tolerated	1	0	0.00000
ATM	11	108199952	A	T	rs587781838	NO	missense	ENST00000278616.4:c.7294A>T	ENSP00000278616.4:p.Ile2432Phe	I2432F	Superfamily_domains:SSF48371,Pfam_domain:PF02259,PTHR11139,PTHR11139:SF66,PROSITE	21	benign	0.021	tolerated	0.07	0	0.00000
ATM	11	108200919	T	A	NA	NO	intron	ENST00000278616.4:c.7308-22T>A	NA	NA	NA	4.203	NA	NA	NA	NA	NA	0.00000
ATM	11	108200946	C	T	rs147604227,COSM21923	NO	missense	ENST00000278616.4:c.7313C>T	ENSP00000278616.4:p.Thr2438Ile	T2438I	Superfamily_domains:SSF48371,Pfam_domain:PF02259,PTHR11139,PTHR11139:SF66,PROSITE	23.9	possibly_damaging	0.667	tolerated	0.38	0	0.00000
ATM	11	108200947	A	C	rs199909913,COSM1350990,COSM1350989	NO	synonymous	ENST00000278616.4:c.7314A>C	NA	T2438T	Superfamily_domains:SSF48371,Pfam_domain:PF02259,PTHR11139,PTHR11139:SF66,PROSITE	0.019	NA	NA	NA	NA	NA	0.00009
ATM	11	108200974	G	C	NA	NO	missense	ENST00000278616.4:c.7341G>C	ENSP00000278616.4:p.Leu2447Phe	L2447F	Low_complexity_(Seg):seg,Superfamily_domains:SSF48371,Pfam_domain:PF02259,PTHR11139,P	22.9	benign	0.378	deleterious	0.04	0	0.00000
ATM	11	108200990	C	T	rs755418571,COSM1351001,COSM1351000	NO	missense	ENST00000278616.4:c.7357C>T	ENSP00000278616.4:p.Arg2453Cys	R2453C	Low_complexity_(Seg):seg,Superfamily_domains:SSF48371,Pfam_domain:PF02259,PTHR11139,P	28.5	possibly_damaging	0.773	deleterious	0.03	15	0.00003
ATM	11	108201015	G	A	rs768461085	NO	missense	ENST00000278616.4:c.7382G>A	ENSP00000278616.4:p.Arg2461His	R2461H	Superfamily_domains:SSF48371,Pfam_domain:PF02259,PTHR11139,PTHR11139:SF66,PROSITE	25.1	benign	0.275	deleterious	0.04	0	0.00003
ATM	11	108201023	T	C	CM016183,rs55801750,COSM758329	NO	missense	ENST00000278616.4:c.7390T>C	ENSP00000278616.4:p.Cys2464Arg	C2464R	Superfamily_domains:SSF48371,Pfam_domain:PF02259,PTHR11139,PTHR11139:SF66,PROSITE	21.9	possibly_damaging	0.53	tolerated	0.83	0	0.00204
ATM	11	108201070	A	G	NA	NO	synonymous	ENST00000278616.4:c.7437A>G	NA	E2479E	Superfamily_domains:SSF48371,Pfam_domain:PF02259,PTHR11139,PTHR11139:SF66,PROSITE	9.2	NA	NA	NA	NA	NA	0.00000
ATM	11	108201100	C	G	rs767846264	NO	synonymous	ENST00000278616.4:c.7467C>G	NA	S2489S	Superfamily_domains:SSF48371,PTHR11139,PTHR11139:SF66,PROSITE_profiles:PS51189	13.61	NA	NA	NA	NA	NA	0.00000
ATM	11	108201108	T	G	rs56399857	NO	missense	ENST00000278616.4:c.7475T>G	ENSP00000278616.4:p.Leu2492Arg	L2492R	Superfamily_domains:SSF48371,PTHR11139,PTHR11139:SF66,PROSITE_profiles:PS51189	28.3	probably_damaging	0.997	deleterious	0	45	0.00021
ATM	11	108202176	C	T	rs751234924	NO	synonymous	ENST00000278616.4:c.7521C>T	NA	D2507D	PTHR11139,PTHR11139:SF66,PROSITE_profiles:PS51189	15.49	NA	NA	NA	NA	NA	0.00003
ATM	11	108202177	G	A	rs754395517	NO	missense	ENST00000278616.4:c.7522G>A	ENSP00000278616.4:p.Gly2508Arg	G2508R	PTHR11139,PTHR11139:SF66,PROSITE_profiles:PS51189	23.9	benign	0.335	tolerated	0.35	0	0.00003
ATM	11	108202197	T	C	CM980151,rs777925486,CM980150	NO	synonymous	ENST00000278616.4:c.7542T>C	NA	Y2514Y	PTHR11139,PTHR11139:SF66,PROSITE_profiles:PS51189	0.508	NA	NA	NA	NA	NA	0.00003
ATM	11	108202243	A	T	NA	YES	stop_gained	ENST00000278616.4:c.7588A>T	ENSP00000278616.4:p.Lys2530Ter	K2530*	PTHR11139,PTHR11139:SF66,PROSITE_profiles:PS51189	48	NA	NA	NA	NA	NA	0.00000
ATM	11	108202273	G	A	rs35203200	NO	missense	ENST00000278616.4:c.7618G>A	ENSP00000278616.4:p.Val2540Ile	V2540I	PTHR11139,PTHR11139:SF66,PROSITE_profiles:PS51189	20.6	benign	0.008	tolerated	0.33	0	0.00018
ATM	11	108202275	C	A	COSM3868370,COSM3868369	NO	synonymous	ENST00000278616.4:c.7620C>A	NA	V2540V	PTHR11139,PTHR11139:SF66,PROSITE_profiles:PS51189	18.08	NA	NA	NA	NA	NA	0.00000
ATM	11	108202553	T	G	NA	NO	intron	ENST00000278616.4:c.7630-53T>G	NA	NA	NA	2.894	NA	NA	NA	NA	NA	0.00000
ATM	11	108202572	T	C	rs747021157	NO	intron	ENST00000278616.4:c.7630-34T>C	NA	NA	NA	4.409	NA	NA	NA	NA	NA	0.00000
ATM	11	108202589	T	C	rs116047570	NO	intron	ENST00000278616.4:c.7630-17T>C	NA	NA	NA	12.24	NA	NA	NA	NA	NA	0.00000
ATM	11	108202591	G	T	rs773603597	NO	intron	ENST00000278616.4:c.7630-15G>T	NA	NA	NA	10.96	NA	NA	NA	NA	NA	0.00000
ATM	11	108202595	T	C	NA	NO	intron	ENST00000278616.4:c.7630-11T>C	NA	NA	NA	15.67	NA	NA	NA	NA	NA	0.00000
ATM	11	108202603	C	G	rs587782448	NO	splice,intron	ENST00000278616.4:c.7630-3C>G	NA	NA	NA	14.98	NA	NA	NA	NA	NA	0.00000
ATM	11	108202611	CTCTAGAATT	C	rs587776547,COSM22478	NO	inframe_deletion	ENST00000278616.4:c.7638_7646delTAGAATTTC	ENSP00000278616.4:p.Arg2547_Ser2549del	SRI2546-2548-	PTHR11139,PTHR11139:SF66,PROSITE_profiles:PS51189	22.4	NA	NA	NA	NA	NA	0.00000
ATM	11	108202624	A	G	NA	NO	missense	ENST00000278616.4:c.7648A>G	ENSP00000278616.4:p.Met2550Val	M2550V	PTHR11139,PTHR11139:SF66,PROSITE_profiles:PS51189	8.279	benign	0.001	tolerated	0.7	0	0.00000
ATM	11	108202751	C	G	rs755009196,CM024739	NO	missense	ENST00000278616.4:c.7775C>G	ENSP00000278616.4:p.Ser2592Cys	S2592C	PTHR11139,PTHR11139:SF66	26.5	probably_damaging	0.94	deleterious	0	65	0.00009
ATM	11	108202753	C	T	rs781215442,COSM1351007,COSM24631,COS	YES	stop_gained	ENST00000278616.4:c.7777C>T	ENSP00000278616.4:p.Gln2593Ter	Q2593*	PTHR11139,PTHR11139:SF66	45	NA	NA	NA	NA	NA	0.00003
ATM	11	108202754	A	G	rs587779867,COSM1351009,COSM1351008	NO	missense	ENST00000278616.4:c.7778A>G	ENSP00000278616.4:p.Gln2593Arg	Q2593R	PTHR11139,PTHR11139:SF66	11.69	benign	0.042	tolerated	0.42	0	0.00021
ATM	11	108202765	G	C	NA	YES	splice_donor	ENST00000278616.4:c.7788+1G>C	NA	NA	NA	24.8	NA	NA	NA	NA	NA	0.00000
ATM	11	108202772	G	T	CS014123,rs112775908	NO	splice,intron	ENST00000278616.4:c.7788+8G>T	NA	NA	NA	8.104	NA	NA	NA	NA	NA	0.00000
ATM	11	108203437	G	A	NA	NO	intron	ENST00000278616.4:c.7789-52G>A	NA	NA	NA	3.293	NA	NA	NA	NA	NA	0.00000
ATM	11	108203445	G	A	NA	NO	intron	ENST00000278616.4:c.7789-44G>A	NA	NA	NA	5.034	NA	NA	NA	NA	NA	0.00000
ATM	11	108203466	T	A	rs751393915	NO	intron	ENST00000278616.4:c.7789-23T>A	NA	NA	NA	10.59	NA	NA	NA	NA	NA	0.00000
ATM	11	108203468	T	G	rs371070275	NO	intron	ENST00000278616.4:c.7789-21T>G	NA	NA	NA	5.471	NA	NA	NA	NA	NA	0.00000
ATM	11	108203486	T	G	CS961481	NO	splice,intron	ENST00000278616.4:c.7789-3T>G	NA	NA	NA	12.52	NA	NA	NA	NA	NA	0.00000
ATM	11	108203549	G	T	NA	NO	missense	ENST00000278616.4:c.7849G>T	ENSP00000278616.4:p.Val2617Phe	V2617F	3.30.1010.10,PTHR11139,PTHR11139:SF66	23.5	benign	0.129	deleterious	0	0	0.00000
ATM	11	108203579	T	C	NA	NO	missense	ENST00000278616.4:c.7879T>C	ENSP00000278616.4:p.Tyr2627His	Y2627H	Superfamily_domains:SSF56112,3.30.1010.10,PTHR11139,PTHR11139:SF66	28.6	probably_damaging	0.995	deleterious	0	65	0.00000
ATM	11	108203619	C	T	rs4988125	NO	missense	ENST00000278616.4:c.7919C>T	ENSP00000278616.4:p.Thr2640Ile	T2640I	Superfamily_domains:SSF56112,3.30.1010.10,PTHR11139,PTHR11139:SF66	21.4	benign	0.058	tolerated	0.06	0	0.00027
ATM	11	108203637	T	C	rs730881277	NO	intron	ENST00000278616.4:c.7927+10T>C	NA	NA	NA	7.452	NA	NA	NA	NA	NA	0.00000
ATM	11	108203640	T	A	rs748853277	NO	intron	ENST00000278616.4:c.7927+13T>A	NA	NA	NA	4.792	NA	NA	NA	NA	NA	0.00000
ATM	11	108203651	C	T	rs759278155	NO	intron	ENST00000278616.4:c.7927+24C>T	NA	NA	NA	1.151	NA	NA	NA	NA	NA	0.00000
ATM	11	108204518	C	A	rs3218704	NO	intron	ENST00000278616.4:c.7928-95C>A	NA	NA	NA	8.493	NA	NA	NA	NA	NA	0.00000
ATM	11	108204528	C	T	NA	NO	intron	ENST00000278616.4:c.7928-85C>T	NA	NA	NA	7.107	NA	NA	NA	NA	NA	0.00000
ATM	11	108204540	G	A	rs149047841	NO	intron	ENST00000278616.4:c.7928-73G>A	NA	NA	NA	3.431	NA	NA	NA	NA	NA	0.00000
ATM	11	108204557	GACCTTC	G	TMP_ESP_11_108204558_108204563	NO	intron,feature_truncation	ENST00000278616.4:c.7928-54_7928-49delCCTTCA	NA	NA	NA	11.53	NA	NA	NA	NA	NA	0.00000
ATM	11	108204559	C	T	NA	NO	intron	ENST00000278616.4:c.7928-54C>T	NA	NA	NA	12.61	NA	NA	NA	NA	NA	0.00000
ATM	11	108204593	C	T	NA	NO	intron	ENST00000278616.4:c.7928-20C>T	NA	NA	NA	7.286	NA	NA	NA	NA	NA	0.00000
ATM	11	108204598	C	CA	NA	NO	intron,feature_elongation	ENST00000278616.4:c.7928-14dupA	NA	NA	NA	9.016	NA	NA	NA	NA	NA	0.00000
ATM	11	108204603	T	C	rs188404773	NO	intron	ENST00000278616.4:c.7928-10T>C	NA	NA	NA	11.03	NA	NA	NA	NA	NA	0.00000
ATM	11	108204608	T	C	NA	NO	splice,intron	ENST00000278616.4:c.7928-5T>C	NA	NA	NA	9.191	NA	NA	NA	NA	NA	0.00000
ATM	11	108204610	C	G	NA	NO	splice,intron	ENST00000278616.4:c.7928-3C>G	NA	NA	NA	12.16	NA	NA	NA	NA	NA	0.00000
ATM	11	108204668	T	C	rs143972422	NO	synonymous	ENST00000278616.4:c.7983T>C	NA	D2661D	Superfamily_domains:SSF56112,3.30.1010.10,PTHR11139,PTHR11139:SF66	6.492	NA	NA	NA	NA	NA	0.00030
ATM	11	108204685	T	C	CM0910526	NO	missense	ENST00000278616.4:c.8000T>C	ENSP00000278616.4:p.Met2667Thr	M2667T	Superfamily_domains:SSF56112,3.30.1010.10,PTHR11139,PTHR11139:SF66	26.3	possibly_damaging	0.601	deleterious	0.05	25	0.00000
ATM	11	108204726	A	C	NA	NO	intron	ENST00000278616.4:c.8010+31A>C	NA	NA	NA	9.523	NA	NA	NA	NA	NA	0.00000
ATM	11	108204726	A	G	NA	NO	intron	ENST00000278616.4:c.8010+31A>G	NA	NA	NA	10.41	NA	NA	NA	NA	NA	0.00000
ATM	11	108204738	T	G	NA	NO	intron	ENST00000278616.4:c.8010+43T>G	NA	NA	NA	3.317	NA	NA	NA	NA	NA	0.00000
ATM	11	108204740	T	C	NA	NO	intron	ENST00000278616.4:c.8010+45T>C	NA	NA	NA	8.581	NA	NA	NA	NA	NA	0.00000
ATM	11	108205690	T	G	NA	NO	splice,intron	ENST00000278616.4:c.8011-6T>G	NA	NA	NA	4.844	NA	NA	NA	NA	NA	0.00000
ATM	11	108205700	A	C	rs763161651	NO	missense	ENST00000278616.4:c.8015A>C	ENSP00000278616.4:p.Asp2672Ala	D2672A	Superfamily_domains:SSF56112,3.30.1010.10,PTHR11139,PTHR11139:SF66	29.3	probably_damaging	0.949	deleterious	0.01	65	0.00003
ATM	11	108205704	CA	C	NA	YES	frameshift,feature_truncation	ENST00000278616.4:c.8020delA	ENSP00000278616.4:p.Thr2674GlnfsTer8	T2674X	Superfamily_domains:SSF56112,3.30.1010.10,PTHR11139,PTHR11139:SF66	36	NA	NA	NA	NA	NA	0.00000
ATM	11	108205713	A	G	NA	NO	synonymous	ENST00000278616.4:c.8028A>G	NA	E2676E	Superfamily_domains:SSF56112,3.30.1010.10,PTHR11139,PTHR11139:SF66	6.69	NA	NA	NA	NA	NA	0.00000
ATM	11	108205729	A	C	rs730881320	NO	missense	ENST00000278616.4:c.8044A>C	ENSP00000278616.4:p.Thr2682Pro	T2682P	Superfamily_domains:SSF56112,3.30.1010.10,PTHR11139,PTHR11139:SF66	24.3	probably_damaging	0.963	tolerated	0.22	0	0.00000
ATM	11	108205732	A	G	rs587781344	NO	missense	ENST00000278616.4:c.8047A>G	ENSP00000278616.4:p.Ile2683Val	I2683V	Superfamily_domains:SSF56112,3.30.1010.10,PTHR11139,PTHR11139:SF66	2.856	benign	0.003	tolerated	0.58	0	0.00000
ATM	11	108205736	A	G	NA	NO	missense	ENST00000278616.4:c.8051A>G	ENSP00000278616.4:p.Gln2684Arg	Q2684R	Superfamily_domains:SSF56112,3.30.1010.10,PTHR11139,PTHR11139:SF66	0.585	benign	0	tolerated	1	0	0.00000
ATM	11	108205767	A	C	rs540266635	NO	synonymous	ENST00000278616.4:c.8082A>C	NA	G2694G	Superfamily_domains:SSF56112,3.30.1010.10,PTHR11139,PTHR11139:SF66	6.184	NA	NA	NA	NA	NA	0.00000
ATM	11	108205768	G	C	COSM540828,COSM540829,COSM1139596,C	NO	missense	ENST00000278616.4:c.8083G>C	ENSP00000278616.4:p.Gly2695Arg	G2695R	Superfamily_domains:SSF56112,3.30.1010.10,PTHR11139,PTHR11139:SF66	33	probably_damaging	1	deleterious	0	65	0.00000
ATM	11	108205807	G	A	rs587782719,CM063855,COSM48246	NO	missense	ENST00000278616.4:c.8122G>A	ENSP00000278616.4:p.Asp2708Asn	D2708N	Superfamily_domains:SSF56112,3.30.1010.10,PTHR11139,PTHR11139:SF66	33	probably_damaging	0.953	deleterious	0	15	0.00000
ATM	11	108205847	C	T	rs555381912	NO	intron	ENST00000278616.4:c.8151+11C>T	NA	NA	NA	5.398	NA	NA	NA	NA	NA	0.00000
ATM	11	108206505	C	CAGATA	rs576179904	NO	intron,feature_elongation	ENST00000278616.4:c.8152-63_8152-62insAAGAT	NA	NA	NA	6.202	NA	NA	NA	NA	NA	0.00000
ATM	11	108206518	G	A	NA	NO	intron	ENST00000278616.4:c.8152-54G>A	NA	NA	NA	10.04	NA	NA	NA	NA	NA	0.00000
ATM	11	108206526	A	G	rs373054235	NO	intron	ENST00000278616.4:c.8152-46A>G	NA	NA	NA	6.424	NA	NA	NA	NA	NA	0.00000
ATM	11	108206545	G	A	NA	NO	intron	ENST00000278616.4:c.8152-27G>A	NA	NA	NA	4.201	NA	NA	NA	NA	NA	0.00000
ATM	11	108206575	C	T	rs138526014	NO	missense	ENST00000278616.4:c.8155C>T	ENSP00000278616.4:p.Arg2719Cys	R2719C	Superfamily_domains:SSF56112,SMART_domains:SM00146,3.30.1010.10,Pfam_domain:PF00454	35	probably_damaging	0.977	deleterious	0	35	0.00003
ATM	11	108206576	G	A	CS081865,CM0910527,rs55982963	NO	missense	ENST00000278616.4:c.8156G>A	ENSP00000278616.4:p.Arg2719His	R2719H	Superfamily_domains:SSF56112,SMART_domains:SM00146,3.30.1010.10,Pfam_domain:PF00454	34	probably_damaging	0.967	deleterious	0.02	0	0.00022
ATM	11	108206594	A	G	COSM21882,COSM22481,COSM3358963	NO	missense	ENST00000278616.4:c.8174A>G	ENSP00000278616.4:p.Asp2725Gly	D2725G	Superfamily_domains:SSF56112,SMART_domains:SM00146,3.30.1010.10,Pfam_domain:PF00454	31	probably_damaging	0.995	deleterious	0.01	65	0.00000
ATM	11	108206614	T	G	COSM1315818,COSM22482	NO	missense	ENST00000278616.4:c.8194T>G	ENSP00000278616.4:p.Phe2732Val	F2732V	Superfamily_domains:SSF56112,SMART_domains:SM00146,3.30.1010.10,Pfam_domain:PF00454	31	probably_damaging	0.987	deleterious	0	45	0.00000
ATM	11	108206620	A	T	NA	NO	missense	ENST00000278616.4:c.8200A>T	ENSP00000278616.4:p.Met2734Leu	M2734L	Superfamily_domains:SSF56112,SMART_domains:SM00146,3.30.1010.10,Pfam_domain:PF00454	15.44	benign	0.004	tolerated	0.48	0	0.00000

ATM	11	108206683	TATAAG	T	rs730881294	YES	frameshift,splice,feature_truncation	ENST00000278616.4:c.8264_8268delATAAG	ENSP00000278616.4:p.Tyr2755CysfsTer12	YK2755-2756X	Superfamily_domains:SSF56112,SMART_domains:SM00146,3.30.1010.10,Pfam_domain:PF00454	36	NA	NA	NA	NA	NA	0.00000
ATM	11	108206686	A	T	rs371638537,CM960108,COSM758333	YES	stop_gained,splice	ENST00000278616.4:c.8266A>T	ENSP00000278616.4:p.Lys2756Ter	K2756*	Superfamily_domains:SSF56112,SMART_domains:SM00146,3.30.1010.10,Pfam_domain:PF00454	54	NA	NA	NA	NA	NA	0.00003
ATM	11	108206694	T	A	rs747153940	NO	splice,intron	ENST00000278616.4:c.8268+6T>A	NA	NA	NA	6.482	NA	NA	NA	NA	NA	0.00000
ATM	11	108213935	A	T	rs114320959	NO	intron	ENST00000278616.4:c.8269-14A>T	NA	NA	NA	3.089	NA	NA	NA	NA	NA	0.00000
ATM	11	108213949	G	A	rs761625350	NO	missense,splice	ENST00000278616.4:c.8269G>A	ENSP00000278616.4:p.Val2757Met	V2757M	Superfamily_domains:SSF56112,SMART_domains:SM00146,3.30.1010.10,Pfam_domain:PF00454	24.9	probably_damaging	0.954	tolerated	0.05	15	0.00000
ATM	11	108213952	G	T	NA	NO	missense	ENST00000278616.4:c.8272G>T	ENSP00000278616.4:p.Val2758Phe	V2758F	Superfamily_domains:SSF56112,SMART_domains:SM00146,3.30.1010.10,Pfam_domain:PF00454	29.2	probably_damaging	0.977	deleterious	0	0	0.00000
ATM	11	108213967	C	T	CM080077,COSM1183943,COSM1183942	YES	stop_gained	ENST00000278616.4:c.8287C>T	ENSP00000278616.4:p.Arg2763Ter	R2763*	Superfamily_domains:SSF56112,SMART_domains:SM00146,1.10.1070.11,Pfam_domain:PF00454	49	NA	NA	NA	NA	NA	0.00000
ATM	11	108213987	G	A	rs778269655,CM960109,COSM265664	YES	stop_gained	ENST00000278616.4:c.8307G>A	ENSP00000278616.4:p.Trp2769Ter	W2769*	Superfamily_domains:SSF56112,SMART_domains:SM00146,1.10.1070.11,Pfam_domain:PF00454	51	NA	NA	NA	NA	NA	0.00000
ATM	11	108214001	TC	T	NA	YES	frameshift,feature_truncation	ENST00000278616.4:c.8325delC	ENSP00000278616.4:p.Ile2776LeufsTer30	V2774X	Superfamily_domains:SSF56112,SMART_domains:SM00146,1.10.1070.11,Pfam_domain:PF00454	35	NA	NA	NA	NA	NA	0.00000
ATM	11	108214007	T	C	rs746475628	NO	missense	ENST00000278616.4:c.8327T>C	ENSP00000278616.4:p.Ile2776Thr	I2776T	Superfamily_domains:SSF56112,SMART_domains:SM00146,1.10.1070.11,Pfam_domain:PF00454	27.9	probably_damaging	0.965	deleterious	0	25	0.00009
ATM	11	108214098	GGTGA	G	rs769139997	YES	splice_donor,intron,feature_truncation	ENST00000278616.4:c.8418+5_8418+8delGTGA	NA	NA	NA	27.3	NA	NA	NA	NA	NA	0.00003
ATM	11	108214132	G	C	rs764846903	NO	intron	ENST00000278616.4:c.8418+34G>C	NA	NA	NA	1.267	NA	NA	NA	NA	NA	0.00000
ATM	11	108216545	C	T	rs587779872,CM980159,COSM22488,COSM3	NO	missense	ENST00000278616.4:c.8494C>T	ENSP00000278616.4:p.Arg2832Cys	R2832C	Superfamily_domains:SSF56112,SMART_domains:SM00146,Pfam_domain:PF00454,PTHR11139,	35	probably_damaging	0.993	deleterious	0	45	0.00003
ATM	11	108216546	G	A	rs529296539	NO	missense	ENST00000278616.4:c.8495G>A	ENSP00000278616.4:p.Arg2832His	R2832H	Superfamily_domains:SSF56112,SMART_domains:SM00146,Pfam_domain:PF00454,PTHR11139,	24	benign	0.183	tolerated	0.36	0	0.00015
ATM	11	108216597	G	A	rs587782202,CM990219	NO	missense	ENST00000278616.4:c.8546G>A	ENSP00000278616.4:p.Arg2849Gln	R2849Q	Superfamily_domains:SSF56112,SMART_domains:SM00146,1.10.1070.11,Pfam_domain:PF00454	35	probably_damaging	0.998	deleterious	0	35	0.00000
ATM	11	108216609	C	T	rs141534716,COSM3358965,COSM922753,CO	NO	missense	ENST00000278616.4:c.8558C>T	ENSP00000278616.4:p.Thr2853Met	T2853M	Superfamily_domains:SSF56112,SMART_domains:SM00146,1.10.1070.11,Pfam_domain:PF00454	34	probably_damaging	0.999	deleterious	0	65	0.00015
ATM	11	108216610	G	A	rs368058202	NO	synonymous	ENST00000278616.4:c.8559G>A	NA	T2853T	Superfamily_domains:SSF56112,SMART_domains:SM00146,1.10.1070.11,Pfam_domain:PF00454	14.34	NA	NA	NA	NA	NA	0.00000
ATM	11	108216611	C	T	rs201958469,CM092588	NO	missense	ENST00000278616.4:c.8560C>T	ENSP00000278616.4:p.Arg2854Cys	R2854C	Superfamily_domains:SSF56112,SMART_domains:SM00146,1.10.1070.11,Pfam_domain:PF00454	35	probably_damaging	0.995	deleterious	0	35	0.00039
ATM	11	108216623	ACTT	A	NA	NO	inframe_deletion	ENST00000278616.4:c.8578_8580delTCT	ENSP00000278616.4:p.Ser2860del	TS2858-2859T	Superfamily_domains:SSF56112,SMART_domains:SM00146,1.10.1070.11,Pfam_domain:PF00454	22.6	NA	NA	NA	NA	NA	0.00000
ATM	11	108216625	T	C	NA	NO	synonymous	ENST00000278616.4:c.8574T>C	NA	T2858T	Superfamily_domains:SSF56112,SMART_domains:SM00146,1.10.1070.11,Pfam_domain:PF00454	2.414	NA	NA	NA	NA	NA	0.00000
ATM	11	108216628	T	C	COSM1351033,COSM1351032	NO	synonymous	ENST00000278616.4:c.8577T>C	NA	S2859S	Superfamily_domains:SSF56112,SMART_domains:SM00146,1.10.1070.11,Pfam_domain:PF00454	2	NA	NA	NA	NA	NA	0.00000
ATM	11	108216639	A	G	NA	NO	splice,intron	ENST00000278616.4:c.8584+4A>G	NA	NA	NA	15.2	NA	NA	NA	NA	NA	0.00000
ATM	11	108218002	C	A	NA	NO	splice,intron	ENST00000278616.4:c.8585-4C>A	NA	NA	NA	7.693	NA	NA	NA	NA	NA	0.00000
ATM	11	108218013	C	T	rs56025670	NO	synonymous	ENST00000278616.4:c.8592C>T	NA	Y2864Y	Superfamily_domains:SSF56112,SMART_domains:SM00146,1.10.1070.11,Pfam_domain:PF00454	9.667	NA	NA	NA	NA	NA	0.00140
ATM	11	108218017	C	G	rs368666328,COSM922754,COSM1585328	NO	missense	ENST00000278616.4:c.8596C>G	ENSP00000278616.4:p.Leu2866Val	L2866V	Superfamily_domains:SSF56112,SMART_domains:SM00146,1.10.1070.11,Pfam_domain:PF00454	16.67	benign	0.371	tolerated	0.84	0	0.00000
ATM	11	108218020	G	T	CM960111	YES	stop_gained	ENST00000278616.4:c.8599G>T	ENSP00000278616.4:p.Gly2867Ter	G2867*	Superfamily_domains:SSF56112,SMART_domains:SM00146,1.10.1070.11,Pfam_domain:PF00454	55	NA	NA	NA	NA	NA	0.00000
ATM	11	108218101	T	G	rs200190537	NO	intron	ENST00000278616.4:c.8671+9T>G	NA	NA	NA	5.503	NA	NA	NA	NA	NA	0.00000
ATM	11	108218126	A	G	NA	NO	intron	ENST00000278616.4:c.8671+34A>G	NA	NA	NA	16.68	NA	NA	NA	NA	NA	0.00000
ATM	11	108224433	A	G	rs761306969	NO	intron	ENST00000278616.4:c.8672-60A>G	NA	NA	NA	2.49	NA	NA	NA	NA	NA	0.00000
ATM	11	108224493	G	A	rs748192003,CM0910529,COSM758347	NO	missense,splice	ENST00000278616.4:c.8672G>A	ENSP00000278616.4:p.Gly2891Asp	G2891D	Superfamily_domains:SSF56112,SMART_domains:SM00146,1.10.1070.11,Pfam_domain:PF00454	32	probably_damaging	1	deleterious	0	65	0.00000
ATM	11	108224527	T	C	NA	NO	synonymous	ENST00000278616.4:c.8706T>C	NA	T2902T	Superfamily_domains:SSF56112,SMART_domains:SM00146,1.10.1070.11,Pfam_domain:PF00454	7.455	NA	NA	NA	NA	NA	0.00000
ATM	11	108224555	A	G	rs376676328,CM014034	NO	missense	ENST00000278616.4:c.8734A>G	ENSP00000278616.4:p.Arg2912Gly	R2912G	Superfamily_domains:SSF56112,SMART_domains:SM00146,1.10.1070.11,Pfam_domain:PF00454	29.3	probably_damaging	0.999	deleterious	0	65	0.00099
ATM	11	108224558	G	T	rs756899044	NO	missense	ENST00000278616.4:c.8737G>T	ENSP00000278616.4:p.Asp2913Tyr	D2913Y	Superfamily_domains:SSF56112,SMART_domains:SM00146,1.10.1070.11,Pfam_domain:PF00454	33	probably_damaging	1	deleterious	0	65	0.00000
ATM	11	108224593	A	G	NA	NO	synonymous	ENST00000278616.4:c.8772A>G	NA	E2924E	Superfamily_domains:SSF56112,SMART_domains:SM00146,1.10.1070.11,Pfam_domain:PF00454	8.984	NA	NA	NA	NA	NA	0.00000
ATM	11	108224608	G	A	CS961484,COSM21633,COSM1351047,COSM	YES	splice_donor	ENST00000278616.4:c.8786+1G>A	NA	NA	NA	26.3	NA	NA	NA	NA	NA	0.00006
ATM	11	108224615	A	C	rs4986839	NO	splice,intron	ENST00000278616.4:c.8786+8A>C	NA	NA	NA	1.963	NA	NA	NA	NA	NA	0.00000
ATM	11	108224622	G	A	NA	NO	intron	ENST00000278616.4:c.8786+15G>A	NA	NA	NA	5.042	NA	NA	NA	NA	NA	0.00000
ATM	11	108224632	G	A	rs764861531	NO	intron	ENST00000278616.4:c.8786+25G>A	NA	NA	NA	5.642	NA	NA	NA	NA	NA	0.00000
ATM	11	108224639	T	C	NA	NO	intron	ENST00000278616.4:c.8786+32T>C	NA	NA	NA	1.47	NA	NA	NA	NA	NA	0.00000
ATM	11	108224644	G	A	NA	NO	intron	ENST00000278616.4:c.8786+37G>A	NA	NA	NA	5.258	NA	NA	NA	NA	NA	0.00000
ATM	11	108225473	G	A	rs139490310	NO	intron	ENST00000278616.4:c.8787-65G>A	NA	NA	NA	2.938	NA	NA	NA	NA	NA	0.00000
ATM	11	108225477	A	AAGAT	rs775653463	NO	intron,feature_elongation	ENST00000278616.4:c.8787-59_8787-56dupGATA	NA	NA	NA	0.933	NA	NA	NA	NA	NA	0.00000
ATM	11	108225483	C	T	rs664982	NO	intron	ENST00000278616.4:c.8787-55C>T	NA	NA	NA	2.77	NA	NA	NA	NA	NA	0.00000
ATM	11	108225489	CA	C	NA	NO	intron,feature_truncation	ENST00000278616.4:c.8787-47delA	NA	NA	NA	8.501	NA	NA	NA	NA	NA	0.00000
ATM	11	108225492	C	T	NA	NO	intron	ENST00000278616.4:c.8787-46C>T	NA	NA	NA	5.115	NA	NA	NA	NA	NA	0.00000
ATM	11	108225493	A	T	rs751581707	NO	intron	ENST00000278616.4:c.8787-45A>T	NA	NA	NA	1.734	NA	NA	NA	NA	NA	0.00000
ATM	11	108225494	T	C	NA	NO	intron	ENST00000278616.4:c.8787-44T>C	NA	NA	NA	1.616	NA	NA	NA	NA	NA	0.00000
ATM	11	108225512	C	T	rs373681524	NO	intron	ENST00000278616.4:c.8787-26C>T	NA	NA	NA	1.293	NA	NA	NA	NA	NA	0.00000
ATM	11	108225605	A	C	rs587782335	NO	splice,intron	ENST00000278616.4:c.8850+4A>C	NA	NA	NA	13.26	NA	NA	NA	NA	NA	0.00000
ATM	11	108225615	T	C	NA	NO	intron	ENST00000278616.4:c.8850+14T>C	NA	NA	NA	11.08	NA	NA	NA	NA	NA	0.00000
ATM	11	108225642	C	T	rs759533987	NO	intron	ENST00000278616.4:c.8850+41C>T	NA	NA	NA	7.932	NA	NA	NA	NA	NA	0.00000
ATM	11	108225651	T	G	rs758956006	NO	intron	ENST00000278616.4:c.8850+50T>G	NA	NA	NA	6.452	NA	NA	NA	NA	NA	0.00000
ATM	11	108225658	C	G	rs761586242	NO	intron	ENST00000278616.4:c.8850+57C>G	NA	NA	NA	2.57	NA	NA	NA	NA	NA	0.00000
ATM	11	108225659	T	C	rs547667907	NO	intron	ENST00000278616.4:c.8850+58T>C	NA	NA	NA	5.544	NA	NA	NA	NA	NA	0.00000
ATM	11	108225661	A	G	rs664143	NO	intron	ENST00000278616.4:c.8850+60A>G	NA	NA	NA	0.543	NA	NA	NA	NA	NA	0.00000
ATM	11	108235747	G	A	NA	NO	intron	ENST00000278616.4:c.8851-62G>A	NA	NA	NA	7.532	NA	NA	NA	NA	NA	0.00000
ATM	11	108235750	T	A	NA	NO	intron	ENST00000278616.4:c.8851-59T>A	NA	NA	NA	7.225	NA	NA	NA	NA	NA	0.00000
ATM	11	108235751	C	A	NA	NO	intron	ENST00000278616.4:c.8851-58C>A	NA	NA	NA	4.712	NA	NA	NA	NA	NA	0.00000
ATM	11	108235758	T	G	NA	NO	intron	ENST00000278616.4:c.8851-51T>G	NA	NA	NA	2.5	NA	NA	NA	NA	NA	0.00000
ATM	11	108235761	G	A	rs778014061	NO	intron	ENST00000278616.4:c.8851-48G>A	NA	NA	NA	3.579	NA	NA	NA	NA	NA	0.00000
ATM	11	108235776	T	G	rs746011368	NO	intron	ENST00000278616.4:c.8851-33T>G	NA	NA	NA	4.866	NA	NA	NA	NA	NA	0.00000
ATM	11	108235806	T	G	rs748874219	NO	splice,intron	ENST00000278616.4:c.8851-3T>G	NA	NA	NA	12.98	NA	NA	NA	NA	NA	0.00000
ATM	11	108235818	T	C	rs371619067,COSM1645451,COSM1351049,C	NO	missense	ENST00000278616.4:c.8860T>C	ENSP00000278616.4:p.Tyr2954His	Y2954H	Superfamily_domains:SSF56112,SMART_domains:SM00146,1.10.1070.11,Pfam_domain:PF00454	28	probably_damaging	0.954	deleterious	0	65	0.00003
ATM	11	108235886	T	G	NA	NO	missense	ENST00000278616.4:c.8928T>G	ENSP00000278616.4:p.Asp2976Glu	D2976E	SMART_domains:SM00146,PTHR11139,PTHR11139:SF66,PROSITE_profiles:PS50290	14.9	possibly_damaging	0.459	tolerated	0.09	0	0.00000
ATM	11	108235935	C	T	rs770641163,CM960113,COSM428365,COSM1	YES	stop_gained	ENST00000278616.4:c.8977C>T	ENSP00000278616.4:p.Arg2993Ter	R2993*	SMART_domains:SM00146,PTHR11139,PTHR11139:SF66,PROSITE_profiles:PS50290	47	NA	NA	NA	NA	NA	0.00006
ATM	11	108235937	A	G	NA	NO	synonymous	ENST00000278616.4:c.8979A>G	NA	R2993R	SMART_domains:SM00146,PTHR11139,PTHR11139:SF66,PROSITE_profiles:PS50290	7.207	NA	NA	NA	NA	NA	0.00000
ATM	11	108235941	C	T	rs142322668	NO	missense	ENST00000278616.4:c.8983C>T	ENSP00000278616.4:p.Leu2995Phe	L2995F	SMART_domains:SM00146,PTHR11139,PTHR11139:SF66,PROSITE_profiles:PS50290	16.24	benign	0.187	tolerated	0.57	0	0.00000
ATM	11	108235955	A	G	NA	NO	intron	ENST00000278616.4:c.8987+10A>G	NA	NA	NA	1.668	NA	NA	NA	NA	NA	0.00000
ATM	11	108235995	A	T	NA	NO	intron	ENST00000278616.4:c.8987+50A>T	NA	NA	NA	1.806	NA	NA	NA	NA	NA	0.00000
ATM	11	108236010	A	G	rs758662688	NO	intron	ENST00000278616.4:c.8988-42A>G	NA	NA	NA	2.138	NA	NA	NA	NA	NA	0.00000
ATM	11	108236086	C	T	CM000656,rs587782292,COSM1561110,COSM	NO	missense	ENST00000278616.4:c.9022C>T	ENSP00000278616.4:p.Arg3008Cys	R3008C	Coiled-coils_(Ncoils):Coil,SMART_domains:SM00146,1.10.1070.11,PTHR11139,PTHR11139:SF6	34	probably_damaging	0.988	deleterious	0	35	0.00003
ATM	11	108236109	G	A	NA	NO	synonymous	ENST00000278616.4:c.9045G>A	NA	E3015E	Coiled-coils_(Ncoils):Coil,SMART_domains:SM00146,1.10.1070.11,PTHR11139,PTHR11139:SF6	9.945	NA	NA	NA	NA	NA	0.00000
ATM	11	108236135	C	T	NA	NO	missense	ENST00000278616.4:c.9071C>T	ENSP00000278616.4:p.Thr3024Ile	T3024I	Coiled-coils_(Ncoils):Coil,1.10.1070.11,PTHR11139,PTHR11139:SF66,PROSITE_profiles:PS5029	22.7	benign	0.098	tolerated	0.27	0	0.00000
ATM	11	108236150	G	A	rs201199629	NO	missense	ENST00000278616.4:c.9086G>A	ENSP00000278616.4:p.Gly3029Asp	G3029D	1.10.1070.11,Pfam_domain:PF02260,PTHR11139,PTHR11139:SF66,PROSITE_profiles:PS50290,F	15.83	benign	0.021	tolerated	0.11	0	0.00051
ATM	11	108236183	T	A	rs369870357	NO	missense	ENST00000278616.4:c.9119T>A	ENSP00000278616.4:p.Ile3040Lys	I3040K	1.10.1070.11,Pfam_domain:PF02260,PTHR11139,PTHR11139:SF66,PROSITE_profiles:PS50290,F	24.3	benign	0.038	deleterious	0.02	0	0.00000
ATM	11	108236187	C	T	NA	NO	synonymous	ENST00000278616.4:c.9123C>T	NA	D3041D	1.10.1070.11,Pfam_domain:PF02260,PTHR11139,PTHR11139:SF66,PROSITE_profiles:PS50290,F	13.23	NA	NA	NA	NA	NA	0.00000
ATM	11	108236203	C	T	CM960114,rs121434219,COSM21624,COSM11	YES	stop_gained	ENST00000278616.4:c.9139C>T	ENSP00000278616.4:p.Arg3047Ter	R3047*	Pfam_domain:PF02260,PTHR11139,PTHR11139:SF66,PROSITE_profiles:PS50290,PROSITE_pro	49	NA	NA	NA	NA	NA	0.00000
CHEK2	22	29083867	G	A	rs17884403	NO	3_prime_UTR	ENST00000328354.6:c.*18C>T	NA	NA	NA	6.344	NA	NA	NA	NA	NA	0.00000
CHEK2	22	29083878	A	G	rs121908710	NO	3_prime_UTR	ENST00000328354.6:c.*7T>C	NA	NA	NA	6.767	NA	NA	NA	NA	NA	0.00000
CHEK2	22	29083880	G	A	rs587781367	NO	3_prime_UTR	ENST00000328354.6:c.*5C>T	NA	NA	NA	0.888	NA	NA	NA	NA	NA	0.00000
CHEK2	22	29083894	A	G	rs773670297	NO	synonymous	ENST00000328354.6:c.1623T>C	NA	A541A	NA	0.483	NA	NA	NA	NA	NA	0.00000
CHEK2	22	29083924	C	T	rs758555487	NO	synonymous	ENST00000328354.6:c.1593G>A	NA	E531E	PTHR24344:SF21,PTHR24344	10.19	NA	NA	NA	NA	NA	0.00000
CHEK2	22	29083932	C	T	NA	NO	missense	ENST00000328354.6:c.1585G>A	ENSP00000329178.6:p.Gly529Ser	G529S	PTHR24344:SF21,PTHR24344	10.95	benign	0.004	tolerated	0.4	0	0.00000
CHEK2	22	29083961	C	A	rs587780180,COSM1327286	NO	missense	ENST00000328354.6:c.1556G>T	ENSP00000329178.6:p.Arg519Leu	R519L	PTHR24344:SF21,PTHR24344	24.9	benign	0.33	deleterious	0.01	0	0.00054
CHEK2	22	29083961	C	T	rs587780180,COSM1327286	NO	missense	ENST00000328354.6:c.1556G>A	ENSP00000329178.6:p.Arg519Gln	R519Q	PTHR24344:SF21,PTHR24344	23.1	benign	0.036	tolerated	0.09	0	0.00003
CHEK2	22	29085107	T	C	rs749728970	NO	intron	ENST00000328354.6:c.1542+16A>G	NA	NA	NA	1.213	NA	NA	NA	NA	NA	0.00000
CHEK2	22	29085112	A	T	rs17881716	NO	intron	ENST00000328354.6:c.1542+11T>A	NA	NA	NA	3.48	NA	NA	NA	NA	NA	0.00000
CHEK2	22	29085139	G	A	rs587782541	NO	missense	ENST00000328354.6:c.1526C>T	ENSP00000329178.6:p.Pro509Leu	P509L	PTHR24344:SF21,PTHR24344	10.4	benign	0.003	tolerated	0.1	0	0.00000
CHEK2	22	29090098	G	C	NA	NO	missense	ENST00000328354.6:c.1383C>G	ENSP00000329178.6:p.Asp461Glu	D461E	Low_complexity_(Seg):seg,PROSITE_profiles:PS50011,PTHR24344:SF21,PTHR24344,1.10.510.1	22.1	benign	0.215	tolerated	0.05	35	0.00000
CHEK2	22	29091037	G	C	rs5762749	NO	intron	ENST00000328354.6:c.1375+78C>G	NA	NA	NA	5.976	NA	NA	NA	NA	NA	0.00000
CHEK2	22	29091068	T	C	NA	NO	intron	ENST00000328354.6:c.1375+47A>G	NA	NA	NA	17.58	NA	NA	NA	NA	NA	0.00000
CHEK2	22	29091089	A	G	rs527418829	NO	intron	ENST00000328354.6:c.1375+26T>C	NA	NA	NA	17.54	NA	NA	NA	NA	NA	0.00000
CHEK2	22	29091098	C	T	NA	NO	intron	ENST00000328354.6:c.1375+17G>A	NA	NA	NA	19.08	NA	NA	NA	NA	NA	0.00000
CHEK2	22	29091127	C	T	NA	NO	missense	ENST00000328354.6:c.1363G>A	ENSP00000329178.6:p.Val455Ile	V455I	PROSITE_profiles:PS50011,PTHR24344:SF21,PTHR24344,1.10.510.10,Pfam_domain:PF00069,S	22.8	benign	0.048	tolerated	0.28	25	0.00000
CHEK2	22	29091142	C	T	NA	NO	missense	ENST00000328354.6:c.1348G>A	ENSP00000329178.6:p.Glu450Lys	E450K	PROSITE_profiles:PS50011,PTHR24344:SF21,PTHR24344,1.10.510.10,Pfam_domain:PF00069,S	20.6	benign	0.001	tolerated	0.69	0	0.00000
CHEK2	22	29091147	A	C	rs17886163	NO	missense	ENST00000328354.6:c.1343T>G	ENSP00000329178.6:p.Ile448Ser	I448S	PROSITE_profiles:PS50011,PTHR24344:SF21,PTHR24344,1.10.510.10,Pfam_domain:PF00069,S	20.7	benign	0.309	tolerated	0.76	15	0.00000
CHEK2	22	29091172	T	G	rs587780176	NO	missense	ENST00000328354.6:c.1318A>C	ENSP00000329178.6:p.Ile440Leu	I440L	PROSITE_profiles:PS50011,PTHR24344:SF21,PTHR24344,1.10.510.10,Pfam_domain:PF00069,S	31	probably_damaging	0.909	deleterious	0.01	0	0.00000
CHEK2	22	29091175	G	A	rs778989252	YES	stop_gained	ENST00000328354.6:c.1315C>T	ENSP00000329178.6:p.Gln439Ter	Q439*	PROSITE_profiles:PS50011,PTHR24344:SF21,PTHR24344,1.10.510.10,Pfam_domain:PF00069,S	46	NA	NA	NA	NA	NA	0.00000
CHEK2	22	29091178	C	A	rs200050883,CM077522	NO	missense	ENST00000328354.6:c.1312G>T	ENSP00000329178.6:p.Asp438Tyr	D438Y	PROSITE_profiles:PS50011,PTHR24344:SF21,PTHR24344,1.10.510.10,Pfam_domain:PF00069,S	33	probably_damaging	0.915	deleterious	0	25	0.00069
CHEK2	22	29091207	G	A	CM051021,rs137853011,COSM2935967	NO	missense	ENST00000328354.6:c.1283C>T	ENSP00000329178.6:p.Ser428Phe	S428F	PROSITE_profiles:PS50011,PTHR24344:SF21,PTHR24344,1.10.510.10,Pfam_domain:PF00069,S	29.8	probably_damaging	0.989	deleterious	0.01	15	0.00111
CHEK2	22	29091220	A	G	rs139366548	NO	missense	ENST00000328354.6:c.1270T>C	ENSP00000329178.6:p.Tyr424His	Y424H	PROSITE_profiles:PS50011,PTHR24344:SF21,PTHR24344,1.10.510.10,Pfam_domain:PF00069,S	28.7	probably_damaging	1	deleterious	0.03	65	0.00100
CHEK2	22	29091225	C	T	rs549755590	NO	missense	ENST00000328354.6:c.1265G>A	ENSP00000329178.6:p.Ser422Asn	S422N	PROSITE_profiles:PS50011,PTHR24344:SF21,PTHR24344,1.10.510.10,Pfam_domain:PF00069,S	26	possibly_damaging	0.66	tolerated	0.06	0	0.00000
CHEK2	22	29091226	TA	T	rs587780174,CD110739	YES	frameshift,feature_truncation	ENST00000328354.6:c.1263delT	ENSP00000329178.6:p.Ser422ValfsTer15	L421X	PROSITE_profiles:PS50011,PTHR24344:SF21,PTHR24344,1.10.510.10,Pfam_domain:PF00069,S	35	NA	NA	NA	NA	NA	0.00016
CHEK2	22	29091682	T	C	rs756361647	NO	intron	ENST00000328354.6:c.1259+16A>G	NA	NA	NA	1.475	NA	NA	NA	NA	NA	0.00000
CHEK2	22	29091684	A	T	rs780245762	NO	intron	ENST00000328354.6:c.1259+14T>A	NA	NA	NA	7.321	NA	NA	NA	NA	NA	0.00000
CHEK2	22	29091690	T	C	rs368388249	NO	splice,intron	ENST00000328354.6:c.1259+8A>G	NA	NA	NA	2.738	NA	NA	NA	NA	NA	0.00000
CHEK2	22	29091714	C	T	NA	NO	missense	ENST00000328354.6:c.1243G>A	ENSP00000329178.6:p.Val415Ile	V415I	PROSITE_profiles:PS50011,PTHR24344:SF21,PTHR24344,1.10.510.10,Pfam_domain:PF00069,S	33	probably_damaging	0.977	deleterious	0.01	25	0.00000
CHEK2	22	29091732	C	A	rs766191039	NO	missense	ENST00000328354.6:c.1225G>T	ENSP00000329178.6:p.Asp409Tyr	D409Y	PROSITE_profiles:PS50011,PTHR24344:SF21,PTHR24344,1.10.510.10,Pfam_domain:PF00069,S	34	probably_damaging	1	deleterious	0	65	0.00000
CHEK2	22	29091741	G	A	rs587782527	NO	missense	ENST00000328354.6:c.1216C>T	ENSP00000329178.6:p.Arg406Cys	R406C	PROSITE_profiles:PS50011,PTHR24344:SF21,PTHR24344,1.10.510.10,Pfam_domain:PF00069,S	26.7	benign	0.251	deleterious	0.05	15	0.00012
CHEK2	22	29091742	G	T	rs587780171	NO	missense	ENST00000328354.6:c.1215C>A	ENSP00000329178.6:p.Asn405Lys	N405K	PROSITE_profiles:PS50011,PTHR24344:SF21,PTHR24344,1.10.510.10,Pfam_domain:PF00069,S	17.96	benign	0.016	tolerated	0.15	0	0.00018
CHEK2	22	29091743	T	C	NA	NO	missense	ENST00000328354.6:c.1214A>G	ENSP00000329178.6:p.Asn405Ser	N405S	PROSITE_profiles:PS50011,PTHR24344:SF21,PTHR24344,1.10.510.10,Pfam_domain:PF00069,S	6.853	benign	0.001	tolerated	0.7	0	0.00006
CHEK2	22	29091771	G	C	COSM3842388	NO	missense	ENST00000328354.6:c.1186C>G	ENSP00000329178.6:p.Leu396Val	L396V	PROSITE_profiles:PS50011,PTHR24344:SF21,PTHR24344,1.10.510.10,Pfam_domain:PF00069,S	22.9	benign	0.211	deleterious	0.02	0	0.00000
CHEK2	22	29091774	C	G	rs587780170	NO	missense	ENST00000328354.6:c.1183G>C	ENSP00000329178.6:p.Val395Leu	V395L	PROSITE_profiles:PS50011,PTHR24344:SF21,PTHR24344,1.10.510.10,Pfam_domain:PF00069,S	23.8	benign	0.138	deleterious	0.04	0	0.00015
CHEK2	22	29091782	G	A	rs373073383,COSM1415431,COSM1535330	NO	missense	ENST00000328354.6:c.1175C>T	ENSP00000329178.6:p.Ala392Val	A392V	PROSITE_profiles:PS50011,PTHR24344:SF21,PTHR24344,1.10.510.10,Pfam_domain:PF00069,S	34	probably_damaging	0.988	deleterious	0	65	0.00018
CHEK2	22	29091787	G	T	COSM3371882	YES	stop_gained	ENST00000328354.6:c.1170C>A	ENSP00000329178.6:p.Tyr390Ter	Y390*	PROSITE_profiles:PS50011,PTHR24344:SF21,PTHR24344,1.10.510.10,Pfam_domain:PF00069,S	39	NA	NA	NA	NA	NA	0.00000
CHEK2	22	29091810	T	C	rs769439021	NO	missense	ENST00000328354.6:c.1147A>G	ENSP00000329178.6:p.Thr383Ala	T383A	PROSITE_profiles:PS50011,PTHR24344:SF21,PTHR24344,1.10.510.10,Pfam_domain:PF00069,S	26.6	probably_damaging	0.916	deleterious	0.01	55	0.00003
CHEK2	22	29091816	TGA	T	NA	YES	frameshift,feature_truncation	ENST00000328354.6:c.1139_1140delTC	ENSP00000329178.6:p.Leu380HisfsTer14	L380X	PROSITE_profiles:PS50011,PTHR24344:SF21,PTHR24344,1.10.510.10,Pfam_domain:PF00069,S	35	NA	NA	NA	NA	NA	0.00000
CHEK2	22	29091856	AG	A	CD993415,rs555607708,KinMutBase_CHEK2_	YES	frameshift,feature_truncation	ENST00000328354.6:c.1100delC	ENSP00000329178.6:p.Thr367MetfsTer15	T367X	PROSITE_profiles:PS50011,PTHR24344:SF21,PTHR24344,1.10.510.10,Pfam_domain:PF00069,S	35	NA	NA	NA	NA	NA	0.00468
CHEK2	22	29091865	A	G	rs587782840	NO	splice,intron	ENST00000328354.6:c.1096-4T>C	NA	NA	NA	13.68	NA	NA	NA	NA	NA	0.00000
CHEK2	22	29091871	A	G	NA	NO	intron	ENST00000328354.6:c.1096-10T>C	NA	NA	NA	15.76	NA	NA	NA	NA	NA	0.00000
CHEK2	22	29092870	C	T	rs200020484	NO	intron	ENST00000328354.6:c.1095+19G>A	NA	NA	NA	8.769	NA	NA	NA	NA	NA	0.00000
CHEK2	22	29092904	C	T	NA	NO	synonymous	ENST00000328354.6:c.1080G>A	NA	E360E	PROSITE_profiles:PS50011,PTHR24344:SF21,PTHR24344,1.10.510.10,Pfam_domain:PF00069,S	14.36	NA	NA	NA	NA	NA	0.00000
CHEK2	22	29092908	T	C	rs760449049	NO	missense	ENST00000328354.6:c.1076A>G	ENSP00000329178.6:p.Glu359Gly	E359G	PROSITE_profiles:PS50011,PTHR24344:SF21,PTHR24344,1.10.510.10,Pfam_domain:PF00069,S	22.8	benign	0.164	tolerated	0.07	0	0.00006
CHEK2	22	29092914	G	A	rs765425451,CM118874	NO	missense	ENST00000328354.6:c.1070C>T	ENSP00000329178.6:p.Ser357Phe	S357F	PROSITE_profiles:PS50011,PTHR24344:SF21,PTHR24344,1.10.510.10,Pfam_domain:PF00069,S	28.2	probably_damaging	0.998	deleterious	0	65	0.00000
CHEK2	22	29092931	C	A	rs587782268,COSM1033063	NO	missense	ENST00000328354.6:c.1053G>T	ENSP00000329178.6:p.Glu351Asp	E351D	PROSITE_profiles:PS50011,PTHR24344:SF21,PTHR24344,PS00108,1.10.510.10,Pfam_domain:P	24.8	probably_damaging	0.999	tolerated	0.12	35	0.00009
CHEK2	22	29092945	C	T	rs28909980	NO	missense	ENST00000328354.6:c.1039G>A	ENSP00000329178.6:p.Asp347Asn	D347N	PROSITE_profiles:PS50011,PTHR24344:SF21,PTHR24344,PS00108,1.10.510.10,Pfam_domain:P	33	probably_damaging	1	deleterious	0.01	15	0.00000
CHEK2	22	29092948	G	A	CM110730,rs201206424,COSM1415436,COSM	NO	missense	ENST00000328354.6:c.1036C>T	ENSP00000329178.6:p.Arg346Cys	R346C	PROSITE_profiles:PS50011,PTHR24344:SF21,PTHR24344,PS00108,1.10.510.10,Pfam_domain:P	33	probably_damaging	1	deleterious	0	65	0.00015
CHEK2	22	29092951	G	A	NA	NO	missense	ENST00000328354.6:c.1033C>T	ENSP00000329178.6:p.His345Tyr	H345Y	PROSITE_profiles:PS50011,PTHR24344:SF21,PTHR24344,PS00108,1.10.510.10,Pfam_domain:P	28.8	probably_damaging	1	deleterious	0	65	0.00000
CHEK2	22	29092961	G	A	NA	NO	synonymous	ENST00000328354.6:c.1023C>T	NA	N341N	PROSITE_profiles:PS50011,PTHR24344:SF21,PTHR24344,1.10.510.10,Pfam_domain:PF00069,S	11.07	NA	NA	NA	NA	NA	0.00009
CHEK2	22	29092962	T	G	rs773846607	NO	missense	ENST00000328354.6:c.1022A>C	ENSP00000329178.6:p.Asn341Thr	N341T	PROSITE_profiles:PS50011,PTHR24344:SF21,PTHR24344,1.10.510.10,Pfam_domain:PF00069,S	24.9	probably_damaging	0.952	tolerated	0.06	55	0.00006
CHEK2	22	29092973	G	C	rs760502479	YES	stop_gained,splice	ENST00000328354.6:c.1011C>G	ENSP00000329178.6:p.Tyr337Ter	Y337*	PROSITE_profiles:PS50011,PTHR24344:SF21,PTHR24344,1.10.510.10,Pfam_domain:PF00069,S	39	NA	NA	NA	NA	NA	0.00000
CHEK2	22	29092981	C	T	rs775593362	NO	splice,intron	ENST00000328354.6:c.1009-6G>A	NA	NA	NA	13.65	NA	NA	NA	NA	NA	0.00000
CHEK2	22	29095801	C	A	rs773164077	NO	intron	ENST00000328354.6:c.1008+25G>T	NA	NA	NA	5.562	NA	NA	NA	NA	NA	0.00000
CHEK2	22	29095811	G	C	NA	NO	intron	ENST00000328354.6:c.1008+15C>G	NA	NA	NA	0.313	NA	NA	NA	NA	NA	0.00000
CHEK2	22	29095844	C	T	NA	NO	synonymous	ENST00000328354.6:c.990G>A	NA	Q330Q	PROSITE_profiles:PS50011,PTHR24344:SF21,PTHR24344,1.10.510.10,Pfam_domain:PF00069,S	11.97	NA	NA	NA	NA	NA	0.00000
CHEK2	22	29095911	T	C	NA	NO	missense	ENST00000328354.6:c.923A>G	ENSP00000329178.6:p.Glu308Gly	E308G	PROSITE_profiles:PS50011,PTHR24344:SF21,PTHR24344,1.10.510.10,Pfam_domain:PF00069,S	25	benign	0.425	deleterious	0	65	0.00000
CHEK2	22	29095913	T	TC	NA	YES	frameshift,feature_elongation	ENST00000328354.6:c.920dupG	ENSP00000329178.6:p.Glu308ArgfsTer4	G307GX	PROSITE_profiles:PS50011,PTHR24344:SF21,PTHR24344,1.10.510.10,Pfam_domain:PF00069,S	35	NA	NA	NA	NA	NA	0.00000
CHEK2	22	29095918	C	A	rs587783051,COSM94077	NO	missense	ENST00000328354.6:c.916G>T	ENSP00000329178.6:p.Gly306Trp	G306W	PROSITE_profiles:PS50011,PTHR24344:SF21,PTHR24344,1.10.510.10,Pfam_domain:PF00069,S	35	probably_damaging	1	deleterious	0	35	0.00000
CHEK2	22	29095919	T	G	rs587780191	NO	missense	ENST00000328354.6:c.915A>C	ENSP00000329178.6:p.Glu305Asp	E305D	PROSITE_profiles:PS50011,PTHR24344:SF21,PTHR24344,1.10.510.10,Pfam_domain:PF00069,S	23.7	benign	0.158	deleterious	0.05	35	0.00003
CHEK2	22	29095924	T	C	rs769436449	NO	missense,splice	ENST00000328354.6:c.910A>G	ENSP00000329178.6:p.Met304Val	M304V	PROSITE_profiles:PS50011,PTHR24344:SF21,PTHR24344,1.10.510.10,Pfam_domain:PF00069,S	11.33	benign	0.048	tolerated	0.45	0	0.00003
CHEK2	22	29099423	A	G	NA	NO	intron	ENST00000328354.6:c.908+70T>C	NA	NA	NA	12.83	NA	NA	NA	NA	NA	0.00000
CHEK2	22	29099431	C	G	NA	NO	intron	ENST00000328354.6:c.908+62G>C	NA	NA	NA	17.46	NA	NA	NA	NA	NA	0.00000
CHEK2	22	29099437	T	G	NA	NO	intron	ENST00000328354.6:c.908+56A>C	NA	NA	NA	18.66	NA	NA	NA	NA	NA	0.00000
CHEK2	22	29099474	T	A	rs759973493	NO	intron	ENST00000328354.6:c.908+19A>T	NA	NA	NA	1.087	NA	NA	NA	NA	NA	0.00000
CHEK2	22	29099475	A	T	rs755416802,rs778668907	NO	intron	ENST00000328354.6:c.908+18T>A	NA	NA	NA	0.42	NA	NA	NA	NA	NA	0.00000
CHEK2	22	29099483	T	C	rs774973319	NO	intron	ENST00000328354.6:c.908+10A>G	NA	NA	NA	10.47	NA	NA	NA	NA	NA	0.00000
CHEK2	22	29099487	A	G	rs748988275	NO	splice,intron	ENST00000328354.6:c.908+6T>C	NA	NA	NA	15.23	NA	NA	NA	NA	NA	0.00000
CHEK2	22	29099492	C	A	rs587781699	YES	splice_donor	ENST00000328354.6:c.908+1G>T	NA	NA	NA	26.5	NA	NA	NA	NA	NA	0.00000
CHEK2	22	29099494	A	G	rs752359705	NO	splice,synonymous	ENST00000328354.6:c.907T>C	NA	L303L	PROSITE_profiles:PS50011,PTHR24344:SF21,PTHR24344,1.10.510.10,Pfam_domain:PF00069,S	13.7	NA	NA	NA	NA	NA	0.00000
CHEK2	22	29099518	C	G	NA	NO	missense	ENST00000328354.6:c.883G>C	ENSP00000329178.6:p.Glu295Gln	E295Q	PROSITE_profiles:PS50011,PTHR24344:SF21,PTHR24344,1.10.510.10,Pfam_domain:PF00069,S	22.7	benign	0.117	tolerated	0.21	0	0.00000
CHEK2	22	29105959	A	G	rs17884483	NO	intron	ENST00000328354.6:c.846+35T>C	NA	NA	NA	3.935	NA	NA	NA	NA	NA	0.00000
CHEK2	22	29105960	TAA	T	NA	NO	intron,feature_truncation	ENST00000328354.6:c.846+32_846+33delTT	NA	NA	NA	9.092	NA	NA	NA	NA	NA	0.00000
CHEK2	22	29105962	A	G	NA	NO	intron	ENST00000328354.6:c.846+32T>C	NA	NA	NA	4.865	NA	NA	NA	NA	NA	0.00000
CHEK2	22	29105962	AAT	A	NA	NO	intron,feature_truncation	ENST00000328354.6:c.846+30_846+31delAT	NA	NA	NA	1.086	NA	NA	NA	NA	NA	0.00000
CHEK2	22	29105964	T	G	NA	NO	intron	ENST00000328354.6:c.846+30A>C	NA	NA	NA	4.997	NA	NA	NA	NA	NA	0.00000
CHEK2	22	29106019	A	C	NA	NO	missense	ENST00000328354.6:c.821T>G	ENSP00000329178.6:p.Ile274Arg	I274R	PROSITE_profiles:PS50011,PTHR24344:SF21,PTHR24344,Pfam_domain:PF00069,3.30.200.20,S	26.7	probably_damaging	0.988	deleterious	0	35	0.00000
CHEK2	22	29106021	TTC	T	NA	YES	frameshift,feature_truncation	ENST00000328354.6:c.817_818delGA	ENSP00000329178.6:p.Glu273AsnfsTer16	E273X	PROSITE_profiles:PS50011,PTHR24344:SF21,PTHR24344,Pfam_domain:PF00069,3.30.200.20,S	35	NA	NA	NA	NA	NA	0.00000
CHEK2	22	29106040	G	C	NA	NO	missense	ENST00000328354.6:c.800C>G	ENSP00000329178.6:p.Ala267Gly	A267G	PROSITE_profiles:PS50011,PTHR24344:SF21,PTHR24344,Pfam_domain:PF00069,3.30.200.20,S	19.29	benign	0.001	tolerated	0.41	0	0.00000
CHEK2	22	29106075	T	A	rs201218422	NO	intron	ENST00000328354.6:c.793-28A>T	NA	NA	NA	10.78	NA	NA	NA	NA	NA	0.00000
CHEK2	22	29106092	C	T	NA	NO	intron	ENST00000328354.6:c.793-45G>A	NA	NA	NA	9.663	NA	NA	NA	NA	NA	0.00000
CHEK2	22	29107824	T	A	NA	NO	regulatory	ENST00000328354.6:c.792+73A>T	NA	NA	NA	6.958	NA	NA	NA	NA	NA	0.00000
CHEK2	22	29107841	T	C	NA	NO	regulatory	ENST00000328354.6:c.792+56A>G	NA	NA	NA	6.232	NA	NA	NA	NA	NA	0.00000
CHEK2	22	29107846	A	T	rs769832204	NO	regulatory	ENST00000328354.6:c.792+51T>A	NA	NA	NA	6.451	NA	NA	NA	NA	NA	0.00000
CHEK2	22	29107855	G	C	rs368452810	NO	regulatory	ENST00000328354.6:c.792+42C>G	NA	NA	NA	1.94	NA	NA	NA	NA	NA	0.00000
CHEK2	22	29107857	C	T	rs372212841	NO	regulatory	ENST00000328354.6:c.792+40G>A	NA	NA	NA	6.97	NA	NA	NA	NA	NA	0.00000
CHEK2	22	29107858	G	A	rs140551986	NO	regulatory	ENST00000328354.6:c.792+39C>T	NA	NA	NA	3.413	NA	NA	NA	NA	NA	0.00000
CHEK2	22	29107873	G	A	rs374434631	NO	regulatory	ENST00000328354.6:c.792+24C>T	NA	NA	NA	5.278	NA	NA	NA	NA	NA	0.00000
CHEK2	22	29107902	C	G	rs730881686	NO	missense	ENST00000328354.6:c.787G>C	ENSP00000329178.6:p.Glu263Gln	E263Q	PROSITE_profiles:PS50011,PTHR24344:SF21,PTHR24344,Pfam_domain:PF00069,3.30.200.20,S	14.55	benign	0.041	tolerated	0.43	0	0.00000
CHEK2	22	29107973	T	C	rs763892124	NO	missense	ENST00000328354.6:c.716A>G	ENSP00000329178.6:p.Glu239Gly	E239G	PROSITE_profiles:PS50011,PTHR24344:SF21,PTHR24344,Pfam_domain:PF00069,3.30.200.20,S	28.1	possibly_damaging	0.864	deleterious	0.01	35	0.00000
CHEK2	22	29107974	C	T	CM030422,CM030421,rs121908702,KinMutBa	NO	missense	ENST00000328354.6:c.715G>A	ENSP00000329178.6:p.Glu239Lys	E239K	PROSITE_profiles:PS50011,PTHR24344:SF21,PTHR24344,Pfam_domain:PF00069,3.30.200.20,S	24.8	benign	0.29	tolerated	0.06	15	0.00015
CHEK2	22	29108001	C	A	rs748636216,COSM3720426	NO	missense	ENST00000328354.6:c.688G>T	ENSP00000329178.6:p.Ala230Ser	A230S	PROSITE_profiles:PS50011,PTHR24344:SF21,PTHR24344,Pfam_domain:PF00069,3.30.200.20,S	25.7	probably_damaging	0.974	tolerated	0.15	15	0.00006
CHEK2	22	29108009	G	A	rs121908695	NO	splice,intron	ENST00000328354.6:c.684-4C>T	NA	NA	NA	4.619	NA	NA	NA	NA	NA	0.00000
CHEK2	22	29108021	A	G	NA	NO	regulatory	ENST00000328354.6:c.684-16T>C	NA	NA	NA	1.709	NA	NA	NA	NA	NA	0.00000
CHEK2	22	29115374	A	G	NA	NO	intron	ENST00000328354.6:c.683+9T>C	NA	NA	NA	9.216	NA	NA	NA	NA	NA	0.00000
CHEK2	22	29115402	T	C	NA	NO	missense	ENST00000328354.6:c.664A>G	ENSP00000329178.6:p.Met222Val	M222V	PROSITE_profiles:PS50011,PTHR24344:SF21,PTHR24344,Pfam_domain:PF00069,3.30.200.20,S	11.19	benign	0.023	tolerated	0.45	0	0.00000
CHEK2	22	29115405	T	C	rs199749372	NO	missense	ENST00000328354.6:c.661A>G	ENSP00000329178.6:p.Ile221Val	I221V	PROSITE_profiles:PS50011,PTHR24344:SF21,PTHR24344,3.30.200.20,SMART_domains:SM002	13.07	benign	0.051	tolerated	0.49	0	0.00014
CHEK2	22	29115415	T	C	rs774245273	NO	synonymous	ENST00000328354.6:c.651A>G	NA	R217R	PTHR24344:SF21,PTHR24344,3.30.200.20,Superfamily_domains:SSF56112	5.463	NA	NA	NA	NA	NA	0.00000
CHEK2	22	29115437	G	A	rs767253467,COSM1033069	NO	missense	ENST00000328354.6:c.629C>T	ENSP00000329178.6:p.Ser210Leu	S210L	PTHR24344:SF21,PTHR24344	23.1	benign	0.039	tolerated	0.19	0	0.00013
CHEK2	22	29115455	A	G	NA	NO	missense	ENST00000328354.6:c.611T>C	ENSP00000329178.6:p.Leu204Pro	L204P	PTHR24344:SF21,PTHR24344,2.60.200.20,Superfamily_domains:SSF49879	23	benign	0.072	tolerated	0.06	0	0.00000
CHEK2	22	29120911	C	A	NA	NO	intron	ENST00000328354.6:c.592+54G>T	NA	NA	NA	4.869	NA	NA	NA	NA	NA	0.00000
CHEK2	22	29120915	TGA	T	rs756853924	NO	intron,feature_truncation	ENST00000328354.6:c.592+48_592+49delTC	NA	NA	NA	1.957	NA	NA	NA	NA	NA	0.00000
CHEK2	22	29120930	A	C	rs149092712	NO	intron	ENST00000328354.6:c.592+35T>G	NA	NA	NA	0.148	NA	NA	NA	NA	NA	0.00000
CHEK2	22	29120961	T	C	NA	NO	splice,intron	ENST00000328354.6:c.592+4A>G	NA	NA	NA	1.515	NA	NA	NA	NA	NA	0.00000
CHEK2	22	29120965	CT	C	rs587782245,CD077681	YES	frameshift,splice,feature_truncation	ENST00000328354.6:c.591delA	ENSP00000329178.6:p.Val198PhefsTer7	K197X	PTHR24344:SF21,PTHR24344,2.60.200.20,Superfamily_domains:SSF49879	26.4	NA	NA	NA	NA	NA	0.00000
CHEK2	22	29120977	T	A	NA	NO	missense	ENST00000328354.6:c.580A>T	ENSP00000329178.6:p.Ser194Cys	S194C	PTHR24344:SF21,PTHR24344,2.60.200.20,Superfamily_domains:SSF49879	3.004	benign	0.005	tolerated	0.07	0	0.00000
CHEK2	22	29120979	A	G	rs766599514	NO	missense	ENST00000328354.6:c.578T>C	ENSP00000329178.6:p.Leu193Pro	L193P	PTHR24344:SF21,PTHR24344,2.60.200.20,Superfamily_domains:SSF49879	22.5	possibly_damaging	0.894	tolerated	0.21	0	0.00003
CHEK2	22	29120987	T	G	rs754689169	NO	synonymous	ENST00000328354.6:c.570A>C	NA	A190A	PTHR24344:SF21,PTHR24344,Pfam_domain:PF00498,2.60.200.20,Superfamily_domains:SSF498	0.847	NA	NA	NA	NA	NA	0.00000
CHEK2	22	29121000	T	C	rs369223840,COSM3371883	NO	missense	ENST00000328354.6:c.557A>G	ENSP00000329178.6:p.Asn186Ser	N186S	PTHR24344:SF21,PTHR24344,Pfam_domain:PF00498,2.60.200.20,Superfamily_domains:SSF498	22.1	probably_damaging	0.95	tolerated	0.1	45	0.00003
CHEK2	22	29121001	T	G	rs146198085	NO	missense	ENST00000328354.6:c.556A>C	ENSP00000329178.6:p.Asn186His	N186H	PTHR24344:SF21,PTHR24344,Pfam_domain:PF00498,2.60.200.20,Superfamily_domains:SSF498	24.1	probably_damaging	0.998	deleterious	0.01	65	0.00018
CHEK2	22	29121016	G	A	CM030419,rs137853010	NO	missense	ENST00000328354.6:c.541C>T	ENSP00000329178.6:p.Arg181Cys	R181C	PTHR24344:SF21,PTHR24344,Pfam_domain:PF00498,2.60.200.20,Superfamily_domains:SSF498	22.2	benign	0.005	tolerated	0.18	0	0.00006
CHEK2	22	29121018	C	T	CM030418,rs137853009,COSM2935994	NO	missense	ENST00000328354.6:c.539G>A	ENSP00000329178.6:p.Arg180His	R180H	PTHR24344:SF21,PTHR24344,Pfam_domain:PF00498,2.60.200.20,Superfamily_domains:SSF498	21.9	benign	0.03	tolerated	0.09	0	0.00009
CHEK2	22	29121019	G	A	CM030417,rs77130927	NO	missense	ENST00000328354.6:c.538C>T	ENSP00000329178.6:p.Arg180Cys	R180C	PTHR24344:SF21,PTHR24344,Pfam_domain:PF00498,2.60.200.20,Superfamily_domains:SSF498	23.2	benign	0.081	tolerated	0.06	25	0.00342
CHEK2	22	29121037	G	A	CM086683	NO	missense	ENST00000328354.6:c.520C>T	ENSP00000329178.6:p.Leu174Phe	L174F	PROSITE_profiles:PS50006,PTHR24344:SF21,PTHR24344,Pfam_domain:PF00498,2.60.200.20,S	22.8	possibly_damaging	0.875	tolerated	0.22	0	0.00000
CHEK2	22	29121058	C	T	rs72552322,CM030416	NO	missense	ENST00000328354.6:c.499G>A	ENSP00000329178.6:p.Gly167Arg	G167R	PROSITE_profiles:PS50006,PTHR24344:SF21,PTHR24344,Pfam_domain:PF00498,2.60.200.20,S	32	probably_damaging	1	deleterious	0	65	0.00012
CHEK2	22	29121075	T	C	rs730881683	NO	missense	ENST00000328354.6:c.482A>G	ENSP00000329178.6:p.Glu161Gly	E161G	PROSITE_profiles:PS50006,PTHR24344:SF21,PTHR24344,Pfam_domain:PF00498,2.60.200.20,S	28.1	probably_damaging	0.974	deleterious	0.02	65	0.00000
CHEK2	22	29121077	T	C	rs575910805	NO	missense	ENST00000328354.6:c.480A>G	ENSP00000329178.6:p.Ile160Met	I160M	PROSITE_profiles:PS50006,PTHR24344:SF21,PTHR24344,Pfam_domain:PF00498,2.60.200.20,S	23.6	probably_damaging	0.917	deleterious	0	0	0.00009
CHEK2	22	29121081	T	C	COSM1284112	NO	missense	ENST00000328354.6:c.476A>G	ENSP00000329178.6:p.Tyr159Cys	Y159C	PROSITE_profiles:PS50006,PTHR24344:SF21,PTHR24344,Pfam_domain:PF00498,2.60.200.20,S	26	probably_damaging	0.987	deleterious	0	55	0.00000
CHEK2	22	29121083	T	G	rs745699485	NO	synonymous	ENST00000328354.6:c.474A>C	NA	A158A	PROSITE_profiles:PS50006,PTHR24344:SF21,PTHR24344,Pfam_domain:PF00498,2.60.200.20,S	10.69	NA	NA	NA	NA	NA	0.00012
CHEK2	22	29121085	C	T	rs755934632	NO	missense	ENST00000328354.6:c.472G>A	ENSP00000329178.6:p.Ala158Thr	A158T	PROSITE_profiles:PS50006,PTHR24344:SF21,PTHR24344,Pfam_domain:PF00498,2.60.200.20,S	22	benign	0.03	tolerated	0.26	0	0.00000
CHEK2	22	29121087	A	G	CM993368,rs17879961,COSM3693990	NO	missense	ENST00000328354.6:c.470T>C	ENSP00000329178.6:p.Ile157Thr	I157T	PROSITE_profiles:PS50006,PTHR24344:SF21,PTHR24344,Pfam_domain:PF00498,2.60.200.20,S	21.1	benign	0.352	tolerated	0.21	15	0.00947
CHEK2	22	29121162	G	C	rs377700765	NO	intron	ENST00000328354.6:c.445-50C>G	NA	NA	NA	2.541	NA	NA	NA	NA	NA	0.00000
CHEK2	22	29121166	G	A	rs763797009	NO	intron	ENST00000328354.6:c.445-54C>T	NA	NA	NA	6.746	NA	NA	NA	NA	NA	0.00000
CHEK2	22	29121186	A	G	NA	NO	intron	ENST00000328354.6:c.444+45T>C	NA	NA	NA	5.782	NA	NA	NA	NA	NA	0.00000
CHEK2	22	29121207	G	A	rs121908699	NO	intron	ENST00000328354.6:c.444+24C>T	NA	NA	NA	2.665	NA	NA	NA	NA	NA	0.00000
CHEK2	22	29121223	T	C	NA	NO	splice,intron	ENST00000328354.6:c.444+8A>G	NA	NA	NA	0.919	NA	NA	NA	NA	NA	0.00000
CHEK2	22	29121230	C	T	CS030536,CS086684,rs121908698	YES	splice_donor	ENST00000328354.6:c.444+1G>A	NA	NA	NA	22.7	NA	NA	NA	NA	NA	0.00033
CHEK2	22	29121242	G	A	CM013593,rs137853007	NO	missense	ENST00000328354.6:c.433C>T	ENSP00000329178.6:p.Arg145Trp	R145W	PROSITE_profiles:PS50006,PTHR24344:SF21,PTHR24344,Pfam_domain:PF00498,2.60.200.20,S	33	probably_damaging	0.999	deleterious	0.01	65	0.00006
CHEK2	22	29121247	T	C	rs587782300,CM118873	NO	missense	ENST00000328354.6:c.428A>G	ENSP00000329178.6:p.His143Arg	H143R	PROSITE_profiles:PS50006,PTHR24344:SF21,PTHR24344,Pfam_domain:PF00498,2.60.200.20,S	25.3	probably_damaging	1	deleterious	0	25	0.00000
CHEK2	22	29121265	C	T	rs368570187,CM023899	NO	missense	ENST00000328354.6:c.410G>A	ENSP00000329178.6:p.Arg137Gln	R137Q	PROSITE_profiles:PS50006,PTHR24344:SF21,PTHR24344,Pfam_domain:PF00498,2.60.200.20,S	18.16	benign	0.019	tolerated	0.61	0	0.00057
CHEK2	22	29121266	G	A	rs730881701	YES	stop_gained	ENST00000328354.6:c.409C>T	ENSP00000329178.6:p.Arg137Ter	R137*	PROSITE_profiles:PS50006,PTHR24344:SF21,PTHR24344,Pfam_domain:PF00498,2.60.200.20,S	36	NA	NA	NA	NA	NA	0.00009
CHEK2	22	29121275	C	G	rs372874441,COSM1033071	NO	missense	ENST00000328354.6:c.400G>C	ENSP00000329178.6:p.Asp134His	D134H	PROSITE_profiles:PS50006,PTHR24344:SF21,PTHR24344,Pfam_domain:PF00498,2.60.200.20,S	23.8	possibly_damaging	0.62	tolerated	0.11	0	0.00003
CHEK2	22	29121307	T	C	NA	NO	missense	ENST00000328354.6:c.368A>G	ENSP00000329178.6:p.Tyr123Cys	Y123C	PROSITE_profiles:PS50006,PTHR24344:SF21,PTHR24344,Pfam_domain:PF00498,2.60.200.20,S	23.4	possibly_damaging	0.712	tolerated	0.07	35	0.00000
CHEK2	22	29121325	C	T	rs587781982	NO	missense	ENST00000328354.6:c.350G>A	ENSP00000329178.6:p.Arg117Lys	R117K	PROSITE_profiles:PS50006,PTHR24344:SF21,PTHR24344,Pfam_domain:PF00498,2.60.200.20,S	32	probably_damaging	0.999	deleterious	0	25	0.00000
CHEK2	22	29121326	T	C	CM023898,rs28909982	NO	missense	ENST00000328354.6:c.349A>G	ENSP00000329178.6:p.Arg117Gly	R117G	PROSITE_profiles:PS50006,PTHR24344:SF21,PTHR24344,Pfam_domain:PF00498,2.60.200.20,S	26.8	probably_damaging	1	deleterious	0	65	0.00039
CHEK2	22	29121353	A	G	rs730881681	NO	missense,splice	ENST00000328354.6:c.322T>C	ENSP00000329178.6:p.Cys108Arg	C108R	PTHR24344:SF21,PTHR24344,2.60.200.20,Superfamily_domains:SSF49879	26.3	probably_damaging	0.983	deleterious	0.02	0	0.00000
CHEK2	22	29121360	A	T	CS086685,rs121908700	NO	splice,intron	ENST00000328354.6:c.320-5T>A	NA	NA	NA	12.02	NA	NA	NA	NA	NA	0.00000
CHEK2	22	29121362	T	C	NA	NO	splice,intron	ENST00000328354.6:c.320-7A>G	NA	NA	NA	8.379	NA	NA	NA	NA	NA	0.00000
CHEK2	22	29130326	G	A	NA	NO	intron	ENST00000328354.6:c.319+65C>T	NA	NA	NA	1.584	NA	NA	NA	NA	NA	0.00000
CHEK2	22	29130329	A	C	NA	NO	intron	ENST00000328354.6:c.319+62T>G	NA	NA	NA	0.965	NA	NA	NA	NA	NA	0.00000
CHEK2	22	29130330	T	C	NA	NO	intron	ENST00000328354.6:c.319+61A>G	NA	NA	NA	0.285	NA	NA	NA	NA	NA	0.00000
CHEK2	22	29130347	G	GT	rs3841692	NO	intron,feature_elongation	ENST00000328354.6:c.319+43dupA	NA	NA	NA	2.024	NA	NA	NA	NA	NA	0.00000
CHEK2	22	29130351	T	G	NA	NO	intron	ENST00000328354.6:c.319+40A>C	NA	NA	NA	5.298	NA	NA	NA	NA	NA	0.00000
CHEK2	22	29130363	C	T	rs774308320	NO	intron	ENST00000328354.6:c.319+28G>A	NA	NA	NA	2.293	NA	NA	NA	NA	NA	0.00000
CHEK2	22	29130427	G	A	CM110734,rs587781269	YES	stop_gained	ENST00000328354.6:c.283C>T	ENSP00000329178.6:p.Arg95Ter	R95*	PTHR24344:SF21,PTHR24344,2.60.200.20,Superfamily_domains:SSF49879	38	NA	NA	NA	NA	NA	0.00000
CHEK2	22	29130431	C	T	rs587782070	YES	stop_gained	ENST00000328354.6:c.279G>A	ENSP00000329178.6:p.Trp93Ter	W93*	PTHR24344:SF21,PTHR24344,2.60.200.20,Superfamily_domains:SSF49879	38	NA	NA	NA	NA	NA	0.00006
CHEK2	22	29130434	G	A	NA	NO	synonymous	ENST00000328354.6:c.276C>T	NA	P92P	Low_complexity_(Seg):seg,PTHR24344:SF21,PTHR24344,2.60.200.20,Superfamily_domains:SSF	7.624	NA	NA	NA	NA	NA	0.00000
CHEK2	22	29130456	G	C	CM077521,rs17883862,CM119711	NO	missense	ENST00000328354.6:c.254C>G	ENSP00000329178.6:p.Pro85Arg	P85R	Low_complexity_(Seg):seg,PTHR24344:SF21,PTHR24344	23	possibly_damaging	0.894	tolerated	0.08	0	0.00000
CHEK2	22	29130458	T	C	rs1805129,COSM149275	NO	synonymous	ENST00000328354.6:c.252A>G	NA	E84E	Low_complexity_(Seg):seg,PTHR24344:SF21,PTHR24344	2.59	NA	NA	NA	NA	NA	0.05686
CHEK2	22	29130471	G	T	NA	NO	missense	ENST00000328354.6:c.239C>A	ENSP00000329178.6:p.Pro80His	P80H	Low_complexity_(Seg):seg,PTHR24344:SF21,PTHR24344	26.5	probably_damaging	1	deleterious	0.02	0	0.00000
CHEK2	22	29130520	C	T	CM119699,CM030414,rs141568342	NO	missense	ENST00000328354.6:c.190G>A	ENSP00000329178.6:p.Glu64Lys	E64K	PTHR24344:SF21,PTHR24344	23	benign	0.011	tolerated	0.1	15	0.00057
CHEK2	22	29130521	T	C	rs761028062	NO	synonymous	ENST00000328354.6:c.189A>G	NA	L63L	PTHR24344:SF21,PTHR24344	7.454	NA	NA	NA	NA	NA	0.00000
CHEK2	22	29130534	G	T	CM023347,rs149991239	NO	missense	ENST00000328354.6:c.176C>A	ENSP00000329178.6:p.Thr59Lys	T59K	Low_complexity_(Seg):seg,PTHR24344:SF21,PTHR24344	31	probably_damaging	0.99	deleterious	0.01	65	0.00003
CHEK2	22	29130583	T	C	rs749158713	NO	missense	ENST00000328354.6:c.127A>G	ENSP00000329178.6:p.Thr43Ala	T43A	Low_complexity_(Seg):seg,PTHR24344:SF21,PTHR24344	0.023	benign	0.003	tolerated	0.66	0	0.00000
CHEK2	22	29130595	A	G	NA	NO	missense	ENST00000328354.6:c.115T>C	ENSP00000329178.6:p.Ser39Pro	S39P	Low_complexity_(Seg):seg,PTHR24344:SF21,PTHR24344	24.8	possibly_damaging	0.528	deleterious	0.01	0	0.00000
CHEK2	22	29130606	G	C	NA	NO	missense	ENST00000328354.6:c.104C>G	ENSP00000329178.6:p.Ser35Cys	S35C	Low_complexity_(Seg):seg,PTHR24344:SF21,PTHR24344	24	possibly_damaging	0.891	deleterious	0.03	15	0.00000
CHEK2	22	29130607	A	G	NA	NO	missense	ENST00000328354.6:c.103T>C	ENSP00000329178.6:p.Ser35Pro	S35P	Low_complexity_(Seg):seg,PTHR24344:SF21,PTHR24344	24.1	possibly_damaging	0.623	tolerated	0.06	0	0.00000
CHEK2	22	29130636	A	G	rs587780188	NO	missense	ENST00000328354.6:c.74T>C	ENSP00000329178.6:p.Val25Ala	V25A	Low_complexity_(Seg):seg,PTHR24344:SF21,PTHR24344	0.298	benign	0	tolerated	0.08	0	0.00027
CHEK2	22	29130652	G	A	rs536907995	YES	stop_gained	ENST00000328354.6:c.58C>T	ENSP00000329178.6:p.Gln20Ter	Q20*	PTHR24344:SF21,PTHR24344	35	NA	NA	NA	NA	NA	0.00000

CHEK222	29130696	G	A	rs201084748	NO	missense	ENST00000328354.6:c.14C>T	ENSP00000329178.6:p.Ser5Leu	S5L	PTHR24344:SF21,PTHR24344	23.2	benign	0.003	deleterious	0.01	0	0.00015
CHEK222	29130703	G	A	rs199708878	NO	missense	ENST00000328354.6:c.7C>T	ENSP00000329178.6:p.Arg3Trp	R3W	PTHR24344:SF21,PTHR24344	34	possibly_damaging	0.648	deleterious	0	25	0.00055
CHEK222	29130713	G	A	rs374938148,CR068233	NO	splice,5_prime_UTR	ENST00000328354.6:c.-4C>T	NA	NA	NA	14.14	NA	NA	NA	NA	NA	0.00000
CHEK222	29130724	G	C	rs565169061	NO	intron	ENST00000328354.6:c.-6-9C>G	NA	NA	NA	13.72	NA	NA	NA	NA	NA	0.00000
PALB2 16	23614750	C	A	NA	NO	intron	ENST00000261584.4:c.*30G>T	NA	NA	NA	9.745	NA	NA	NA	NA	NA	0.00000
PALB2 16	23614789	G	C	NA	NO	missense	ENST00000261584.4:c.3552C>G	ENSP00000261584.4:p.His1184Gln	H1184Q	PTHR14662:SF2,PTHR14662,2.130.10.10,Superfamily_domains:SSF50978	3.31	benign	0.018	tolerated	0.13	0	0.00000
PALB2 16	23614792	G	C	CM070240,CM070239,rs118203998,PALB2:c.3	YES	stop_gained	ENST00000261584.4:c.3549C>G	ENSP00000261584.4:p.Tyr1183Ter	Y1183*	PTHR14662:SF2,PTHR14662,2.130.10.10,Superfamily_domains:SSF50978	35	NA	NA	NA	NA	NA	0.00006
PALB2 16	23614823	G	A	rs146659762	NO	missense	ENST00000261584.4:c.3518C>T	ENSP00000261584.4:p.Ala1173Val	A1173V	PTHR14662:SF2,PTHR14662,2.130.10.10,Superfamily_domains:SSF50978	29.2	possibly_damaging	0.687	deleterious	0.01	0	0.00000
PALB2 16	23614846	C	T	rs45439097	NO	synonymous	ENST00000261584.4:c.3495G>A	NA	S1165S	PTHR14662:SF2,PTHR14662,2.130.10.10,Superfamily_domains:SSF50978	18.52	NA	NA	NA	NA	NA	0.00216
PALB2 16	23614858	A	G	rs372686500	NO	synonymous	ENST00000261584.4:c.3483T>C	NA	F1161F	PTHR14662:SF2,PTHR14662,2.130.10.10,Superfamily_domains:SSF50978	0.153	NA	NA	NA	NA	NA	0.00003
PALB2 16	23614908	C	T	NA	NO	missense	ENST00000261584.4:c.3433G>A	ENSP00000261584.4:p.Gly1145Ser	G1145S	PTHR14662:SF2,PTHR14662,2.130.10.10,Superfamily_domains:SSF50978	26.6	probably_damaging	1	tolerated	0.14	0	0.00000
PALB2 16	23614913	A	T	CM113953,rs62625284	NO	missense	ENST00000261584.4:c.3428T>A	ENSP00000261584.4:p.Leu1143His	L1143H	PTHR14662:SF2,PTHR14662,2.130.10.10,Superfamily_domains:SSF50978	24.8	probably_damaging	0.984	tolerated	0.09	0	0.00063
PALB2 16	23614986	G	C	rs748338957	NO	missense	ENST00000261584.4:c.3355C>G	ENSP00000261584.4:p.Leu1119Val	L1119V	PTHR14662:SF2,PTHR14662	25.2	possibly_damaging	0.858	deleterious	0.01	25	0.00000
PALB2 16	23614996	A	G	rs770987416	NO	splice,intron	ENST00000261584.4:c.3351-6T>C	NA	NA	NA	5.812	NA	NA	NA	NA	NA	0.00000
PALB2 16	23614997	G	T	NA	NO	splice,intron	ENST00000261584.4:c.3351-7C>A	NA	NA	NA	1.565	NA	NA	NA	NA	NA	0.00000
PALB2 16	23615018	C	T	NA	NO	intron	ENST00000261584.4:c.3351-28G>A	NA	NA	NA	4.59	NA	NA	NA	NA	NA	0.00000
PALB2 16	23619157	C	A	NA	NO	regulatory	ENST00000261584.4:c.3350+28G>T	NA	NA	NA	9.629	NA	NA	NA	NA	NA	0.00000
PALB2 16	23619174	T	C	rs515726114	NO	regulatory	ENST00000261584.4:c.3350+11A>G	NA	NA	NA	5.929	NA	NA	NA	NA	NA	0.00000
PALB2 16	23619182	T	C	NA	NO	splice,intron	ENST00000261584.4:c.3350+3A>G	NA	NA	NA	14.76	NA	NA	NA	NA	NA	0.00000
PALB2 16	23619188	C	T	NA	NO	missense	ENST00000261584.4:c.3347G>A	ENSP00000261584.4:p.Gly1116Asp	G1116D	PTHR14662:SF2,PTHR14662	24.8	benign	0.24	deleterious	0.01	0	0.00000
PALB2 16	23619225	C	T	rs188254555	NO	missense	ENST00000261584.4:c.3310G>A	ENSP00000261584.4:p.Gly1104Ser	G1104S	PTHR14662:SF2,PTHR14662	18.75	benign	0.129	tolerated	0.08	0	0.00000
PALB2 16	23619228	C	G	rs201657283,CM118272	NO	missense	ENST00000261584.4:c.3307G>C	ENSP00000261584.4:p.Val1103Leu	V1103L	PTHR14662:SF2,PTHR14662	15.35	benign	0.041	tolerated	0.08	0	0.00003
PALB2 16	23619228	C	T	rs201657283,CM118272	NO	missense	ENST00000261584.4:c.3307G>A	ENSP00000261584.4:p.Val1103Met	V1103M	PTHR14662:SF2,PTHR14662	16.26	benign	0.043	tolerated	0.11	0	0.00003
PALB2 16	23619235	A	C	rs45516100	NO	synonymous	ENST00000261584.4:c.3300T>G	NA	T1100T	PTHR14662:SF2,PTHR14662	0.079	NA	NA	NA	NA	NA	0.05571
PALB2 16	23619238	C	T	rs45565738	NO	synonymous	ENST00000261584.4:c.3297G>A	NA	T1099T	PTHR14662:SF2,PTHR14662	18.68	NA	NA	NA	NA	NA	0.00036
PALB2 16	23619239	G	C	rs142132127	NO	missense	ENST00000261584.4:c.3296C>G	ENSP00000261584.4:p.Thr1099Arg	T1099R	PTHR14662:SF2,PTHR14662	31	probably_damaging	0.971	deleterious	0	15	0.00012
PALB2 16	23619253	C	T	rs747861082	NO	synonymous	ENST00000261584.4:c.3282G>A	NA	V1094V	PTHR14662:SF2,PTHR14662	13.77	NA	NA	NA	NA	NA	0.00000
PALB2 16	23619279	G	A	rs587776527,CM092063,COSM313874	YES	stop_gained	ENST00000261584.4:c.3256C>T	ENSP00000261584.4:p.Arg1086Ter	R1086*	PTHR14662:SF2,PTHR14662	37	NA	NA	NA	NA	NA	0.00003
PALB2 16	23619283	C	T	rs141570833	NO	synonymous	ENST00000261584.4:c.3252G>A	NA	S1084S	PTHR14662:SF2,PTHR14662	14.42	NA	NA	NA	NA	NA	0.00009
PALB2 16	23619284	G	A	rs62625271	NO	missense	ENST00000261584.4:c.3251C>T	ENSP00000261584.4:p.Ser1084Leu	S1084L	PTHR14662:SF2,PTHR14662	13.18	benign	0.01	tolerated	0.37	0	0.00027
PALB2 16	23619286	C	G	CM118271,rs147045425,COSM1637813	NO	missense	ENST00000261584.4:c.3249G>C	ENSP00000261584.4:p.Glu1083Asp	E1083D	PTHR14662:SF2,PTHR14662	23.1	benign	0.134	deleterious	0.03	15	0.00006
PALB2 16	23619300	C	A	rs730881878	NO	missense	ENST00000261584.4:c.3235G>T	ENSP00000261584.4:p.Ala1079Ser	A1079S	PTHR14662:SF2,PTHR14662	22.7	benign	0.437	tolerated	0.18	0	0.00000
PALB2 16	23619302	C	G	NA	NO	missense	ENST00000261584.4:c.3233G>C	ENSP00000261584.4:p.Cys1078Ser	C1078S	PTHR14662:SF2,PTHR14662	24	possibly_damaging	0.636	tolerated	0.4	0	0.00000
PALB2 16	23619313	C	A	NA	NO	synonymous	ENST00000261584.4:c.3222G>T	NA	L1074L	PTHR14662:SF2,PTHR14662,2.130.10.10,Superfamily_domains:SSF50978	16.85	NA	NA	NA	NA	NA	0.00000
PALB2 16	23619315	G	C	NA	NO	missense	ENST00000261584.4:c.3220C>G	ENSP00000261584.4:p.Leu1074Val	L1074V	PTHR14662:SF2,PTHR14662,2.130.10.10,Superfamily_domains:SSF50978	23.3	possibly_damaging	0.489	tolerated	0.06	0	0.00000
PALB2 16	23619332	C	T	rs759587160	NO	missense,splice	ENST00000261584.4:c.3203G>A	ENSP00000261584.4:p.Gly1068Glu	G1068E	PTHR14662:SF2,PTHR14662,2.130.10.10,Superfamily_domains:SSF50978	33	probably_damaging	0.994	deleterious	0	65	0.00003
PALB2 16	23619341	C	A	rs367979106	NO	splice,intron	ENST00000261584.4:c.3202-8G>T	NA	NA	NA	3.79	NA	NA	NA	NA	NA	0.00000
PALB2 16	23619341	C	T	rs367979106	NO	splice,intron	ENST00000261584.4:c.3202-8G>A	NA	NA	NA	3.715	NA	NA	NA	NA	NA	0.00000
PALB2 16	23619342	G	A	rs757444247	NO	intron	ENST00000261584.4:c.3202-9C>T	NA	NA	NA	1.742	NA	NA	NA	NA	NA	0.00000
PALB2 16	23625320	C	G	NA	NO	splice,intron	ENST00000261584.4:c.3201+5G>C	NA	NA	NA	13.28	NA	NA	NA	NA	NA	0.00000
PALB2 16	23625409	AT	A	rs180177133,CD070513,PALB2:c.3116delA	YES	frameshift,splice,feature_truncation	ENST00000261584.4:c.3116delA	ENSP00000261584.4:p.Asn1039IlefsTer2	N1039X	PTHR14662:SF2,PTHR14662,2.130.10.10,Superfamily_domains:SSF50978	35	NA	NA	NA	NA	NA	0.00000
PALB2 16	23632655	G	T	rs145973806	NO	intron	ENST00000261584.4:c.3113+28C>A	NA	NA	NA	0.579	NA	NA	NA	NA	NA	0.00000
PALB2 16	23632683	C	T	CM070242,rs180177132	YES	stop_gained,splice	ENST00000261584.4:c.3113G>A	ENSP00000261584.4:p.Trp1038Ter	W1038*	PTHR14662:SF2,PTHR14662,2.130.10.10,Superfamily_domains:SSF50978	43	NA	NA	NA	NA	NA	0.00006
PALB2 16	23632690	C	G	rs756906403	NO	missense	ENST00000261584.4:c.3106G>C	ENSP00000261584.4:p.Val1036Leu	V1036L	PTHR14662:SF2,PTHR14662,2.130.10.10,Superfamily_domains:SSF50978	21.5	benign	0.092	tolerated	0.05	0	0.00006
PALB2 16	23632708	T	C	NA	NO	missense	ENST00000261584.4:c.3088A>G	ENSP00000261584.4:p.Thr1030Ala	T1030A	PTHR14662:SF2,PTHR14662,2.130.10.10,Superfamily_domains:SSF50978	25.1	probably_damaging	0.976	deleterious	0.01	55	0.00000
PALB2 16	23632736	T	C	NA	NO	synonymous	ENST00000261584.4:c.3060A>G	NA	Q1020Q	PTHR14662:SF2,PTHR14662,Superfamily_domains:SSF50978	1.782	NA	NA	NA	NA	NA	0.00000
PALB2 16	23632752	G	A	rs367840862	NO	missense	ENST00000261584.4:c.3044C>T	ENSP00000261584.4:p.Thr1015Ile	T1015I	PTHR14662:SF2,PTHR14662,Superfamily_domains:SSF50978	26	probably_damaging	0.996	deleterious	0.01	0	0.00006
PALB2 16	23632827	G	A	NA	NO	intron	ENST00000261584.4:c.2997-28C>T	NA	NA	NA	2.511	NA	NA	NA	NA	NA	0.00000
PALB2 16	23634248	T	C	NA	NO	intron	ENST00000261584.4:c.2996+42A>G	NA	NA	NA	12.52	NA	NA	NA	NA	NA	0.00000
PALB2 16	23634273	A	G	rs180177128	NO	intron	ENST00000261584.4:c.2996+17T>C	NA	NA	NA	18.26	NA	NA	NA	NA	NA	0.00000
PALB2 16	23634293	C	T	rs45551636,CM098533	NO	missense	ENST00000261584.4:c.2993G>A	ENSP00000261584.4:p.Gly998Glu	G998E	PTHR14662:SF2,PTHR14662,Superfamily_domains:SSF50978	33	probably_damaging	0.992	deleterious	0	65	0.04238
PALB2 16	23634308	G	A	rs61756146	NO	missense	ENST00000261584.4:c.2978C>T	ENSP00000261584.4:p.Thr993Met	T993M	PTHR14662:SF2,PTHR14662,Superfamily_domains:SSF50978	24.7	possibly_damaging	0.743	tolerated	0.1	15	0.00000
PALB2 16	23634315	C	A	rs765175665	NO	missense	ENST00000261584.4:c.2971G>T	ENSP00000261584.4:p.Val991Phe	V991F	PTHR14662:SF2,PTHR14662,Superfamily_domains:SSF50978	23.1	possibly_damaging	0.822	deleterious	0.01	0	0.00003
PALB2 16	23634324	G	A	CM070236,rs118203999,PALB2:c.2962C>T	YES	stop_gained	ENST00000261584.4:c.2962C>T	ENSP00000261584.4:p.Gln988Ter	Q988*	PTHR14662:SF2,PTHR14662,Superfamily_domains:SSF50978	36	NA	NA	NA	NA	NA	0.00000
PALB2 16	23634339	T	A	rs587782211	NO	missense	ENST00000261584.4:c.2947A>T	ENSP00000261584.4:p.Thr983Ser	T983S	PTHR14662:SF2,PTHR14662,Superfamily_domains:SSF50978	13.27	benign	0.173	tolerated	0.42	0	0.00000
PALB2 16	23634350	C	A	NA	NO	missense	ENST00000261584.4:c.2936G>T	ENSP00000261584.4:p.Ser979Ile	S979I	PTHR14662:SF2,PTHR14662,Superfamily_domains:SSF50978	22.9	benign	0.197	deleterious	0.03	0	0.00000
PALB2 16	23634364	CTT	C	rs180177126	YES	frameshift,feature_truncation	ENST00000261584.4:c.2920_2921delAA	ENSP00000261584.4:p.Lys974GlufsTer5	K974X	PTHR14662:SF2,PTHR14662,Superfamily_domains:SSF50978	34	NA	NA	NA	NA	NA	0.00000
PALB2 16	23634427	A	G	rs515726101	NO	synonymous	ENST00000261584.4:c.2859T>C	NA	D953D	PTHR14662:SF2,PTHR14662,Superfamily_domains:SSF50978	2.72	NA	NA	NA	NA	NA	0.00003
PALB2 16	23634435	A	G	rs149522412	NO	missense	ENST00000261584.4:c.2851T>C	ENSP00000261584.4:p.Ser951Pro	S951P	PTHR14662:SF2,PTHR14662,Superfamily_domains:SSF50978	7.041	benign	0.045	tolerated	0.26	0	0.00018
PALB2 16	23634446	A	G	rs45464500	NO	missense	ENST00000261584.4:c.2840T>C	ENSP00000261584.4:p.Leu947Ser	L947S	PTHR14662:SF2,PTHR14662,Superfamily_domains:SSF50978	25.5	probably_damaging	0.995	deleterious	0	65	0.00016
PALB2 16	23635308	T	G	rs766701745	NO	non_coding_exon,nc	ENST00000261584.4:c.2834+22A>C	NA	NA	NA	7.802	NA	NA	NA	NA	NA	0.00000
PALB2 16	23635311	C	T	NA	NO	non_coding_exon,nc	ENST00000261584.4:c.2834+19G>A	NA	NA	NA	6.039	NA	NA	NA	NA	NA	0.00000
PALB2 16	23635317	G	T	rs551516577	NO	non_coding_exon,nc	ENST00000261584.4:c.2834+13C>A	NA	NA	NA	6.102	NA	NA	NA	NA	NA	0.00000
PALB2 16	23635333	A	T	rs201817103	NO	missense	ENST00000261584.4:c.2831T>A	ENSP00000261584.4:p.Ile944Asn	I944N	PTHR14662:SF2,PTHR14662,Superfamily_domains:SSF50978	31	probably_damaging	0.997	deleterious	0	15	0.00000
PALB2 16	23635348	A	C	CM105609,rs45478192	NO	missense	ENST00000261584.4:c.2816T>G	ENSP00000261584.4:p.Leu939Trp	L939W	PTHR14662:SF2,PTHR14662,Superfamily_domains:SSF50978	28	probably_damaging	1	deleterious	0	55	0.00318
PALB2 16	23635365	A	G	rs748232940	NO	synonymous	ENST00000261584.4:c.2799T>C	NA	C933C	PTHR14662:SF2,PTHR14662,Superfamily_domains:SSF50978	15.26	NA	NA	NA	NA	NA	0.00003
PALB2 16	23635370	C	T	rs45624036,CM112100	NO	missense	ENST00000261584.4:c.2794G>A	ENSP00000261584.4:p.Val932Met	V932M	PTHR14662:SF2,PTHR14662,Superfamily_domains:SSF50978	26.6	probably_damaging	0.995	tolerated	0.1	0	0.01617
PALB2 16	23635412	G	A	CM118268	NO	missense	ENST00000261584.4:c.2752C>T	ENSP00000261584.4:p.Pro918Ser	P918S	PTHR14662:SF2,PTHR14662,2.130.10.10,Superfamily_domains:SSF50978	27.9	probably_damaging	1	deleterious	0.04	0	0.00000
PALB2 16	23637527	G	A	NA	NO	intron	ENST00000261584.4:c.2748+30C>T	NA	NA	NA	6.547	NA	NA	NA	NA	NA	0.00000
PALB2 16	23637541	C	T	rs730881873	NO	intron	ENST00000261584.4:c.2748+16G>A	NA	NA	NA	6.241	NA	NA	NA	NA	NA	0.00000
PALB2 16	23637542	G	A	rs749756493	NO	intron	ENST00000261584.4:c.2748+15C>T	NA	NA	NA	1.334	NA	NA	NA	NA	NA	0.00000
PALB2 16	23637544	G	C	rs771355581	NO	intron	ENST00000261584.4:c.2748+13C>G	NA	NA	NA	1.231	NA	NA	NA	NA	NA	0.00000
PALB2 16	23637552	C	T	NA	NO	splice,intron	ENST00000261584.4:c.2748+5G>A	NA	NA	NA	11.85	NA	NA	NA	NA	NA	0.00000
PALB2 16	23637563	G	A	rs115759702	NO	synonymous	ENST00000261584.4:c.2742C>T	NA	F914F	PTHR14662:SF2,PTHR14662,2.130.10.10,Superfamily_domains:SSF50978	9.451	NA	NA	NA	NA	NA	0.00006
PALB2 16	23637581	T	A	NA	NO	missense	ENST00000261584.4:c.2724A>T	ENSP00000261584.4:p.Lys908Asn	K908N	PTHR14662:SF2,PTHR14662,2.130.10.10,Superfamily_domains:SSF50978	23.2	probably_damaging	0.998	tolerated	0.08	0	0.00000
PALB2 16	23637587	C	T	CM111013,rs180177122	YES	stop_gained	ENST00000261584.4:c.2718G>A	ENSP00000261584.4:p.Trp906Ter	W906*	PTHR14662:SF2,PTHR14662,2.130.10.10,Superfamily_domains:SSF50978	39	NA	NA	NA	NA	NA	0.00000
PALB2 16	23637631	C	T	rs45476495	NO	missense	ENST00000261584.4:c.2674G>A	ENSP00000261584.4:p.Glu892Lys	E892K	PTHR14662:SF2,PTHR14662,2.130.10.10,Superfamily_domains:SSF50978	33	probably_damaging	0.999	deleterious	0.05	0	0.00024
PALB2 16	23637631	C	A	rs45476495	YES	stop_gained	ENST00000261584.4:c.2674G>T	ENSP00000261584.4:p.Glu892Ter	E892*	PTHR14662:SF2,PTHR14662,2.130.10.10,Superfamily_domains:SSF50978	42	NA	NA	NA	NA	NA	0.00000
PALB2 16	23637632	G	A	rs78179744	NO	synonymous	ENST00000261584.4:c.2673C>T	NA	C891C	PTHR14662:SF2,PTHR14662,2.130.10.10,Superfamily_domains:SSF50978	9.786	NA	NA	NA	NA	NA	0.00000
PALB2 16	23637643	T	C	NA	NO	missense	ENST00000261584.4:c.2662A>G	ENSP00000261584.4:p.Ile888Val	I888V	PTHR14662:SF2,PTHR14662,2.130.10.10,Superfamily_domains:SSF50978	8.969	benign	0.034	tolerated	0.55	0	0.00000
PALB2 16	23637645	A	C	rs45550935	NO	missense	ENST00000261584.4:c.2660T>G	ENSP00000261584.4:p.Ile887Ser	I887S	PTHR14662:SF2,PTHR14662,2.130.10.10,Superfamily_domains:SSF50978	29.9	probably_damaging	1	deleterious	0	15	0.00000
PALB2 16	23637660	C	T	rs45486906	NO	missense	ENST00000261584.4:c.2645G>A	ENSP00000261584.4:p.Cys882Tyr	C882Y	PTHR14662:SF2,PTHR14662,2.130.10.10,Superfamily_domains:SSF50978	0.256	benign	0.002	tolerated	1	0	0.00000
PALB2 16	23637664	C	T	rs766315705	NO	missense	ENST00000261584.4:c.2641G>A	ENSP00000261584.4:p.Gly881Ser	G881S	PTHR14662:SF2,PTHR14662,2.130.10.10,Superfamily_domains:SSF50978	28.6	probably_damaging	0.949	tolerated	0.11	0	0.00003
PALB2 16	23637686	A	C	rs587782387	NO	missense	ENST00000261584.4:c.2619T>G	ENSP00000261584.4:p.Ser873Arg	S873R	PTHR14662:SF2,PTHR14662,2.130.10.10,Superfamily_domains:SSF50978	26.1	probably_damaging	0.998	deleterious	0.01	0	0.00000
PALB2 16	23637690	A	G	rs730881877	NO	missense	ENST00000261584.4:c.2615T>C	ENSP00000261584.4:p.Val872Ala	V872A	PTHR14662:SF2,PTHR14662,2.130.10.10,Superfamily_domains:SSF50978	27.3	probably_damaging	0.981	deleterious	0.02	0	0.00000
PALB2 16	23637698	G	A	rs767633741,rs45542234	NO	synonymous	ENST00000261584.4:c.2607C>T	NA	S869S	PTHR14662:SF2,PTHR14662	14.93	NA	NA	NA	NA	NA	0.00000
PALB2 16	23637698	G	T	rs767633741,rs45542234	NO	synonymous	ENST00000261584.4:c.2607C>A	NA	S869S	PTHR14662:SF2,PTHR14662	11.43	NA	NA	NA	NA	NA	0.00015
PALB2 16	23637699	G	C	rs779279139	NO	missense	ENST00000261584.4:c.2606C>G	ENSP00000261584.4:p.Ser869Cys	S869C	PTHR14662:SF2,PTHR14662	27.7	probably_damaging	0.999	deleterious	0.02	15	0.00003
PALB2 16	23637715	G	A	CM105610,rs45568339	NO	missense	ENST00000261584.4:c.2590C>T	ENSP00000261584.4:p.Pro864Ser	P864S	PTHR14662:SF2,PTHR14662	18.44	benign	0.348	tolerated	0.25	0	0.00806
PALB2 16	23637754	T	C	rs760914088	NO	intron	ENST00000261584.4:c.2587-36A>G	NA	NA	NA	4.187	NA	NA	NA	NA	NA	0.00000
PALB2 16	23637756	G	C	rs180177119	NO	intron	ENST00000261584.4:c.2587-38C>G	NA	NA	NA	1.441	NA	NA	NA	NA	NA	0.00000
PALB2 16	23637758	G	C	NA	NO	intron	ENST00000261584.4:c.2587-40C>G	NA	NA	NA	1.667	NA	NA	NA	NA	NA	0.00000
PALB2 16	23637777	A	C	rs371085248	NO	intron	ENST00000261584.4:c.2587-59T>G	NA	NA	NA	4.083	NA	NA	NA	NA	NA	0.00000
PALB2 16	23640467	G	A	rs249954	NO	intron	ENST00000261584.4:c.2586+58C>T	NA	NA	NA	5.159	NA	NA	NA	NA	NA	0.00000
PALB2 16	23640484	T	C	rs780327695	NO	intron	ENST00000261584.4:c.2586+41A>G	NA	NA	NA	4.922	NA	NA	NA	NA	NA	0.00000
PALB2 16	23640515	T	C	rs373321719	NO	intron	ENST00000261584.4:c.2586+10A>G	NA	NA	NA	11.84	NA	NA	NA	NA	NA	0.00000
PALB2 16	23640550	T	C	rs146455175	NO	missense	ENST00000261584.4:c.2561A>G	ENSP00000261584.4:p.Asn854Ser	N854S	PTHR14662:SF2,PTHR14662	0.003	benign	0.023	tolerated	0.4	0	0.00000
PALB2 16	23640589	GT	G	rs368990417,CD070510,rs180177116,PALB2:c.	YES	frameshift,feature_truncation	ENST00000261584.4:c.2521delA	ENSP00000261584.4:p.Thr841GlnfsTer10	T841X	PTHR14662:SF2,PTHR14662	22.7	NA	NA	NA	NA	NA	0.00000
PALB2 16	23640963	G	C	rs750650768	NO	missense,splice	ENST00000261584.4:c.2512C>G	ENSP00000261584.4:p.Gln838Glu	Q838E	PTHR14662:SF2,PTHR14662	23.3	probably_damaging	0.998	tolerated	0.12	0	0.00000
PALB2 16	23640967	G	A	NA	NO	synonymous	ENST00000261584.4:c.2508C>T	NA	V836V	PTHR14662:SF2,PTHR14662	6.117	NA	NA	NA	NA	NA	0.00000
PALB2 16	23640969	C	T	COSM417101	NO	missense	ENST00000261584.4:c.2506G>A	ENSP00000261584.4:p.Val836Ile	V836I	PTHR14662:SF2,PTHR14662	0.001	benign	0.002	tolerated	0.73	0	0.00012
PALB2 16	23640972	A	T	COSM3507838	NO	missense	ENST00000261584.4:c.2503T>A	ENSP00000261584.4:p.Ser835Thr	S835T	PTHR14662:SF2,PTHR14662	7.512	benign	0.297	tolerated	0.28	0	0.00000
PALB2 16	23640997	G	A	NA	NO	synonymous	ENST00000261584.4:c.2478C>T	NA	N826N	PTHR14662:SF2,PTHR14662	1.473	NA	NA	NA	NA	NA	0.00000
PALB2 16	23641022	A	G	NA	NO	missense	ENST00000261584.4:c.2453T>C	ENSP00000261584.4:p.Phe818Ser	F818S	PTHR14662:SF2,PTHR14662	0.594	benign	0.023	tolerated	0.4	0	0.00009
PALB2 16	23641033	C	T	rs140776736	NO	synonymous	ENST00000261584.4:c.2442G>A	NA	E814E	PTHR14662:SF2,PTHR14662	10.09	NA	NA	NA	NA	NA	0.00012
PALB2 16	23641037	A	G	rs763191051	NO	missense	ENST00000261584.4:c.2438T>C	ENSP00000261584.4:p.Ile813Thr	I813T	PTHR14662:SF2,PTHR14662	0.001	benign	0.002	tolerated	1	0	0.00000
PALB2 16	23641058	G	A	rs45464991	NO	missense	ENST00000261584.4:c.2417C>T	ENSP00000261584.4:p.Pro806Leu	P806L	PTHR14662:SF2,PTHR14662	4.978	benign	0.023	tolerated	0.41	0	0.00006
PALB2 16	23641089	C	A	CM070238,rs180177112	YES	stop_gained	ENST00000261584.4:c.2386G>T	ENSP00000261584.4:p.Gly796Ter	G796*	PTHR14662:SF2,PTHR14662	34	NA	NA	NA	NA	NA	0.00000
PALB2 16	23641096	G	A	rs377626805	NO	synonymous	ENST00000261584.4:c.2379C>T	NA	G793G	PTHR14662:SF2,PTHR14662	3.292	NA	NA	NA	NA	NA	0.00018
PALB2 16	23641146	C	T	rs148026749,COSM1219154	NO	missense	ENST00000261584.4:c.2329G>A	ENSP00000261584.4:p.Asp777Asn	D777N	PTHR14662:SF2,PTHR14662	0.002	benign	0.006	tolerated	0.84	0	0.00000
PALB2 16	23641147	G	A	rs45508997,COSM968811	NO	synonymous	ENST00000261584.4:c.2328C>T	NA	F776F	PTHR14662:SF2,PTHR14662	9.552	NA	NA	NA	NA	NA	0.00000
PALB2 16	23641149	A	AT	NA	YES	frameshift,feature_elongation	ENST00000261584.4:c.2325dupA	ENSP00000261584.4:p.Phe776IlefsTer26	-775-776X	PTHR14662:SF2,PTHR14662	23.1	NA	NA	NA	NA	NA	0.00000
PALB2 16	23641186	C	G	rs373478248	NO	missense	ENST00000261584.4:c.2289G>C	ENSP00000261584.4:p.Leu763Phe	L763F	PTHR14662:SF2,PTHR14662	17.95	possibly_damaging	0.897	tolerated	0.37	0	0.00000
PALB2 16	23641218	G	A	rs180177110,CM070237,PALB2:c.2257C>T	YES	stop_gained	ENST00000261584.4:c.2257C>T	ENSP00000261584.4:p.Arg753Ter	R753*	PTHR14662:SF2,PTHR14662	35	NA	NA	NA	NA	NA	0.00009
PALB2 16	23641219	T	C	rs147120218	NO	synonymous	ENST00000261584.4:c.2256A>G	NA	G752G	PTHR14662:SF2,PTHR14662	0.108	NA	NA	NA	NA	NA	0.00000
PALB2 16	23641231	T	C	rs750048627	NO	synonymous	ENST00000261584.4:c.2244A>G	NA	T748T	PTHR14662:SF2,PTHR14662	0.132	NA	NA	NA	NA	NA	0.00009
PALB2 16	23641275	T	A	rs45543843	NO	missense	ENST00000261584.4:c.2200A>T	ENSP00000261584.4:p.Thr734Ser	T734S	PTHR14662:SF2,PTHR14662	28.1	probably_damaging	1	deleterious	0	0	0.00009
PALB2 16	23641308	T	A	NA	NO	missense	ENST00000261584.4:c.2167A>T	ENSP00000261584.4:p.Met723Leu	M723L	PTHR14662:SF2,PTHR14662	0.002	benign	0.001	tolerated	0.65	0	0.00000
PALB2 16	23641314	T	TC	NA	YES	frameshift,feature_elongation	ENST00000261584.4:c.2160_2161insG	ENSP00000261584.4:p.Thr721AspfsTer24	-720-721X	PTHR14662:SF2,PTHR14662	23.3	NA	NA	NA	NA	NA	0.00000
PALB2 16	23641340	G	A	rs141458731	NO	missense	ENST00000261584.4:c.2135C>T	ENSP00000261584.4:p.Ala712Val	A712V	PTHR14662:SF2,PTHR14662	0.353	benign	0.009	tolerated	0.37	0	0.00075
PALB2 16	23641345	C	T	rs774049060,COSM4059424	NO	synonymous	ENST00000261584.4:c.2130G>A	NA	T710T	PTHR14662:SF2,PTHR14662	8.302	NA	NA	NA	NA	NA	0.00006
PALB2 16	23641351	T	A	NA	NO	missense	ENST00000261584.4:c.2124A>T	ENSP00000261584.4:p.Leu708Phe	L708F	PTHR14662:SF2,PTHR14662	0.158	benign	0.077	tolerated	0.19	0	0.00000
PALB2 16	23641369	T	C	rs730881886	NO	missense	ENST00000261584.4:c.2106A>G	ENSP00000261584.4:p.Ile702Met	I702M	PTHR14662:SF2,PTHR14662	0.003	benign	0.061	tolerated	0.3	0	0.00003
PALB2 16	23641372	G	A	rs753583700	NO	synonymous	ENST00000261584.4:c.2103C>T	NA	S701S	PTHR14662:SF2,PTHR14662	7.164	NA	NA	NA	NA	NA	0.00003
PALB2 16	23641375	T	A	rs757145884	NO	synonymous	ENST00000261584.4:c.2100A>T	NA	S700S	PTHR14662:SF2,PTHR14662	9.667	NA	NA	NA	NA	NA	0.00009
PALB2 16	23641382	A	T	NA	NO	missense	ENST00000261584.4:c.2093T>A	ENSP00000261584.4:p.Leu698His	L698H	PTHR14662:SF2,PTHR14662	23.8	probably_damaging	0.989	deleterious	0	0	0.00000
PALB2 16	23641384	G	T	NA	NO	synonymous	ENST00000261584.4:c.2091C>A	NA	G697G	PTHR14662:SF2,PTHR14662	0.953	NA	NA	NA	NA	NA	0.00000
PALB2 16	23641388	G	A	rs587780820	NO	missense	ENST00000261584.4:c.2087C>T	ENSP00000261584.4:p.Thr696Met	T696M	PTHR14662:SF2,PTHR14662	14.97	benign	0.424	deleterious	0.03	0	0.00000
PALB2 16	23641393	T	C	rs781440401	NO	synonymous	ENST00000261584.4:c.2082A>G	NA	T694T	PTHR14662:SF2,PTHR14662	0.031	NA	NA	NA	NA	NA	0.00015
PALB2 16	23641398	G	T	rs150585579	NO	missense	ENST00000261584.4:c.2077C>A	ENSP00000261584.4:p.His693Asn	H693N	PTHR14662:SF2,PTHR14662	0.016	benign	0.001	tolerated	0.38	0	0.00000
PALB2 16	23641422	TG	T	rs587782680	YES	frameshift,feature_truncation	ENST00000261584.4:c.2052delC	ENSP00000261584.4:p.Arg686GlyfsTer23	P684X	PTHR14662:SF2,PTHR14662	22.9	NA	NA	NA	NA	NA	0.00006
PALB2 16	23641461	C	G	CM112099,rs45532440	NO	missense	ENST00000261584.4:c.2014G>C	ENSP00000261584.4:p.Glu672Gln	E672Q	Low_complexity_(Seg):seg,PTHR14662:SF2,PTHR14662	8.057	benign	0.21	tolerated	0.19	0	0.05574
PALB2 16	23641552	T	C	rs775283927	NO	synonymous	ENST00000261584.4:c.1923A>G	NA	K641K	PTHR14662:SF2,PTHR14662	0.006	NA	NA	NA	NA	NA	0.00006
PALB2 16	23641584	AGCAGGACTT	A	rs587778583	NO	inframe_deletion	ENST00000261584.4:c.1882_1890delAAGTCCTGC	ENSP00000261584.4:p.Lys628_Cys630del	KSC628-630-	PTHR14662:SF2,PTHR14662	17.66	NA	NA	NA	NA	NA	0.00012
PALB2 16	23641632	G	A	NA	NO	missense	ENST00000261584.4:c.1843C>T	ENSP00000261584.4:p.Pro615Ser	P615S	PTHR14662:SF2,PTHR14662	31	probably_damaging	1	deleterious	0	65	0.00000
PALB2 16	23641674	TTA	T	NA	YES	frameshift,feature_truncation	ENST00000261584.4:c.1799_1800delTA	ENSP00000261584.4:p.Leu600Ter	L600X	PTHR14662:SF2,PTHR14662	24.6	NA	NA	NA	NA	NA	0.00000
PALB2 16	23641682	A	G	NA	NO	missense	ENST00000261584.4:c.1793T>C	ENSP00000261584.4:p.Leu598Pro	L598P	PTHR14662:SF2,PTHR14662	22	possibly_damaging	0.589	deleterious	0.01	0	0.00000
PALB2 16	23641727	A	C	rs587782151	NO	missense	ENST00000261584.4:c.1748T>G	ENSP00000261584.4:p.Leu583Trp	L583W	PTHR14662:SF2,PTHR14662	22.5	probably_damaging	0.997	deleterious	0.01	0	0.00009
PALB2 16	23641733	A	G	NA	NO	missense	ENST00000261584.4:c.1742T>C	ENSP00000261584.4:p.Leu581Ser	L581S	PTHR14662:SF2,PTHR14662	16.32	benign	0.406	deleterious	0.01	0	0.00000
PALB2 16	23641778	C	G	rs144617793,COSM1219152	NO	missense	ENST00000261584.4:c.1697G>C	ENSP00000261584.4:p.Arg566Pro	R566P	PTHR14662:SF2,PTHR14662	10.77	benign	0.14	tolerated	0.22	0	0.00004
PALB2 16	23641784	T	C	rs62625276	NO	missense	ENST00000261584.4:c.1691A>G	ENSP00000261584.4:p.Lys564Arg	K564R	PTHR14662:SF2,PTHR14662	6.557	benign	0.047	tolerated	0.5	0	0.00000
PALB2 16	23641785	T	C	NA	NO	missense	ENST00000261584.4:c.1690A>G	ENSP00000261584.4:p.Lys564Glu	K564E	PTHR14662:SF2,PTHR14662	0.06	benign	0	tolerated	0.78	0	0.00000
PALB2 16	23641797	A	C	NA	NO	splice,intron	ENST00000261584.4:c.1685-7T>G	NA	NA	NA	0.014	NA	NA	NA	NA	NA	0.00000
PALB2 16	23641819	G	A	rs773252502	NO	intron	ENST00000261584.4:c.1685-29C>T	NA	NA	NA	10.29	NA	NA	NA	NA	NA	0.00000
PALB2 16	23646105	G	GA	NA	NO	intron,feature_elongation	ENST00000261584.4:c.1684+77dupT	NA	NA	NA	0.185	NA	NA	NA	NA	NA	0.00000
PALB2 16	23646135	T	C	rs774211412	NO	intron	ENST00000261584.4:c.1684+48A>G	NA	NA	NA	3.761	NA	NA	NA	NA	NA	0.00000
PALB2 16	23646154	T	C	rs74320059	NO	intron	ENST00000261584.4:c.1684+29A>G	NA	NA	NA	7.42	NA	NA	NA	NA	NA	0.00000
PALB2 16	23646189	CT	C	NA	YES	frameshift,feature_truncation	ENST00000261584.4:c.1677delA	ENSP00000261584.4:p.Val560Ter	Q559X	PTHR14662:SF2,PTHR14662	23.4	NA	NA	NA	NA	NA	0.00000
PALB2 16	23646191	T	C	rs152451	NO	missense	ENST00000261584.4:c.1676A>G	ENSP00000261584.4:p.Gln559Arg	Q559R	PTHR14662:SF2,PTHR14662	0.001	benign	0.001	tolerated	0.79	0	0.18324
PALB2 16	23646202	T	C	rs45518838	NO	synonymous	ENST00000261584.4:c.1665A>G	NA	K555K	PTHR14662:SF2,PTHR14662	0.601	NA	NA	NA	NA	NA	0.00000
PALB2 16	23646230	A	G	rs148647206,COSM968815	NO	missense	ENST00000261584.4:c.1637T>C	ENSP00000261584.4:p.Val546Ala	V546A	PTHR14662:SF2,PTHR14662	15.46	possibly_damaging	0.716	tolerated	0.19	15	0.00006
PALB2 16	23646244	C	T	rs745665968	NO	synonymous	ENST00000261584.4:c.1623G>A	NA	R541R	PTHR14662:SF2,PTHR14662	10.31	NA	NA	NA	NA	NA	0.00003
PALB2 16	23646295	T	C	rs45472400	NO	synonymous	ENST00000261584.4:c.1572A>G	NA	S524S	PTHR14662:SF2,PTHR14662	0.365	NA	NA	NA	NA	NA	0.00818
PALB2 16	23646300	C	A	NA	NO	missense	ENST00000261584.4:c.1567G>T	ENSP00000261584.4:p.Ala523Ser	A523S	PTHR14662:SF2,PTHR14662	0.007	benign	0.096	tolerated	0.73	0	0.00000
PALB2 16	23646320	CT	C	rs587781560	YES	frameshift,feature_truncation	ENST00000261584.4:c.1546delA	ENSP00000261584.4:p.Arg516GlufsTer45	R516X	PTHR14662:SF2,PTHR14662	28.4	NA	NA	NA	NA	NA	0.00000
PALB2 16	23646375	C	A	rs75023630	NO	missense	ENST00000261584.4:c.1492G>T	ENSP00000261584.4:p.Asp498Tyr	D498Y	PTHR14662:SF2,PTHR14662	24.3	probably_damaging	0.927	deleterious	0.02	0	0.00000
PALB2 16	23646397	G	A	rs45612837	NO	synonymous	ENST00000261584.4:c.1470C>T	NA	P490P	PTHR14662:SF2,PTHR14662	9.513	NA	NA	NA	NA	NA	0.00084
PALB2 16	23646413	G	A	NA	NO	missense	ENST00000261584.4:c.1454C>T	ENSP00000261584.4:p.Thr485Ile	T485I	PTHR14662:SF2,PTHR14662	15.08	benign	0.16	deleterious	0.01	0	0.00000
PALB2 16	23646413	G	C	NA	NO	missense	ENST00000261584.4:c.1454C>G	ENSP00000261584.4:p.Thr485Ser	T485S	PTHR14662:SF2,PTHR14662	2.008	benign	0.177	tolerated	0.29	0	0.00000
PALB2 16	23646416	A	T	NA	YES	stop_gained	ENST00000261584.4:c.1451T>A	ENSP00000261584.4:p.Leu484Ter	L484*	PTHR14662:SF2,PTHR14662	27.6	NA	NA	NA	NA	NA	0.00000
PALB2 16	23646421	G	C	rs368759893	NO	synonymous	ENST00000261584.4:c.1446C>G	NA	L482L	PTHR14662:SF2,PTHR14662	2.684	NA	NA	NA	NA	NA	0.00003
PALB2 16	23646488	T	C	rs749494645	NO	missense	ENST00000261584.4:c.1379A>G	ENSP00000261584.4:p.Gln460Arg	Q460R	PTHR14662:SF2,PTHR14662	15.95	benign	0.012	deleterious	0.04	0	0.00000
PALB2 16	23646499	C	G	NA	NO	missense	ENST00000261584.4:c.1368G>C	ENSP00000261584.4:p.Glu456Asp	E456D	PTHR14662:SF2,PTHR14662	23.4	possibly_damaging	0.889	tolerated	0.06	0	0.00000
PALB2 16	23646520	T	C	rs587780205	NO	synonymous	ENST00000261584.4:c.1347A>G	NA	K449K	PTHR14662:SF2,PTHR14662	0.904	NA	NA	NA	NA	NA	0.00000
PALB2 16	23646524	CTTGCATCCTTAT	C	NA	YES	frameshift,feature_truncation	ENST00000261584.4:c.1329_1342delAAATAAGGATGCAA	ENSP00000261584.4:p.Asn444Ter	KNKDAS443-448KX	Low_complexity_(Seg):seg,PTHR14662:SF2,PTHR14662	32	NA	NA	NA	NA	NA	0.00000
PALB2 16	23646524	C	T	NA	NO	missense	ENST00000261584.4:c.1343G>A	ENSP00000261584.4:p.Ser448Asn	S448N	PTHR14662:SF2,PTHR14662	10.47	benign	0.003	tolerated	0.21	0	0.00000
PALB2 16	23646549	AC	A	TMP_ESP_16_23646550_23646550,CD107613	YES	frameshift,feature_truncation	ENST00000261584.4:c.1317delG	ENSP00000261584.4:p.Phe440LeufsTer12	G439X	Low_complexity_(Seg):seg,PTHR14662:SF2,PTHR14662	21.6	NA	NA	NA	NA	NA	0.00000
PALB2 16	23646595	G	A	rs754720030	NO	synonymous	ENST00000261584.4:c.1272C>T	NA	A424A	PTHR14662:SF2,PTHR14662	13.92	NA	NA	NA	NA	NA	0.00000
PALB2 16	23646617	G	T	rs45510998	NO	missense	ENST00000261584.4:c.1250C>A	ENSP00000261584.4:p.Ser417Tyr	S417Y	PTHR14662:SF2,PTHR14662	26.5	probably_damaging	1	deleterious	0	15	0.00042
PALB2 16	23646626	C	T	rs749461008	NO	missense	ENST00000261584.4:c.1241G>A	ENSP00000261584.4:p.Arg414Gln	R414Q	PTHR14662:SF2,PTHR14662	26.7	possibly_damaging	0.786	tolerated	0.06	35	0.00003
PALB2 16	23646627	G	A	CM106645,rs180177100	YES	stop_gained	ENST00000261584.4:c.1240C>T	ENSP00000261584.4:p.Arg414Ter	R414*	PTHR14662:SF2,PTHR14662	38	NA	NA	NA	NA	NA	0.00000
PALB2 16	23646673	C	T	rs61755173	NO	synonymous	ENST00000261584.4:c.1194G>A	NA	V398V	PTHR14662:SF2,PTHR14662	11.39	NA	NA	NA	NA	NA	0.00231
PALB2 16	23646678	T	A	rs367578415	NO	missense	ENST00000261584.4:c.1189A>T	ENSP00000261584.4:p.Thr397Ser	T397S	PTHR14662:SF2,PTHR14662	25.5	probably_damaging	1	deleterious	0	55	0.00003
PALB2 16	23646679	G	A	rs587780817	NO	synonymous	ENST00000261584.4:c.1188C>T	NA	C396C	PTHR14662:SF2,PTHR14662	6.053	NA	NA	NA	NA	NA	0.00006
PALB2 16	23646691	T	C	NA	NO	synonymous	ENST00000261584.4:c.1176A>G	NA	E392E	PTHR14662:SF2,PTHR14662	0.012	NA	NA	NA	NA	NA	0.00000
PALB2 16	23646711	T	C	rs148102335	NO	missense	ENST00000261584.4:c.1156A>G	ENSP00000261584.4:p.Thr386Ala	T386A	PTHR14662:SF2,PTHR14662	0.001	benign	0.006	tolerated	0.39	0	0.00006
PALB2 16	23646717	C	T	NA	NO	missense	ENST00000261584.4:c.1150G>A	ENSP00000261584.4:p.Glu384Lys	E384K	PTHR14662:SF2,PTHR14662	18.46	benign	0.209	tolerated	0.08	0	0.00000
PALB2 16	23646722	C	A	rs515726063	NO	missense	ENST00000261584.4:c.1145G>T	ENSP00000261584.4:p.Ser382Ile	S382I	PTHR14662:SF2,PTHR14662	18.43	benign	0.187	deleterious	0	15	0.00003
PALB2 16	23646742	T	C	rs767284930	NO	synonymous	ENST00000261584.4:c.1125A>G	NA	L375L	PTHR14662:SF2,PTHR14662	0.021	NA	NA	NA	NA	NA	0.00000
PALB2 16	23646772	C	T	CM118267	NO	synonymous	ENST00000261584.4:c.1095G>A	NA	R365R	PTHR14662:SF2,PTHR14662	6.099	NA	NA	NA	NA	NA	0.00003
PALB2 16	23646774	T	C	rs773001248	NO	missense	ENST00000261584.4:c.1093A>G	ENSP00000261584.4:p.Arg365Gly	R365G	PTHR14662:SF2,PTHR14662	0.833	benign	0.059	tolerated	0.14	0	0.00003
PALB2 16	23646788	T	C	NA	NO	missense	ENST00000261584.4:c.1079A>G	ENSP00000261584.4:p.Asp360Gly	D360G	PTHR14662:SF2,PTHR14662	4.004	benign	0.096	tolerated	0.05	0	0.00000
PALB2 16	23646809	TTC	T	rs180177099	YES	frameshift,feature_truncation	ENST00000261584.4:c.1056_1057delGA	ENSP00000261584.4:p.Lys353IlefsTer7	EK352-353EX	PTHR14662:SF2,PTHR14662	26	NA	NA	NA	NA	NA	0.00000
PALB2 16	23646825	G	T	rs375699023	NO	missense	ENST00000261584.4:c.1042C>A	ENSP00000261584.4:p.Gln348Lys	Q348K	PTHR14662:SF2,PTHR14662	10.84	benign	0.023	tolerated	0.27	0	0.00030
PALB2 16	23646837	T	C	NA	NO	missense	ENST00000261584.4:c.1030A>G	ENSP00000261584.4:p.Asn344Asp	N344D	PTHR14662:SF2,PTHR14662	13.61	possibly_damaging	0.457	tolerated	0.08	0	0.00000
PALB2 16	23646857	A	G	rs45494092,CM112098	NO	missense	ENST00000261584.4:c.1010T>C	ENSP00000261584.4:p.Leu337Ser	L337S	PTHR14662:SF2,PTHR14662	5.275	benign	0.056	tolerated	0.12	0	0.03808
PALB2 16	23646866	T	C	rs200620434	NO	missense	ENST00000261584.4:c.1001A>G	ENSP00000261584.4:p.Tyr334Cys	Y334C	PTHR14662:SF2,PTHR14662	0.068	benign	0.013	tolerated	0.13	0	0.00045
PALB2 16	23646887	C	T	NA	NO	missense	ENST00000261584.4:c.980G>A	ENSP00000261584.4:p.Cys327Tyr	C327Y	PTHR14662:SF2,PTHR14662	0.002	benign	0.022	tolerated	1	0	0.00000
PALB2 16	23646888	A	G	NA	NO	missense	ENST00000261584.4:c.979T>C	ENSP00000261584.4:p.Cys327Arg	C327R	PTHR14662:SF2,PTHR14662	0.014	benign	0.022	tolerated	0.33	0	0.00000
PALB2 16	23646942	T	C	rs3809683,COSM3957351	NO	missense	ENST00000261584.4:c.925A>G	ENSP00000261584.4:p.Ile309Val	I309V	PTHR14662:SF2,PTHR14662	0.001	benign	0.001	tolerated	1	0	0.00003
PALB2 16	23646947	T	G	COSM3690699	NO	missense	ENST00000261584.4:c.920A>C	ENSP00000261584.4:p.Lys307Thr	K307T	PTHR14662:SF2,PTHR14662	16.35	possibly_damaging	0.842	tolerated	0.07	0	0.00000
PALB2 16	23646958	G	A	rs145788619	NO	synonymous	ENST00000261584.4:c.909C>T	NA	L303L	PTHR14662:SF2,PTHR14662	0.229	NA	NA	NA	NA	NA	0.00000
PALB2 16	23646968	G	A	rs528541334,CM108903	NO	missense	ENST00000261584.4:c.899C>T	ENSP00000261584.4:p.Thr300Ile	T300I	PTHR14662:SF2,PTHR14662	4.678	benign	0.109	tolerated	0.06	0	0.00000
PALB2 16	23646980	AT	A	rs587776408,COSM1376814	YES	frameshift,feature_truncation	ENST00000261584.4:c.886delA	ENSP00000261584.4:p.Met296Ter	M296X	PTHR14662:SF2,PTHR14662	8.653	NA	NA	NA	NA	NA	0.00000
PALB2 16	23646987	T	C	rs753676934	NO	missense	ENST00000261584.4:c.880A>G	ENSP00000261584.4:p.Lys294Glu	K294E	PTHR14662:SF2,PTHR14662	1.603	benign	0.032	tolerated	0.07	0	0.00003
PALB2 16	23647023	T	C	rs45447991	NO	missense	ENST00000261584.4:c.844A>G	ENSP00000261584.4:p.Arg282Gly	R282G	PTHR14662:SF2,PTHR14662	7.986	benign	0.002	tolerated	0.07	0	0.00000
PALB2 16	23647038	C	T	rs778309339	NO	missense	ENST00000261584.4:c.829G>A	ENSP00000261584.4:p.Asp277Asn	D277N	PTHR14662:SF2,PTHR14662	0.151	benign	0.001	tolerated	0.53	0	0.00006
PALB2 16	23647044	T	A	NA	NO	missense	ENST00000261584.4:c.823A>T	ENSP00000261584.4:p.Thr275Ser	T275S	PTHR14662:SF2,PTHR14662	0.002	benign	0.034	tolerated	0.42	0	0.00000
PALB2 16	23647102	A	G	rs45465299	NO	synonymous	ENST00000261584.4:c.765T>C	NA	D255D	PTHR14662:SF2,PTHR14662	0.264	NA	NA	NA	NA	NA	0.00045
PALB2 16	23647108	T	TA	rs756660214	YES	frameshift,feature_elongation	ENST00000261584.4:c.758dupT	ENSP00000261584.4:p.Ser254IlefsTer3	L253LX	PTHR14662:SF2,PTHR14662	16.06	NA	NA	NA	NA	NA	0.00009
PALB2 16	23647127	G	C	rs587782658	NO	missense	ENST00000261584.4:c.740C>G	ENSP00000261584.4:p.Thr247Arg	T247R	PTHR14662:SF2,PTHR14662	13.61	possibly_damaging	0.595	tolerated	0.05	0	0.00000
PALB2 16	23647128	T	C	NA	NO	missense	ENST00000261584.4:c.739A>G	ENSP00000261584.4:p.Thr247Ala	T247A	PTHR14662:SF2,PTHR14662	0.001	benign	0.006	tolerated	0.89	0	0.00000
PALB2 16	23647133	G	A	rs571063157	NO	missense	ENST00000261584.4:c.734C>T	ENSP00000261584.4:p.Ala245Val	A245V	PTHR14662:SF2,PTHR14662	0.003	benign	0.002	tolerated	1	0	0.00000
PALB2 16	23647146	T	C	rs113217267	NO	missense	ENST00000261584.4:c.721A>G	ENSP00000261584.4:p.Asn241Asp	N241D	PTHR14662:SF2,PTHR14662	0.001	benign	0.003	tolerated	0.65	0	0.00003
PALB2 16	23647155	T	A	rs779278389	YES	stop_gained	ENST00000261584.4:c.712A>T	ENSP00000261584.4:p.Arg238Ter	R238*	PTHR14662:SF2,PTHR14662	32	NA	NA	NA	NA	NA	0.00000
PALB2 16	23647211	T	C	rs45594034	NO	missense	ENST00000261584.4:c.656A>G	ENSP00000261584.4:p.Asp219Gly	D219G	PTHR14662:SF2,PTHR14662	0.008	benign	0	tolerated	0.43	0	0.00048
PALB2 16	23647238	G	A	rs57605939	NO	missense	ENST00000261584.4:c.629C>T	ENSP00000261584.4:p.Pro210Leu	P210L	PTHR14662:SF2,PTHR14662	4.78	benign	0.045	tolerated	0.16	0	0.00015
PALB2 16	23647267	TA	T	rs587782081	YES	frameshift,feature_truncation	ENST00000261584.4:c.599delT	ENSP00000261584.4:p.Leu200Ter	L200X	PTHR14662:SF2,PTHR14662	21.4	NA	NA	NA	NA	NA	0.00003
PALB2 16	23647284	T	C	rs760784181	NO	missense	ENST00000261584.4:c.583A>G	ENSP00000261584.4:p.Ile195Val	I195V	PTHR14662:SF2,PTHR14662	0.001	benign	0.023	tolerated	0.74	0	0.00000
PALB2 16	23647296	G	C	rs587780221	NO	missense	ENST00000261584.4:c.571C>G	ENSP00000261584.4:p.Pro191Ala	P191A	PTHR14662:SF2,PTHR14662	9.656	possibly_damaging	0.846	tolerated	0.16	0	0.00000
PALB2 16	23647323	T	C	NA	NO	missense	ENST00000261584.4:c.544A>G	ENSP00000261584.4:p.Ile182Val	I182V	PTHR14662:SF2,PTHR14662	0.002	benign	0.004	tolerated	0.5	0	0.00000
PALB2 16	23647334	TC	T	NA	YES	frameshift,feature_truncation	ENST00000261584.4:c.532delG	ENSP00000261584.4:p.Glu178AsnfsTer15	E178X	PTHR14662:SF2,PTHR14662	22.7	NA	NA	NA	NA	NA	0.00000
PALB2 16	23647378	GAC	G	rs745533713	YES	frameshift,feature_truncation	ENST00000261584.4:c.487_488delGT	ENSP00000261584.4:p.Val163LeufsTer4	V163X	PTHR14662:SF2,PTHR14662	23.3	NA	NA	NA	NA	NA	0.00000
PALB2 16	23647405	T	A	NA	NO	synonymous	ENST00000261584.4:c.462A>T	NA	T154T	PTHR14662:SF2,PTHR14662	11.76	NA	NA	NA	NA	NA	0.00000
PALB2 16	23647416	G	A	rs587776407	YES	stop_gained	ENST00000261584.4:c.451C>T	ENSP00000261584.4:p.Gln151Ter	Q151*	PTHR14662:SF2,PTHR14662	26.2	NA	NA	NA	NA	NA	0.00000
PALB2 16	23647429	T	G	rs587781498	NO	missense	ENST00000261584.4:c.438A>C	ENSP00000261584.4:p.Arg146Ser	R146S	PTHR14662:SF2,PTHR14662	5.329	benign	0.407	tolerated	0.19	0	0.00000
PALB2 16	23647443	T	A	rs587782005	YES	stop_gained	ENST00000261584.4:c.424A>T	ENSP00000261584.4:p.Lys142Ter	K142*	PTHR14662:SF2,PTHR14662	34	NA	NA	NA	NA	NA	0.00003
PALB2 16	23647454	T	G	NA	NO	missense	ENST00000261584.4:c.413A>C	ENSP00000261584.4:p.Glu138Ala	E138A	PTHR14662:SF2,PTHR14662	23.5	possibly_damaging	0.713	deleterious	0.01	0	0.00000
PALB2 16	23647462	A	G	NA	NO	synonymous	ENST00000261584.4:c.405T>C	NA	P135P	PTHR14662:SF2,PTHR14662	0.03	NA	NA	NA	NA	NA	0.00000
PALB2 16	23647464	G	A	NA	NO	missense	ENST00000261584.4:c.403C>T	ENSP00000261584.4:p.Pro135Ser	P135S	PTHR14662:SF2,PTHR14662	2.406	benign	0.336	tolerated	0.07	0	0.00000
PALB2 16	23647465	G	A	NA	NO	synonymous	ENST00000261584.4:c.402C>T	NA	D134D	PTHR14662:SF2,PTHR14662	0.202	NA	NA	NA	NA	NA	0.00000
PALB2 16	23647473	C	T	NA	NO	missense	ENST00000261584.4:c.394G>A	ENSP00000261584.4:p.Val132Ile	V132I	PTHR14662:SF2,PTHR14662	0.96	benign	0.023	tolerated	0.49	0	0.00000
PALB2 16	23647514	A	G	rs370404126	NO	missense	ENST00000261584.4:c.353T>C	ENSP00000261584.4:p.Ile118Thr	I118T	PTHR14662:SF2,PTHR14662	0.062	benign	0.004	tolerated	0.24	0	0.00006
PALB2 16	23647518	G	T	NA	NO	missense	ENST00000261584.4:c.349C>A	ENSP00000261584.4:p.Pro117Thr	P117T	PTHR14662:SF2,PTHR14662	0.002	benign	0.19	tolerated	0.4	0	0.00000
PALB2 16	23647523	C	A	rs145598272,COSM3817575	NO	missense	ENST00000261584.4:c.344G>T	ENSP00000261584.4:p.Gly115Val	G115V	PTHR14662:SF2,PTHR14662	0.109	benign	0.009	tolerated	0.19	0	0.00021
PALB2 16	23647552	C	G	rs515726108	NO	missense	ENST00000261584.4:c.315G>C	ENSP00000261584.4:p.Glu105Asp	E105D	PTHR14662:SF2,PTHR14662	12.52	benign	0.066	deleterious	0.02	0	0.00009
PALB2 16	23647569	G	A	CM112097,rs61756147	NO	missense	ENST00000261584.4:c.298C>T	ENSP00000261584.4:p.Leu100Phe	L100F	PTHR14662:SF2,PTHR14662	0.056	benign	0.019	tolerated	0.18	0	0.00072
PALB2 16	23647575	T	C	rs587782831	NO	missense	ENST00000261584.4:c.292A>G	ENSP00000261584.4:p.Ile98Val	I98V	PTHR14662:SF2,PTHR14662	0.001	benign	0	tolerated	1	0	0.00003
PALB2 16	23647599	C	A	NA	YES	stop_gained	ENST00000261584.4:c.268G>T	ENSP00000261584.4:p.Glu90Ter	E90*	PTHR14662:SF2,PTHR14662	26.3	NA	NA	NA	NA	NA	0.00000
PALB2 16	23649159	G	A	NA	NO	intron	ENST00000261584.4:c.211+12C>T	NA	NA	NA	5.451	NA	NA	NA	NA	NA	0.00000
PALB2 16	23649186	G	A	CM111012,rs180177083	YES	stop_gained	ENST00000261584.4:c.196C>T	ENSP00000261584.4:p.Gln66Ter	Q66*	PTHR14662:SF2,PTHR14662	33	NA	NA	NA	NA	NA	0.00000
PALB2 16	23649187	C	T	rs751176316,COSM1376816	NO	synonymous	ENST00000261584.4:c.195G>A	NA	P65P	PTHR14662:SF2,PTHR14662	10.36	NA	NA	NA	NA	NA	0.00006
PALB2 16	23649188	G	A	rs62625272	NO	missense	ENST00000261584.4:c.194C>T	ENSP00000261584.4:p.Pro65Leu	P65L	PTHR14662:SF2,PTHR14662	0.017	benign	0.014	tolerated	0.78	0	0.00012
PALB2 16	23649219	G	A	NA	YES	stop_gained	ENST00000261584.4:c.163C>T	ENSP00000261584.4:p.Gln55Ter	Q55*	PTHR14662:SF2,PTHR14662	34	NA	NA	NA	NA	NA	0.00000
PALB2 16	23649266	TG	T	NA	YES	frameshift,feature_truncation	ENST00000261584.4:c.115delC	ENSP00000261584.4:p.Gln39LysfsTer14	Q39X	Coiled-coils_(Ncoils):Coil,PTHR14662:SF2,PTHR14662	26.1	NA	NA	NA	NA	NA	0.00000
PALB2 16	23649272	C	T	rs202194596	NO	missense,splice	ENST00000261584.4:c.110G>A	ENSP00000261584.4:p.Arg37His	R37H	Coiled-coils_(Ncoils):Coil,PTHR14662:SF2,PTHR14662	27.9	probably_damaging	0.993	deleterious	0.01	25	0.00006
PALB2 16	23649357	A	G	rs750296674	NO	intron	ENST00000261584.4:c.108+34T>C	NA	NA	NA	0.13	NA	NA	NA	NA	NA	0.00000
PALB2 16	23649399	G	A	NA	NO	missense	ENST00000261584.4:c.100C>T	ENSP00000261584.4:p.Arg34Cys	R34C	Coiled-coils_(Ncoils):Coil,PTHR14662:SF2,PTHR14662	34	probably_damaging	1	deleterious	0	65	0.00003

PALB2 16	23649405	G	C	rs151316635	NO	missense	ENST00000261584.4:c.94C>G	ENSP00000261584.4:p.Leu32Val	L32V	Coiled-coils_(Ncoils):Coil,PTHR14662:SF2,PTHR14662	24.2	probably_damaging	0.998	deleterious	0	0	0.00003
PALB2 16	23649415	G	A	rs761533286	NO	synonymous	ENST00000261584.4:c.84C>T	NA	Y28Y	Coiled-coils_(Ncoils):Coil,PTHR14662:SF2,PTHR14662	4.639	NA	NA	NA	NA	NA	0.00006
PALB2 16	23649428	A	G	NA	NO	missense	ENST00000261584.4:c.71T>C	ENSP00000261584.4:p.Leu24Ser	L24S	Low_complexity_(Seg):seg,Coiled-coils_(Ncoils):Coil,PTHR14662:SF2,PTHR14662	27.9	probably_damaging	1	deleterious	0	65	0.00000
PALB2 16	23649435	C	A	NA	NO	missense	ENST00000261584.4:c.64G>T	ENSP00000261584.4:p.Ala22Ser	A22S	Low_complexity_(Seg):seg,Coiled-coils_(Ncoils):Coil,PTHR14662:SF2,PTHR14662	25.6	probably_damaging	1	tolerated	0.06	0	0.00000
PALB2 16	23649446	T	C	rs138789658	NO	missense	ENST00000261584.4:c.53A>G	ENSP00000261584.4:p.Lys18Arg	K18R	Low_complexity_(Seg):seg,Coiled-coils_(Ncoils):Coil,PTHR14662:SF2,PTHR14662	26.2	probably_damaging	1	deleterious	0	0	0.00012
PALB2 16	23652352	C	T	NA	NO	regulatory	ENST00000261584.4:c.48+79G>A	NA	NA	NA	6.673	NA	NA	NA	NA	NA	0.00000
PALB2 16	23652364	GC	G	NA	NO	intron	ENST00000261584.4:c.48+66delG	NA	NA	NA	8.117	NA	NA	NA	NA	NA	0.00000
PALB2 16	23652369	G	A	NA	NO	regulatory	ENST00000261584.4:c.48+62C>T	NA	NA	NA	6.356	NA	NA	NA	NA	NA	0.00000
PALB2 16	23652388	C	A	rs758587524	NO	regulatory	ENST00000261584.4:c.48+43G>T	NA	NA	NA	9.567	NA	NA	NA	NA	NA	0.00000
PALB2 16	23652391	G	A	rs747570989	NO	regulatory	ENST00000261584.4:c.48+40C>T	NA	NA	NA	12	NA	NA	NA	NA	NA	0.00000
PALB2 16	23652392	G	T	rs768839390	NO	regulatory	ENST00000261584.4:c.48+39C>A	NA	NA	NA	10.39	NA	NA	NA	NA	NA	0.00000
PALB2 16	23652404	C	A	rs515726119	NO	regulatory	ENST00000261584.4:c.48+27G>T	NA	NA	NA	17.6	NA	NA	NA	NA	NA	0.00000
PALB2 16	23652405	G	A	rs748775669	NO	regulatory	ENST00000261584.4:c.48+26C>T	NA	NA	NA	13.71	NA	NA	NA	NA	NA	0.00000
PALB2 16	23652405	G	T	rs748775669	NO	regulatory	ENST00000261584.4:c.48+26C>A	NA	NA	NA	12.87	NA	NA	NA	NA	NA	0.00000
PALB2 16	23652410	C	T	rs770451617	NO	regulatory	ENST00000261584.4:c.48+21G>A	NA	NA	NA	20.6	NA	NA	NA	NA	NA	0.00000
PALB2 16	23652442	C	T	rs373287455	NO	missense	ENST00000261584.4:c.37G>A	ENSP00000261584.4:p.Glu13Lys	E13K	Low_complexity_(Seg):seg,PTHR14662:SF2,PTHR14662	32	probably_damaging	1	tolerated	0.07	0	0.00003
PALB2 16	23652464	G	A	COSM358998	NO	synonymous	ENST00000261584.4:c.15C>T	NA	P5P	PTHR14662:SF2,PTHR14662	17.11	NA	NA	NA	NA	NA	0.00000
PALB2 16	23652466	G	A	rs377085677,CM118266	NO	missense	ENST00000261584.4:c.13C>T	ENSP00000261584.4:p.Pro5Ser	P5S	PTHR14662:SF2,PTHR14662	8.301	benign	0.046	tolerated	0.55	0	0.00010
PALB2 16	23652467	A	G	rs567706422	NO	synonymous	ENST00000261584.4:c.12T>C	NA	P4P	PTHR14662:SF2,PTHR14662	11.11	NA	NA	NA	NA	NA	0.00029
PALB2 16	23652468	G	A	rs45619737	NO	missense	ENST00000261584.4:c.11C>T	ENSP00000261584.4:p.Pro4Leu	P4L	PTHR14662:SF2,PTHR14662	22.9	possibly_damaging	0.589	tolerated	0.16	0	0.00026
PALB2 16	23652478	T	C	NA	NO	initiator_codon	ENST00000261584.4:c.1A>G	ENSP00000261584.4:p.Met1?	M1V	NA	22.3	benign	0.129	deleterious	0	NA	0.00000
XRCC27	152345726	A	G	rs3218539	NO	3_prime_UTR	ENST00000359321.1:c.*1T>C	NA	NA	NA	12.68	NA	NA	NA	NA	NA	0.00000
XRCC27	152345759	TAAAA	T	NA	YES	frameshift,feature_truncation	ENST00000359321.1:c.807_810delTTTT	ENSP00000352271.1:p.Phe269LeufsTer27	FF269-270X	PTHR22942:SF5,PTHR22942,3.40.50.300,Superfamily_domains:SSF52540	35	NA	NA	NA	NA	NA	0.00000
XRCC27	152345759	T	C	rs587780132	NO	missense	ENST00000359321.1:c.811A>G	ENSP00000352271.1:p.Ile271Val	I271V	PTHR22942:SF5,PTHR22942,3.40.50.300,Superfamily_domains:SSF52540	2.043	benign	0.003	tolerated	0.64	0	0.00003
XRCC27	152345762	A	C	rs145085742	NO	missense	ENST00000359321.1:c.808T>G	ENSP00000352271.1:p.Phe270Val	F270V	PTHR22942:SF5,PTHR22942,3.40.50.300,Superfamily_domains:SSF52540	24.8	possibly_damaging	0.728	deleterious	0	45	0.00000
XRCC27	152345766	AT	A	rs776336749	YES	frameshift,feature_truncation	ENST00000359321.1:c.803delA	ENSP00000352271.1:p.His268LeufsTer29	H268X	PTHR22942:SF5,PTHR22942,3.40.50.300,Superfamily_domains:SSF52540	28.3	NA	NA	NA	NA	NA	0.00000
XRCC27	152345797	C	T	rs149186933,COSM206351	NO	missense	ENST00000359321.1:c.773G>A	ENSP00000352271.1:p.Arg258His	R258H	PTHR22942:SF5,PTHR22942,3.40.50.300,Superfamily_domains:SSF52540	11.03	benign	0.002	tolerated	0.57	0	0.00003
XRCC27	152345798	G	A	rs759300252	NO	missense	ENST00000359321.1:c.772C>T	ENSP00000352271.1:p.Arg258Cys	R258C	PTHR22942:SF5,PTHR22942,3.40.50.300,Superfamily_domains:SSF52540	18.26	benign	0.001	tolerated	0.21	0	0.00000
XRCC27	152345826	T	G	rs750589771	NO	missense	ENST00000359321.1:c.744A>C	ENSP00000352271.1:p.Gln248His	Q248H	PTHR22942:SF5,PTHR22942,3.40.50.300,Superfamily_domains:SSF52540	0.218	benign	0.001	tolerated	0.13	0	0.00009
XRCC27	152345840	G	A	NA	YES	stop_gained	ENST00000359321.1:c.730C>T	ENSP00000352271.1:p.Gln244Ter	Q244*	PTHR22942:SF5,PTHR22942,3.40.50.300,Superfamily_domains:SSF52540	35	NA	NA	NA	NA	NA	0.00000
XRCC27	152345858	T	A	NA	NO	missense	ENST00000359321.1:c.712A>T	ENSP00000352271.1:p.Arg238Trp	R238W	PTHR22942:SF5,PTHR22942,3.40.50.300,Superfamily_domains:SSF52540	26.9	probably_damaging	1	deleterious	0	65	0.00000
XRCC27	152345901	G	A	rs374213093	NO	synonymous	ENST00000359321.1:c.669C>T	NA	Y223Y	PTHR22942:SF5,PTHR22942,3.40.50.300,Superfamily_domains:SSF52540	0.007	NA	NA	NA	NA	NA	0.00003
XRCC27	152345903	A	G	rs587780130	NO	missense	ENST00000359321.1:c.667T>C	ENSP00000352271.1:p.Tyr223His	Y223H	PTHR22942:SF5,PTHR22942,3.40.50.300,Superfamily_domains:SSF52540	17.79	probably_damaging	0.999	tolerated	0.05	0	0.00009
XRCC27	152345910	G	A	NA	NO	synonymous	ENST00000359321.1:c.660C>T	NA	D220D	PTHR22942:SF5,PTHR22942,3.40.50.300,Superfamily_domains:SSF52540	0.03	NA	NA	NA	NA	NA	0.00000
XRCC27	152345917	TCA	T	rs746142129	YES	frameshift,feature_truncation	ENST00000359321.1:c.651_652delTG	ENSP00000352271.1:p.Cys217Ter	CD217-218*	PTHR22942:SF5,PTHR22942,3.40.50.300,Superfamily_domains:SSF52540	22.9	NA	NA	NA	NA	NA	0.00000
XRCC27	152345927	G	A	rs143153871	YES	stop_gained	ENST00000359321.1:c.643C>T	ENSP00000352271.1:p.Arg215Ter	R215*	PTHR22942:SF5,PTHR22942,3.40.50.300,Superfamily_domains:SSF52540	34	NA	NA	NA	NA	NA	0.00015
XRCC27	152345930	G	A	NA	YES	stop_gained	ENST00000359321.1:c.640C>T	ENSP00000352271.1:p.Arg214Ter	R214*	PTHR22942:SF5,PTHR22942,3.40.50.300,Superfamily_domains:SSF52540	34	NA	NA	NA	NA	NA	0.00000
XRCC27	152345945	G	T	rs780202744	NO	missense	ENST00000359321.1:c.625C>A	ENSP00000352271.1:p.Pro209Thr	P209T	Low_complexity_(Seg):seg,PTHR22942:SF5,PTHR22942,3.40.50.300,Superfamily_domains:SSF5	0.002	benign	0.186	tolerated	0.6	0	0.00003
XRCC27	152345950	T	C	rs61762969	NO	missense	ENST00000359321.1:c.620A>G	ENSP00000352271.1:p.Glu207Gly	E207G	Low_complexity_(Seg):seg,PTHR22942:SF5,PTHR22942,3.40.50.300,Superfamily_domains:SSF5	8.437	benign	0.134	tolerated	0.21	0	0.00036
XRCC27	152345958	G	C	rs772419235	NO	missense	ENST00000359321.1:c.612C>G	ENSP00000352271.1:p.Ser204Arg	S204R	Low_complexity_(Seg):seg,PTHR22942:SF5,PTHR22942,Pfam_domain:PF08423,3.40.50.300,Sup	0.007	benign	0.049	tolerated	0.32	0	0.00003
XRCC27	152345965	G	A	NA	NO	missense	ENST00000359321.1:c.605C>T	ENSP00000352271.1:p.Ala202Val	A202V	Low_complexity_(Seg):seg,PTHR22942:SF5,PTHR22942,Pfam_domain:PF08423,3.40.50.300,Sup	2.161	benign	0.243	tolerated	0.54	0	0.00000
XRCC27	152345973	C	T	NA	NO	missense	ENST00000359321.1:c.597G>A	ENSP00000352271.1:p.Met199Ile	M199I	PTHR22942:SF5,PTHR22942,Pfam_domain:PF08423,3.40.50.300,Superfamily_domains:SSF5254	23.1	possibly_damaging	0.676	tolerated	0.2	0	0.00000
XRCC27	152345989	G	A	rs775565256,COSM1488418	NO	missense	ENST00000359321.1:c.581C>T	ENSP00000352271.1:p.Thr194Met	T194M	PROSITE_profiles:PS50162,PTHR22942:SF5,PTHR22942,Pfam_domain:PF08423,3.40.50.300,Su	26.6	probably_damaging	0.982	deleterious	0	15	0.00000
XRCC27	152345993	C	G	NA	NO	missense	ENST00000359321.1:c.577G>C	ENSP00000352271.1:p.Ala193Pro	A193P	PROSITE_profiles:PS50162,PTHR22942:SF5,PTHR22942,Pfam_domain:PF08423,3.40.50.300,Su	25.6	probably_damaging	0.969	deleterious	0.01	25	0.00000
XRCC27	152346007	C	T	rs3218536,CM021368	NO	missense	ENST00000359321.1:c.563G>A	ENSP00000352271.1:p.Arg188His	R188H	PROSITE_profiles:PS50162,PTHR22942:SF5,PTHR22942,Pfam_domain:PF08423,3.40.50.300,Su	15.95	benign	0.015	tolerated	0.28	0	0.15267
XRCC27	152346043	C	T	NA	NO	missense	ENST00000359321.1:c.527G>A	ENSP00000352271.1:p.Cys176Tyr	C176Y	PROSITE_profiles:PS50162,PTHR22942:SF5,PTHR22942,Pfam_domain:PF08423,3.40.50.300,Su	26.3	probably_damaging	1	deleterious	0.01	0	0.00006
XRCC27	152346061	T	G	rs778143946	NO	missense	ENST00000359321.1:c.509A>C	ENSP00000352271.1:p.Glu170Ala	E170A	PROSITE_profiles:PS50162,PTHR22942:SF5,PTHR22942,Pfam_domain:PF08423,3.40.50.300,Su	27.2	probably_damaging	1	deleterious	0.03	0	0.00006
XRCC27	152346080	C	A	NA	YES	stop_gained	ENST00000359321.1:c.490G>T	ENSP00000352271.1:p.Glu164Ter	E164*	PROSITE_profiles:PS50162,PTHR22942:SF5,PTHR22942,Pfam_domain:PF08423,3.40.50.300,Su	38	NA	NA	NA	NA	NA	0.00000
XRCC27	152346088	T	C	NA	NO	missense	ENST00000359321.1:c.482A>G	ENSP00000352271.1:p.Asn161Ser	N161S	PROSITE_profiles:PS50162,PTHR22942:SF5,PTHR22942,Pfam_domain:PF08423,3.40.50.300,Su	8.981	benign	0.303	tolerated	0.46	0	0.00000
XRCC27	152346093	G	A	rs202226401,COSM1087678	NO	synonymous	ENST00000359321.1:c.477C>T	NA	R159R	PROSITE_profiles:PS50162,PTHR22942:SF5,PTHR22942,Pfam_domain:PF08423,3.40.50.300,Su	7.052	NA	NA	NA	NA	NA	0.00021
XRCC27	152346094	C	T	rs367696886	NO	missense	ENST00000359321.1:c.476G>A	ENSP00000352271.1:p.Arg159His	R159H	PROSITE_profiles:PS50162,PTHR22942:SF5,PTHR22942,Pfam_domain:PF08423,3.40.50.300,Su	32	probably_damaging	1	deleterious	0	25	0.00009
XRCC27	152346095	G	A	rs587780128	NO	missense	ENST00000359321.1:c.475C>T	ENSP00000352271.1:p.Arg159Cys	R159C	PROSITE_profiles:PS50162,PTHR22942:SF5,PTHR22942,Pfam_domain:PF08423,3.40.50.300,Su	32	probably_damaging	1	deleterious	0	65	0.00000
XRCC27	152346114	T	G	rs758944840	NO	synonymous	ENST00000359321.1:c.456A>C	NA	S152S	PROSITE_profiles:PS50162,PTHR22942:SF5,PTHR22942,Pfam_domain:PF08423,3.40.50.300,Su	5.511	NA	NA	NA	NA	NA	0.00000
XRCC27	152346120	G	C	rs371453207	NO	missense	ENST00000359321.1:c.450C>G	ENSP00000352271.1:p.Ser150Arg	S150R	PROSITE_profiles:PS50162,PTHR22942:SF5,PTHR22942,Pfam_domain:PF08423,3.40.50.300,Su	25.3	probably_damaging	0.969	deleterious	0	65	0.00015
XRCC27	152346212	A	G	NA	NO	missense	ENST00000359321.1:c.358T>C	ENSP00000352271.1:p.Cys120Arg	C120R	PROSITE_profiles:PS50162,PTHR22942:SF5,PTHR22942,Pfam_domain:PF08423,3.40.50.300,Su	23.6	probably_damaging	1	deleterious	0	0	0.00000
XRCC27	152346216	C	T	rs558977371	NO	synonymous	ENST00000359321.1:c.354G>A	NA	V118V	PROSITE_profiles:PS50162,PTHR22942:SF5,PTHR22942,Pfam_domain:PF08423,3.40.50.300,Su	3.397	NA	NA	NA	NA	NA	0.00000
XRCC27	152346227	A	T	NA	NO	missense	ENST00000359321.1:c.343T>A	ENSP00000352271.1:p.Phe115Ile	F115I	PROSITE_profiles:PS50162,PTHR22942:SF5,PTHR22942,Pfam_domain:PF08423,3.40.50.300,Su	0.033	benign	0.101	tolerated	0.06	0	0.00000
XRCC27	152346229	C	T	rs779741034	NO	missense	ENST00000359321.1:c.341G>A	ENSP00000352271.1:p.Arg114Lys	R114K	PROSITE_profiles:PS50162,PTHR22942:SF5,PTHR22942,Pfam_domain:PF08423,3.40.50.300,Su	24.2	probably_damaging	0.999	deleterious	0	0	0.00003
XRCC27	152346270	T	C	rs146700851	NO	synonymous	ENST00000359321.1:c.300A>G	NA	L100L	PROSITE_profiles:PS50162,PTHR22942:SF5,PTHR22942,Pfam_domain:PF08423,3.40.50.300,Su	0.016	NA	NA	NA	NA	NA	0.00000
XRCC27	152346284	G	T	rs778698188	NO	missense	ENST00000359321.1:c.286C>A	ENSP00000352271.1:p.Leu96Ile	L96I	PROSITE_profiles:PS50162,PTHR22942:SF5,PTHR22942,Pfam_domain:PF08423,3.40.50.300,Su	24.5	probably_damaging	0.999	deleterious	0.01	0	0.00000
XRCC27	152346287	T	C	rs140214637	NO	missense	ENST00000359321.1:c.283A>G	ENSP00000352271.1:p.Ile95Val	I95V	PROSITE_profiles:PS50162,PTHR22942:SF5,PTHR22942,Pfam_domain:PF08423,3.40.50.300,Su	0.001	benign	0.036	tolerated	0.18	0	0.00117
XRCC27	152346288	T	C	rs376598559	NO	synonymous	ENST00000359321.1:c.282A>G	NA	T94T	PROSITE_profiles:PS50162,PTHR22942:SF5,PTHR22942,Pfam_domain:PF08423,3.40.50.300,Su	0.004	NA	NA	NA	NA	NA	0.00009
XRCC27	152346299	G	A	rs730882043	NO	missense	ENST00000359321.1:c.271C>T	ENSP00000352271.1:p.Arg91Trp	R91W	PROSITE_profiles:PS50162,PTHR22942:SF5,PTHR22942,Pfam_domain:PF08423,3.40.50.300,Su	26.9	probably_damaging	1	deleterious	0	65	0.00003
XRCC27	152346322	G	GT	NA	YES	frameshift,feature_elongation	ENST00000359321.1:c.247dupA	ENSP00000352271.1:p.Thr83AsnfsTer6	T83NX	PROSITE_profiles:PS50162,PTHR22942:SF5,PTHR22942,Pfam_domain:PF08423,3.40.50.300,Su	27.3	NA	NA	NA	NA	NA	0.00000
XRCC27	152346322	G	A	NA	NO	missense	ENST00000359321.1:c.248C>T	ENSP00000352271.1:p.Thr83Ile	T83I	PROSITE_profiles:PS50162,PTHR22942:SF5,PTHR22942,Pfam_domain:PF08423,3.40.50.300,Su	22.6	probably_damaging	1	tolerated	0.25	15	0.00000
XRCC27	152346345	T	C	rs751651780	NO	synonymous	ENST00000359321.1:c.225A>G	NA	E75E	PROSITE_profiles:PS50162,PTHR22942:SF5,PTHR22942,Pfam_domain:PF08423,3.40.50.300,Su	0.684	NA	NA	NA	NA	NA	0.00018
XRCC27	152346380	G	A	rs151110146	YES	stop_gained	ENST00000359321.1:c.190C>T	ENSP00000352271.1:p.Arg64Ter	R64*	PROSITE_profiles:PS50162,PTHR22942:SF5,PTHR22942,Pfam_domain:PF08423,3.40.50.300,Su	38	NA	NA	NA	NA	NA	0.00009
XRCC27	152346449	C	T	rs201836415	YES	splice_acceptor	ENST00000359321.1:c.122-1G>A	NA	NA	NA	24.8	NA	NA	NA	NA	NA	0.00000
XRCC27	152357788	T	C	rs777753452	NO	missense,splice	ENST00000359321.1:c.119A>G	ENSP00000352271.1:p.His40Arg	H40R	PROSITE_profiles:PS50162,PTHR22942:SF5,PTHR22942,3.40.50.300,Superfamily_domains:SSF:	21	possibly_damaging	0.529	tolerated	0.2	0	0.00000
XRCC27	152357792	C	T	rs730882040	NO	missense	ENST00000359321.1:c.115G>A	ENSP00000352271.1:p.Val39Met	V39M	PROSITE_profiles:PS50162,PTHR22942:SF5,PTHR22942	22.2	benign	0.33	tolerated	0.11	0	0.00003
XRCC27	152357842	C	T	NA	NO	missense	ENST00000359321.1:c.65G>A	ENSP00000352271.1:p.Ser22Asn	S22N	PROSITE_profiles:PS50162	22.7	benign	0.357	tolerated	0.17	0	0.00000
XRCC27	152357852	C	T	rs766826828	NO	missense	ENST00000359321.1:c.55G>A	ENSP00000352271.1:p.Glu19Lys	E19K	PROSITE_profiles:PS50162	27.3	probably_damaging	0.998	tolerated	0.06	0	0.00000
XRCC27	152357861	C	G	rs4987090	NO	missense	ENST00000359321.1:c.46G>C	ENSP00000352271.1:p.Ala16Pro	A16P	PROSITE_profiles:PS50162	28.9	probably_damaging	0.965	deleterious	0.01	0	0.00003
XRCC27	152373062	G	C	NA	NO	regulatory	ENST00000359321.1:c.39+64C>G	NA	NA	NA	5.93	NA	NA	NA	NA	NA	0.00000
XRCC27	152373083	G	A	rs764865989	NO	regulatory	ENST00000359321.1:c.39+43C>T	NA	NA	NA	7.786	NA	NA	NA	NA	NA	0.00000
XRCC27	152373090	T	C	NA	NO	regulatory	ENST00000359321.1:c.39+36A>G	NA	NA	NA	5.639	NA	NA	NA	NA	NA	0.00000
XRCC27	152373096	C	A	rs751787818	NO	regulatory	ENST00000359321.1:c.39+30G>T	NA	NA	NA	11.68	NA	NA	NA	NA	NA	0.00000
XRCC27	152373100	C	G	NA	NO	regulatory	ENST00000359321.1:c.39+26G>C	NA	NA	NA	9.588	NA	NA	NA	NA	NA	0.00000
XRCC27	152373100	C	T	NA	NO	regulatory	ENST00000359321.1:c.39+26G>A	NA	NA	NA	10.61	NA	NA	NA	NA	NA	0.00000
XRCC27	152373114	G	A	NA	NO	regulatory	ENST00000359321.1:c.39+12C>T	NA	NA	NA	15.6	NA	NA	NA	NA	NA	0.00000
XRCC27	152373121	C	G	NA	NO	splice,intron	ENST00000359321.1:c.39+5G>C	NA	NA	NA	20.5	NA	NA	NA	NA	NA	0.00000
XRCC27	152373125	C	T	rs560785131	YES	splice_donor	ENST00000359321.1:c.39+1G>A	NA	NA	NA	26.4	NA	NA	NA	NA	NA	0.00006
XRCC27	152373150	G	T	NA	NO	missense	ENST00000359321.1:c.15C>A	ENSP00000352271.1:p.Phe5Leu	F5L	NA	16.81	benign	0.004	tolerated	0.26	0	0.00000
XRCC27	152373165	C	T	rs768232997	NO	regulatory	ENST00000359321.1:c.-1G>A	NA	NA	NA	19.6	NA	NA	NA	NA	NA	0.00000
XRCC27	152373174	G	A	rs377067415	NO	regulatory	ENST00000359321.1:c.-10C>T	NA	NA	NA	13.39	NA	NA	NA	NA	NA	0.00000
XRCC27	152373178	C	T	rs750338805	NO	regulatory	ENST00000359321.1:c.-14G>A	NA	NA	NA	13.03	NA	NA	NA	NA	NA	0.00000
XRCC27	152373181	C	A	rs760436323	NO	regulatory	ENST00000359321.1:c.-17G>T	NA	NA	NA	10.36	NA	NA	NA	NA	NA	0.00000
XRCC27	152373181	C	T	rs760436323	NO	regulatory	ENST00000359321.1:c.-17G>A	NA	NA	NA	10.13	NA	NA	NA	NA	NA	0.00000
XRCC27	152373194	C	T	rs371060479	NO	regulatory	ENST00000359321.1:c.-30G>A	NA	NA	NA	10.58	NA	NA	NA	NA	NA	0.00000

ClinVar Significance	SEARCH Control Carrier Count	SEARCH Control Homozygous Carrier Count	SEARCH Control Carrier Frequency	SEARCH Case Carrier Count	SEARCH Case Homozygous Carrier Count	SEARCH Case Carrier Frequency
NA	0	0	0.00000	1	0	0.00008
likely_pathogenic	0	0	0.00000	2	0	0.00015
NA	1	0	0.00018	0	0	0.00000
uncertain_significance	0	0	0.00000	3	0	0.00023
NA	0	0	0.00000	1	0	0.00008
NA	0	0	0.00000	1	0	0.00008
NA	1	0	0.00018	0	0	0.00000
NA	0	0	0.00000	1	0	0.00008
NA	2	0	0.00036	6	0	0.00046
NA	0	0	0.00000	1	0	0.00008
NA	1	0	0.00018	0	0	0.00000
NA	0	0	0.00000	1	0	0.00008
uncertain_significance	1	0	0.00018	1	0	0.00008
NA	1	0	0.00018	1	0	0.00008
uncertain_significance,benign,risk_factor	159	1	0.02897	374	4	0.02858
benign	43	0	0.00784	98	0	0.00749
NA	1	0	0.00018	0	0	0.00000
NA	0	0	0.00000	1	0	0.00008
NA	0	0	0.00000	1	0	0.00008
NA	1	0	0.00018	1	0	0.00008
NA	0	0	0.00000	3	0	0.00023
NA	2	0	0.00036	1	0	0.00008
NA	1	0	0.00018	1	0	0.00008
NA	1	0	0.00018	0	0	0.00000
NA	0	0	0.00000	1	0	0.00008
NA	0	0	0.00000	2	0	0.00015
NA	0	0	0.00000	1	0	0.00008
NA	1	0	0.00018	2	0	0.00015
NA	0	0	0.00000	2	0	0.00015
NA	0	0	0.00000	1	0	0.00008
uncertain_significance	0	0	0.00000	1	0	0.00008
NA	0	0	0.00000	1	0	0.00008
NA	1	0	0.00018	0	0	0.00000
NA	0	0	0.00000	1	0	0.00008
uncertain_significance	2	0	0.00036	1	0	0.00008
uncertain_significance	0	0	0.00000	1	0	0.00008
NA	0	0	0.00000	1	0	0.00008
NA	0	0	0.00000	1	0	0.00008
uncertain_significance	0	0	0.00000	1	0	0.00008
NA	1	0	0.00018	1	0	0.00008
uncertain_significance,likely_benign	1	0	0.00018	1	0	0.00008
likely_benign	2	0	0.00036	3	0	0.00023
pathogenic,benign	13	0	0.00237	39	0	0.00298
uncertain_significance	0	0	0.00000	1	0	0.00008
uncertain_significance	0	0	0.00000	3	0	0.00023
NA	0	0	0.00000	2	0	0.00015
NA	1	0	0.00018	0	0	0.00000
NA	5	0	0.00091	25	0	0.00191
NA	0	0	0.00000	1	0	0.00008
NA	1	0	0.00018	0	0	0.00000
NA	0	0	0.00000	2	0	0.00015
NA	0	0	0.00000	1	0	0.00008
benign,likely_benign	4	0	0.00073	16	0	0.00122
NA	1	0	0.00018	0	0	0.00000
likely_benign	17	0	0.00310	62	0	0.00474
uncertain_significance	1	0	0.00018	3	0	0.00023
uncertain_significance	0	0	0.00000	1	0	0.00008
benign,likely_benign	3	0	0.00055	15	0	0.00115
NA	1	0	0.00018	0	0	0.00000
NA	5	0	0.00091	10	0	0.00076
NA	1	0	0.00018	1	0	0.00008
NA	0	0	0.00000	1	0	0.00008
uncertain_significance	3	0	0.00055	5	0	0.00038
NA	0	0	0.00000	1	0	0.00008
benign	138	2	0.02515	332	3	0.02537
NA	1	0	0.00018	3	0	0.00023
uncertain_significance	1	0	0.00018	4	0	0.00031
NA	0	0	0.00000	1	0	0.00008
NA	0	0	0.00000	1	0	0.00008
uncertain_significance	0	0	0.00000	1	0	0.00008
NA	0	0	0.00000	3	0	0.00023
NA	1	0	0.00018	0	0	0.00000
NA	0	0	0.00000	1	0	0.00008
NA	0	0	0.00000	1	0	0.00008
NA	0	0	0.00000	1	0	0.00008
NA	0	0	0.00000	2	0	0.00015
NA	13	0	0.00237	39	0	0.00298
NA	0	0	0.00000	1	0	0.00008
uncertain_significance	0	0	0.00000	3	0	0.00023
NA	0	0	0.00000	1	0	0.00008
NA	2	0	0.00036	1	0	0.00008
NA	0	0	0.00000	1	0	0.00008
NA	1	0	0.00018	0	0	0.00000
not_provided,likely_benign	11	0	0.00200	46	0	0.00351
uncertain_significance	1	0	0.00018	3	0	0.00023
uncertain_significance	0	0	0.00000	1	0	0.00008
NA	0	0	0.00000	2	0	0.00015
NA	1	0	0.00018	0	0	0.00000
NA	0	0	0.00000	2	0	0.00015
NA	0	0	0.00000	1	0	0.00008
NA	0	0	0.00000	1	0	0.00008
NA	0	0	0.00000	1	0	0.00008
NA	1	0	0.00018	3	0	0.00023
NA	1	0	0.00018	8	0	0.00061
uncertain_significance,risk_factor	21	0	0.00383	85	0	0.00649
NA	0	0	0.00000	1	0	0.00008
NA	0	0	0.00000	1	0	0.00008
NA	0	0	0.00000	1	0	0.00008
likely_benign,pathogenic	0	0	0.00000	1	0	0.00008
uncertain_significance	4	0	0.00073	1	0	0.00008
NA	1	0	0.00018	0	0	0.00000
benign	1	0	0.00018	6	0	0.00046
NA	0	0	0.00000	1	0	0.00008
uncertain_significance,likely_benign	29	0	0.00528	74	1	0.00565
NA	2	0	0.00036	4	0	0.00031
NA	1	0	0.00018	1	0	0.00008
benign	9	0	0.00164	32	0	0.00245
likely_benign	0	0	0.00000	6	0	0.00046
NA	0	0	0.00000	2	0	0.00015
likely_benign	0	0	0.00000	2	0	0.00015
NA	0	0	0.00000	1	0	0.00008
likely_pathogenic,pathogenic	0	0	0.00000	1	0	0.00008
uncertain_significance	0	0	0.00000	1	0	0.00008
pathogenic	0	0	0.00000	3	0	0.00023
NA	0	0	0.00000	1	0	0.00008
NA	0	0	0.00000	1	0	0.00008
NA	1	0	0.00018	0	0	0.00000
uncertain_significance	1	0	0.00018	0	0	0.00000
likely_pathogenic,pathogenic	0	0	0.00000	3	0	0.00023
NA	2	0	0.00036	1	0	0.00008
uncertain_significance	1	0	0.00018	2	0	0.00015
NA	1	0	0.00018	0	0	0.00000
uncertain_significance	1	0	0.00018	4	0	0.00031
uncertain_significance	1	0	0.00018	3	0	0.00023
NA	1	0	0.00018	0	0	0.00000
uncertain_significance	0	0	0.00000	1	0	0.00008
NA	1	0	0.00018	0	0	0.00000
not_provided,benign	2	0	0.00036	10	0	0.00076
NA	0	0	0.00000	8	0	0.00061
uncertain_significance,not_provided	4	0	0.00073	2	0	0.00015
NA	2	0	0.00036	0	0	0.00000
NA	1	0	0.00018	0	0	0.00000
NA	1	0	0.00018	0	0	0.00000
NA	1	0	0.00018	0	0	0.00000
benign	1	0	0.00018	10	0	0.00076
NA	1	0	0.00018	2	0	0.00015
NA	0	0	0.00000	1	0	0.00008
NA	0	0	0.00000	1	0	0.00008
uncertain_significance	0	0	0.00000	3	0	0.00023
uncertain_significance	0	0	0.00000	1	0	0.00008
NA	0	0	0.00000	1	0	0.00008
NA	0	0	0.00000	1	0	0.00008
benign,likely_benign	3	0	0.00055	6	0	0.00046
uncertain_significance	0	0	0.00000	2	0	0.00015
NA	4	0	0.00073	9	0	0.00069
NA	0	0	0.00000	3	0	0.00023
uncertain_significance,not_provided,benign	19	0	0.00346	34	0	0.00260
NA	0	0	0.00000	2	0	0.00015
uncertain_significance	0	0	0.00000	1	0	0.00008
NA	0	0	0.00000	1	0	0.00008
NA	0	0	0.00000	2	0	0.00015
NA	0	0	0.00000	1	0	0.00008
NA	0	0	0.00000	1	0	0.00008
NA	1	0	0.00018	0	0	0.00000
NA	0	0	0.00000	1	0	0.00008
NA	0	0	0.00000	1	0	0.00008
NA	0	0	0.00000	2	0	0.00015
NA	0	0	0.00000	1	0	0.00008
NA	0	0	0.00000	2	0	0.00015
NA	0	0	0.00000	1	0	0.00008
NA	0	0	0.00000	1	0	0.00008
NA	1	0	0.00018	1	0	0.00008
uncertain_significance	1	0	0.00018	0	0	0.00000
likely_benign	12	0	0.00219	24	0	0.00183
NA	1	0	0.00018	1	0	0.00008
NA	1	0	0.00018	0	0	0.00000
NA	0	0	0.00000	1	0	0.00008
NA	0	0	0.00000	1	0	0.00008
uncertain_significance	1	0	0.00018	0	0	0.00000
NA	0	0	0.00000	1	0	0.00008
NA	1	0	0.00018	0	0	0.00000
NA	0	0	0.00000	1	0	0.00008
uncertain_significance	0	0	0.00000	1	0	0.00008
NA	0	0	0.00000	1	0	0.00008
NA	0	0	0.00000	1	0	0.00008
NA	0	0	0.00000	1	0	0.00008
benign	118	3	0.02150	345	6	0.02636
NA	0	0	0.00000	1	0	0.00008
NA	0	0	0.00000	1	0	0.00008
NA	1	0	0.00018	2	0	0.00015
NA	0	0	0.00000	1	0	0.00008
NA	1	0	0.00018	0	0	0.00000
uncertain_significance	0	0	0.00000	1	0	0.00008
NA	0	0	0.00000	1	0	0.00008
NA	1	0	0.00018	1	0	0.00008
benign	1	0	0.00018	10	0	0.00076
likely_benign	1	0	0.00018	0	0	0.00000
NA	0	0	0.00000	1	0	0.00008
NA	1	0	0.00018	1	0	0.00008
NA	0	0	0.00000	1	0	0.00008
pathogenic	0	0	0.00000	3	0	0.00023
NA	0	0	0.00000	3	0	0.00023
NA	0	0	0.00000	1	0	0.00008
NA	0	0	0.00000	1	0	0.00008
NA	0	0	0.00000	1	0	0.00008
uncertain_significance,pathogenic	0	0	0.00000	3	0	0.00023
uncertain_significance	0	0	0.00000	1	0	0.00008
NA	0	0	0.00000	1	0	0.00008
uncertain_significance	1	0	0.00018	0	0	0.00000
NA	0	0	0.00000	2	0	0.00015
NA	0	0	0.00000	1	0	0.00008
uncertain_significance	4	0	0.00073	12	0	0.00092
uncertain_significance	1	0	0.00018	0	0	0.00000
benign,likely_benign	0	0	0.00000	2	0	0.00015
NA	1	0	0.00018	0	0	0.00000
NA	0	0	0.00000	1	0	0.00008
uncertain_significance	0	0	0.00000	1	0	0.00008
NA	1	0	0.00018	2	0	0.00015
NA	0	0	0.00000	1	0	0.00008
NA	0	0	0.00000	1	0	0.00008
uncertain_significance	0	0	0.00000	1	0	0.00008
NA	1	0	0.00018	0	0	0.00000
uncertain_significance,pathogenic	0	0	0.00000	1	0	0.00008
NA	1	0	0.00018	0	0	0.00000
not_provided,benign,likely_benign	0	0	0.00000	4	0	0.00031
NA	1	0	0.00018	1	0	0.00008
NA	0	0	0.00000	1	0	0.00008
uncertain_significance	0	0	0.00000	3	0	0.00023
NA	0	0	0.00000	2	0	0.00015
uncertain_significance	2	0	0.00036	4	0	0.00031
NA	0	0	0.00000	1	0	0.00008
uncertain_significance	1	0	0.00018	0	0	0.00000
uncertain_significance	1	0	0.00018	1	0	0.00008
likely_benign	0	0	0.00000	1	0	0.00008
NA	1	0	0.00018	0	0	0.00000
NA	0	0	0.00000	1	0	0.00008
benign	176	1	0.03207	396	8	0.03026
NA	2	0	0.00036	0	0	0.00000
NA	1	0	0.00018	1	0	0.00008
likely_benign	1	0	0.00018	2	0	0.00015
benign	1	0	0.00018	9	0	0.00069
NA	0	0	0.00000	1	0	0.00008

NA	0	0	0.00000	1	0	0.00008
benign	5	0	0.00091	25	0	0.00191
likely_benign	0	0	0.00000	2	0	0.00015
benign	1	0	0.00018	9	0	0.00069
uncertain_significance	1	0	0.00018	1	0	0.00008
NA	0	0	0.00000	1	0	0.00008
NA	0	0	0.00000	1	0	0.00008
uncertain_significance	3	0	0.00055	1	0	0.00008
likely_benign	10	0	0.00182	22	0	0.00168
likely_benign	0	0	0.00000	1	0	0.00008
uncertain_significance	0	0	0.00000	1	0	0.00008
NA	0	0	0.00000	1	0	0.00008
NA	0	0	0.00000	1	0	0.00008
NA	84	0	0.01531	196	1	0.01498
NA	0	0	0.00000	1	0	0.00008
NA	0	0	0.00000	1	0	0.00008
NA	0	0	0.00000	1	0	0.00008
NA	1	0	0.00018	1	0	0.00008
NA	0	0	0.00000	1	0	0.00008
NA	0	0	0.00000	1	0	0.00008
NA	0	0	0.00000	1	0	0.00008
NA	0	0	0.00000	1	0	0.00008
NA	0	0	0.00000	1	0	0.00008
NA	1	0	0.00018	0	0	0.00000
NA	1	0	0.00018	5	0	0.00038
NA	0	0	0.00000	2	0	0.00015
NA	0	0	0.00000	1	0	0.00008
NA	0	0	0.00000	1	0	0.00008
NA	0	0	0.00000	1	0	0.00008
NA	0	0	0.00000	1	0	0.00008
NA	0	0	0.00000	1	0	0.00008
NA	5	0	0.00091	4	0	0.00031
NA	3	0	0.00055	2	0	0.00015
NA	0	0	0.00000	1	0	0.00008
uncertain_significance	1	0	0.00018	3	0	0.00023
uncertain_significance	9	0	0.00164	25	0	0.00191
likely_benign	0	0	0.00000	1	0	0.00008
NA	0	0	0.00000	1	0	0.00008
NA	0	0	0.00000	1	0	0.00008
uncertain_significance	1	0	0.00018	0	0	0.00000
NA	0	0	0.00000	1	0	0.00008
uncertain_significance	0	0	0.00000	5	0	0.00038
uncertain_significance	1	0	0.00018	1	0	0.00008
NA	0	0	0.00000	1	0	0.00008
NA	1	0	0.00018	0	0	0.00000
NA	0	0	0.00000	1	0	0.00008
NA	1	0	0.00018	1	0	0.00008
NA	0	0	0.00000	2	0	0.00015
NA	4492	1827	0.81851	10701	4378	0.81768
NA	3	0	0.00055	3	0	0.00023
NA	0	0	0.00000	1	0	0.00008
NA	1	0	0.00018	0	0	0.00000
NA	1	0	0.00018	0	0	0.00000
NA	0	0	0.00000	1	0	0.00008
NA	0	0	0.00000	1	0	0.00008
NA	1	0	0.00018	0	0	0.00000
NA	0	0	0.00000	1	0	0.00008
benign,likely_benign,pathogenic	1	0	0.00018	4	0	0.00031
NA	0	0	0.00000	1	0	0.00008
benign,likely_benign	2	0	0.00036	4	0	0.00031
NA	0	0	0.00000	1	0	0.00008
NA	1	0	0.00018	3	0	0.00023
NA	0	0	0.00000	1	0	0.00008
NA	0	0	0.00000	1	0	0.00008
uncertain_significance	1	0	0.00018	0	0	0.00000
NA	0	0	0.00000	4	0	0.00031
NA	1	0	0.00018	0	0	0.00000
benign	317	2	0.05776	749	14	0.05723
uncertain_significance	1	0	0.00018	0	0	0.00000
NA	0	0	0.00000	1	0	0.00008
likely_benign	0	0	0.00000	1	0	0.00008
uncertain_significance	0	0	0.00000	2	0	0.00015
NA	1	0	0.00018	0	0	0.00000
uncertain_significance	1	0	0.00018	2	0	0.00015
NA	2	0	0.00036	2	0	0.00015
NA	0	0	0.00000	3	0	0.00023
NA	0	0	0.00000	1	0	0.00008
benign	1507	119	0.27460	3482	252	0.26607
NA	0	0	0.00000	1	0	0.00008
uncertain_significance	0	0	0.00000	1	0	0.00008
likely_benign	2	0	0.00036	5	0	0.00038
pathogenic	1	0	0.00018	0	0	0.00000
uncertain_significance	0	0	0.00000	1	0	0.00008
likely_benign	1	0	0.00018	5	0	0.00038
uncertain_significance	0	0	0.00000	1	0	0.00008
likely_benign	2	0	0.00036	5	0	0.00038
likely_benign,pathogenic	2	0	0.00036	4	0	0.00031
NA	0	0	0.00000	1	0	0.00008
likely_benign	2	0	0.00036	1	0	0.00008
not_provided,benign	0	0	0.00000	5	0	0.00038
NA	16	0	0.00292	29	0	0.00222
NA	1	0	0.00018	0	0	0.00000
NA	2	0	0.00036	5	0	0.00038
NA	0	0	0.00000	1	0	0.00008
NA	170	3	0.03098	438	12	0.03347
NA	2	0	0.00036	6	0	0.00046
uncertain_significance	0	0	0.00000	1	0	0.00008
NA	1	0	0.00018	0	0	0.00000
uncertain_significance	1	0	0.00018	5	0	0.00038
likely_benign	4	0	0.00073	2	0	0.00015
NA	0	0	0.00000	3	0	0.00023
NA	0	0	0.00000	1	0	0.00008
NA	1	0	0.00018	2	0	0.00015
NA	0	0	0.00000	1	0	0.00008
NA	1	0	0.00018	2	0	0.00015
NA	1	0	0.00018	0	0	0.00000
NA	1	0	0.00018	1	0	0.00008
likely_benign	1	0	0.00018	3	0	0.00023
NA	1	0	0.00018	0	0	0.00000
NA	0	0	0.00000	1	0	0.00008
NA	0	0	0.00000	1	0	0.00008
uncertain_significance,not_provided	0	0	0.00000	1	0	0.00008
NA	0	0	0.00000	1	0	0.00008
uncertain_significance	0	0	0.00000	3	0	0.00023
NA	1	0	0.00018	0	0	0.00000
NA	0	0	0.00000	1	0	0.00008
uncertain_significance	0	0	0.00000	1	0	0.00008
NA	1	0	0.00018	3	0	0.00023
uncertain_significance	0	0	0.00000	2	0	0.00015
NA	0	0	0.00000	1	0	0.00008
NA	1	0	0.00018	0	0	0.00000
uncertain_significance	27	0	0.00492	40	0	0.00306
uncertain_significance	0	0	0.00000	1	0	0.00008
NA	0	0	0.00000	1	0	0.00008
NA	0	0	0.00000	1	0	0.00008
NA	0	0	0.00000	1	0	0.00008
NA	98	0	0.01786	256	2	0.01956
NA	0	0	0.00000	4	0	0.00031
NA	1	0	0.00018	0	0	0.00000
NA	0	0	0.00000	1	0	0.00008
NA	2	0	0.00036	5	0	0.00038
NA	0	0	0.00000	1	0	0.00008
NA	1	0	0.00018	1	0	0.00008
NA	0	0	0.00000	1	0	0.00008
NA	0	0	0.00000	1	0	0.00008
NA	0	0	0.00000	1	0	0.00008
NA	0	0	0.00000	1	0	0.00008
NA	1	0	0.00018	0	0	0.00000
benign	0	0	0.00000	1	0	0.00008
uncertain_significance	3	0	0.00055	12	0	0.00092
uncertain_significance	5	0	0.00091	13	0	0.00099
NA	0	0	0.00000	1	0	0.00008
NA	0	0	0.00000	2	0	0.00015
NA	2	0	0.00036	1	0	0.00008
NA	2	0	0.00036	3	0	0.00023
NA	0	0	0.00000	1	0	0.00008
not_provided,benign	5	0	0.00091	25	0	0.00191
uncertain_significance	3	0	0.00055	0	0	0.00000
likely_benign	0	0	0.00000	1	0	0.00008
benign	7	0	0.00128	8	0	0.00061
NA	1	0	0.00018	0	0	0.00000
NA	0	0	0.00000	1	0	0.00008
NA	0	0	0.00000	1	0	0.00008
benign,likely_benign	205	6	0.03735	487	2	0.03721
uncertain_significance	1	0	0.00018	3	0	0.00023
uncertain_significance	0	0	0.00000	1	0	0.00008
uncertain_significance	14	0	0.00255	18	0	0.00138
likely_benign	0	0	0.00000	1	0	0.00008
likely_benign	0	0	0.00000	1	0	0.00008
uncertain_significance	2	0	0.00036	1	0	0.00008
uncertain_significance	1	0	0.00018	15	0	0.00115
uncertain_significance	0	0	0.00000	1	0	0.00008
NA	1	0	0.00018	0	0	0.00000
uncertain_significance	2	0	0.00036	3	0	0.00023
uncertain_significance,not_provided,benign	4	0	0.00073	14	0	0.00107
uncertain_significance	0	0	0.00000	1	0	0.00008
NA	0	0	0.00000	2	0	0.00015
uncertain_significance	0	0	0.00000	2	0	0.00015
likely_benign	0	0	0.00000	2	0	0.00015
uncertain_significance	22	0	0.00401	36	0	0.00275
NA	0	0	0.00000	1	0	0.00008
NA	1	0	0.00018	0	0	0.00000
NA	0	0	0.00000	1	0	0.00008
NA	1	0	0.00018	1	0	0.00008
NA	0	0	0.00000	3	0	0.00023
uncertain_significance	0	0	0.00000	2	0	0.00015
benign	30	0	0.00547	76	0	0.00581
NA	1	0	0.00018	0	0	0.00000
likely_benign	2	0	0.00036	0	0	0.00000
NA	0	0	0.00000	1	0	0.00008
NA	0	0	0.00000	1	0	0.00008
NA	0	0	0.00000	1	0	0.00008
NA	1	0	0.00018	1	0	0.00008
benign,likely_benign	535	18	0.09749	1280	33	0.09781
NA	0	0	0.00000	1	0	0.00008
NA	1	0	0.00018	0	0	0.00000
NA	1	0	0.00018	1	0	0.00008
NA	0	0	0.00000	1	0	0.00008
uncertain_significance	4	0	0.00073	2	0	0.00015
likely_benign	1	0	0.00018	2	0	0.00015
NA	0	0	0.00000	1	0	0.00008
NA	0	0	0.00000	1	0	0.00008
NA	1	0	0.00018	0	0	0.00000
NA	0	0	0.00000	4	0	0.00031
NA	1	0	0.00018	0	0	0.00000
NA	0	0	0.00000	1	0	0.00008
benign	13	0	0.00237	39	0	0.00298
not_provided,likely_benign	0	0	0.00000	1	0	0.00008
NA	0	0	0.00000	1	0	0.00008
likely_benign	0	0	0.00000	1	0	0.00008
NA	0	0	0.00000	1	0	0.00008
NA	0	0	0.00000	1	0	0.00008
NA	0	0	0.00000	3	0	0.00023
NA	0	0	0.00000	1	0	0.00008
uncertain_significance	0	0	0.00000	1	0	0.00008
uncertain_significance,not_provided	0	0	0.00000	1	0	0.00008
likely_benign	10	0	0.00182	15	0	0.00115
NA	0	0	0.00000	1	0	0.00008
NA	1	0	0.00018	0	0	0.00000
benign,likely_benign	1	0	0.00018	5	0	0.00038
NA	3	0	0.00055	1	0	0.00008
NA	0	0	0.00000	2	0	0.00015
NA	0	0	0.00000	5	0	0.00038
NA	0	0	0.00000	1	0	0.00008
NA	13	0	0.00237	38	0	0.00290
NA	0	0	0.00000	1	0	0.00008
NA	0	0	0.00000	1	0	0.00008
NA	0	0	0.00000	1	0	0.00008
NA	1	0	0.00018	3	0	0.00023
NA	1	0	0.00018	0	0	0.00000
uncertain_significance	0	0	0.00000	2	0	0.00015
NA	0	0	0.00000	1	0	0.00008
uncertain_significance,not_provided	29	0	0.00528	53	0	0.00405
uncertain_significance	0	0	0.00000	7	0	0.00053
NA	0	0	0.00000	4	0	0.00031
NA	12	0	0.00219	36	0	0.00275
NA	0	0	0.00000	2	0	0.00015
NA	7	0	0.00128	31	0	0.00237
NA	1	0	0.00018	1	0	0.00008
NA	0	0	0.00000	3	0	0.00023
NA	0	0	0.00000	2	0	0.00015
NA	0	0	0.00000	1	0	0.00008
NA	0	0	0.00000	1	0	0.00008
uncertain_significance,likely_pathogenic	0	0	0.00000	4	0	0.00031
NA	1	0	0.00018	0	0	0.00000
uncertain_significance	0	0	0.00000	1	0	0.00008
NA	0	0	0.00000	1	0	0.00008
NA	0	0	0.00000	3	0	0.00023

NA	2	0	0.00036	3	0	0.00023
likely_pathogenic,pathogenic	1	0	0.00018	1	0	0.00008
NA	1	0	0.00018	0	0	0.00000
NA	2	0	0.00036	0	0	0.00000
NA	1	0	0.00018	0	0	0.00000
NA	1	0	0.00018	0	0	0.00000
NA	1	0	0.00018	0	0	0.00000
NA	0	0	0.00000	2	0	0.00015
likely_benign	1	0	0.00018	0	0	0.00000
NA	1	0	0.00018	0	0	0.00000
NA	0	0	0.00000	1	0	0.00008
NA	0	0	0.00000	1	0	0.00008
NA	0	0	0.00000	4	0	0.00031
NA	0	0	0.00000	1	0	0.00008
NA	1	0	0.00018	0	0	0.00000
benign	55	1	0.01002	134	0	0.01024
benign	341	6	0.06214	843	6	0.06442
NA	0	0	0.00000	2	0	0.00015
NA	0	0	0.00000	1	0	0.00008
NA	1	0	0.00018	1	0	0.00008
NA	0	0	0.00000	1	0	0.00008
likely_benign	0	0	0.00000	1	0	0.00008
NA	1	0	0.00018	0	0	0.00000
NA	0	0	0.00000	1	0	0.00008
benign	1512	120	0.27551	3484	253	0.26622
not_provided,benign,likely_benign	43	0	0.00784	133	0	0.01016
NA	0	0	0.00000	1	0	0.00008
NA	1	0	0.00018	4	0	0.00031
NA	0	0	0.00000	1	0	0.00008
NA	1	0	0.00018	0	0	0.00000
NA	0	0	0.00000	1	0	0.00008
uncertain_significance	0	0	0.00000	2	0	0.00015
NA	0	0	0.00000	1	0	0.00008
NA	0	0	0.00000	1	0	0.00008
NA	0	0	0.00000	1	0	0.00008
NA	0	0	0.00000	1	0	0.00008
NA	0	0	0.00000	1	0	0.00008
NA	0	0	0.00000	1	0	0.00008
uncertain_significance	0	0	0.00000	1	0	0.00008
NA	0	0	0.00000	3	0	0.00023
NA	1	0	0.00018	0	0	0.00000
likely_benign	84	0	0.01531	196	1	0.01498
uncertain_significance	5	0	0.00091	4	0	0.00031
uncertain_significance	1	0	0.00018	1	0	0.00008
NA	1	0	0.00018	0	0	0.00000
uncertain_significance,pathogenic	0	0	0.00000	2	0	0.00015
NA	0	0	0.00000	1	0	0.00008
NA	0	0	0.00000	1	0	0.00008
NA	0	0	0.00000	1	0	0.00008
NA	0	0	0.00000	2	0	0.00015
NA	0	0	0.00000	1	0	0.00008
NA	0	0	0.00000	1	0	0.00008
pathogenic	0	0	0.00000	2	0	0.00015
NA	0	0	0.00000	1	0	0.00008
NA	0	0	0.00000	1	0	0.00008
NA	0	0	0.00000	1	0	0.00008
uncertain_significance	0	0	0.00000	4	0	0.00031
NA	0	0	0.00000	1	0	0.00008
NA	0	0	0.00000	1	0	0.00008
NA	1	0	0.00018	0	0	0.00000
NA	1	0	0.00018	3	0	0.00023
NA	1	0	0.00018	2	0	0.00015
uncertain_significance	1	0	0.00018	1	0	0.00008
NA	0	0	0.00000	1	0	0.00008
not_provided,likely_benign	22	0	0.00401	45	0	0.00344
benign	0	0	0.00000	3	0	0.00023
NA	0	0	0.00000	3	0	0.00023
benign	1	0	0.00018	9	0	0.00069
NA	1	0	0.00018	0	0	0.00000
NA	2	0	0.00036	2	0	0.00015
NA	0	0	0.00000	2	0	0.00015
NA	0	0	0.00000	1	0	0.00008
NA	0	0	0.00000	2	0	0.00015
NA	0	0	0.00000	1	0	0.00008
pathogenic	3	0	0.00055	1	0	0.00008
uncertain_significance	0	0	0.00000	1	0	0.00008
NA	0	0	0.00000	1	0	0.00008
likely_benign	0	0	0.00000	1	0	0.00008
NA	0	0	0.00000	3	0	0.00023
NA	1	0	0.00018	0	0	0.00000
NA	1	0	0.00018	0	0	0.00000
uncertain_significance,not_provided	1	0	0.00018	0	0	0.00000
NA	0	0	0.00000	1	0	0.00008
NA	1	0	0.00018	0	0	0.00000
NA	1	0	0.00018	0	0	0.00000
NA	2	0	0.00036	0	0	0.00000
NA	0	0	0.00000	1	0	0.00008
likely_benign	0	0	0.00000	2	0	0.00015
not_provided,benign	5	0	0.00091	17	1	0.00130
NA	0	0	0.00000	1	0	0.00008
uncertain_significance	1	0	0.00018	0	0	0.00000
NA	1	0	0.00018	1	0	0.00008
likely_benign	0	0	0.00000	1	0	0.00008
NA	0	0	0.00000	1	0	0.00008
NA	0	0	0.00000	2	0	0.00015
NA	62	0	0.01130	140	1	0.01070
NA	0	0	0.00000	1	0	0.00008
NA	0	0	0.00000	1	0	0.00008
NA	1	0	0.00018	2	0	0.00015
benign	1	0	0.00018	0	0	0.00000
NA	0	0	0.00000	3	0	0.00023
NA	0	0	0.00000	1	0	0.00008
pathogenic	0	0	0.00000	1	0	0.00008
NA	0	0	0.00000	1	0	0.00008
not_provided,benign	0	0	0.00000	4	0	0.00031
NA	0	0	0.00000	1	0	0.00008
NA	1	0	0.00018	0	0	0.00000
NA	1	0	0.00018	1	0	0.00008
NA	0	0	0.00000	1	0	0.00008
NA	1	0	0.00018	1	0	0.00008
likely_benign	0	0	0.00000	2	0	0.00015
uncertain_significance	0	0	0.00000	2	0	0.00015
NA	0	0	0.00000	1	0	0.00008
NA	0	0	0.00000	3	0	0.00023
NA	1	0	0.00018	0	0	0.00000
NA	1	0	0.00018	0	0	0.00000
uncertain_significance	0	0	0.00000	2	0	0.00015
NA	0	0	0.00000	1	0	0.00008
NA	1	0	0.00018	2	0	0.00015
benign	1	0	0.00018	8	0	0.00061
uncertain_significance	2	0	0.00036	3	0	0.00023
NA	0	0	0.00000	1	0	0.00008
NA	0	0	0.00000	1	0	0.00008
likely_pathogenic	0	0	0.00000	1	0	0.00008
likely_benign	1	0	0.00018	4	0	0.00031
NA	1	0	0.00018	0	0	0.00000
uncertain_significance	0	0	0.00000	2	0	0.00015
likely_benign	8	0	0.00146	10	0	0.00076
NA	0	0	0.00000	1	0	0.00008
uncertain_significance	0	0	0.00000	1	0	0.00008
uncertain_significance	4	0	0.00073	6	0	0.00046
NA	1	0	0.00018	0	0	0.00000
uncertain_significance,likely_benign	2	0	0.00036	2	0	0.00015
likely_benign	0	0	0.00000	1	0	0.00008
NA	0	0	0.00000	1	0	0.00008
not_provided,benign	1	0	0.00018	15	0	0.00115
NA	0	0	0.00000	1	0	0.00008
NA	0	0	0.00000	1	0	0.00008
uncertain_significance	2	0	0.00036	2	0	0.00015
uncertain_significance	1	0	0.00018	0	0	0.00000
NA	0	0	0.00000	1	0	0.00008
NA	0	0	0.00000	1	0	0.00008
NA	0	0	0.00000	2	0	0.00015
not_provided,benign	1	0	0.00018	4	0	0.00031
NA	0	0	0.00000	1	0	0.00008
NA	0	0	0.00000	2	0	0.00015
likely_benign	3	0	0.00055	6	0	0.00046
benign	0	0	0.00000	1	0	0.00008
NA	0	0	0.00000	1	0	0.00008
NA	1	0	0.00018	1	0	0.00008
uncertain_significance	0	0	0.00000	2	0	0.00015
NA	0	0	0.00000	1	0	0.00008
likely_benign	0	0	0.00000	1	0	0.00008
NA	0	0	0.00000	2	0	0.00015
uncertain_significance	3	0	0.00055	5	0	0.00038
NA	0	0	0.00000	1	0	0.00008
NA	0	0	0.00000	1	0	0.00008
pathogenic,risk_factor	0	0	0.00000	8	0	0.00061
NA	1	0	0.00018	0	0	0.00000
NA	0	0	0.00000	1	0	0.00008
likely_benign	0	0	0.00000	1	0	0.00008
likely_benign	1	0	0.00018	1	0	0.00008
NA	0	0	0.00000	1	0	0.00008
uncertain_significance	0	0	0.00000	1	0	0.00008
NA	1	0	0.00018	1	0	0.00008
NA	1	0	0.00018	0	0	0.00000
NA	0	0	0.00000	1	0	0.00008
NA	0	0	0.00000	1	0	0.00008
uncertain_significance	18	0	0.00328	16	0	0.00122
NA	0	0	0.00000	1	0	0.00008
NA	0	0	0.00000	1	0	0.00008
uncertain_significance	1	0	0.00018	4	0	0.00031
likely_benign	0	0	0.00000	2	0	0.00015
uncertain_significance	0	0	0.00000	2	0	0.00015
likely_benign	0	0	0.00000	1	0	0.00008
NA	0	0	0.00000	1	0	0.00008
uncertain_significance	1	0	0.00018	6	0	0.00046
NA	0	0	0.00000	1	0	0.00008
NA	0	0	0.00000	1	0	0.00008
NA	1	0	0.00018	0	0	0.00000
benign	0	0	0.00000	1	0	0.00008
NA	0	0	0.00000	1	0	0.00008
NA	0	0	0.00000	1	0	0.00008
uncertain_significance	1	0	0.00018	0	0	0.00000
pathogenic	0	0	0.00000	1	0	0.00008
NA	0	0	0.00000	1	0	0.00008
uncertain_significance	1	0	0.00018	1	0	0.00008
NA	0	0	0.00000	1	0	0.00008
uncertain_significance	0	0	0.00000	1	0	0.00008
NA	0	0	0.00000	1	0	0.00008
benign,likely_benign	36	0	0.00656	77	0	0.00588
NA	0	0	0.00000	2	0	0.00015
NA	0	0	0.00000	1	0	0.00008
NA	0	0	0.00000	1	0	0.00008
NA	0	0	0.00000	5	0	0.00038
NA	0	0	0.00000	1	0	0.00008
NA	0	0	0.00000	1	0	0.00008
NA	1	0	0.00018	0	0	0.00000
uncertain_significance,not_provided	9	0	0.00164	8	0	0.00061
benign	2	0	0.00036	1	0	0.00008
NA	0	0	0.00000	1	0	0.00008
NA	0	0	0.00000	1	0	0.00008
NA	1	0	0.00018	6	0	0.00046
NA	0	0	0.00000	1	0	0.00008
NA	0	0	0.00000	1	0	0.00008
NA	2	0	0.00036	3	0	0.00023
NA	0	0	0.00000	1	0	0.00008
NA	1	0	0.00018	0	0	0.00000
NA	0	0	0.00000	1	0	0.00008
uncertain_significance	0	0	0.00000	1	0	0.00008
NA	0	0	0.00000	1	0	0.00008
NA	0	0	0.00000	1	0	0.00008
likely_benign	1	0	0.00018	3	0	0.00023
NA	1	0	0.00018	0	0	0.00000
NA	1	0	0.00018	0	0	0.00000
NA	1	0	0.00018	0	0	0.00000
NA	0	0	0.00000	1	0	0.00008
NA	2	0	0.00036	1	0	0.00008
NA	0	0	0.00000	1	0	0.00008
NA	0	0	0.00000	1	0	0.00008
NA	0	0	0.00000	1	0	0.00008
NA	0	0	0.00000	1	0	0.00008
uncertain_significance	0	0	0.00000	1	0	0.00008
uncertain_significance	0	0	0.00000	1	0	0.00008
NA	1	0	0.00018	0	0	0.00000
NA	1	0	0.00018	1	0	0.00008
NA	0	0	0.00000	1	0	0.00008
uncertain_significance	0	0	0.00000	1	0	0.00008
NA	1	0	0.00018	0	0	0.00000
NA	0	0	0.00000	1	0	0.00008
NA	0	0	0.00000	1	0	0.00008
NA	1	0	0.00018	0	0	0.00000
NA	0	0	0.00000	1	0	0.00008
uncertain_significance	0	0	0.00000	4	0	0.00031
uncertain_significance	1	0	0.00018	7	0	0.00053
NA	0	0	0.00000	1	0	0.00008
NA	0	0	0.00000	1	0	0.00008
NA	1	0	0.00018	0	0	0.00000

pathogenic	0	0	0.00000	1	0	0.00008
pathogenic	0	0	0.00000	2	0	0.00015
NA	0	0	0.00000	1	0	0.00008
benign	1	0	0.00018	1	0	0.00008
NA	0	0	0.00000	1	0	0.00008
NA	0	0	0.00000	1	0	0.00008
NA	0	0	0.00000	1	0	0.00008
likely_pathogenic	1	0	0.00018	3	0	0.00023
NA	0	0	0.00000	1	0	0.00008
NA	1	0	0.00018	1	0	0.00008
NA	1	0	0.00018	1	0	0.00008
NA	0	0	0.00000	1	0	0.00008
likely_pathogenic,pathogenic	0	0	0.00000	6	0	0.00046
uncertain_significance,not_provided	0	0	0.00000	2	0	0.00015
uncertain_significance	0	0	0.00000	1	0	0.00008
uncertain_significance	0	0	0.00000	1	0	0.00008
NA	0	0	0.00000	1	0	0.00008
uncertain_significance	3	0	0.00055	5	0	0.00038
NA	1	0	0.00018	0	0	0.00000
NA	1	0	0.00018	2	0	0.00015
NA	0	0	0.00000	1	0	0.00008
NA	0	0	0.00000	1	0	0.00008
NA	0	0	0.00000	1	0	0.00008
likely_benign	13	0	0.00237	40	0	0.00306
uncertain_significance	0	0	0.00000	1	0	0.00008
NA	0	0	0.00000	1	0	0.00008
benign	9	0	0.00164	11	0	0.00084
NA	0	0	0.00000	1	0	0.00008
NA	1	0	0.00018	7	0	0.00053
NA	1	0	0.00018	1	0	0.00008
NA	0	0	0.00000	1	0	0.00008
uncertain_significance,not_provided	1	0	0.00018	7	0	0.00053
NA	0	0	0.00000	1	0	0.00008
NA	1	0	0.00018	0	0	0.00000
NA	0	0	0.00000	3	0	0.00023
benign	478	14	0.08710	1149	29	0.08780
NA	0	0	0.00000	1	0	0.00008
NA	0	0	0.00000	1	0	0.00008
NA	0	0	0.00000	1	0	0.00008
NA	0	0	0.00000	1	0	0.00008
NA	115	0	0.02095	234	2	0.01788
NA	3	0	0.00055	2	0	0.00015
NA	4441	1777	0.80922	10594	4225	0.80951
NA	0	0	0.00000	1	0	0.00008
NA	0	0	0.00000	1	0	0.00008
NA	0	0	0.00000	1	0	0.00008
NA	1	0	0.00018	1	0	0.00008
NA	4	0	0.00073	0	0	0.00000
uncertain_significance	0	0	0.00000	1	0	0.00008
NA	0	0	0.00000	1	0	0.00008
NA	2	0	0.00036	0	0	0.00000
NA	2	0	0.00036	0	0	0.00000
NA	0	0	0.00000	1	0	0.00008
NA	0	0	0.00000	2	0	0.00015
NA	4494	1830	0.81888	10705	4376	0.81799
NA	0	0	0.00000	1	0	0.00008
NA	0	0	0.00000	1	0	0.00008
NA	0	0	0.00000	1	0	0.00008
NA	0	0	0.00000	2	0	0.00015
NA	1	0	0.00018	1	0	0.00008
NA	0	0	0.00000	4	0	0.00031
NA	1	0	0.00018	2	0	0.00015
NA	1	0	0.00018	2	0	0.00015
NA	0	0	0.00000	1	0	0.00008
likely_pathogenic,pathogenic	1	0	0.00018	4	0	0.00031
NA	0	0	0.00000	1	0	0.00008
uncertain_significance	0	0	0.00000	1	0	0.00008
NA	2	0	0.00036	1	0	0.00008
NA	1	0	0.00018	0	0	0.00000
NA	1	0	0.00018	0	0	0.00000
likely_pathogenic,pathogenic	1	0	0.00018	0	0	0.00000
NA	0	0	0.00000	1	0	0.00008
NA	0	0	0.00000	1	0	0.00008
uncertain_significance	7	0	0.00128	7	0	0.00053
uncertain_significance	1	0	0.00018	0	0	0.00000
NA	0	0	0.00000	1	0	0.00008
pathogenic	0	0	0.00000	1	0	0.00008
benign	5	0	0.00091	34	0	0.00260
not_provided,benign	1	0	0.00018	9	0	0.00069
uncertain_significance	1	0	0.00018	1	0	0.00008
NA	1	0	0.00018	0	0	0.00000
NA	0	0	0.00000	1	0	0.00008
NA	0	0	0.00000	1	0	0.00008
uncertain_significance	0	0	0.00000	2	0	0.00015
uncertain_significance	2	0	0.00036	3	0	0.00023
NA	0	0	0.00000	1	0	0.00008
benign	0	0	0.00000	7	0	0.00053
uncertain_significance	2	0	0.00036	3	0	0.00023
NA	0	0	0.00000	2	0	0.00015
NA	4752	2201	0.86589	11201	5147	0.85589
NA	0	0	0.00000	1	0	0.00008
NA	2	0	0.00036	4	0	0.00031
NA	0	0	0.00000	1	0	0.00008
NA	0	0	0.00000	3	0	0.00023
NA	0	0	0.00000	1	0	0.00008
not_provided,benign	1	0	0.00018	4	0	0.00031
uncertain_significance	0	0	0.00000	1	0	0.00008
NA	1	0	0.00018	1	0	0.00008
NA	6	0	0.00109	9	0	0.00069
pathogenic,risk_factor	3	0	0.00055	2	0	0.00015
uncertain_significance	0	0	0.00000	2	0	0.00015
uncertain_significance	0	0	0.00000	1	0	0.00008
pathogenic	3	0	0.00055	15	0	0.00115
NA	1	0	0.00018	0	0	0.00000
NA	1	0	0.00018	1	0	0.00008
NA	0	0	0.00000	1	0	0.00008
NA	1	0	0.00018	0	0	0.00000
NA	0	0	0.00000	1	0	0.00008
uncertain_significance	1	0	0.00018	0	0	0.00000
uncertain_significance	0	0	0.00000	1	0	0.00008
NA	1	0	0.00018	1	0	0.00008
NA	0	0	0.00000	1	0	0.00008
uncertain_significance	1	0	0.00018	5	0	0.00038
uncertain_significance	1	0	0.00018	5	0	0.00038
NA	0	0	0.00000	2	0	0.00015
NA	1	0	0.00018	1	0	0.00008
NA	0	0	0.00000	3	0	0.00023
pathogenic	23	0	0.00419	173	0	0.01322
uncertain_significance	0	0	0.00000	1	0	0.00008
NA	0	0	0.00000	2	0	0.00015
NA	0	0	0.00000	1	0	0.00008
NA	0	0	0.00000	1	0	0.00008
NA	1	0	0.00018	0	0	0.00000
NA	0	0	0.00000	1	0	0.00008
uncertain_significance	0	0	0.00000	2	0	0.00015
uncertain_significance	0	0	0.00000	3	0	0.00023
uncertain_significance	0	0	0.00000	5	0	0.00038
NA	0	0	0.00000	3	0	0.00023
NA	1	0	0.00018	0	0	0.00000
NA	0	0	0.00000	2	0	0.00015
pathogenic	0	0	0.00000	1	0	0.00008
NA	0	0	0.00000	1	0	0.00008
NA	1	0	0.00018	1	0	0.00008
NA	1	0	0.00018	0	0	0.00000
NA	1	0	0.00018	0	0	0.00000
NA	0	0	0.00000	1	0	0.00008
NA	0	0	0.00000	2	0	0.00015
uncertain_significance	0	0	0.00000	1	0	0.00008
uncertain_significance	1	0	0.00018	1	0	0.00008
uncertain_significance	0	0	0.00000	1	0	0.00008
NA	2	0	0.00036	3	0	0.00023
NA	1	0	0.00018	0	0	0.00000
NA	1	0	0.00018	0	0	0.00000
NA	0	0	0.00000	1	0	0.00008
NA	1	0	0.00018	2	0	0.00015
NA	1	0	0.00018	5	0	0.00038
NA	0	0	0.00000	1	0	0.00008
likely_pathogenic	0	0	0.00000	1	0	0.00008
likely_benign	0	0	0.00000	1	0	0.00008
NA	0	0	0.00000	1	0	0.00008
NA	1	0	0.00018	2	0	0.00015
NA	2	0	0.00036	3	0	0.00023
NA	1	0	0.00018	1	0	0.00008
NA	2	0	0.00036	3	0	0.00023
NA	1	0	0.00018	2	0	0.00015
NA	0	0	0.00000	1	0	0.00008
NA	0	0	0.00000	1	0	0.00008
NA	0	0	0.00000	1	0	0.00008
NA	0	0	0.00000	1	0	0.00008
NA	0	0	0.00000	1	0	0.00008
NA	0	0	0.00000	1	0	0.00008
NA	1	0	0.00018	1	0	0.00008
NA	1	0	0.00018	0	0	0.00000
NA	0	0	0.00000	1	0	0.00008
NA	0	0	0.00000	1	0	0.00008
NA	0	0	0.00000	1	0	0.00008
NA	2	0	0.00036	2	0	0.00015
uncertain_significance	0	0	0.00000	1	0	0.00008
NA	0	0	0.00000	2	0	0.00015
uncertain_significance,not_provided,pathogenic	1	1	0.00018	5	3	0.00038
uncertain_significance	1	0	0.00018	0	0	0.00000
not_provided	0	0	0.00000	1	0	0.00008
NA	0	0	0.00000	1	0	0.00008
NA	2	0	0.00036	1	0	0.00008
NA	1	0	0.00018	0	0	0.00000
uncertain_significance	0	0	0.00000	1	0	0.00008
likely_benign	0	0	0.00000	3	0	0.00023
NA	0	0	0.00000	1	0	0.00008
NA	0	0	0.00000	1	0	0.00008
NA	1	0	0.00018	1	0	0.00008
NA	1	0	0.00018	2	0	0.00015
NA	0	0	0.00000	1	0	0.00008
NA	1	0	0.00018	0	0	0.00000
pathogenic	0	0	0.00000	4	0	0.00031
NA	1	0	0.00018	0	0	0.00000
NA	0	0	0.00000	1	0	0.00008
NA	0	0	0.00000	1	0	0.00008
uncertain_significance	1	0	0.00018	1	0	0.00008
uncertain_significance	2	0	0.00036	11	0	0.00084
uncertain_significance,pathogenic	0	0	0.00000	1	0	0.00008
uncertain_significance,pathogenic	2	0	0.00036	6	0	0.00046
uncertain_significance	8	0	0.00146	24	0	0.00183
NA	0	0	0.00000	1	0	0.00008
NA	1	0	0.00018	1	0	0.00008
uncertain_significance	1	0	0.00018	0	0	0.00000
uncertain_significance	0	0	0.00000	2	0	0.00015
NA	0	0	0.00000	1	0	0.00008
likely_benign	3	0	0.00055	3	0	0.00023
uncertain_significance	2	0	0.00036	0	0	0.00000
not_provided,likely_pathogenic,pathogenic,risk_factor	4	0	0.00073	20	0	0.00153
NA	0	0	0.00000	1	0	0.00008
NA	0	0	0.00000	1	0	0.00008
NA	0	0	0.00000	1	0	0.00008
not_provided	33	0	0.00601	54	0	0.00413
NA	1	0	0.00018	0	0	0.00000
not_provided,pathogenic	1	0	0.00018	2	0	0.00015
not_provided,pathogenic	0	0	0.00000	1	0	0.00008
uncertain_significance	1	0	0.00018	1	0	0.00008
likely_benign	1	0	0.00018	7	0	0.00053
pathogenic	0	0	0.00000	2	0	0.00015
uncertain_significance	0	0	0.00000	1	0	0.00008
NA	0	0	0.00000	1	0	0.00008
uncertain_significance	0	0	0.00000	2	0	0.00015
likely_pathogenic	3	0	0.00055	10	0	0.00076
uncertain_significance	1	0	0.00018	4	0	0.00031
uncertain_significance,not_provided	1	0	0.00018	33	0	0.00252
NA	0	0	0.00000	2	0	0.00015
NA	0	0	0.00000	1	0	0.00008
NA	0	0	0.00000	1	0	0.00008
NA	1	0	0.00018	0	0	0.00000
NA	1635	159	0.29792	3943	363	0.30129
NA	0	0	0.00000	1	0	0.00008
NA	0	0	0.00000	1	0	0.00008
pathogenic	0	0	0.00000	5	0	0.00038
pathogenic	0	0	0.00000	1	0	0.00008
NA	1	0	0.00018	0	0	0.00000
not_provided,pathogenic	0	0	0.00000	3	0	0.00023
benign	260	3	0.04738	655	13	0.05005
NA	0	0	0.00000	1	0	0.00008
NA	3	0	0.00055	12	0	0.00092
NA	1	0	0.00018	1	0	0.00008
uncertain_significance	2	0	0.00036	1	0	0.00008
NA	0	0	0.00000	1	0	0.00008
NA	0	0	0.00000	1	0	0.00008
NA	0	0	0.00000	1	0	0.00008
NA	0	0	0.00000	1	0	0.00008
likely_benign	1	0	0.00018	0	0	0.00000
not_provided	1	0	0.00018	0	0	0.00000

uncertain_significance,not_provided	4	0	0.00073	8	0	0.00061
uncertain_significance	2	0	0.00036	2	0	0.00015
uncertain_significance	0	0	0.00000	4	0	0.00031
uncertain_significance	0	0	0.00000	1	0	0.00008
NA	0	0	0.00000	1	0	0.00008
NA	1	0	0.00018	0	0	0.00000
not_provided,pathogenic,risk_factor	0	0	0.00000	3	0	0.00023
NA	0	0	0.00000	1	0	0.00008
likely_benign	13	0	0.00237	57	0	0.00436
likely_benign	1	0	0.00018	1	0	0.00008
NA	0	0	0.00000	2	0	0.00015
uncertain_significance	1	0	0.00018	6	0	0.00046
NA	1	0	0.00018	0	0	0.00000
NA	0	0	0.00000	1	0	0.00008
NA	0	0	0.00000	1	0	0.00008
NA	1	0	0.00018	0	0	0.00000
NA	0	0	0.00000	1	0	0.00008
likely_benign	0	0	0.00000	2	0	0.00015
NA	0	0	0.00000	1	0	0.00008
NA	0	0	0.00000	1	0	0.00008
NA	0	0	0.00000	1	0	0.00008
uncertain_significance	1	0	0.00018	2	0	0.00015
uncertain_significance	0	0	0.00000	1	0	0.00008
benign	347	1	0.06323	803	11	0.06136
likely_benign	0	0	0.00000	1	0	0.00008
uncertain_significance	0	0	0.00000	1	0	0.00008
likely_benign	0	0	0.00000	2	0	0.00015
pathogenic	0	0	0.00000	4	0	0.00031
likely_benign	0	0	0.00000	2	0	0.00015
uncertain_significance	0	0	0.00000	1	0	0.00008
uncertain_significance	0	0	0.00000	1	0	0.00008
uncertain_significance	0	0	0.00000	2	0	0.00015
NA	0	0	0.00000	1	0	0.00008
NA	0	0	0.00000	1	0	0.00008
NA	0	0	0.00000	1	0	0.00008
uncertain_significance	0	0	0.00000	2	0	0.00015
benign	0	0	0.00000	2	0	0.00015
benign	0	0	0.00000	2	0	0.00015
NA	0	0	0.00000	2	0	0.00015
NA	0	0	0.00000	1	0	0.00008
pathogenic,risk_factor	1	0	0.00018	8	0	0.00061
NA	0	0	0.00000	1	0	0.00008
pathogenic,risk_factor	2	0	0.00036	19	0	0.00145
uncertain_significance	1	0	0.00018	1	0	0.00008
NA	1	0	0.00018	1	0	0.00008
NA	2	0	0.00036	0	0	0.00000
uncertain_significance	1	0	0.00018	0	0	0.00000
NA	0	0	0.00000	2	0	0.00015
NA	1	0	0.00018	0	0	0.00000
benign,likely_benign	7	0	0.00128	15	0	0.00115
not_provided,benign	270	0	0.04920	591	4	0.04516
NA	0	0	0.00000	1	0	0.00008
NA	0	0	0.00000	1	0	0.00008
pathogenic,risk_factor	0	0	0.00000	1	0	0.00008
uncertain_significance	1	0	0.00018	2	0	0.00015
NA	1	0	0.00018	0	0	0.00000
pathogenic	0	0	0.00000	1	0	0.00008
likely_benign	0	0	0.00000	1	0	0.00008
uncertain_significance,not_provided	1	0	0.00018	0	0	0.00000
NA	0	0	0.00000	1	0	0.00008
NA	0	0	0.00000	1	0	0.00008
NA	1	0	0.00018	0	0	0.00000
NA	0	0	0.00000	1	0	0.00008
uncertain_significance	0	0	0.00000	2	0	0.00015
uncertain_significance	17	0	0.00310	47	0	0.00359
NA	1	0	0.00018	0	0	0.00000
not_provided,benign	65	0	0.01184	155	0	0.01184
NA	0	0	0.00000	1	0	0.00008
NA	0	0	0.00000	1	0	0.00008
benign	1	0	0.00018	2	0	0.00015
NA	0	0	0.00000	1	0	0.00008
NA	0	0	0.00000	2	0	0.00015
NA	0	0	0.00000	1	0	0.00008
NA	0	0	0.00000	2	0	0.00015
NA	0	0	0.00000	1	0	0.00008
pathogenic	1	0	0.00018	7	0	0.00053
uncertain_significance	3	0	0.00055	8	0	0.00061
uncertain_significance	0	0	0.00000	3	0	0.00023
NA	1	0	0.00018	0	0	0.00000
NA	0	0	0.00000	1	0	0.00008
NA	0	0	0.00000	1	0	0.00008
NA	0	0	0.00000	2	0	0.00015
NA	0	0	0.00000	1	0	0.00008
uncertain_significance	0	0	0.00000	1	0	0.00008
uncertain_significance	0	0	0.00000	1	0	0.00008
benign,likely_benign	0	0	0.00000	3	0	0.00023
benign,likely_benign	0	0	0.00000	1	0	0.00008
NA	1	0	0.00018	1	0	0.00008
not_provided,likely_benign	44	0	0.00802	114	0	0.00871
NA	1	0	0.00018	2	0	0.00015
likely_benign	13	0	0.00237	23	0	0.00176
NA	0	0	0.00000	1	0	0.00008
likely_benign	0	0	0.00000	1	0	0.00008
benign	2271	303	0.41381	5471	755	0.41805
NA	1	0	0.00018	1	0	0.00008
benign	3	0	0.00055	8	0	0.00061
NA	1	0	0.00018	0	0	0.00000
uncertain_significance,pathogenic	1	0	0.00018	0	0	0.00000
NA	1	0	0.00018	0	0	0.00000
NA	0	0	0.00000	3	0	0.00023
NA	1	0	0.00018	1	0	0.00008
NA	0	0	0.00000	1	0	0.00008
NA	0	0	0.00000	2	0	0.00015
NA	0	0	0.00000	2	0	0.00015
likely_benign	3	0	0.00055	3	0	0.00023
NA	0	0	0.00000	1	0	0.00008
uncertain_significance	0	0	0.00000	2	0	0.00015
pathogenic	0	0	0.00000	3	0	0.00023
uncertain_significance	1	0	0.00018	5	0	0.00038
uncertain_significance	1	0	0.00018	0	0	0.00000
NA	0	0	0.00000	2	0	0.00015
NA	1	0	0.00018	0	0	0.00000
uncertain_significance	0	0	0.00000	1	0	0.00008
pathogenic	0	0	0.00000	1	0	0.00008
likely_benign	0	0	0.00000	2	0	0.00015
likely_benign	0	0	0.00000	1	0	0.00008
uncertain_significance	2	0	0.00036	5	0	0.00038
NA	0	0	0.00000	1	0	0.00008
NA	0	0	0.00000	3	0	0.00023
likely_benign	1	0	0.00018	1	0	0.00008
likely_benign	0	0	0.00000	1	0	0.00008
NA	1	0	0.00018	0	0	0.00000
uncertain_significance	0	0	0.00000	1	0	0.00008
NA	0	0	0.00000	1	0	0.00008
likely_benign	1	0	0.00018	2	0	0.00015
NA	0	0	0.00000	2	0	0.00015
NA	0	0	0.00000	1	0	0.00008
uncertain_significance	1	0	0.00018	1	0	0.00008
likely_benign	2	0	0.00036	1	0	0.00008
NA	0	0	0.00000	1	0	0.00008
pathogenic	0	0	0.00000	6	0	0.00046
not_provided,benign	346	1	0.06305	803	11	0.06136
NA	0	0	0.00000	1	0	0.00008
uncertain_significance,not_provided	1	0	0.00018	2	0	0.00015
NA	0	0	0.00000	1	0	0.00008
NA	0	0	0.00000	1	0	0.00008
NA	0	0	0.00000	1	0	0.00008
uncertain_significance	0	0	0.00000	1	0	0.00008
NA	2	0	0.00036	0	0	0.00000
uncertain_significance	0	0	0.00000	1	0	0.00008
uncertain_significance	0	0	0.00000	1	0	0.00008
NA	0	0	0.00000	1	0	0.00008
NA	1	0	0.00018	0	0	0.00000
NA	0	0	0.00000	1	0	0.00008
NA	7	0	0.00128	7	0	0.00053
NA	2	0	0.00036	1	0	0.00008
benign	0	0	0.00000	8	0	0.00061
NA	0	0	0.00000	1	0	0.00008
benign	923	41	0.16819	2136	89	0.16322
NA	0	0	0.00000	1	0	0.00008
uncertain_significance	0	0	0.00000	2	0	0.00015
likely_benign	1	0	0.00018	2	0	0.00015
benign	61	1	0.01112	154	1	0.01177
NA	0	0	0.00000	1	0	0.00008
pathogenic	0	0	0.00000	1	0	0.00008
NA	0	0	0.00000	1	0	0.00008
likely_benign	7	0	0.00128	12	0	0.00092
NA	1	0	0.00018	1	0	0.00008
NA	1	0	0.00018	0	0	0.00000
NA	0	0	0.00000	1	0	0.00008
NA	1	0	0.00018	3	0	0.00023
uncertain_significance	0	0	0.00000	1	0	0.00008
NA	0	0	0.00000	1	0	0.00008
NA	0	0	0.00000	1	0	0.00008
NA	0	0	0.00000	1	0	0.00008
NA	1	0	0.00018	0	0	0.00000
NA	0	0	0.00000	1	0	0.00008
likely_benign	0	0	0.00000	1	0	0.00008
uncertain_significance	5	0	0.00091	4	0	0.00031
uncertain_significance	1	0	0.00018	0	0	0.00000
pathogenic	0	0	0.00000	2	0	0.00015
benign	14	0	0.00255	42	0	0.00321
uncertain_significance	1	0	0.00018	5	0	0.00038
likely_benign	0	0	0.00000	2	0	0.00015
NA	0	0	0.00000	1	0	0.00008
uncertain_significance	1	0	0.00018	1	0	0.00008
NA	0	0	0.00000	1	0	0.00008
uncertain_significance	0	0	0.00000	1	0	0.00008
NA	0	0	0.00000	1	0	0.00008
NA	1	0	0.00018	2	0	0.00015
uncertain_significance	0	0	0.00000	3	0	0.00023
NA	0	0	0.00000	2	0	0.00015
pathogenic	0	0	0.00000	2	0	0.00015
uncertain_significance	1	0	0.00018	1	0	0.00008
NA	1	0	0.00018	0	0	0.00000
uncertain_significance,not_provided,benign	229	4	0.04173	544	4	0.04157
uncertain_significance	1	0	0.00018	1	0	0.00008
NA	0	0	0.00000	1	0	0.00008
NA	1	0	0.00018	0	0	0.00000
not_provided,benign	3	0	0.00055	2	0	0.00015
NA	0	0	0.00000	1	0	0.00008
likely_benign	0	0	0.00000	2	0	0.00015
uncertain_significance,not_provided	0	0	0.00000	1	0	0.00008
pathogenic	0	0	0.00000	2	0	0.00015
NA	0	0	0.00000	1	0	0.00008
NA	0	0	0.00000	1	0	0.00008
uncertain_significance	0	0	0.00000	1	0	0.00008
NA	0	0	0.00000	1	0	0.00008
likely_benign	1	0	0.00018	2	0	0.00015
pathogenic	0	0	0.00000	2	0	0.00015
uncertain_significance	1	0	0.00018	1	0	0.00008
NA	0	0	0.00000	2	0	0.00015
uncertain_significance	0	0	0.00000	1	0	0.00008
not_provided,likely_benign	0	0	0.00000	1	0	0.00008
NA	1	0	0.00000	0	0	0.00008
uncertain_significance	2	0	0.00036	9	0	0.00069
benign	0	0	0.00000	8	0	0.00061
pathogenic	0	0	0.00000	5	0	0.00038
NA	0	0	0.00000	1	0	0.00008
uncertain_significance	0	0	0.00000	1	0	0.00008
NA	0	0	0.00000	1	0	0.00008
NA	0	0	0.00000	1	0	0.00008
NA	0	0	0.00000	1	0	0.00008
NA	0	0	0.00000	2	0	0.00015
pathogenic	0	0	0.00000	1	0	0.00008
uncertain_significance	0	0	0.00000	1	0	0.00008
pathogenic	0	0	0.00000	1	0	0.00008
NA	0	0	0.00000	1	0	0.00008
NA	1	0	0.00018	0	0	0.00000
NA	0	0	0.00000	1	0	0.00008
NA	0	0	0.00000	1	0	0.00008
NA	0	0	0.00000	1	0	0.00008
uncertain_significance	1	0	0.00018	2	0	0.00015
NA	1	0	0.00018	0	0	0.00000
uncertain_significance,likely_benign	0	0	0.00000	3	0	0.00023
uncertain_significance	0	0	0.00000	1	0	0.00008
uncertain_significance	0	0	0.00000	6	0	0.00046
uncertain_significance	1	0	0.00018	1	0	0.00008
NA	0	0	0.00000	1	0	0.00008
NA	0	0	0.00000	1	0	0.00008
pathogenic	1	0	0.00018	3	0	0.00023
likely_benign	2	0	0.00036	2	0	0.00015
uncertain_significance,not_provided	2	0	0.00036	6	0	0.00046
NA	0	0	0.00000	1	0	0.00008
NA	0	0	0.00000	2	0	0.00015
uncertain_significance	1	0	0.00018	2	0	0.00015
NA	0	0	0.00000	1	0	0.00008
NA	0	0	0.00000	1	0	0.00008

uncertain_significance,not_provided	1	0	0.00018	6	0	0.00046
likely_benign	0	0	0.00000	1	0	0.00008
NA	1	0	0.00018	0	0	0.00000
NA	0	0	0.00000	1	0	0.00008
not_provided,benign	2	0	0.00036	2	0	0.00015
NA	1	0	0.00018	5	0	0.00038
NA	0	0	0.00000	1	0	0.00008
NA	1	0	0.00018	0	0	0.00000
NA	0	0	0.00000	1	0	0.00008
NA	0	0	0.00000	6	0	0.00046
NA	1	0	0.00018	0	0	0.00000
likely_benign	0	0	0.00000	1	0	0.00008
NA	0	0	0.00000	1	0	0.00008
NA	0	0	0.00000	4	0	0.00031
NA	0	0	0.00000	1	0	0.00008
uncertain_significance	0	0	0.00000	1	0	0.00008
NA	0	0	0.00000	1	0	0.00008
uncertain_significance	0	0	0.00000	1	0	0.00008
likely_benign	2	0	0.00036	12	0	0.00092
uncertain_significance	1	0	0.00018	1	0	0.00008
NA	0	0	0.00000	1	0	0.00008
NA	3	0	0.00055	33	1	0.00252
NA	0	0	0.00000	1	0	0.00008
uncertain_significance	0	0	0.00000	1	0	0.00008
uncertain_significance	0	0	0.00000	2	0	0.00015
NA	0	0	0.00000	1	0	0.00008
uncertain_significance	0	0	0.00000	2	0	0.00015
NA	1	0	0.00018	0	0	0.00000
NA	0	0	0.00000	1	0	0.00008
NA	0	0	0.00000	1	0	0.00008
NA	1	0	0.00018	0	0	0.00000
NA	0	0	0.00000	1	0	0.00008
uncertain_significance	0	0	0.00000	1	0	0.00008
NA	0	0	0.00000	1	0	0.00008
NA	1	0	0.00018	2	0	0.00015
uncertain_significance	0	0	0.00000	1	0	0.00008
NA	1	0	0.00018	0	0	0.00000
NA	0	0	0.00000	1	0	0.00008
uncertain_significance	3	0	0.00055	2	0	0.00015
NA	0	0	0.00000	1	0	0.00008
NA	0	0	0.00000	1	0	0.00008
NA	0	0	0.00000	1	0	0.00008
NA	0	0	0.00000	1	0	0.00008
NA	1	0	0.00018	0	0	0.00000
NA	951	42	0.17329	2039	108	0.15580
NA	0	0	0.00000	1	0	0.00008
NA	0	0	0.00000	1	0	0.00008
NA	0	0	0.00000	1	0	0.00008
NA	0	0	0.00000	1	0	0.00008
benign	0	0	0.00000	1	0	0.00008
uncertain_significance	1	0	0.00018	2	0	0.00015
uncertain_significance	1	0	0.00018	0	0	0.00000
NA	0	0	0.00000	2	0	0.00015
uncertain_significance	0	0	0.00000	8	0	0.00061
NA	0	0	0.00000	1	0	0.00008
benign	0	0	0.00000	2	0	0.00015
NA	0	0	0.00000	1	0	0.00008
NA	1	0	0.00018	0	0	0.00000
NA	0	0	0.00000	1	0	0.00008
NA	1	0	0.00018	0	0	0.00000
uncertain_significance	5	0	0.00091	15	0	0.00115
benign	2	0	0.00036	5	0	0.00038
uncertain_significance	0	0	0.00000	1	0	0.00008
NA	1	0	0.00018	0	0	0.00000
NA	1	0	0.00018	0	0	0.00000
NA	0	0	0.00000	1	0	0.00008
NA	0	0	0.00000	1	0	0.00008
NA	1	0	0.00018	0	0	0.00000
NA	2	0	0.00036	0	0	0.00000
uncertain_significance	0	0	0.00000	2	0	0.00015
NA	0	0	0.00000	2	0	0.00015
NA	1	0	0.00018	0	0	0.00000
NA	0	0	0.00000	1	0	0.00008
NA	0	0	0.00000	1	0	0.00008
NA	0	0	0.00000	1	0	0.00008
NA	0	0	0.00000	2	0	0.00015
NA	0	0	0.00000	1	0	0.00008
NA	0	0	0.00000	1	0	0.00008
NA	0	0	0.00000	3	0	0.00023
NA	1	0	0.00018	3	0	0.00023
NA	0	0	0.00000	1	0	0.00008
NA	0	0	0.00000	1	0	0.00008
NA	0	0	0.00000	1	0	0.00008
NA	0	0	0.00000	1	0	0.00008
NA	0	0	0.00000	1	0	0.00008
NA	1	0	0.00018	1	0	0.00008
NA	1	0	0.00018	0	0	0.00000
NA	0	0	0.00000	1	0	0.00008
NA	0	0	0.00000	2	0	0.00015
