# Supplementary material for: Rare, protein-truncating variants in ATM, CHEK2 and PALB2, but not XRCC2, are associated with increased breast cancer risks
Source: J Med Genet. 2017 Aug 4;54(11):732–41. doi: 10.1136/jmedgenet-2017-104588 (PMC5740532; doi:10.1136/jmedgenet-2017-104588)
Supplement: Supplementary data [file jmedgenet-2017-104588supp004.pdf]

Table S4. Tally of variants by primary annotation category and frequency.

Total Number of Variants Discovered by Gene and Type											
	Canonical Splice*	Frame Shift*	Inframe	Intronic	Missense	Regulatory	Splice Site	Stop Gained*	Synonymous	UTR	Total
**ATM**	10	25	4	230	324	6	43	24	119	0	**785**
**CHEK2**	2	6	0	40	77	8	11	7	11	3	**165**
**PALB2**	0	18	1	28	125	11	8	16	48	0	**255**
**XRCC2**	2	4	0	0	34	13	1	5	8	1	**68**
**Total**	**14**	**53**	**5**	**298**	**560**	**38**	**63**	**52**	**186**	**4**	**1273**

Variants Classified as Common (Carrier Frequency >0.1%) by Gene and Type											
	Canonical Splice*	Frame Shift*	Inframe	Intronic	Missense	Regulatory	Splice Site	Stop Gained*	Synonymous	UTR	Total
**ATM**	0	0	0	10	25	0	4	0	11	0	**50**
**CHEK2**	0	1	0	0	4	0	0	0	1	0	**6**
**PALB2**	0	0	0	2	7	0	0	0	4	0	**13**
**XRCC2**	0	0	0	0	2	0	0	0	0	1	**3**
**Total**	**0**	**1**	**0**	**12**	**38**	**0**	**4**	**0**	**16**	**1**	**72**

Variants Classifed as Rare (Carrier Frequency <0.1%) by Gene and Type											
	Canonical Splice*	Frame Shift*	Inframe	Intronic	Missense	Regulatory	Splice Site	Stop Gained*	Synonymous	UTR	Total
**ATM**	10	25	4	220	299	6	39	24	108	0	**735**
**CHEK2**	2	5	0	40	73	8	11	7	10	3	**159**
**PALB2**	0	18	1	26	118	11	8	16	44	0	**242**
**XRCC2**	2	4	0	0	32	13	1	5	8	0	**65**
**Total**	**14**	**52**	**5**	**286**	**522**	**38**	**59**	**52**	**170**	**3**	**1201**

*Truncating variants
