# Supplementary material for: Rare, protein-truncating variants in ATM, CHEK2 and PALB2, but not XRCC2, are associated with increased breast cancer risks
Source: J Med Genet. 2017 Aug 4;54(11):732–41. doi: 10.1136/jmedgenet-2017-104588 (PMC5740532; doi:10.1136/jmedgenet-2017-104588)
Supplement: Supplementary data [file jmedgenet-2017-104588supp006.pdf]

Table S6. Risk estimates of overall breast cancer and estrogen-receptor subtypes for truncating variants using ExAC controls.

		ExAC European		ExAC All	
		OR (95% CI)	P-Value	OR (95% CI)	P-Value
ATM	Overall	**2.18 (1.63-2.91)**	**1.1E-07**	**2.62 (2.01-3.42)**	**3.8E-13**
	ER Positive	**2.13 (1.52-3.00)**	**1.3E-05**	**2.57 (1.86-3.54)**	**4.4E-09**
	ER Negative	0.86 (0.32-2.34)	1.0	1.03 (0.38-2.79)	0.80
CHEK2	Overall	**2.73 (2.25-3.31)**	**2.2E-16**	**3.34 (2.80-3.99)**	**2.2E-16**
	ER Positive	**2.99 (2.41-3.73)**	**2.2E-16**	**3.67 (3.00-4.49)**	**2.2E-16**
	ER Negative	1.39 (0.19-2.45)	0.32	1.70 (0.98-2.97)	0.087
PALB2	Overall	**5.30 (3.69-7.64)**	**2.2E-16**	**4.11 (3.09-5.48)**	**2.2E-16**
	ER Positive	**4.27 (2.80-6.52)**	**6.7E-13**	**3.32 (2.32-4.74)**	**8.9E-12**
	ER Negative	**5.56 (2.86-10.8)**	**7.7E-08**	**4.32 (2.31-8.06)**	**2.6E-06**
XRCC2	Overall	1.04 (0.48-2.27)	1.0	1.26 (0.60-2.62)	0.69
	ER Positive	1.01 (0.38-2.66)	1.0	1.16 (0.45-2.98)	0.95
	ER Negative	0.98 (0.13-7.25)	1.0	1.17 (0.16-8.60)	0.58
