# Supplementary material for: Rare, protein-truncating variants in ATM, CHEK2 and PALB2, but not XRCC2, are associated with increased breast cancer risks
Source: J Med Genet. 2017 Aug 4;54(11):732–41. doi: 10.1136/jmedgenet-2017-104588 (PMC5740532; doi:10.1136/jmedgenet-2017-104588)
Supplement: Supplementary data [file jmedgenet-2017-104588supp007.pdf]

Table S7. Risk estimates for common missense, synonymous, and non-coding variants.

Gene	VEP Consequences	Amino Acid Substitution	SEACH Case Homozygote Count	SEARCH Case Heterozygote Count	SEARCH Case Non-Carrier Count	SEACH Control Homozygote Count	SEARCH Control Heterozygote Count	SEARCH Control Non-Carrier Count	Odds Ratio	95% CI	Conchran-Armitage P-Trend
CHEK2	**splice,intron**	**NA**	**0**	**33**	**13054**	**0**	**1**	**5487**	**13.9**	**1.89-101**	**6.7E-04**
ATM	**missense**	**C2464R**	**0**	**16**	**13071**	**0**	**18**	**5470**	**0.37**	**0.19-0.73**	**2.8E-03**
XRCC2	**3_prime_UTR**	**NA**	**1**	**32**	**13054**	**0**	**3**	**5485**	**4.76**	**1.46-15.5**	**5.3E-03**
XRCC2	**missense**	**R188H**	**108**	**1931**	**11048**	**42**	**909**	**4537**	**0.90**	**0.83-0.97**	**8.0E-03**
CHEK2	**3_prime_UTR**	**NA**	**0**	**34**	**13053**	**0**	**5**	**5483**	**2.85**	**1.12-7.30**	**0.022**
ATM	**splice,intron**	**NA**	**0**	**85**	**13002**	**0**	**21**	**5467**	**1.70**	**1.05-2.74**	**0.028**
PALB2	**synonymous**	**NA**	**0**	**57**	**13030**	**0**	**13**	**5475**	**1.84**	**1.01-3.36**	**0.044**
ATM	missense	K1454N	0	15	13072	0	1	5487	6.29	0.83-47.6	0.052
ATM	missense	L2307F	0	15	13072	0	1	5487	6.29	0.83-47.6	0.052
ATM	missense	A1309T	0	40	13047	0	27	5461	0.62	0.38-1.01	0.053
ATM	missense	S707P	6	339	12742	3	115	5370	1.22	0.99-1.50	0.064
ATM	missense	Y1442H	0	18	13069	0	14	5474	0.54	0.27-1.08	0.078
CHEK2	intron	NA	0	54	13033	0	33	5455	0.69	0.44-1.05	0.086
ATM	missense	S333F	0	46	13041	0	11	5477	1.75	0.91-3.39	0.089
CHEK2	intron	NA	5147	6054	1886	2201	2551	736	0.96	0.91-1.01	0.11
ATM	synonymous	NA	0	62	13025	0	17	5471	1.53	0.89-2.62	0.12
ATM	missense	H1380Y	0	25	13062	0	5	5483	2.10	0.80-5.48	0.12
ATM	intron	NA	0	25	13062	0	5	5483	2.10	0.80-2.48	0.12
ATM	upstream	NA	0	25	13062	0	5	5483	2.10	0.80-2.48	0.12
ATM	intron	NA	0	11	13076	0	9	5479	0.51	0.21-1.24	0.13
ATM	intron	NA	0	31	13056	0	7	5481	1.86	0.82-4.22	0.13
ATM	missense	D1853V	0	133	12954	0	43	5445	1.29	0.92-1.83	0.14
CHEK2	missense	S428F	0	2	13085	0	3	5485	0.28	1.15-1.67	0.14
ATM	missense	D1853N	253	3231	9603	120	1392	3976	0.95	0.90-1.02	0.14
ATM	missense	Y1475C	0	36	13051	0	22	5466	0.69	0.40-1.17	0.16
CHEK2	missense	I157T	0	20	13067	0	4	5484	2.10	0.72-6.14	0.17
ATM	intron,feature_truncation	NA	252	3230	9605	119	1388	3981	0.96	0.90-1.02	0.17
ATM	intron	NA	2	232	12853	0	115	5373	0.86	0.69-1.08	0.18
ATM	missense	S1691R	0	53	13034	0	29	5459	0.77	0.49-1.21	0.25
PALB2	missense	G998E	4	587	12496	0	270	5218	0.92	0.80-1.07	0.27
ATM	synonymous	NA	0	32	13055	0	9	5479	1.49	0.71-3.12	0.29
ATM	missense	P604S	0	34	13053	0	19	5469	0.75	0.43-1.32	0.31
ATM	intron	NA	12	426	12649	3	167	5318	1.09	0.92-1.30	0.33
ATM	missense	V182L	0	16	13071	0	4	5484	1.68	0.56-5.02	0.35
PALB2	missense	Q559R	89	2047	10951	41	882	4565	0.96	0.89-1.04	0.37
ATM	intron,feature_truncation	NA	0	29	13058	0	16	5472	0.76	0.41-1.40	0.38
CHEK2	synonymous	NA	13	642	12432	3	257	5228	1.07	0.92-1.23	0.38
PALB2	downstream	NA	0	23	13064	0	13	5475	0.74	0.38-1.46	0.39
ATM	intron	NA	2	254	12831	0	98	5390	1.11	0.87-1.40	0.40
ATM	synonymous	NA	0	40	13047	0	13	5475	1.29	0.69-2.41	0.42
ATM	missense	V2079I	1	16	13070	0	5	5483	1.51	0.56-4.07	0.43
PALB2	synonymous	NA	0	42	13045	0	14	5474	1.26	0.69-2.31	0.46
ATM	missense	D126E	0	39	13048	0	13	5475	1.25	0.67-2.36	0.47
ATM	intron	NA	0	39	13048	0	13	5475	1.26	0.67-2.36	0.47
ATM	intron	NA	0	39	13048	0	13	5475	1.26	0.67-2.36	0.47
PALB2	intron	NA	755	4716	7616	303	1968	3217	1.02	0.97-1.07	0.49
ATM	intron	NA	0	36	13051	0	12	5476	1.26	0.65-2.42	0.49
ATM	missense	F1463C	0	14	13073	0	4	5484	1.47	0.48-4.46	0.50
ATM	intron	NA	0	38	13049	0	13	5475	1.23	0.65-2.30	0.53
ATM	missense	G2023R	0	45	13042	0	22	5466	0.85	0.51-1.43	0.55
CHEK2	missense	R180C	0	24	13063	0	8	5480	1.26	0.57-2.80	0.57
ATM	splice,intron	NA	0	77	13010	0	36	5452	0.90	0.60-1.33	0.59
PALB2	missense	L939W	0	47	13040	0	17	5471	1.16	0.67-2.02	0.60
ATM	synonymous	NA	0	24	13063	0	12	5476	0.84	0.42-1.68	0.62
ATM	missense	F858L	8	388	12691	1	175	5312	0.96	0.80-1.14	0.63
PALB2	missense	P864S	0	114	12973	0	44	5444	1.09	0.77-1.54	0.64
XRCC2	missense	I95V	0	15	13072	0	5	5483	1.26	0.46-3.46	0.66
ATM	splice,intron	NA	6	837	12244	6	335	5147	1.03	0.90-1.17	0.68
ATM	missense	S978P	0	25	13062	0	9	5479	1.16	0.54-2.50	0.69
ATM	missense	V410A	1	73	13013	0	29	5459	1.08	0.71-1.67	0.71
ATM	missense	L1420F	2	485	12600	6	199	5283	0.97	0.82-1.14	0.73
PALB2	synonymous	NA	1	153	12933	1	60	5427	1.05	0.78-1.41	0.75
ATM	regulatory	NA	1	139	12947	0	62	5426	0.95	0.71-1.29	0.76
PALB2	synonymous	NA	11	792	12284	1	346	5141	0.98	0.86-1.11	0.76
ATM	synonymous	NA	0	76	13011	0	30	5458	1.06	0.70-1.62	0.78
PALB2	missense	E672Q	11	792	12284	1	345	5142	0.98	0.87-1.12	0.79
CHEK2	intron,feature_elongation	NA	363	3580	9144	159	1476	3853	1.01	0.95-1.07	0.80
ATM	missense	F582L	0	6	13081	0	3	5485	0.83	0.21-3.35	0.80
ATM	synonymous	NA	0	98	12989	0	43	5445	0.96	0.67-1.37	0.80
PALB2	intron	NA	0	15	13072	0	7	5481	0.90	0.37-2.20	0.82
PALB2	missense	L337S	4	540	12543	4	225	5259	0.99	0.84-1.15	0.86
ATM	upstream	NA	1	195	12891	0	84	5404	0.98	0.76-1.27	0.90
ATM	synonymous	NA	1	195	12891	0	84	5404	0.98	0.76-1.27	0.90
ATM	missense	S49C	4	370	12713	1	158	5329	0.99	0.82-1.19	0.92
ATM	synonymous	NA	33	1247	11807	18	517	4953	1.00	0.90-1.10	0.93
ATM	splice,intron	NA	29	1120	11938	14	464	5010	1.00	0.90-1.12	0.94
ATM	intron	NA	4378	6323	2386	1827	2665	996	1.00	0.96-1.05	0.94
ATM	intron	NA	4225	6369	2493	1777	2664	1047	1.00	0.95-1.04	0.95
ATM	missense	P1054R	14	735	12338	2	315	5171	1.00	0.88-1.15	0.96
ATM	synonymous	NA	3	329	12755	2	136	5350	1.00	0.82-1.22	0.97
ATM	downstream	NA	0	134	12953	1	54	5433	1.00	0.73-1.37	0.98
ATM	intron	NA	4376	6329	2382	1830	2664	994	1.00	0.96-1.05	1.00
PALB2	missense	V932M	0	155	12932	0	65	5423	1.00	0.75-1.34	1.00
CHEK2	missense	Y424H	0	2	13085	0	0	5488	NA	NA	NA

Chromosome	Position	Reference Allele	Alternate Allele	Known Variation	CADD Scaled Score	PolyPhen2 Category	SIFT Category	ExAC Non-Finnish European Carrier Frequency
**22**	**29121360**	**A**	**T**	**CS086685,rs121908700**	**12.02**	**NA**	**NA**	**0**
**11**	**108201023**	**T**	**C**	**CM016183,rs55801750,COSM758329**	**21.9**	**possibly_damaging**	**tolerated**	**0.00204**
**7**	**152345726**	**A**	**G**	**rs3218539**	**12.68**	**NA**	**NA**	**0**
**7**	**152346007**	**C**	**T**	**rs3218536,CM021368**	**15.95**	**benign**	**tolerated**	**0.15267**
**22**	**29083867**	**G**	**A**	**rs17884403**	**6.344**	**NA**	**NA**	**0**
**11**	**108119654**	**T**	**G**	**rs201686625,CS000846,COSM758349**	**11.93**	**NA**	**NA**	**0**
**16**	**23614846**	**C**	**T**	**rs45439097**	**18.52**	**NA**	**NA**	**0.002157885**
11	108160454	A	C	CM0910503,rs148993589,COSM22501	15.75	benign	tolerated	0.00134
11	108196896	C	T	rs56009889	31	probably_damaging	deleterious	0.00384
11	108155132	G	A	rs149711770,COSM22507	20.6	benign	tolerated	0.00183
11	108124761	T	C	rs4986761,CM013692,COSM41595	15.81	benign	tolerated	0.02192
11	108160416	T	C	rs201666889,CM0910502	27.3	probably_damaging	deleterious	0.00150
22	29121207	G	A	rs121908699	2.665	NA	NA	0
11	108117787	C	T	rs28904919	25.9	possibly_damaging	deleterious	0.00280
22	29091037	G	C	rs5762749	5.976	NA	NA	0
11	108114792	C	T	rs144709948,CM0910480	14.94	NA	NA	0.004376037
11	108159732	C	T	CM021944,rs3092856,COSM24627	6.964	benign	tolerated	0.00123
11	108114632	T	G	rs2235001	0.205	NA	NA	0
11	108139120	G	T	rs2234994	6.735	NA	NA	0
11	108218101	T	G	rs200190537	5.503	NA	NA	0
11	108170698	C	T	rs531375734	3.042	NA	NA	0
11	108175463	A	T	rs1801673,CM083593,COSM1350895,COSM21628,COSM1350894,COSM3752120	23.6	benign	deleterious	0.01384
22	29091207	G	A	CM051021,rs137853011,COSM2935967	29.8	probably_damaging	deleterious	0.00111
11	108175462	G	A	CM077896,rs1801516,COSM41596,COSM3736031	23.2	benign	tolerated	0.26659
11	108160516	A	G	rs34640941,COSM22503	18.12	benign	tolerated	0.00153
22	29121087	A	G	CM993368,rs17879961,COSM3693990	21.1	benign	tolerated	0.00947
11	108150207	CT	C	rs1799757	7.431	NA	NA	0
11	108225473	G	A	rs139490310	2.938	NA	NA	0
11	108170506	A	C	CM980141,rs1800059	11.03	benign	tolerated	0.00623
16	23634293	C	T	rs45551636,CM098533	33	probably_damaging	deleterious	0.04238
11	108121446	A	G	rs4987943	0.816	NA	NA	0.001533695
11	108123551	C	T	CM045888,rs2227922,COSM1350778,COSM22499,COSM1350777	24.3	possibly_damaging	tolerated	0.00742
11	108151688	T	A	rs148368017	7.549	NA	NA	0
11	108114727	G	C	CM035638,rs3218707	12.39	benign	tolerated	0.00223
16	23646191	T	C	rs152451	0.001	benign	tolerated	0.18324
11	108150364	TATC	T	rs545376366	7.454	NA	NA	0
22	29130458	T	C	rs1805129,COSM149275	2.59	NA	NA	0.056860804
16	23637756	G	C	rs180177119	1.441	NA	NA	0
11	108155240	G	A	rs3218709	5.965	NA	NA	0
11	108218013	C	T	rs56025670	9.667	NA	NA	0.001397072
11	108188136	G	A	rs1800060	0.005	benign	tolerated	0.00243
16	23646673	C	T	rs61755173	11.39	NA	NA	0.002308915
11	108106443	T	A	rs587781449,rs2234997,CM077897,COSM22498	4.635	benign	tolerated	0.00324
11	108115778	T	G	rs1800735	5.574	NA	NA	0
11	108165634	A	G	rs3218678	2.868	NA	NA	0
16	23640467	G	A	rs249954	5.159	NA	NA	0
11	108170680	A	G	rs558610972	6.493	NA	NA	0
11	108160480	T	G	CM022199,rs138327406,COSM22502,COSM1350855	28.7	probably_damaging	deleterious	0.00446
11	108170373	T	G	rs3092906	8.257	NA	NA	0
11	108186610	G	A	rs11212587,CM092585,COSM24635	31	probably_damaging	deleterious	0.00465
22	29121019	G	A	CM030417,rs77130927	23.2	benign	tolerated	0.00342
11	108202772	G	T	CS014123,rs112775908	8.104	NA	NA	0
16	23635348	A	C	CM105609,rs45478192	28	probably_damaging	deleterious	0.00318
11	108124628	T	C	rs1800055	0.337	NA	NA	0.001439108
11	108138003	T	C	CM061641,rs1800056,COSM21826	8.823	benign	tolerated	0.02903
16	23637715	G	A	CM105610,rs45568339	18.44	benign	tolerated	0.00806
7	152346287	T	C	rs140214637	0.001	benign	tolerated	0.00117
11	108175394	T	C	rs3092829	3.28	NA	NA	0
11	108141988	T	C	rs139552233,COSM922715,COSM21828,COSM1585357	26.8	possibly_damaging	deleterious	0.00147
11	108119823	T	C	CM035782,rs56128736,COSM1350756,COSM21825,COSM1350755,COSM1350754	23.2	benign	deleterious	0.00649
11	108160350	C	T	CM000653,rs1800058	15.68	benign	tolerated	0.03759
16	23646295	T	C	rs45472400	0.365	NA	NA	0.008181001
11	108190627	T	C	rs116924981	2.495	NA	NA	0
16	23619235	A	C	rs45516100	0.079	NA	NA	0.055712059
11	108163382	C	T	rs4988008	16.65	NA	NA	0.004405682
16	23641461	C	G	CM112099,rs45532440	8.057	benign	tolerated	0.05574
22	29130347	G	GT	rs3841692	2.024	NA	NA	0
11	108122700	T	C	CM023329,rs2235006	3.12	benign	tolerated	0.00201
11	108098592	T	C	rs3218690	0.051	NA	NA	0.00498304
16	23634273	A	G	rs180177128	18.26	NA	NA	0
16	23646857	A	G	rs45494092,CM112098	5.275	benign	tolerated	0.03808
11	108139364	T	TA	rs2234995	3.857	NA	NA	0
11	108180917	T	C	rs3092910	7.568	NA	NA	0.012627022
11	108098576	C	G	CM990201,rs1800054	25.1	possibly_damaging	deleterious	0.02247
11	108163487	C	T	rs1800889	12.84	NA	NA	0.092004316
11	108224615	A	C	rs4986839	1.963	NA	NA	0
11	108143182	C	T	rs664677	4.058	NA	NA	0
11	108225483	C	T	rs664982	2.77	NA	NA	0
11	108143456	C	G	CM973365,rs1800057,COSM21827	26.5	probably_damaging	deleterious	0.05150
11	108115587	C	T	rs3218674	11.67	NA	NA	0.031149753
11	108175387	G	C	rs3092828	0.772	NA	NA	0
11	108225661	A	G	rs664143	0.543	NA	NA	0
16	23635370	C	T	rs45624036,CM112100	26.6	probably_damaging	tolerated	0.01617
22	29091220	A	G	rs139366548	28.7	probably_damaging	deleterious	0.00100
