# Supplementary material for: Rare, protein-truncating variants in ATM, CHEK2 and PALB2, but not XRCC2, are associated with increased breast cancer risks
Source: J Med Genet. 2017 Aug 4;54(11):732–41. doi: 10.1136/jmedgenet-2017-104588 (PMC5740532; doi:10.1136/jmedgenet-2017-104588)
Supplement: Supplementary data [file jmedgenet-2017-104588supp008.pdf]

Table S8. Risk estimates for rare missense variants grouped by CADD, PolyPhen2, SIFT, and AlignGVGD predictions of deleteriousness.

	ATM					*CHEK2*					*PALB2*					Combined		
	**Case Carriers**	**Control Carriers**	**OR (95%CI)**	**P**	**P_{diff}**	**Case Carriers**	**Control Carriers**	**OR (95%CI)**	**P**	**P_{diff}**	**Case Carriers**	**Control Carriers**	**OR (95%CI)**	**P**	**P_{diff}**	**OR (95%CI)**	**P**	**P_{diff}**
**CADD <20***	230	82	1.18 (0.91-1.52)	0.23	1.00	18	7	1.08 (0.45-2.58)	1.00	0.76	98	31	1.33 (0.89-1.99)	0.20	0.92	1.21 (0.98-1.50)	0.077	0.91
**CADD >20***	259	93	1.17 (0.92-1.49)	0.22		144	43	1.41 (1.00-1.98)	0.058		76	26	1.23 (0.78-1.92)	0.43		**1.25 (1.04-1.50)**	**0.018**	
**PolyPhen Benign^**	322	116	1.17 (0.94-1.45)	0.17	0.99	76	26	1.23 (0.79-1.92)	0.43	0.64	97	31	1.31 (0.88-1.97)	0.22	0.98	**1.21 (1.01-1.44)**	**0.039**	0.74
**PolyPhen Damaging^**	167	59	1.19 (0.88-1.60)	0.29		86	24	1.51 (0.96-2.37)	0.094		77	26	1.24 (0.80-1.94)	0.39		**1.28 (1.03-1.59)**	**0.032**	
**SIFT Tolerated~**	310	112	1.16 (0.94-1.45)	0.19	0.96	72	21	1.44 (0.88-2.34)	0.17	0.89	113	32	1.49 (1.00-2.20)	0.059	0.30	**1.27 (1.06-1.52)**	**0.010**	0.71
**SIFT Deleterious~**	179	63	1.19 (0.89-1.59)	0.26		90	29	1.30 (0.86-1.98)	0.25		61	25	1.02 (0.64-1.63)	1.00		1.19 (0.96-1.47)	0.13	
**AlignGVGD <35`**	400	142	1.19 (0.98-1.44)	0.092	0.94	107	37	1.21 (0.83-1.77)	0.35	0.38	163	53	1.29 (0.95-1.77)	0.12	0.77	**1.22 (1.05-1.42)**	**0.011**	0.76
**AlignGVGD ≥35`**	89	33	1.13 (0.76-1.69)	0.61		55	13	1.78 (0.97-3.26)	0.79		11	4	1.15 (0.37-3.62)	1.00		1.30 (0.95-1.80)	0.12	
**C0**	349	122	1.21 (0.82-2.81)	0.23		65	20	1.36 (0.83-2.25)	0.27		147	45	1.37 (0.98-1.92)	0.074		**1.27 (1.07-1.50)**	**6.1E-03**	
**C15**	29	10	1.22 (0.49-2.50)	0.72		25	7	1.50 (0.65-3.47)	0.45		14	6	0.98 (0.38-2.55)	1.0		1.24 (0.77-1.99)	0.44	
**C25**	22	10	0.92 (0.44-1.95)	0.99		17	10	0.71 (0.33-1.56)	0.52		2	2	0.42 (0.06-2.98)	0.59		0.78 (0.46-1.31)	0.42	
**C35**	17	12	0.59 (0.28-1.24)	0.23		10	1	4.20 (0.54-32.8)	0.19		0	1	NA	NA		0.81 (0.42-1.54)	0.63	
**C45**	12	2	2.52 (0.56-11.3)	0.26		1	1	0.42 (0.03-6.70)	1.0		0	0	NA	NA		1.82 (0.52-6.38)	0.42	
**C55**	13	6	0.91 (0.25-2.39)	1.0		4	1	1.68 (0.19-15.0)	1.0		6	2	1.26 (0.25-6.24)	1.0		1.07 (0.50-2.32)	1.0	
**C65**	47	13	1.52 (0.82-2.81)	0.23		40	10	1.68 (0.84-3.36)	0.18		5	1	2.10 (0.24-17.95)	0.68		**1.61 (1.03-2.53)**	**0.046**	

*Variants with a PHRED-scaled CADD score >20 are predicted to be more deleterious than 99% of theoretical substitutions genome-wide (Reference 21)
^PolyPhen2 prediction of "probably damaging" or "possibly damaging" (PolyPhen2 Score >0.5) (Reference 22)
~SIFT prediction of "damaging" (SIFT score <0.05) (Reference 23)
`Variants with AlignGVGD score >=35 are predicted to confer an OR >2.0 (Reference 24)
