# Supplementary material for: Rare, protein-truncating variants in ATM, CHEK2 and PALB2, but not XRCC2, are associated with increased breast cancer risks
Source: J Med Genet. 2017 Aug 4;54(11):732–41. doi: 10.1136/jmedgenet-2017-104588 (PMC5740532; doi:10.1136/jmedgenet-2017-104588)
Supplement: Supplementary data [file jmedgenet-2017-104588supp010.pdf]

Figure S2A

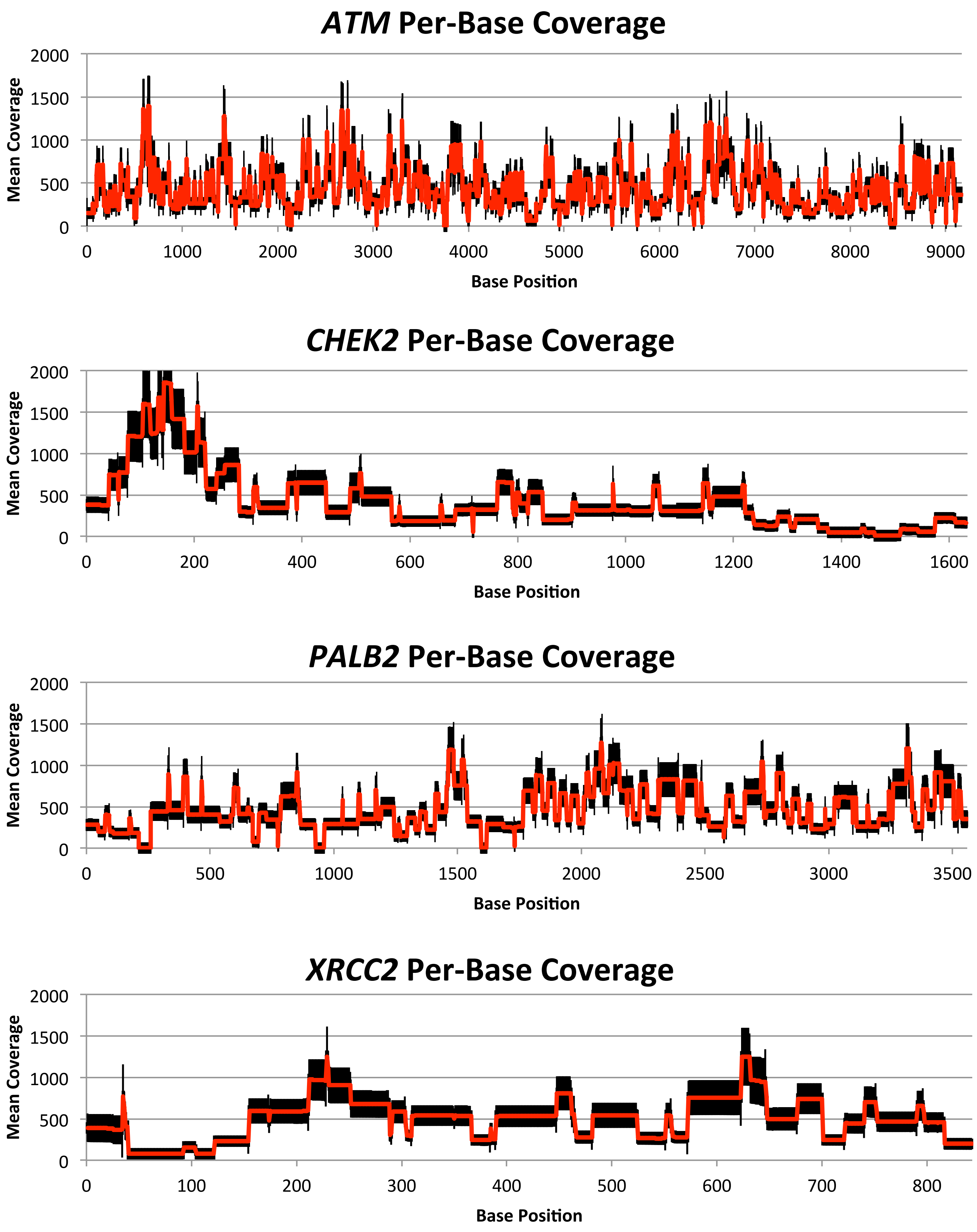
ATM Per-Base Coverage
Mean Coverage
2000
1500
1000
500
0
0
1000
2000
3000
4000
5000
6000
7000
8000
9000
Base Position
CHEK2 Per-Base Coverage
Mean Coverage
2000
1500
1000
500
0
0
200
400
600
800
1000
1200
1400
1600
Base Position
PALB2 Per-Base Coverage
Mean Coverage
2000
1500
1000
500
0
0
500
1000
1500
2000
2500
3000
3500
Base Position
XRCC2 Per-Base Coverage
Mean Coverage
2000
1500
1000
500
0
0
100
200
300
400
500
600
700
800
Base Position

Figure S2B

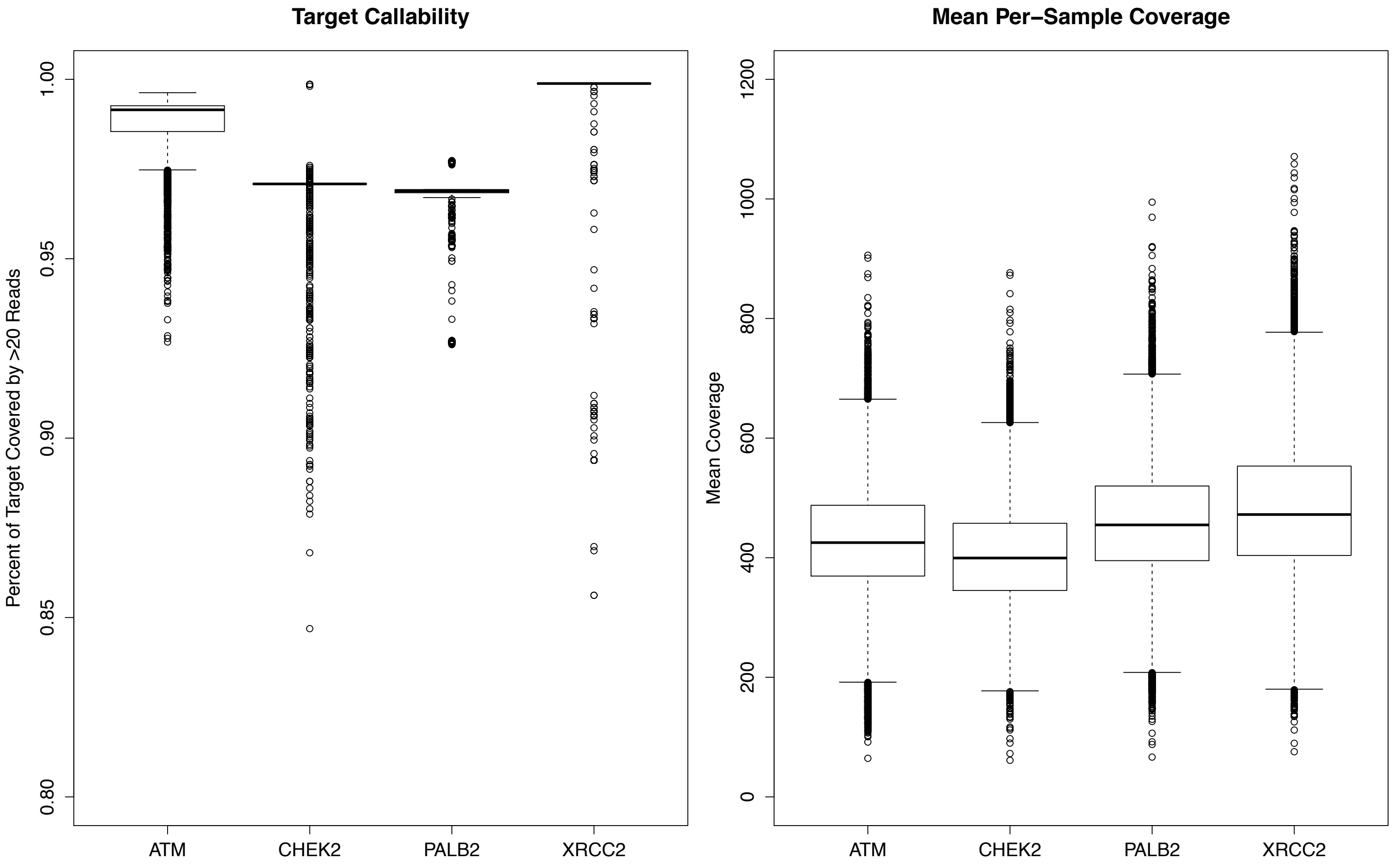

Target Callability
Percent of Target Covered by >20 Reads
1.00
0.95
0.90
0.85
0.80
ATM
CHEK2
PALB2
XRCC2
Mean Per−Sample Coverage
Mean Coverage
1200
1000
800
600
400
200
0
ATM
CHEK2
PALB2
XRCC2
